# The Impact of Secondary Structure on the Base‐Filling of *N*‐Methoxy‐1,3‐Oxazinane (MOANA) and *N*‐Methoxy‐1,3‐Oxazolidine Glycol Nucleic Acid (MOGNA) Oligonucleotides

**DOI:** 10.1002/cbic.202400666

**Published:** 2024-10-27

**Authors:** Mark N. K. Afari, Ninna Heikinmäki, Pasi Virta, Tuomas Lönnberg

**Affiliations:** ^1^ Department of Chemistry University of Turku Henrikinkatu 2 20500 Turku Finland

**Keywords:** Base-filling, Base pairing, Oligonucleotides, Oxazinanes, Oxazolidines

## Abstract

Various single‐stranded and hairpin‐forming DNA and 2'‐*O*‐methyl‐RNA oligonucleotides bearing a single (2*R*,3*S*)‐4‐(methoxyamino)butane‐1,2,3‐triol residue esterified from either O1 and O2 or O1 and O3 were synthesized. Incubation of these oligonucleotides with equimolar mixtures of formylmethyl derivatives of the canonical nucleobases and 2‐methylbenzimidazole under mildly acidic conditions revealed base‐filling of the modified site to be strongly favored by base stacking of a double‐helix, especially an A‐type one. In 2'‐*O*‐methyl‐RNA hairpin oligonucleotides, base‐filling of the (2*R*,3*S*)‐4‐(methoxyamino)butane‐1,2,3‐triol residue with nucleobase aldehydes followed the rules of Watson–Crick base pairing, thymine being the only exception. In single‐stranded oligonucleotides or the Hoogsteen strand of triple helices, both the yield and selectivity of base‐filling were much more modest.

## Graphical abstract

The efficiency and selectivity of base‐filling of a (2*R*,3*S*)‐4‐(methoxyamino)butane‐1,2,3‐triol scaffold to give *N*‐methoxy‐1,3‐Oxazinane (MOANA) and *N*‐methoxy‐1,3‐oxazolidine glycol (MOGNA) nucleotides is strongly dependent on the secondary structure of the surrounding oligonucleotide. The highest yields and strictest adherence to the rules of Watson‐Crick base pairing were observed within the rigid base stack of an A‐type double helix.

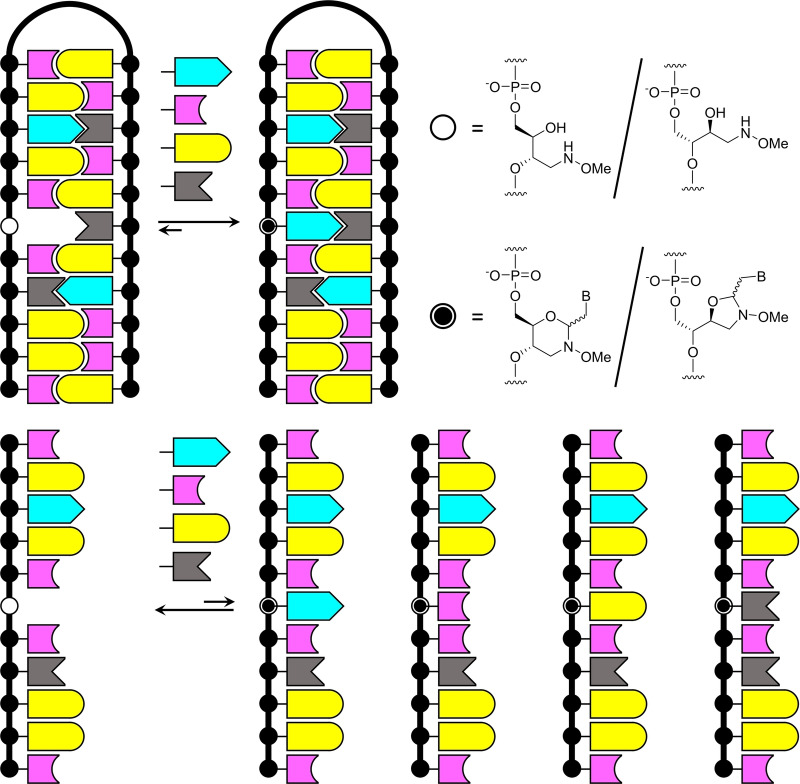

## Introduction

Post‐synthetic introduction of nucleobases or their analogues to an oligonucleotide scaffold, often referred to as “base‐filling”,[^1^, ^2^, ^3^, ^4^, ^5^] is an attractive alternative to conventional stepwise coupling of activated building blocks, especially for preparing a library of oligonucleotides differing only at a single residue. Arguably the most successful application of base‐filling to date is in probes for SNP genotyping[^6^, ^7^, ^8^, ^9^, ^10^] and detection of circulating microRNAs,[^11^, ^12^, ^13^] with reductive amination between nucleobase acetaldehydes and an abasic site within a PNA backbone[^2^, ^14^] as the preferred coupling chemistry. Base‐filling could also provide a useful framework for studying the various factors affecting base pairing, base stacking and, ultimately, secondary and tertiary structure of the nucleic acid itself. For such applications, coupling chemistry more compatible with the sugar phosphate backbone of natural DNA and RNA would be desirable.

**Scheme 1:**
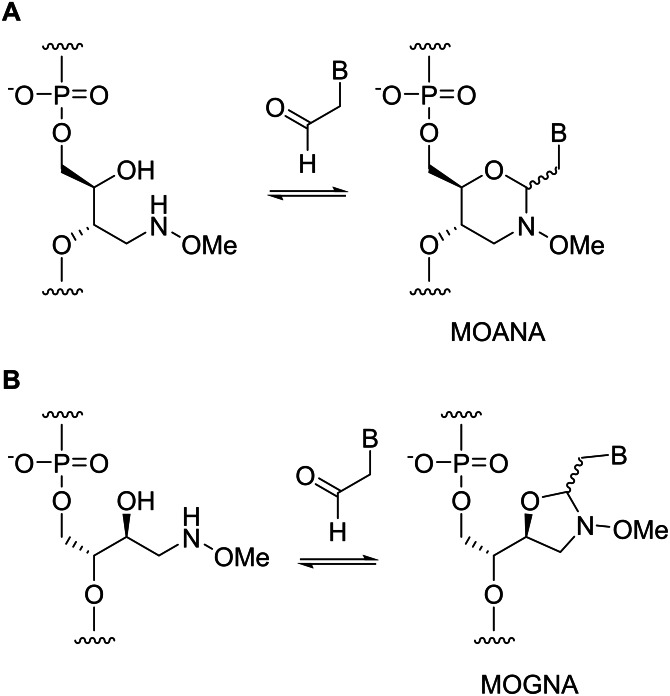
Base‐filling of isomeric (2*R*,3*S*)‐4‐(methoxyamino)butane‐1,2,3‐triol residues, affording A) *N*‐methoxy‐1,3‐oxazinane (MOANA) and B) *N*‐methoxy‐1,3‐oxazolidine glycol (MOGNA) nucleotide analogues.

We have recently become interested in harnessing base‐filling as a screening technique for high‐affinity nucleobase surrogates that could stabilize double helices[^15^, ^16^, ^17^, ^18^, ^19^, ^20^, ^21^, ^22^, ^23^, ^24^, ^25^, ^26^, ^27^, ^28^, ^29^, ^30^, ^31^] without compromising sequence selectivity. Such surrogates include, but are not limited to, organometallic complexes that are notoriously challenging to introduce to oligonucleotides by the established methods.[^32^, ^33^, ^34^] For this application, the base‐filling reaction should ideally be reversible under conditions where the rules of Watson‐Crick base pairing apply, giving rise to a dynamic combinatorial library (DCL).[^35^, ^36^] The first step of reductive amination, namely imine formation, meets this requirement but the reduction step needed to “freeze” the product mixture changes the hybridization of the nitrogen and oxygen atoms involved and thus the geometry of the product. For avoiding this complication, we have previously reported on base‐filling through reaction of an aldehyde and (2*R*,3*S*)‐4‐(methoxyamino)butane‐1,2,3‐triol, affording an *N*‐methoxy‐1,3‐oxazinane nucleic acid (MOANA) residue (Scheme 1A).^[37]^ This reaction is readily reversible at pH 5.5, while the “freezing” is achieved by simply adjusting the pH to neutral, rather than adding a reducing agent.

Our first studies on base filling of a (2*R*,3*S*)‐4‐(methoxyamino)butane‐1,2,3‐triol residue within a double helix employed nucleobase analogues having the reactive formyl group bonded directly to an aromatic carbon atom.^[38]^ This design results in a MOANA nucleotide that is a close structural mimic of its natural counterparts. However, the base‐filling yields were relatively low, presumably owing to the loss of conjugation in the aromatic aldehyde. In the present study, we have investigated whether the formylmethyl derivatives of natural nucleobases, employed by several other groups, would be incorporated more efficiently. Furthermore, 2'‐*O*‐methyl‐RNA versions of the hairpins were also used to assess the impact of the more rigid A‐type double helix on the selectivity of base‐filling. The potential of Hoogsteen base pairing to drive base‐filling was tested on a series of triple helices. Finally, we have explored an alternative attachment (2*R*,3*S*)‐4‐(methoxyamino)butane‐1,2,3‐triol to the oligonucleotide chain, allowing formation of the isomeric *N*‐methoxy‐1,3‐oxazolidine glycol nucleic acid (MOGNA, Scheme 1B) residue on base‐filling.

## Results and Discussion

### Phosphoramidite Building Block Synthesis

**Scheme 2:**
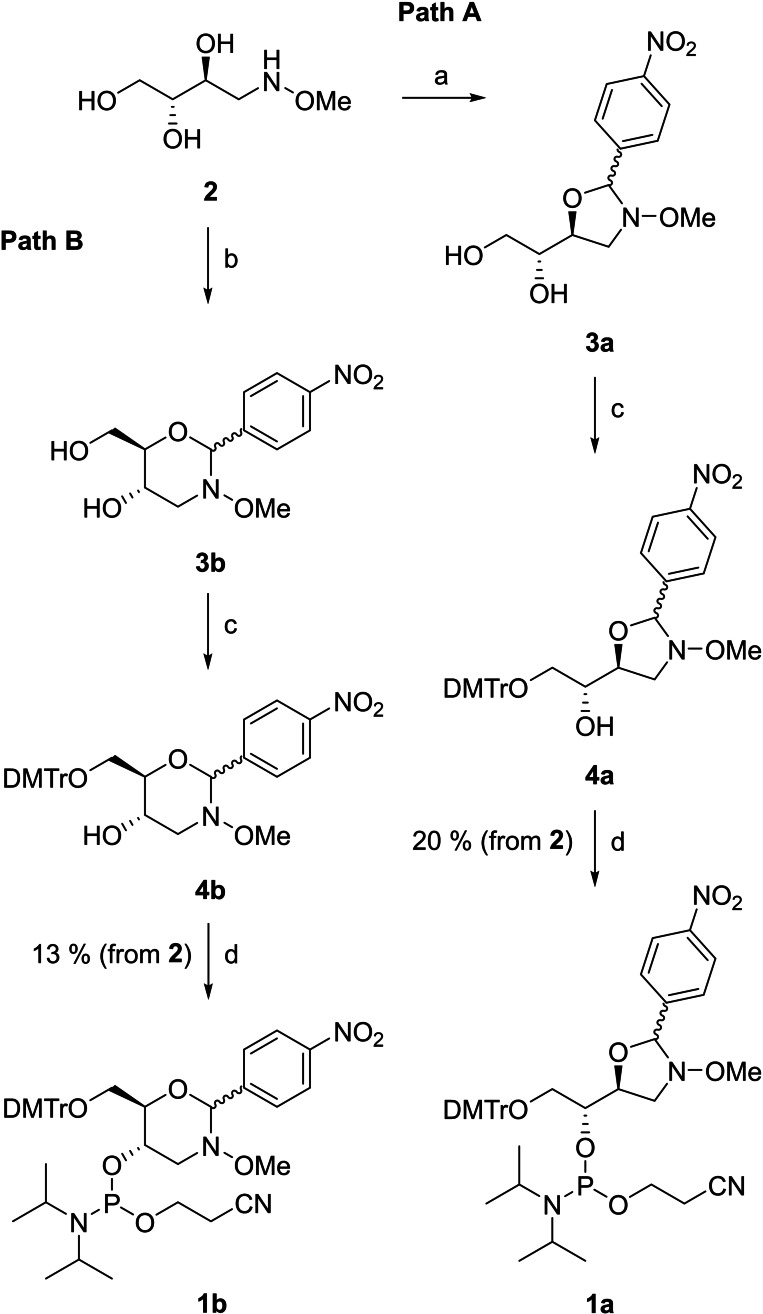
Synthesis of the phosphoramidite building blocks **1 a** and **1 b**. Reagents and conditions: a) 4‐nitrobenzaldehyde, AcOH, 1,4‐dioxane, 50 °C, 16 h; b) 4‐nitrobenzaldehyde, AcOH, 1,4‐dioxane, 50 °C, 3 h; c) DMTrCl, CH_2_Cl_2_, pyridine, N_2_ atmosphere, 25 °C, 15 h; d) 2‐cyanoethyl‐*N*,*N*‐diisopropylchlorophosphoramidite, Et_3_N, CH_2_Cl_2_, N_2_ atmosphere, 25 °C, 1 h.

**Figure 1:**
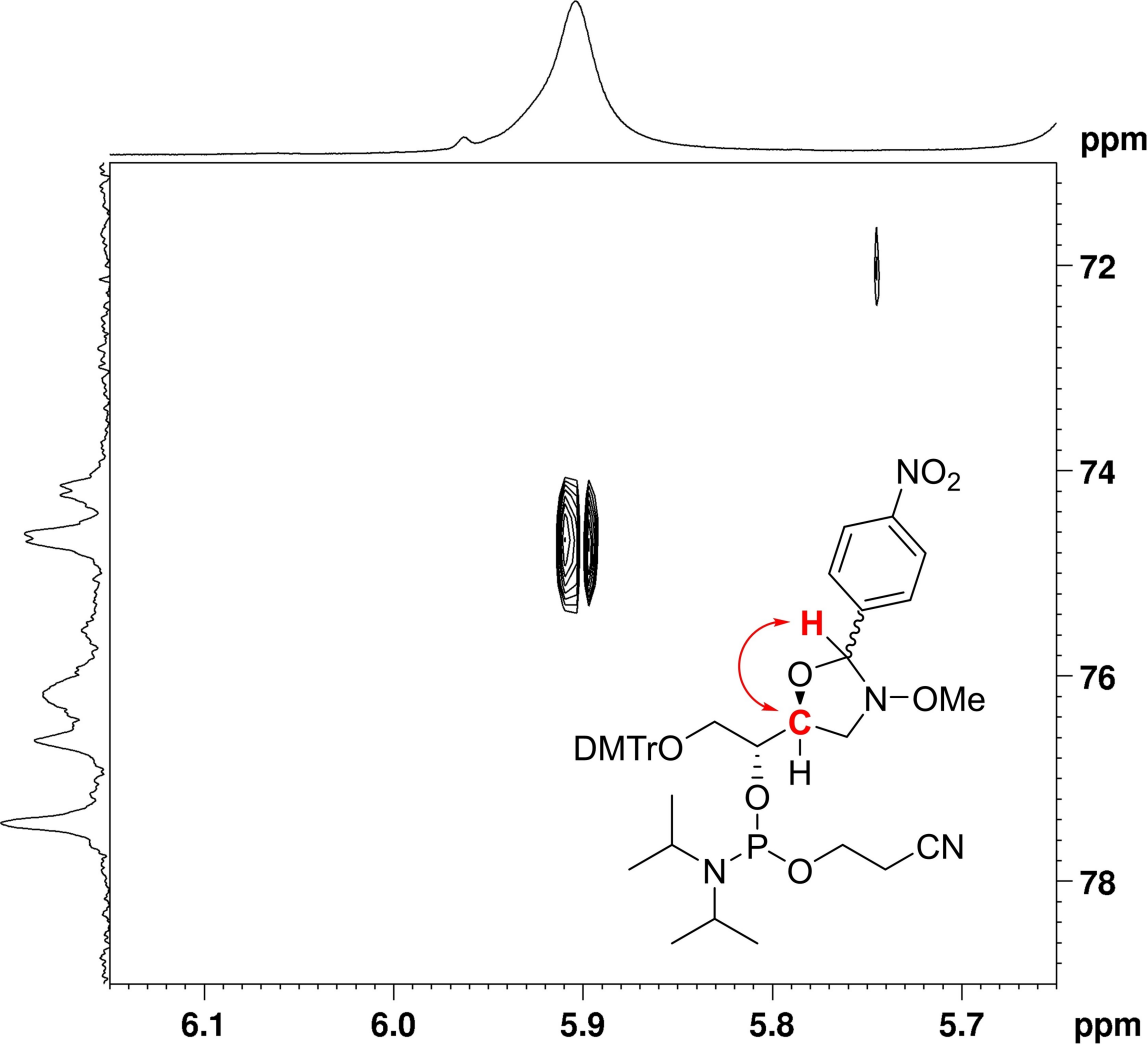
HMBC cross‐peak between the pseudoanomeric proton and C3 of the (2*R*,3*S*)‐4‐(methoxyamino)butane‐1,2,3‐triol chain of the faster‐eluting diastereomer of compound **1 a**, confirming the desired five‐membered ring structure.

Scheme 2 outlines the synthesis of the (2*R*,3*S*)‐4‐(methoxyamino)butane‐1,2,3‐triol phosphoramidite building blocks **1 a** and **1 b**, designed for introduction of either *N*‐methoxy‐1,3‐oxazolidine glycol nucleic acid (MOGNA) or *N*‐methoxy‐1,3‐oxazinane nucleic acid (MOANA) residues into oligonucleotides. The procedure was modified from the one previously reported^[39]^ for a MOANA building block by changing the 4‐(benzoyloxy)benzylidene protection to a 4‐nitrobenzylidene protection, the premise being that the more electron‐deficient aldehyde would react slower and thus allow trapping of the kinetic five‐membered ring product. Accordingly, (2*R*,3*S*)‐4‐(methoxyamino)butane‐1,2,3‐triol (**2**) was first allowed to react with 4‐nitrobenzaldehyde under acidic conditions to afford oxazolidine **3 a**. A longer reaction time led to predominant formation of the thermodynamic six‐membered ring product **3 b**. The primary hydroxy function was then dimethoxytritylated and the secondary one phosphitylated by conventional methods, “locking” the oxazolidine–oxazinane equilibrium and thus allowing chromatographic separation of these isomers. HMBC spectra of the *R*_P_ and *S*_P_ diastereomers of the final product of path A revealed a coupling between the pseudoanomeric proton and C3 of the (2*R*,3*S*)‐4‐(methoxyamino)butane‐1,2,3‐triol chain, establishing the desired oxazolidine structure **1 a**. A relevant expansion of the HMBC spectrum of the faster‐eluting diastereomer is presented in Figure 1 and the complete spectra of both diastereomers in the Supporting Information as Figures S4 and S8. The final product of path B, in turn, was assigned as the oxazinane **1 b** based on the similarity of its spectra to those of the previously reported analogue^[39]^ differing only in the para substituent of the benzylidene protecting group.

### Aldehyde Synthesis

**Scheme 3:**
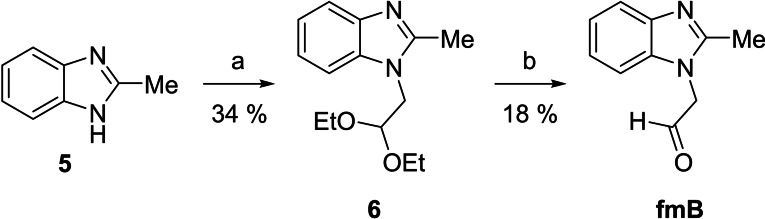
Synthesis of 1‐(formylmethyl)‐2‐methylbenzimidazole (**fmB**). Reagents and conditions: a) bromoacetaldehyde diethyl acetal, Cs_2_CO_3_, DMF, 90 °C, 90 min; b) HCl, H_2_O, 90 °C, 60 min.

Synthesis of formylmethyl derivatives of the canonical nucleobases (**fmA**, **fmC**, **fmG** and **fmT**) has been described in the literature.^[40]^ For a control compound featuring a similar stacking surface as purine nucleobases but lacking the hydrogen bond acceptors and donors, 2‐(2‐methylbenzimidazol‐1‐yl)acetaldehyde (**fmB**) was prepared by a similar procedure (Scheme 3). Accordingly, 2‐methylbenzimidazole (**5**) was first alkylated by treatment with bromoacetaldehyde diethyl acetal to afford intermediate **6**. Removal of the acetal protection by acid‐catalyzed hydrolysis then gave the desired aldehyde **fmB**.

### Oligonucleotide Synthesis

Sequences of the modified oligonucleotide scaffolds used in the present study are summarized in Table 1. The DNA oligonucleotides **ON11a**, **ON11c**, **ON11g**, **ON11t**, **ON12a**, **ON12c**, **ON12g** and **ON12t** were commercial products and syntheses of the DNA‐MOANA oligonucleotides **ON1**, **ON2a**, **ON2c**, **ON2g**, **ON2t** and **ON2s** have been reported previously.[^38^, ^39^] The other modified oligonucleotides were assembled on an automated DNA/RNA synthesizer by the conventional phosphoramidite strategy, using building blocks **1 a** and **1 b** for introduction of the single MOGNA or MOANA residue, respectively. With oligonucleotides **ON3**, **ON4a**, **ON4c**, **ON4g**, **ON4t** and **ON4s** and **ON5**, **ON6a**, **ON6c**, **ON6g**, **ON6u**, **ON6s**, **ON7**, **ON8a**, **ON8c**, **ON8g**, **ON8u** and **ON8s**, the rest of the sequence was made up of standard DNA and 2'‐*O*‐methyl‐RNA building blocks, respectively. However, due to temporary unavailability of the 2'‐*O*‐methyl‐RNA solid support, oligonucleotides **ON6s**, **ON7**, **ON8a**, **ON8c**, **ON8g**, **ON8u** and **ON8s** were synthesized with the 2'‐OH group of the 3'‐terminal cytidine residue free. Coupling yields for the modified phosphoramidite building blocks **1 a** and **1 b** ranged from 77 to 99 % depending on the batch used. The other couplings proceeded with typical, near‐quantitative, efficiency.

**Table 1:** Sequences of the oligonucleotides used in this study. The variable residues (base‐filling scaffolds and nucleotides opposite to them) are underlined.

Oligonucleotide	Sequence^[a]^	
**ON1**	5'‐CGA GCX CTG GC‐3'	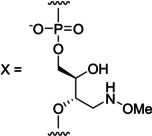
**ON2a**	5'‐GCC AGA GCT CGT TTT CGA GCX CTG GC‐3'	
**ON2c**	5'‐GCC AGC GCT CGT TTT CGA GCX CTG GC‐3'	
**ON2g**	5'‐GCC AGG GCT CGT TTT CGA GCX CTG GC‐3'	
**ON2t**	5'‐GCC AGT GCT CGT TTT CGA GCX CTG GC‐3'	
**ON2s**	5'‐GCC AGS GCT CGT TTT CGA GCX CTG GC‐3'	
**ON3**	5'‐CGA GCY CTG GC‐3'	
**ON4a**	5'‐GCC AGA GCT CGT TTT CGA GCY CTG GC‐3'	
**ON4c**	5'‐GCC AGC GCT CGT TTT CGA GCY CTG GC‐3'	
**ON4g**	5'‐GCC AGG GCT CGT TTT CGA GCY CTG GC‐3'	
**ON4t**	5'‐GCC AGT GCT CGT TTT CGA GCY CTG GC‐3'	
**ON4s**	5'‐GCC AGS GCT CGT TTT CGA GCY CTG GC‐3'	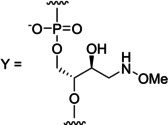
**ON5**	5'‐cga gcX cug gc‐3'	
**ON6a**	5'‐gcc aga gcu cgu uuu cga gcX cug gc‐3'	
**ON6c**	5'‐gcc agc gcu cgu uuu cga gcX cug gc‐3'	
**ON6g**	5'‐gcc agg gcu cgu uuu cga gcX cug gc‐3'	
**ON6u**	5'‐gcc agu gcu cgu uuu cga gcX cug gc‐3'	
**ON6s** ^[b]^	5'‐gcc agS gcu cgu uuu cga gcX cug gc‐3'	
**ON7** ^[b]^	5'‐cga gcY cug gc‐3'	
**ON8a** ^[b]^	5'‐gcc aga gcu cgu uuu cga gcY cug gc‐3'	
**ON8c** ^[b]^	5'‐gcc agc gcu cgu uuu cga gcY cug gc‐3'	
**ON8g** ^[b]^	5'‐gcc agg gcu cgu uuu cga gcY cug gc‐3'	
**ON8u** ^[b]^	5'‐gcc agu gcu cgu uuu cga gcY cug gc‐3'	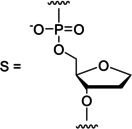
**ON8s** ^[b]^	5'‐gcc agS gcu cgu uuu cga gcY cug gc‐3'	
**ON9**	5'‐TTT TTT TTT TTT TTT XT‐3'	
**ON10**	5'‐TTT TTT TTT TTT TTT YT‐3'	
**ON11a**	5'‐GAT TTT TTT TTT TTT TTG C‐3'	
**ON11c**	5'‐GCT TTT TTT TTT TTT TTG C‐3'	
**ON11g**	5'‐GGT TTT TTT TTT TTT TTG C‐3'	
**ON11t**	5'‐GTT TTT TTT TTT TTT TTG C‐3'	
**ON12a**	5'‐GCA AAA AAA AAA AAA AAA C‐3'	
**ON12c**	5'‐GCA AAA AAA AAA AAA AAC C‐3'	
**ON12g**	5'‐GCA AAA AAA AAA AAA AAG C‐3'	
**ON12t**	5'‐GCA AAA AAA AAA AAA AAT C‐3'	

[a] Uppercase letters refer to DNA and lowercase letters to 2'‐O‐methyl‐RNA nucleotides, “X” to the MOANA residue, “Y” to the MOGNA residue and “S” to the abasic 2‐(hydroxymethyl)tetrahydrofuran‐3‐ol spacer. [b] The 2'‐OH group of the 3'‐terminal nucleoside of this sequence was unmethylated.

After completion of chain assembly, the oligonucleotides were released from the solid supports and their phosphate and base protections removed by conventional ammonolysis. In contrast to the 4‐(benzoyloxy)benzylidene protection used for the MOANA building block in our previous studies,[^38^, ^39^] the 4‐nitrobenzylidene protections of both the MOANA and MOGNA residues were still largely intact after this treatment so an additional acidic hydrolysis step had to be included. On the other hand, the 4‐nitrobenzylidene protecting group facilitated chromatographic purification in cases where coupling of the modified building block had been less than satisfactory. Accordingly, the crude products were first fractioned by RP‐HPLC with this protection on, the fractions containing the desired material incubated in aqueous acetic acid and finally purified again by RP‐HPLC. The pure oligonucleotide products were characterized by UPLC‐ESI‐MS and quantified UV spectrophotomerically. As reported previously,[^37^, ^38^, ^39^] the modified oligonucleotides were prone to react with formaldehyde and acetaldehyde contaminating the solvents so the mass spectra typically showed signals for the corresponding products in dynamic equilibrium with the naked oligonucleotide. In some cases, traces of the 4‐nitrobenzylidene protection could also be detected but this contamination was deemed acceptable as it would anyway get displaced during the dynamic combinatorial chemistry (DCC) experiments.

#### Duplex and Triplex Stability of the Oligonucleotides Under Acidic Conditions

UV melting profiles of the hairpin oligonucleotides as well as the triplex assemblies (Figures S57–S79 in the Supporting Information) were measured under the slightly acidic conditions (pH=5.5) of the DCC experiments to establish a suitable temperature for those experiments. For the DNA‐MOANA hairpin oligonucleotides **ON2a**, **ON2c**, **ON2g**, **ON2t** and **ON2s**, the melting profiles have been reported previously, with melting temperatures ranging from 54 to 57 °C.^[38]^ Curiously, melting temperatures of the DNA–MOGNA hairpins **ON4a**, **ON4c**, **ON4g**, **ON4t** and **ON4s** were considerably higher (62–68 °C), despite the minimal structural difference. The 2'‐OMe‐RNA–MOANA and 2'‐OMe‐RNA–MOGNA hairpins **ON6a**, **ON6c**, **ON6g**, **ON6u** and **ON6s** and **ON8a**, **ON8c**, **ON8g**, **ON8u** and **ON8s** were even higher‐melting (*T*_m_=74–82 °C), consistent with the duplex‐stabilizing effect of the 2'‐*O*‐Me‐RNA backbone. With all duplexes, denaturation at ambient temperature (23 °C) was negligible so this temperature was chosen for the DCC experiments.

The triplexes ON11t ⋅ ON12a*ON9, ON11g ⋅ ON12c*ON9, ON11c ⋅ ON12g*ON9 and ON11a ⋅ ON12t*ON9 and ON11t ⋅ ON12a*ON10, ON11g ⋅ ON12c*ON10, ON11c ⋅ ON12g*ON10 and ON11a ⋅ ON12t*ON10 exhibited typical biphasic melting profiles. The higher melting temperature, assigned to unwinding of the Watson–Crick double helix, was remarkably similar with all triplexes, approximately 42 °C. The lower melting temperature, corresponding to dissociation of the triplex‐forming oligonucleotide **ON9** or **ON10** from the double helix, showed much greater variation, ranging from 21 to 31 °C. In other words, with some triplexes significant Hoogsteen melting would take place already at ambient temperature (23 °C). Nevertheless, this temperature was still chosen for the DCC experiments as decreasing the temperature to the point where Hoogsteen melting would be negligible would have made equilibration of the DCL impractically slow.

#### Dynamic Combinatorial Chemistry

**Figure 2:**
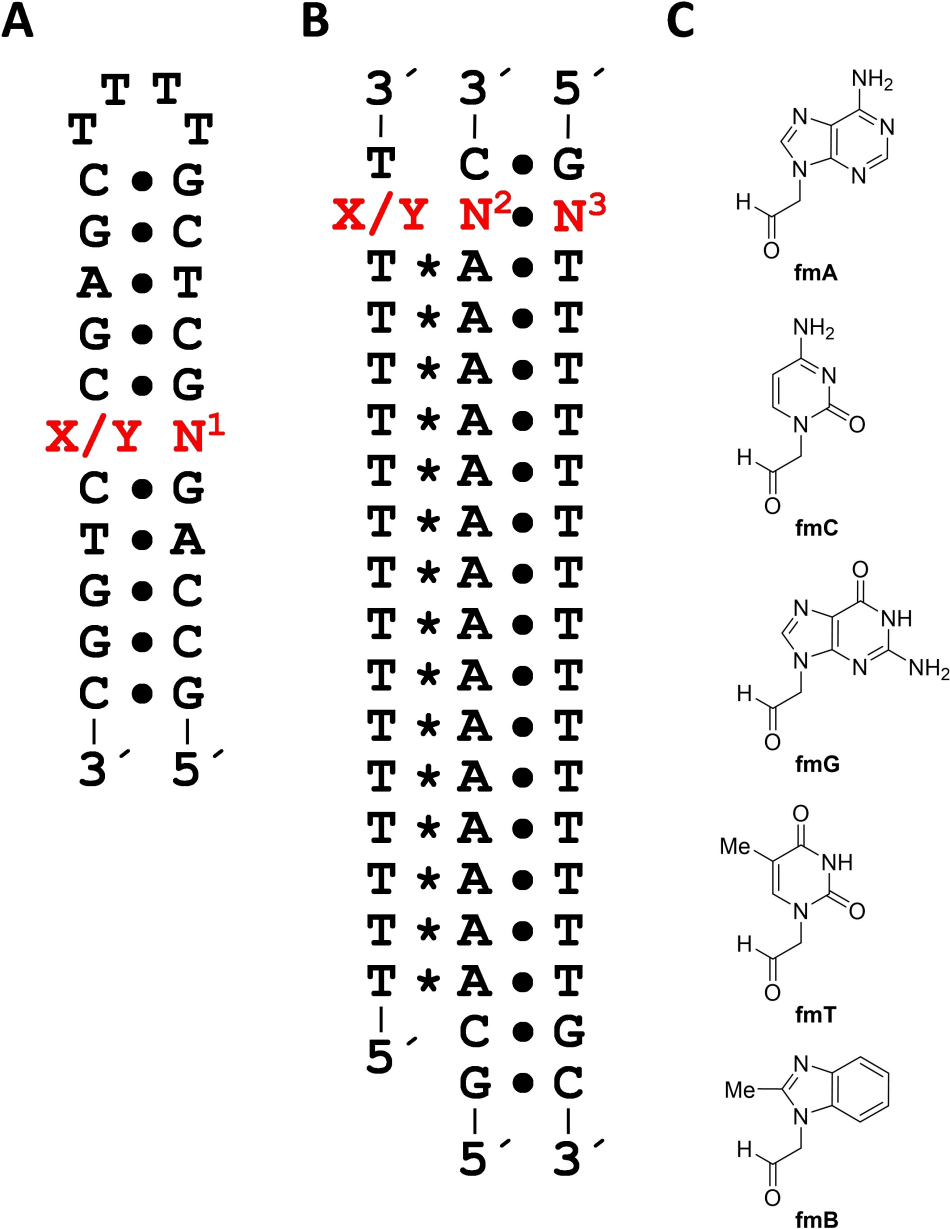
Structures of the A) hairpin and B) triple‐helical oligonucleotides and C) aldehydes used in the dynamic combinatorial chemistry experiments. “X” refers to the MOANA residue and “Y” to the MOGNA residue. N^1^, N^2^ and N^3^ can be any of the canonical nucleosides and N^1^ also the abasic 2‐(hydroxymethyl)tetrahydrofuran‐3‐ol spacer.

Efficiency and selectivity of base‐filling a MOANA or MOGNA residue inside a double helix was studied by incubating each of the hairpin oligonucleotides (Figure 2A) in a mixture of the formylmethyl derivatives of canonical nucleobases (**fmA**, **fmC**, **fmG** and **fmT**) and 2‐methylbenzimidazole (**fmB**) at pH=5.5, *I*=0.10 M and T=23 °C. The same conditions were used for the triple‐helical models incorporating the MOANA or MOGNA residue in the Hoogsteen strand (Figure 2B), allowing analogous investigation of base‐filling within the major groove. Concentration of each of the oligonucleotides was 1.0 μM and concentration of each of the aldehydes 2.0 μM. For single‐stranded references, the 11‐mer oligonucleotides **ON1**, **ON3**, **ON5** and **ON7**, comprising only the 3'‐end of the hairpins, or the Hoogsteen strands **ON9** and **ON10** alone were studied under otherwise the same conditions except that a 10‐fold concentration (20 μM) of the aldehydes was used.

Kinetics of base‐filling were first established by determining the compositions of the reaction mixtures described above by UPLC‐TOF‐MS at appropriate time intervals. Hairpin oligonucleotides **ON2t**, **ON4t**, **ON6u** and **ON8u** were chosen for this study as they were expected to yield strong signals owing to efficient and selective base‐filling by **fmA**. Indeed, first‐order disappearance of the starting material was observed in each case, accompanied by appearance of the **fmA** adduct (time profiles presented as Figures S82–S85 in the Supporting Information). The half‐lives ranged from 9 to 32 h, with no clear dependence on the backbone structure. 120 h, translating to 4–13 half‐lives, was deemed a sufficiently long reaction time for the DCC reactions.

**Figure 3:**
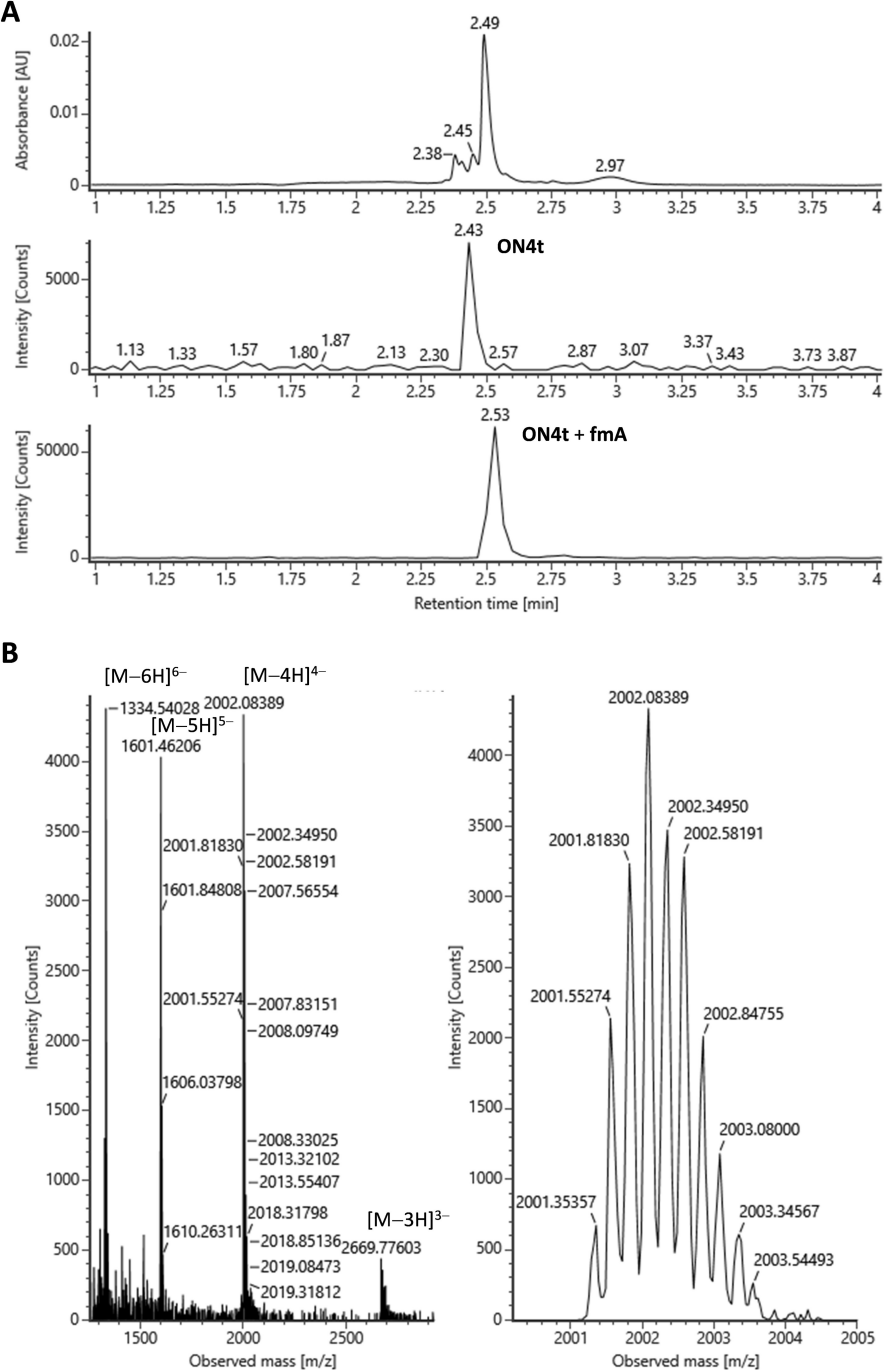
A) UV and extracted ion UPLC traces of the DCC product mixture and B) mass spectrum of the main component on incubation of the hairpin oligonucleotide **ON4t** (1.0 μM) with a mixture of aldehydes **fmA**, **fmC**, **fmG**, **fmT** and **fmB** (2.0 μM) at *T*=23 °C, pH=5.5 (20 mM cacodylate buffer) and *I*(NaClO_4_)=0.10 M for 120 h; ACQUITY Premier OST column (50×2.1 mm, 1.7 μm); flow rate=0.4 mL min^−1^; linear gradient (5–25 % over 4 min) of MeOH in an aqueous solution of hexafluoroisopropanol (40 mM) and triethylamine (7 mM); *λ*=254 nm; *T*=60 °C.

After incubating for at least 120 h, compositions of the DCC reaction mixtures were determined by UPLC‐ESI‐MS. Because of the relatively slow kinetics, “freezing” of the samples by adjusting pH was deemed unnecessary and the samples were instead injected directly from the reaction mixtures. The data obtained for the hairpin oligonucleotide **ON4t** is shown in Figure 3 as an illustrative example and all UV and extracted ion chromatograms in Figures S86–S119 in the Supporting Information. As expected, conversion of the single‐stranded control oligonucleotides to the aldehyde adducts was low (10–40 %) despite the relatively high (20 μM) aldehyde concentration. The thymine derivative **fmT** was in most cases incorporated less efficiently than the other nucleobase derivatives and the 2‐methylbenzimidazole derivative **fmB** not at all. Otherwise, no base‐selectivity was observed in the absence of a templating strand, regardless of the sequence or backbone chemistry of the single‐stranded oligonucleotide.

**Figure 4:**
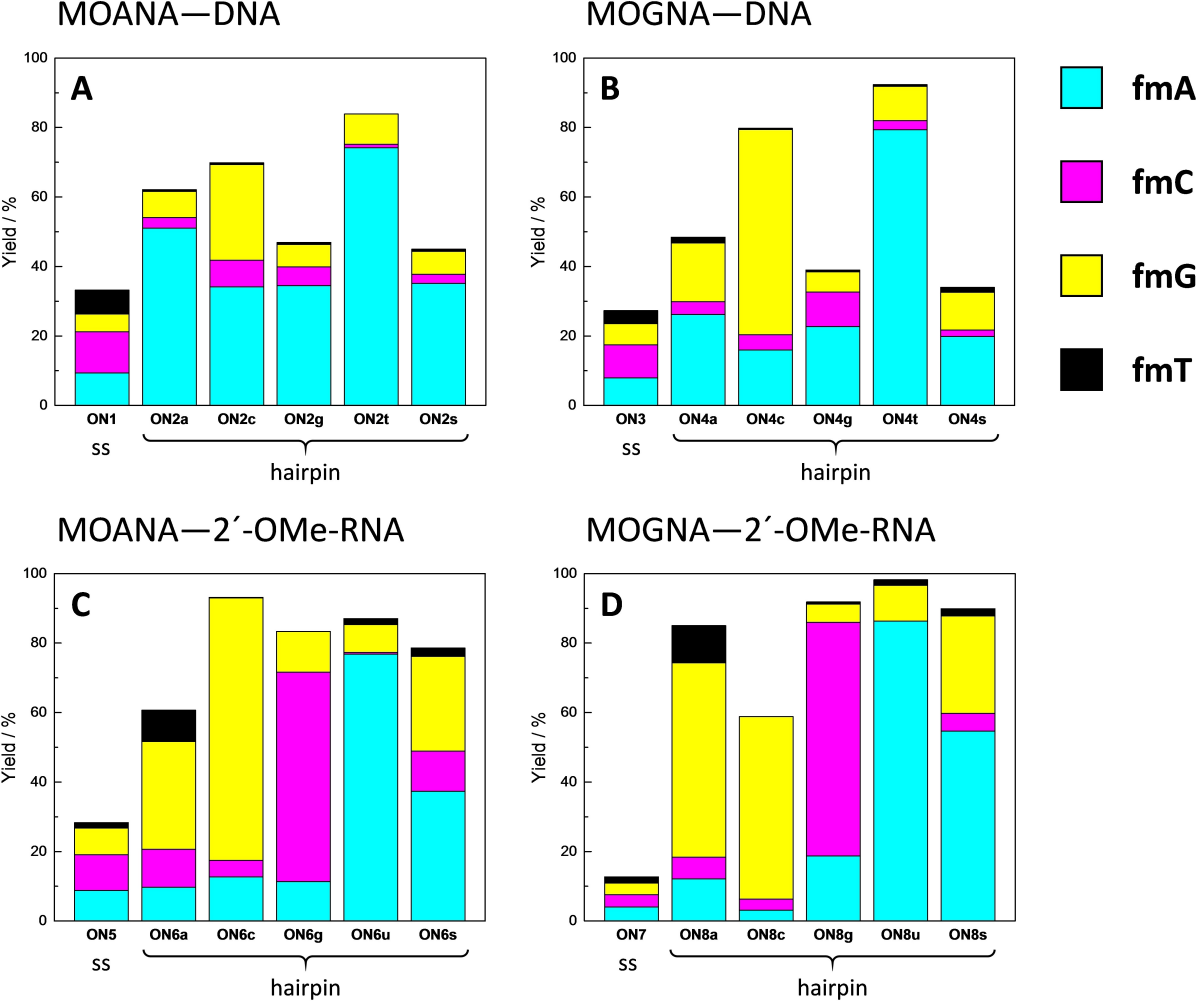
Yields of base‐filling A) MOANA‐DNA, B) MOGNA‐DNA, C) MOANA‐2'‐OMe‐RNA and D) MOANA‐2'‐OMe‐RNA oligonucleotides with a mixture of formylmethylated derivatives of adenine (**fmA**, cyan), cytosine (**fmC**, magenta), guanine (**fmG**, yellow) and thymine (**fmT**, black); [oligonucleotides]=1.0 μM; [aldehydes]=20 μM (with the single‐stranded oligonucleotides **ON1**, **ON3**, **ON5** and **ON7**)/2.0 μM (with the other oligonucleotides); *T*=23 °C; pH=5.50 (20 mM cacodylate buffer); *I*(NaClO_4_)=0.10 M; reaction time=120 h. In addition to the derivatives of the canonical nucleobases, 2‐(2‐methylbenzimidazol‐1‐yl)acetaldehyde (**fmB**) was also included in the reaction mixtures but the corresponding base‐filling products could not be detected.

With the MOANA‐DNA hairpin (Figure 4A) **ON2t**, placing thymine opposite to the modified residue, the adenine derivative **fmA** was incorporated with 74 % yield and 88 % selectivity, consistent with Watson‐Crick base pairing. Corresponding results on the other MOANA‐DNA hairpins were much less impressive, **ON2a**, **ON2c** and **ON2g** all favoring incorporation of the mismatched **fmA**. **ON2s**, placing an abasic site opposite to the modified residue, was also converted mainly to the **fmA** adduct, with 35 % yield and 78 % selectivity.

Results on the MOGNA‐DNA hairpins (Figure 4B) largely paralleled those on the MOANA‐DNA hairpins except that now also **ON4c** appeared to follow the rules of Watson‐Crick base pairing, **fmG** being incorporated with a reasonable 59 % yield and 74 % selectivity. With **ON4t**, **fmA** was incorporated with a 79 % yield and 86 % selectivity. Base‐filling against purines was somewhat less efficient than with the corresponding MOANA‐DNA hairpins (40–50 % overall conversion), with no particular product clearly standing out in either case. The abasic hairpin **ON4s** reacted similarly to its MOANA counterpart **ON2s**, with low yields (19 and 10 %) of the **fmA** and **fmG** adducts and traces (1.9 and 1.4 %) of the **fmC** and **fmT** adducts.

The MOANA‐2'‐OMe‐RNA hairpins (Figure 4C) showed markedly improved base‐filling yields with either of the purine bases opposite to the modified residue. With **ON6a**, the main product was still mismatched (**fmG** at 31 % yield) but, in contrast to the DNA hairpins, a significant fraction (9 %) of the expected **fmT** adduct was also observed. With **ON6g**, in turn, the Watson‐Crick partner **fmC** was favored, although both the yield (60 %) and selectivity (72 %) still left something to desire. Base‐filling opposite to pyrimidine residues again gave the highest yields and selectivities, 75 and 81 % for incorporation of **fmG** into **ON6c** and 78 and 88 % for incorporation of **fmA** into **ON6u**. Interestingly, the abasic hairpin **ON6s** reacted much more readily than either of its DNA counterparts, the **fmA** (37 %) and **fmG** (27 %) adducts dominating in the product mixture.

Changing the base‐filling scaffold from MOANA to MOGNA while retaining the 2'‐OMe‐RNA backbone elsewhere (Figure 4D) had the largest impact on the hairpin placing guanine opposite to the modified residue (**ON8g**). Incorporation of **fmC** into this hairpin proceeded with yield and selectivity comparable to those discussed above for the matched purine nucleobase derivatives (67 and 73 %). Incorporation of **fmG** into **ON8c**, on the other hand, was retarded (52 % yield) although the selectivity was still good (89 %). **ON8a**, **ON8u** and **ON8s** afforded essentially the same product mixtures as their MOANA counterparts but with somewhat higher overall conversions.

**Figure 5:**
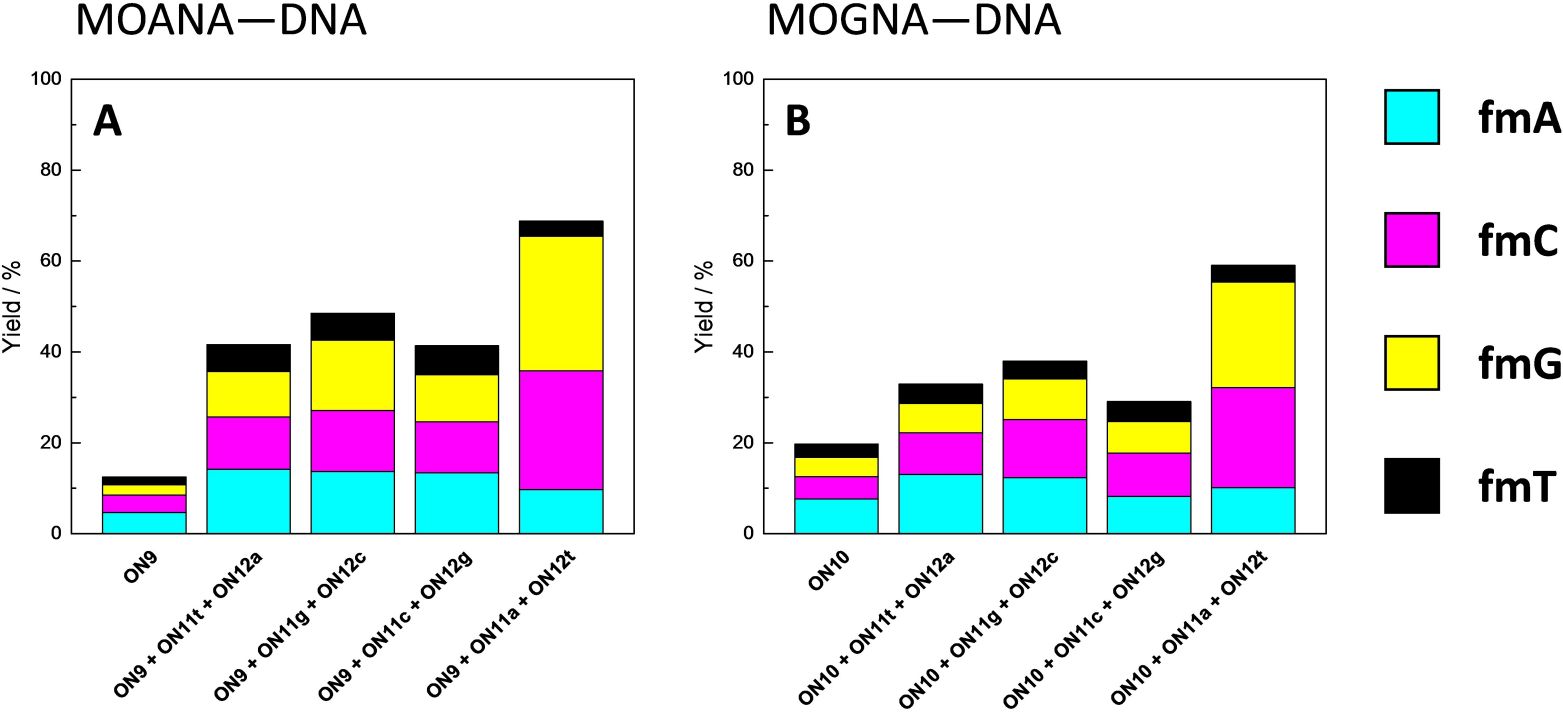
Yields of base‐filling A) MOANA‐DNA and B) MOGNA‐DNA oligonucleotides with a mixture of formylmethylated derivatives of adenine (**fmA**, cyan), cytosine (**fmC**, magenta), guanine (**fmG**, yellow) and thymine (**fmT**, black); [oligonucleotides]=1.0 μM; [aldehydes]=20 μM (with the single‐stranded oligonucleotides **ON9**, and **ON10** alone)/2.0 μM (with the triplex assemblies); *T*=23 °C; pH=5.50 (20 mM cacodylate buffer); *I*(NaClO_4_)=0.10 M; reaction time=120 h. In addition to the derivatives of the canonical nucleobases, 2‐(2‐methylbenzimidazol‐1‐yl)acetaldehyde (**fmB**) was also included in the reaction mixtures but the corresponding base‐filling products could not be detected.

In contrast to the hairpin oligonucleotides, base‐filling of the Hoogsteen strand of the triplex assemblies was largely insensitive to the base pair within the Watson‐Crick duplex and opposite to the MOANA or MOGNA residue (Figure 5). The overall conversions were typically lower than 50 % and no selectivity to any particular aldehyde was observed. A notable exception were the ON11a ⋅ ON12t*ON9 and ON11a ⋅ ON12t*ON10 triplexes, which moderately favored base‐filling with **fmC** (26 and 22 %) and **fmG** (29 and 23 %). The impact of the base‐filling scaffold (MOANA or MOGNA) was minor but slightly higher conversions were observed with the former.

The preference for base‐filling double helices with purine derivatives is in line with previous reports on other backbones and coupling chemistries[^2^, ^14^, ^41^] and probably attributable to base stacking as the main driving force. However, the observed selectivity of the adenine, cytosine and guanine derivatives for hairpins placing their canonical Watson‐Crick partners opposite to the MOANA or MOGNA residue suggests that base pairing also plays an important role. This interpretation is further corroborated by the strong discrimination against 2‐(2‐methylbenzimidazol‐1‐yl)acetaldehyde (**fmB**), featuring a purine‐sized stacking surface but no hydrogen bond donors or acceptors on its “Watson‐Crick” face. The relatively inefficient base‐filling by thymine derivatives is also not unprecedented although in the present case the difference between **fmT** and **fmA**, **fmC** or **fmG** was particularly striking. Similar strong discrimination against thymine (or uracil) has been reported previously in nonenzymatic primer‐extension reactions with activated nucleotides^[42]^ and non‐covalent binding of nucleosides to a gap within double‐helical DNA.^[43]^ With only two hydrogen bonds and a relatively small stacking surface, thymine is expected to bind weaker than the other nucleobases but the drastic difference to adenine and cytosine still seems to require an additional explanation. It is interesting to note that while **fmT** gave the lowest base‐filling yield in all of the systems studied, discrimination against it was strongest in the hairpins and weakest in the single‐stranded oligonucleotides. Perhaps the unnatural geometry of the MOANA and MOGNA nucleotides leads to steric clash between the C5‐methyl substituent and the double‐helical environment.

In most cases, the yield and selectivity of base‐filling a MOANA or MOGNA scaffold were higher with the 2'‐O‐methyl‐RNA hairpins than with their DNA counterparts. This difference was particularly evident with the cytosine derivative **fmC**. Presumably, the more rigid and preorganized A‐type double helix of the 2'‐O‐methyl‐RNA hairpins leads to a lower entropic penalty of base‐filling than the more flexible B‐type double helix of the DNA hairpins. In this regard, it is interesting to note that PNA ‐ a scaffold particularly well‐suited for base‐filling ‐ is highly preorganized for hybridization with both DNA and RNA, especially when furnished with a chiral backbone.[^44^, ^45^, ^46^, ^47^, ^48^] The PNA‐RNA duplex is actually largely A‐type.^[49]^ The PNA‐DNA duplex also exhibits considerable displacement of the base pairs away from the helical axis, typical of the A‐type conformation, but in other respects resembles a B‐type double helix.[^50^, ^51^]

The impact of the base‐filling scaffold (MOANA or MOGNA) is less clear‐cut as that of the helix geometry and rigidity. In the DNA hairpins the MOGNA scaffold appears to offer some advantage. With the 2'‐O‐methyl‐RNA hairpins the situation is more ambiguous although with the cytosine derivative **fmC** a definite improvement in both yield and selectivity was observed. The 5‐membered oxazolidine ring of the MOGNA scaffold is conformationally more flexible than the 6‐membered oxazinane ring of the MOANA scaffold but the situation is further complicated by both presumably existing in a dynamic equilibrium between α and β pseudoanomers. In any case, the observed selectivity of base‐filling the 2'‐O‐methyl‐RNA hairpins strongly suggests that both scaffolds are able to orient the nucleobase moiety conducively for Watson‐Crick base pairing.

To the best of our knowledge, base‐filling of a triple helix has not been attempted before. The results presented herein are obviously far from satisfying but, given that the triplexes were incubated at a tenfold lower aldehyde concentration than the single Hoogsteen strands, a somewhat higher affinity to the former could nevertheless be observed. The lack of any base‐selectivity in most cases as well as the anomalous relatively efficient base‐filling opposite to a thymine residue in the homopurine strand in triplexes ON11a ⋅ ON12t*ON9 and ON11a ⋅ ON12t*ON10 suggests that this affinity cannot be attributed to Hoogsteen base pairing. Terminal base pairs are usually weaker than internal ones so it seems possible that placing the MOANA or MOGNA residue in the middle of the Hoogsteen strand could lead to higher base‐filling yields and selectivities also in triple‐helical systems.

#### Impact of Base‐Filling on Duplex Stability

According to the DCC experiments, at least the hairpin oligonucleotides with thymine or uracil opposite to the MOANA or MOGNA residue should remain base‐filled with **fmA** under conditions of a typical UV melting experiment, allowing assessment of the impact of such derivatization on duplex stability. To this end, UV melting profiles (Figure S118–S137 in the Supporting Information) of 1.0 μM naked hairpins were first determined at pH=7.4 (20 mM cacodylate buffer) and *I*=0.10 M (adjusted with NaClO_4_). All hairpins showed typical sigmoidal melting curves. Most of the curves were monophasic but with the MOGNA‐DNA hairpins **ON4a**, **ON4c**, **ON4g** and **ON4s**, an additional low‐temperature transition was also observed. Melting temperatures for the main transition (Table 2), assigned as unwinding of the double helix, were somewhat higher than those obtained at pH=5.5 with all hairpins. In the case of the 2‐*O*‐methyl‐RNA hairpins, most melting temperatures exceeded 80 °C and the curves did not level off even at 90 °C. Because of their extreme stability even in the naked form, the 2‐*O*‐methyl‐RNA hairpins were deemed unsuitable for studying the impact of base‐filling. Therefore, these experiments were limited to the DNA hairpins.

**Table 2:** UV melting temperatures of the naked hairpin oligonucleotides and their base‐filling products with aldehyde **fmA**; [oligonucleotides]=1.0 μM; pH=7.4 (20 mM cacodylate buffer); *I*(NaClO_4_)=0.10 M.

Oligonucleotide	Tm (naked)/°C	Tm (+ fmA)/°C	ΔTm/°C
**ON2a**	67.4±0.1	67.9±0.3	+0.5
**ON2c**	64.5±0.1	64.4±0.1	−0.1
**ON2g**	68.5±0.1	66.7±0.2	−1.8
**ON2t**	65.4±0.1	66.5±0.3	+1.1
**ON2s**	65.3±0.2	62.8±0.2	−2.5
**ON4a**	66.7±0.1	60.7±0.1	−6.0
**ON4c**	65.9±0.4	61.0±0.2	−4.9
**ON4g**	68.8±0.2	62.9±0.1	−5.9
**ON4t**	64.7±0.1	64.2±0.1	−0.5
**ON4s**	66.9±0.1	61.7±0.1	−5.2
**ON6a**	83.2±0.1	n.a.^[a]^	n.a.^[a]^
**ON6c**	77.8±0.1	n.a.^[a]^	n.a.^[a]^
**ON6g**	83.0±0.1	n.a.^[a]^	n.a.^[a]^
**ON6u**	79.3±0.1	n.a.^[a]^	n.a.^[a]^
**ON6s**	79.5±0.1	n.a.^[a]^	n.a.^[a]^
**ON8a**	83.4±0.1	n.a.^[a]^	n.a.^[a]^
**ON8c**	80.7±0.1	n.a.^[a]^	n.a.^[a]^
**ON8g**	83.7±0.2	n.a.^[a]^	n.a.^[a]^
**ON8u**	80.2±0.5	n.a.^[a]^	n.a.^[a]^
**ON8s**	79.8±0.1	n.a.^[a]^	n.a.^[a]^

[a] Not determined.

**Figure 6:**
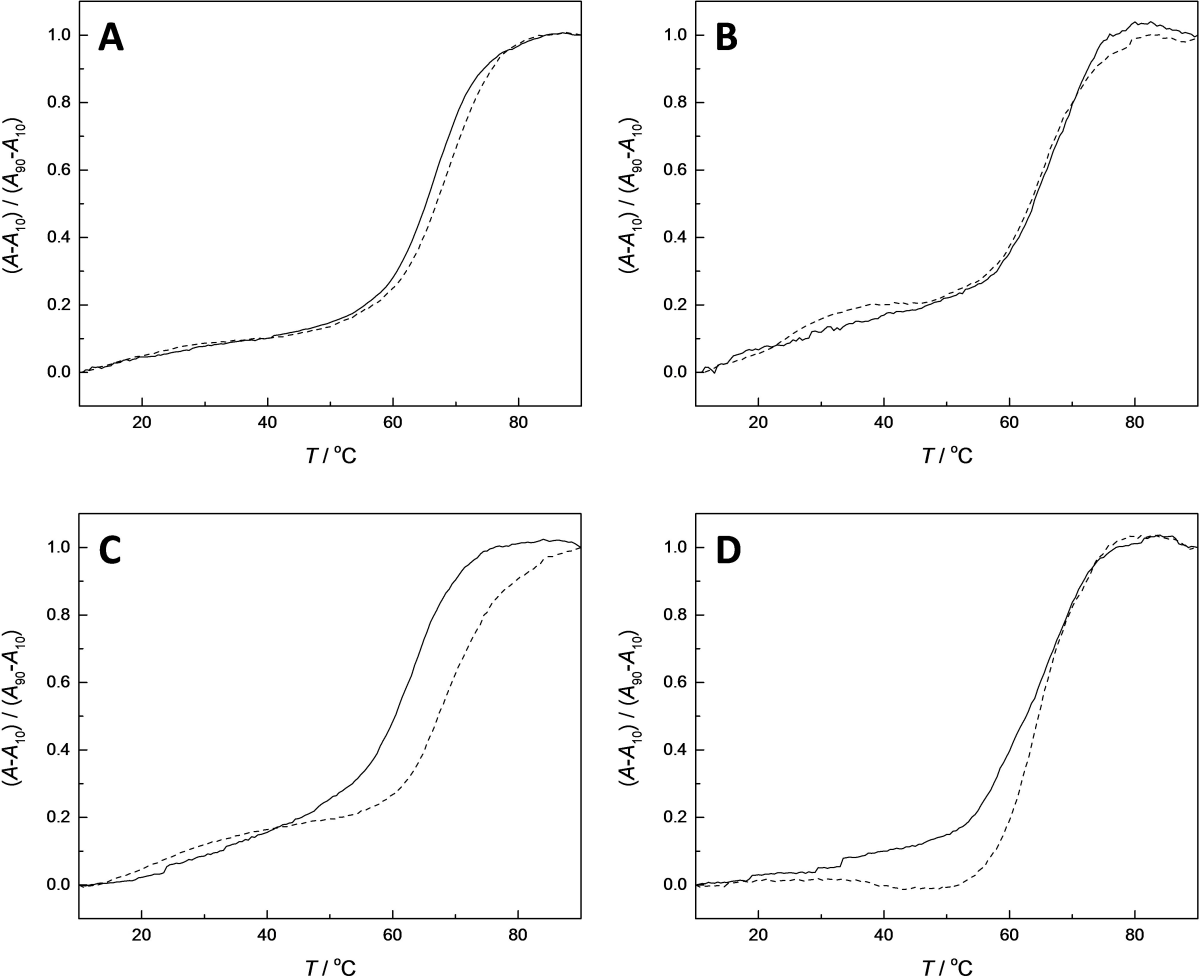
UV melting profiles of hairpins A) **ON2g**, B) **ON2t**, C) **ON4g** and D) **ON4t**, either naked (dashed line) or base‐filled with aldehyde **fmA** (solid line); [oligonucleotides]=1.0 μM; pH=7.4 (20 mM cacodylate buffer); *I*(NaClO_4_)=0.10.

The MOANA‐DNA and MOGNA‐DNA hairpin oligonucleotides were incubated with two equivalents of the adenine derivative **fmA** at 23 °C and pH=5.5 for 120 h, after which the pH of the solutions was adjusted to 7.4. The UV melting profiles of the product mixtures thus obtained were determined under the same conditions as used for the naked hairpin oligonucleotides (representative examples shown in Figure 6). With the MOANA‐DNA hairpins, the impact of base‐filling with **fmA** on the melting temperature was minor and in, most cases, negative. With **ON2a** and **ON2t** modest stabilization was observed, +0.5 and +1.1 °C, respectively. It would be tempting to attribute the latter stabilization to Watson‐Crick base pairing, in line with the observed selectivity in the DCC experiments. However, the differences between the melting temperatures of the various hairpins are too small to allow a firm conclusion. With the MOGNA‐DNA hairpins, base‐filling with **fmA** was universally destabilizing and much more so than with the MOANA‐DNA hairpins, by approximately −5 °C. The sole exception was the Watson‐Crick match **ON4t**, the melting temperature of which decreased by only 0.5 °C. Apparently steric constraints of the MOGNA scaffold are more stringent than those of the MOANA scaffold, resulting in more pronounced disruption of the double helix upon incorporation of a mismatched nucleobase derivative.

## Conclusions

A series of DNA and 2'‐*O*‐methyl‐RNA oligonucleotides incorporating a single (2*R*,3*S*)‐4‐(methoxyamino)butane‐1,2,3‐triol residue were synthesized and their base‐filling with mixtures of aldehyde derivatives of nucleobases studied. Two isomers of this reactive scaffold were tested, affording either an *N*‐methoxy‐1,3‐oxazinane (MOANA) or an *N*‐methoxy‐1,3‐oxazolidine glycol (MOGNA) nucleoside analogue on cyclization with an aldehyde. Base‐filling of (2*R*,3*S*)‐4‐(methoxyamino)butane‐1,2,3‐triol residues was more efficient with aliphatic than aromatic aldehydes, consistent with the loss of conjugation between the carbonyl group and the aromatic π electron system in the latter. The reaction was strongly driven by base stacking and proceeded with a considerably higher yield and base pairing selectivity within the relatively rigid A‐type double helices than within the more flexible B‐type double helices. In the former case, good adherence to the rules of Watson–Crick base pairing was observed with aldehyde derivatives of all canonical nucleobases except thymine. In contrast, the impact of different isomers of the (2*R*,3*S*)‐4‐(methoxyamino)butane‐1,2,3‐triol scaffold was modest and sequence‐dependent.

## Experimental

### General methods

All chemicals, including the unmodified oligonucleotides, were commercial products and used as received. Solvents used in the organic syntheses were dried over activated 4 Å molecular sieves. For preparation of HPLC elution buffers, Et_3_N was freshly distilled. NMR spectra were recorded on Bruker Biospin 500 and 600 MHz NMR spectrometers and the chemical shifts (δ, ppm) referenced to either the residual solvent signal (in the case of ^1^H and ^13^C) or external phosphoric acid (in the case of ^31^ P). Mass spectra were recorded on a Waters ACQUITY RDa mass spectrometer.

#### (*R*)‐2‐(4,4'‐Dimethoxytrityloxy)‐1‐[(*S*)‐3‐Methoxy‐2‐(4‐Nitrophenyl)Oxazolidine‐5‐yl]Ethyl (2‐Cyanoethyl) Diisopropylphosphoramidite (1 a)

(2*R*,3*S*)‐4‐(Methoxyamino)butane‐1,2,3‐triol (**2**, 0.317 g, 2.09 mmol) and 4‐nitrobenzaldehyde (0.380 g, 2.51 mmol) were dissolved in 1,4‐dioxane (7.0 mL). The resulting mixture was acidified to approximately pH=4 with acetic acid and stirred at 55 °C for 3 h. The mixture was made slightly basic (pH=9) with triethylamine and evaporated to dryness. The crude mixture of intermediate **3 a** and its six‐membered ring isomer **3 b** was coevaporated from anhydrous pyridine (3×15 mL) and the residue dissolved in anhydrous pyridine (6.0 mL). A solution of 4,4'‐dimethoxytrityl chloride (0.781 g, 2.30 mmol) in dry CH_2_Cl_2_ (2.0 mL) was added and the resulting mixture stirred under a nitrogen atmosphere at 25 °C for 16 h. Most of the solvent was evaporated, the residue was diluted with CH_2_Cl_2_ (100 mL) and washed with saturated aqueous NaHCO_3_ (3×100 mL). The organic phase was dried over Na_2_SO_4_ and evaporated to dryness. The residue was passed through a silica gel column eluting with a mixture of Et_3_N, MeOH and CH_2_Cl_2_ (1 : 2 : 97, *v*/*v/v*). The mixture of intermediate **4 a** and its six‐membered ring isomer **4 b** thus obtained (0.360 g, 0.61 mmol) was coevaporated from anhydrous toluene (3×15 mL) and the residue was dissolved in anhydrous CH_2_Cl_2_ (4.5 mL). Et_3_N (430 μL, 3.07 mmol) and 2‐cyanoethyl‐*N*,*N*‐diisopropylchlorophosphoramidite (164 μL, 0.74 mmol) were added and the resulting mixture stirred under a nitrogen atmosphere at 25 °C for 1 h, after which it was diluted with CH_2_Cl_2_ (50 mL) and washed with saturated aqueous NaHCO_3_ (50 mL). The organic phase was dried over Na_2_SO_4_ and evaporated to dryness. The residue was purified on a silica gel column eluting with a mixture of Et_3_N, EtOAc and hexane (1 : 30 : 69, *v*/*v/v*), affording 0.340 g (20 % calculated from **2**) of the desired product **1 a** as a diastereomeric mixture. ^1^H NMR (500 MHz, CD_3_CN, major pseudoanomer of the faster‐eluting diastereomer) δ 8.17 (d, *J*=8.5 Hz, 2H, O_2_NPh−H3 and H5), 7.63 (d, *J*=4.5 Hz, 2H, O_2_NPh−H2 and H6), 7.54 (br, 2H, Ph−H2 and H6), 7.41 (m, 4H, MeOPh−H2 and H6), 7.34 (m, 2H, Ph−H3 and H5), 7.25 (t, *J*=7.3 Hz, 1H, Ph−H4), 6.90 (m, 4H, MeOPh−H3 and H5), 5.90 (s, 1H, OCHN), 4.49 (br, 1H, OC*H*CH_2_NO), 4.02 (br, 1H, DMTrOCH_2_C*H*), 3.78 (s, 6H, ArOCH_3_), 3.67 (m, 4H, POC*H*_2_CH_2_ and C*H*(CH_3_)_2_), 3.64 (s, 3H, NOCH_3_), 3.52 (m, 1H, OCHC*H*_2_NO), 3.40 (m, 2H, DMTrOCH_2_), 2.90 (m, 1H, OCHC*H*_2_NO), 2.49 (m, 2H, POCH_2_C*H*_2_), 1.33–1.13 (m, 9H, CH(C*H*_3_)_2_), 1.09 (m, 3H, CH(C*H*_3_)_2_). ^13^C NMR (126 MHz, CD_3_CN, major pseudoanomer of the faster‐eluting diastereomer) δ 158.7 (MeOPh−C4), 147.8 (O_2_NPh−C4), 146.5 (O_2_NPh−C1), 145.3 (Ph−C1), 136.1 (MeOPh−C1), 130.2 (MeOPh−C2 and C6), 128.2 (Ph−C2 and C6), 127.8 (Ph−C3 and C5), 127.6 (O_2_NPh−C3 and C5), 126.8 (Ph−C4), 123.3 (O_2_NPh−C2 and C6), 118.4 (CN), 113.0 (MeOPh−C3 and C5), 99.3 (OCHN), 86.0 (Ar_3_C), 77.4 (O*C*HCH_2_NO), 74.6 (m, DMTrOCH_2_ *C*H), 64.5 (DMTrOCH_2_), 60.1 (NOCH_3_), 58.3 (m, PO*C*H_2_CH_2_), 56.6 (OCH*C*H_2_NO), 54.9 (ArOCH_3_), 43.1 (m, *C*H(CH_3_)_2_), 24.0 (m, CH(*C*H_3_)_2_), 20.0 (m, POCH_2_ *C*H_2_). ^31^P NMR (202 MHz, CD_3_CN, major pseudoanomer of the faster‐eluting diastereomer) δ 149.4. ^1^H NMR (600 MHz, CD_3_CN, major pseudoanomer of the slower‐eluting diastereomer) δ 8.19 (d, *J*=8.6 Hz, 2H, O_2_NPh−H3 and H5), 7.65 (d, *J*=6.9 Hz, 2H, O_2_NPh−H2 and H6), 7.53 (br, 2H, Ph−H2 and H6), 7.39 (br, 4H, MeOPh−H2 and H6), 7.33 (t, *J*=7.7 Hz, 2H, Ph−H3 and H5), 7.25 (m, 1H, Ph−H4), 6.89 (m, 4H, MeOPh−H3 and H5), 5.91 (s, 1H, OCHN), 4.40 (br, 1H, OC*H*CH_2_NO), 4.02 (br, 1H, DMTrOCH_2_C*H*), 3.782 (s, 3H, ArOCH_3_), 3.776 (s, 3H, ArOCH_3_), 3.76 (m, 2H, POC*H*_2_CH_2_), 3.65 (s, 3H, NOCH_3_), 3.61 (m, 1H, C*H*(CH_3_)_2_), 3.56–3.48 (m, 2H, C*H*(CH_3_)_2_ and OCHC*H*_2_NO), 3.35 (m, 2H, DMTrOCH_2_), 2.96 (m, 1H, OCHC*H*_2_NO), 2.62 (m, 2H, POCH_2_C*H*_2_), 1.19 (d, *J*=6.1 Hz, 3H, CH(C*H*_3_)_2_), 1.16 (d, *J*=4.8 Hz, 3H, CH(C*H*_3_)_2_), 1.12 (d, *J*=6.8 Hz, 3H, CH(C*H*_3_)_2_), 1.07 (d, *J*=4.3 Hz, 3H, CH(C*H*_3_)_2_). ^13^C NMR (150 MHz, CD_3_CN, major pseudoanomer of the slower‐eluting diastereomer) δ 158.7 (MeOPh−C4), 147.8 (O_2_NPh−C4), 146.4 (O_2_NPh−C1), 145.2 (Ph−C1), 136.0 (MeOPh−C1), 130.2 (MeOPh−C2 and C6), 128.2 (Ph−C2 and C6), 127.8 (Ph−C3 and C5), 127.6 (O_2_NPh−C3 and C5), 126.8 (Ph−C4), 123.3 (O_2_NPh−C2 and C6), 118.6 (CN), 113.0 (MeOPh−C3 and C5), 99.3 (OCHN), 86.2 (Ar_3_C), 77.4 (O*C*HCH_2_NO), 74.6 (m, DMTrOCH_2_ *C*H), 65.1 (DMTrOCH_2_), 60.0 (NOCH_3_), 58.1 (m, PO*C*H_2_CH_2_), 56.4 (OCH*C*H_2_NO), 54.9 (ArOCH_3_), 43.0 (m, *C*H(CH_3_)_2_), 24.0 (m, CH(*C*H_3_)_2_), 20.1 (m, POCH_2_ *C*H_2_). ^31^P NMR (202 MHz, CD_3_CN, major pseudoanomer of the slower‐eluting diastereomer) δ 148.3. HRMS (ESI^+^‐TOF): m/z calcd for [C_42_H_51_KN_4_O_9_P]: 825.30252; found: 825.30896 [M+K]^+^.

#### (5*S*,6 *R*)‐6‐[(4,4'‐Dimethoxytrityloxy)Methyl]‐3‐Methoxy‐2‐(4‐Nitrophenyl)‐1,3‐Oxazinan‐5‐yl (2‐Cyanoethyl) Diisopropylphosphoramidite (1 b)

(2*R*,3*S*)‐4‐(Methoxyamino)butane‐1,2,3‐triol (**2**, 0.456 g, 3.02 mmol) and 4‐nitrobenzaldehyde (0.548 g, 3.63 mmol) were dissolved in 1,4‐dioxane (11.0 mL). The resulting mixture was acidified to approximately pH=4 with acetic acid and stirred at 55 °C for 17 h. The mixture was made slightly basic (pH=8) with triethylamine and evaporated to dryness. The crude mixture of intermediate **3 b** and its five‐membered ring isomer **3 a** was coevaporated from anhydrous pyridine (3×20 mL) and the residue dissolved in anhydrous pyridine (18.0 mL). A solution of 4,4'‐dimethoxytrityl chloride (0.929 g, 2.74 mmol) in dry CH_2_Cl_2_ (10.0 mL) was added and the resulting mixture stirred under a nitrogen atmosphere at 25 °C for 15 h, after which it was concentrated to approximately one third of the volume. The residue was diluted with CH_2_Cl_2_ (50 mL) and washed with saturated aqueous NaHCO_3_ (50 mL). The organic phase was dried over Na_2_SO_4_ and evaporated to dryness. The residue was passed through a silica gel column eluting with a mixture of Et_3_N, MeOH and CH_2_Cl_2_ (1 : 2 : 97, *v*/*v/v*). The mixture of intermediate **4 b** and its five‐membered ring isomer **4 a** thus obtained (0.733 g, 1.25 mmol) was coevaporated from anhydrous toluene (3×40 mL) and the residue was dissolved in anhydrous CH_2_Cl_2_ (7.0 mL). Et_3_N (851 μL, 6.11 mmol) and 2‐cyanoethyl‐*N*,*N*‐diisopropylchlorophosphoramidite (327 μL, 1.47 mmol) were added and the resulting mixture stirred under a nitrogen atmosphere at 25 °C for 1 h, after which it was diluted with CH_2_Cl_2_ (50 mL) and washed with saturated aqueous NaHCO_3_ (50 mL). The organic phase was dried over Na_2_SO_4_ and evaporated to dryness. The residue was purified on a silica gel column eluting with a mixture of Et_3_N, EtOAc and hexane (1 : 30 : 69, *v*/*v*), affording 0.318 g (13 % calculated from **2**) of the desired product **1 b** as a diastereomeric mixture. ^1^H NMR (500 MHz, CDCl_3_, major pseudoanomer of the faster‐eluting diastereomer) δ 8.31 (d, *J*=8.1 Hz, 2H, O_2_NPh−H3 and H5), 7.88 (d, *J*=7.9 Hz, 2H, O_2_NPh−H2 and H6), 7.56 (br, 2H, Ph−H2 and H6), 7.42 (m, 4H, MeOPh−H2 and H6), 7.33 (m, 2H, Ph−H3 and H5), 7.25 (m, 1H, Ph−H4), 6.87 (m, 4H, MeOPh−H3 and H5), 5.44 (s, 1H, OCHN), 4.25 (m, 1H, OC*H*CH_2_NO), 3.91 (m, 1H, DMTrOCH_2_C*H*), 3.83 (br, 6H, ArOCH_3_), 3.77 (m, 1H, OCHC*H*_2_NO), 3.53 (m, 2H, C*H*(CH_3_)_2_), 3.49 (m, 2H, DMTrOCH_2_), 3.36 (m, 2H, POC*H*_2_CH_2_), 3.13 (s, 3H, NOCH_3_), 3.03 (m, 1H, OCHC*H*_2_NO), 2.36 (m, 2H, POCH_2_C*H*_2_), 1.19 (d, *J*=6.9 Hz, 6H, CH(C*H*_3_)_2_), 1.17 (d, *J*=6.7 Hz, 6H, CH(C*H*_3_)_2_). ^13^C NMR (126 MHz, CDCl_3_, major pseudoanomer of the faster‐eluting diastereomer) δ 158.5 (MeOPh−C4), 147.8 (O_2_NPh−C4), 147.7 (O_2_NPh−C1), 144.9 (Ph−C1), 136.1 (MeOPh−C1), 130.4 (MeOPh−C2 and C6), 130.3 (MeOPh−C2 and C6), 128.5 (Ph−C2 and C6), 128.0 (O_2_NPh−C3 and C5), 127.7 (Ph−C3 and C5), 126.8 (Ph−C4), 123.0 (O_2_NPh−C2 and C6), 117.5 (CN), 113.0 (MeOPh−C3 and C5), 90.7 (OCHN), 85.8 (Ar_3_C), 81.5 (DMTrOCH_2_ *C*H), 63.6 (DMTrOCH_2_), 61.2 (O*C*HCH_2_NO), 60.7 (NOCH_3_), 58.7 (d, *J*=19.1 Hz, PO*C*H_2_CH_2_), 56.1 (OCH*C*H_2_NO), 55.3 (ArOCH_3_), 43.2 (d, *J*=12.5 Hz, *C*H(CH_3_)_2_), 24.7–24.3 (m, CH(*C*H_3_)_2_), 20.1 (d, *J*=7.2 Hz, POCH_2_ *C*H_2_). ^31^ P NMR (202 MHz, CDCl_3_, major pseudoanomer of the faster‐eluting diastereomer) δ 149.5. ^1^H NMR (500 MHz, CDCl_3_, major pseudoanomer of the slower‐eluting diastereomer) δ 8.30 (d, *J*=8.8 Hz, 2H, O_2_NPh−H3 and H5), 7.88 (d, *J*=8.1 Hz, 2H, O_2_NPh−H2 and H6), 7.57 (m, 2H, Ph−H2 and H6), 7.41 (m, 4H, MeOPh−H2 and H6), 7.30 (m, 2H, Ph−H3 and H5), 7.24 (m, 1H, Ph−H4), 6.86 (m, 4H, MeOPh−H3 and H5), 5.45 (s, 1H, OCHN), 4.13 (m, 1H, OC*H*CH_2_NO), 3.93 (m, 2H, DMTrOCH_2_C*H* and OCHC*H*_2_NO), 3.82 (m, 6H, ArOCH_3_), 3.68 (m, 2H, POC*H*_2_CH_2_), 3.57–3.34 (m, 4H, DMTrOCH_2_ and C*H*(CH_3_)_2_), 3.16 (s, 3H, NOCH_3_), 3.04 (m, 1H, OCHC*H*_2_NO), 2.61 (m, 2H, POCH_2_C*H*_2_), 1.13 (d, *J*=6.8 Hz, 6H, CH(C*H*_3_)_2_), 0.95 (d, *J*=6.8 Hz, 6H, CH(C*H*_3_)_2_). ^13^C NMR (126 MHz, CDCl_3_, major pseudoanomer of the slower‐eluting diastereomer) δ 158.4 (MeOPh−C4), 147.7 (O_2_NPh−C4), 147.6 (O_2_NPh−C1), 145.1 (Ph−C1), 136.2 (MeOPh−C1), 130.3 (MeOPh−C2 and C6), 130.2 (MeOPh−C2 and C6), 128.4 (Ph−C2 and C6), 128.1 (O_2_NPh−C3 and C5), 127.6 (Ph−C3 and C5), 126.7 (Ph−C4), 123.0 (O_2_NPh−C2 and C6), 117.6 (CN), 113.0 (MeOPh−C3 and C5), 90.7 (OCHN), 85.8 (Ar_3_C), 81.3 (DMTrOCH_2_ *C*H), 63.8 (DMTrOCH_2_), 62.3 (O*C*HCH_2_NO), 60.7 (NOCH_3_), 57.9 (d, *J*=20.4 Hz, PO*C*H_2_CH_2_), 56.3 (OCH*C*H_2_NO), 55.22 (ArOCH_3_), 55.18 (ArOCH_3_), 43.1 (m, *C*H(CH_3_)_2_), 24.9–24.3 (m, CH(*C*H_3_)_2_), 20.4 (m, POCH_2_ *C*H_2_). ^31^P NMR (202 MHz, CDCl_3_, major pseudoanomer of the slower‐eluting diastereomer) δ 148.9. HRMS (ESI^+^‐TOF): m/z calcd for [C_42_H_51_N_4_NaO_9_P]: 809.32859; found: 809.32908 [M+Na]^+^.

#### 1‐(2,2‐Diethoxyethyl)‐2‐Methylbenzimidazole (6)

2‐Methylbenzimidazole (**5**, 0.500 g, 3.78 mmol) was dissolved in anhydrous DMF (10 mL). Cs_2_CO_3_ (2.468 g, 7.577 mmol) was added and the resulting mixture was refluxed under a nitrogen atmosphere for 30 min. 2‐bromo‐1,1‐diethoxyethane (0.8747 mL, 5.814 mmol) was added gradually and the mixture was stirred at 90 °C for 1.5 h, after which it was cooled to room temperature and filtered. The filtrate was evaporated and the residue purified on a silica gel column eluting with a mixture of MeOH and CH_2_Cl_2_ (1 : 20, *v*/*v*), affording 0.322 g (34 %) of the desired product **6**. ^1^H NMR (500 MHz, D_2_O) δ 7.51 (m, 1H, H7), 7.38 (m, 1H, H4), 7.22 (m, 2H, H5 and H6), 4.76 (t, *J*=5.3 Hz, 1H, OCHO), 4.11 (d, *J*=5.3 Hz, 2H, NCH_2_), 3.62 (q, *J*=7.1 Hz, 1H, OCH_2_), 3.60 (q, *J*=7.1 Hz, 1H, OCH_2_), 3.31 (q, *J*=7.1 Hz, 1H, OCH_2_), 3.29 (q, *J*=7.1 Hz, 1H, OCH_2_), 2.47 (s, 3H, Ar‐CH_3_), 0.90 (t, *J*=7.1 Hz, 6H, CH_2_C*H*_3_). ^13^C NMR (126 MHz, D_2_O) δ 154.2 (C1), 140.5 (C8), 134.9 (C9), 122.6 (C5 or C6), 122.5 (C6 or C5), 117.5 (C4), 110.6 (C7), 101.0 (OCHO), 65.4 (OCH_2_), 46.5 (NCH_2_), 14.2 (CH_2_ *C*H_3_), 12.9 (Ar‐CH_3_). HRMS (ESI^+^‐TOF): m/z calcd for [C_14_H_21_N_2_O_2_]: 249.15975; found: 249.16015 [M + H]^+^.

#### 2‐(2‐Methylbenzimidazol‐1‐yl)Acetaldehyde (fmB)

Starting material **6** (0.322 g, 1.30 mmol) was dissolved in 1.0 M aqueous HCl (10 mL). The solution was stirred at 90 °C for 1 h, after which it was allowed to cool to room temperature. MeOH (10 mL) was added, the mixture was neutralized with 1.0 M aqueous NaOH (9.9 mL) and evaporated to dryness. The residue was suspended in saturated aqueous NaHCO_3_ (80 mL) and extracted with CH_2_Cl_2_ (3×80 mL). The organic phase was dried over anhydrous Na_2_SO_4_ and evaporated to dryness. The residue was purified on a silica gel column eluting with a mixture of MeOH and CH_2_Cl_2_ (1 : 20, *v*/*v*), affording 0.0415 g (18 %) of the desired product **fmB**. ^1^H NMR (600 MHz, D_2_O, geminal hydrate) δ 7.54 (d, *J*=7.5 Hz, 1H, H7), 7.46 (d, *J*=7.5 Hz, 1H, H4), 7.25 (m, 2H, H5 and H6), 5.31 (t, *J*=4.5 Hz, 1H, OCHO), 4.17 (d, *J*=5.1 Hz, 2H, CH_2_), 2.52 (s, 3H, CH_3_). ^13^C NMR (150 MHz, D_2_O, geminal hydrate) δ 154.3 (C1), 140.6 (C8), 135.1 (C9), 122.6 (C5 or C6), 122.5 (C6 or C5), 117.5 (C4), 110.6 (C7), 88.1 (OCHO), 48.9 (CH_2_), 12.9 (CH_3_). HRMS (ESI^+^‐TOF): m/z calcd for [C_10_H_11_N_2_O]: 175.08659; found: 175.08347 [M+H]^+^.

#### Oligonucleotide Synthesis

All modified oligonucleotides were synthesized by an ÄKTA Oligopilot plus 10 DNA/RNA synthesizer in 1 μmol scale on CPG support. Standard phosphoramidite strategy was employed, with 5‐(benzylthio)‐1H‐tetrazole as the activator. A 120 s recycling time was used for both DNA and 2'‐OMe‐RNA building blocks, as well as the MOGNA and MOANA building blocks **1 a** and **1 b**. Coupling yields based on trityl response monitoring were 77–99 % for the modified building blocks **1 a** and **1 b** and near‐quantitative for the other building blocks. On completion of chain assembly, the linker between the oligonucleotide and the solid support as well as the base and phosphate protections were removed by incubation in 25 % aqueous NH_3_ at 55 °C for 16 h. The crude product mixtures were fractioned by RP‐HPLC on a Hypersil ODS C18 column (250×10 mm, 5 μm) eluting with a linear gradient of MeCN in 50 mmol L^−1^ aqueous triethylammonium acetate (chromatograms presented in the Supporting Information, along with the detailed solvent program used in each case). Fractions containing the full‐length oligonucleotide with the p‐nitrobenzylidene protection intact were acidified (pH=4) with AcOH, incubated at room temperature for 5 d and purified again by RP‐HPLC. The purified oligonucleotides were characterized by ESI‐TOF‐MS (mass spectra presented in the Supporting Information) and quantified UV spectrophotometrically using molar absorptivities calculated by an implementation of the nearest‐neighbors method.

#### UV Melting Temperature Measurements

Samples of the naked hairpin oligonucleotides by diluting the appropriate stock solutions to 1.0 μM concentration with 20 mM cacodylate buffer (pH=5.5 or 7.4), the ionic strength of which was adjusted to 0.10 M with NaClO_4_. For the base‐filling product mixtures, the hairpin oligonucleotide and 2.0 equivalents of the aldehyde **fmA** were first incubated in a small volume (approximately 20 μL) of 20 mM cacodylate buffer at 23 °C and pH=5.5 for 120 h. The samples were then diluted to the final volume (400 μL) with the same buffer as used for the naked hairpin oligonucleotides. For measurement of the UV melting profiles, all samples were transferred to quartz cuvettes with optical path length of 10 mm. The profiles were acquired on a PerkinElmer Lambda UV/vis spectrophotometer by measuring the absorbance at 260 nm at 0.5 °C intervals. Three heating and cooling ramps (10–90 °C, 0.5 °C min^−1^) were run by a Peltier temperature control unit. Melting temperatures were obtained as inflection points on the sigmoidal plots of absorbance as a function of temperature.

#### Dynamic Combinatorial Chemistry

The DCC reaction mixtures were prepared by diluting the appropriate oligonucleotide to 1.0 μM concentration and a mixture of the aldehydes **fmA**, **fmC**, **fmG**, **fmT** and **fmB** to either 20 μM (for single‐stranded oligonucleotides) or 2.0 μM (for other systems) concentration with 20 mM cacodylate buffer (pH=5.5), the ionic strength of which was adjusted to 0.10 M with NaClO_4_. The mixtures were incubated at 23 °C for at least 120 h, after which their compositions were determined by UPLC‐ESI‐MS (Figures S86–S119 in the Supporting Information). Quantification of the naked oligonucleotides and their various base‐filling products was based on integration of the extracted ion ([M–2H]^2−^ for the 11‐mers, [M–3H]^3−^ for the Hoogsteen strands and [M–4H]^4−^ for the hairpins) chromatograms, assuming equal ionizability for closely related structures.

## Conflict of Interests

The authors declare no conflict of interest.

1

## Supporting information

As a service to our authors and readers, this journal provides supporting information supplied by the authors. Such materials are peer reviewed and may be re‐organized for online delivery, but are not copy‐edited or typeset. Technical support issues arising from supporting information (other than missing files) should be addressed to the authors.

Supporting Information

## Data Availability

The data that support the findings of this study are available in the supplementary material of this article.
