# Supplementary material for: The Impact of Secondary Structure on the Base‐Filling of N‐Methoxy‐1,3‐Oxazinane (MOANA) and N‐Methoxy‐1,3‐Oxazolidine Glycol Nucleic Acid (MOGNA) Oligonucleotides
Source: Chembiochem. 2024 Oct 27;26(1):e202400666. doi: 10.1002/cbic.202400666 (PMC11727013; doi:10.1002/cbic.202400666)
Supplement: Supplementary file 1 — Supporting Information [file CBIC-26-e202400666-s001.pdf]

ChemBioChem

Supporting Information

The Impact of Secondary Structure on the Base-Filling of *N*-Methoxy-1,3-Oxazinane (MOANA) and *N*-Methoxy-1,3-Oxazolidine Glycol Nucleic Acid (MOGNA) Oligonucleotides

Mark N. K. Afari, Ninna Heikinmäki, Pasi Virta, and Tuomas Lönnberg*

Contents

Figure S1. ^{1}H NMR spectrum of compound 1a (faster-eluting diastereomer, 500 MHz, CD_3CN). S5
Figure S2. ^{13}C NMR spectrum of compound 1a (faster-eluting diastereomer, 126 MHz, CD_3CN). S7
Figure S3. ^{31}P NMR spectrum of compound 1a (faster-eluting diastereomer, 202 MHz, CD_3CN). S9
Figure S4. HMBC spectrum of compound 1a (faster-eluting diastereomer, CD_3CN). S10
Figure S5. ^{1}H NMR spectrum of compound 1a (slower-eluting diastereomer, 500 MHz, CD_3CN). S11
Figure S6. ^{13}C NMR spectrum of compound 1a (slower-eluting diastereomer, 126 MHz, CD_3CN). S13
Figure S7. ^{31}P NMR spectrum of compound 1a (slower-eluting diastereomer, 202 MHz, CD_3CN). S15
Figure S8. HMBC spectrum of compound 1a (slower-eluting diastereomer, CD_3CN). S16
Figure S9. ^{1}H NMR spectrum of compound 1b (faster-eluting diastereomer, 500 MHz, $CDCl_3$). S17
Figure S10. ^{13}C NMR spectrum of compound 1b (faster-eluting diastereomer, 126 MHz, $CDCl_3$). S19
Figure S11. ^{31}P NMR spectrum of compound 1b (faster-eluting diastereomer, 202 MHz, $CDCl_3$). S21
Figure S12. ^{1}H NMR spectrum of compound 1b (slower-eluting diastereomer, 500 MHz, $CDCl_3$). S22
Figure S13. ^{13}C NMR spectrum of compound 1b (slower-eluting diastereomer, 126 MHz, $CDCl_3$). S24
Figure S14. ^{31}P NMR spectrum of compound 1b (slower-eluting diastereomer, 202 MHz, $CDCl_3$). S26
Figure S15. ^{1}H NMR spectrum of compound 6 (600 MHz, D_2O). S27
Figure S16. ^{13}C NMR spectrum of compound 6 (150 MHz, D_2O). S29
Figure S17. ^{1}H NMR spectrum of compound fmB (600 MHz, D_2O). S30
Figure S18. ^{13}C NMR spectrum of compound fmB (150 MHz, D_2O). S32
Figure S19. RP-HPLC traces of A) crude product mixture of the synthesis of oligonucleotide ON3 and B) the purified product after treatment with acetic acid. S33
Figure S20. A) UV and extracted ion UPLC traces and B) mass spectra of oligonucleotide ON3. S34
Figure S21. RP-HPLC traces of A) crude product mixture of the synthesis of oligonucleotide ON4a and B) the purified product after treatment with acetic acid. S35
Figure S22. A) UV and extracted ion UPLC traces and B) mass spectra of oligonucleotide ON4a. S36
Figure S23. RP-HPLC traces of A) crude product mixture of the synthesis of oligonucleotide ON4c and B) the purified product after treatment with acetic acid. S37
Figure S24. A) UV and extracted ion UPLC traces and B) mass spectra of oligonucleotide ON4c. S38
Figure S25. RP-HPLC traces of A) crude product mixture of the synthesis of oligonucleotide ON4g and B) the purified product after treatment with acetic acid. S39
Figure S26. A) UV and extracted ion UPLC traces and B) mass spectra of oligonucleotide ON4g. S40
Figure S27. RP-HPLC traces of A) crude product mixture of the synthesis of oligonucleotide ON4t and B) the purified product after treatment with acetic acid. S41
Figure S28. A) UV and extracted ion UPLC traces and B) mass spectra of oligonucleotide ON4t. S42
Figure S29. RP-HPLC traces of A) crude product mixture of the synthesis of oligonucleotide ON4s and B) the purified product after treatment with acetic acid. S43
Figure S30. A) UV and extracted ion UPLC traces and B) mass spectra of oligonucleotide ON4s. S44
Figure S31. RP-HPLC traces of A) crude product mixture of the synthesis of oligonucleotide ON5 and B) the purified product after treatment with acetic acid. S45
Figure S32. A) UV and extracted ion UPLC traces and B) mass spectra of oligonucleotide ON5. S46
Figure S33. RP-HPLC traces of A) crude product mixture of the synthesis of oligonucleotide ON6a and B) the purified product after treatment with acetic acid. S47
Figure S34. A) UV and extracted ion UPLC traces and B) mass spectra of oligonucleotide ON6a. S48
Figure S35. RP-HPLC traces of A) crude product mixture of the synthesis of oligonucleotide ON6c and B) the purified product after treatment with acetic acid. S49
Figure S36. A) UV and extracted ion UPLC traces and B) mass spectra of oligonucleotide ON6c. S50
Figure S37. RP-HPLC traces of A) crude product mixture of the synthesis of oligonucleotide ON6g and B) the purified product after treatment with acetic acid. S51
Figure S38. A) UV and extracted ion UPLC traces and B) mass spectra of oligonucleotide ON6g. S52
Figure S39. RP-HPLC traces of A) crude product mixture of the synthesis of oligonucleotide ON6u and B) the purified product after treatment with acetic acid. S53

Contents (continued)

Figure S38. A) UV and extracted ion UPLC traces and B) mass spectra of oligonucleotide ON6u. S54
Figure S41. RP-HPLC traces of A) crude product mixture of the synthesis of oligonucleotide ON6s and B) the purified product after treatment with acetic acid. S55
Figure S42. A) UV and extracted ion UPLC traces and B) mass spectra of oligonucleotide ON6s. S56
Figure S43. RP-HPLC traces of A) crude product mixture of the synthesis of oligonucleotide ON7 and B) the purified product after treatment with acetic acid. S57
Figure S44. A) UV and extracted ion UPLC traces and B) mass spectra of oligonucleotide ON7. S58
Figure S45. RP-HPLC traces of A) crude product mixture of the synthesis of oligonucleotide ON8a and B) the purified product after treatment with acetic acid. S59
Figure S46. A) UV and extracted ion UPLC traces and B) mass spectra of oligonucleotide ON8a. S60
Figure S47. RP-HPLC traces of A) crude product mixture of the synthesis of oligonucleotide ON8c and B) the purified product after treatment with acetic acid. S61
Figure S48. A) UV and extracted ion UPLC traces and B) mass spectra of oligonucleotide ON8c. S62
Figure S49. RP-HPLC traces of A) crude product mixture of the synthesis of oligonucleotide ON8g and B) the purified product after treatment with acetic acid. S63
Figure S50. A) UV and extracted ion UPLC traces and B) mass spectra of oligonucleotide ON8g. S64
Figure S51. RP-HPLC traces of A) crude product mixture of the synthesis of oligonucleotide ON8u and B) the purified product after treatment with acetic acid. S65
Figure S52. A) UV and extracted ion UPLC traces and B) mass spectra of oligonucleotide ON8u. S66
Figure S53. RP-HPLC traces of A) crude product mixture of the synthesis of oligonucleotide ON8s and B) the purified product after treatment with acetic acid. S67
Figure S54. A) UV and extracted ion UPLC traces and B) mass spectra of oligonucleotide ON8s. S68
Figure S55. RP-HPLC traces of A) crude product mixture of the synthesis of oligonucleotide ON9 and B) the purified product after treatment with acetic acid. S69
Figure S56. A) UV and extracted ion UPLC traces and B) mass spectra of oligonucleotide ON9. S70
Figure S57. RP-HPLC traces of A) crude product mixture of the synthesis of oligonucleotide ON10 and B) the purified product after treatment with acetic acid. S71
Figure S58. A) UV and extracted ion UPLC traces and B) mass spectra of oligonucleotide ON10. S72
Figure S59. UV melting profile of 1.0 µM hairpin ON4a; pH = 5.5. S73
Figure S60. UV melting profile of 1.0 µM hairpin ON4c; pH = 5.5. S73
Figure S61. UV melting profile of 1.0 µM hairpin ON4g; pH = 5.5. S74
Figure S62. UV melting profile of 1.0 µM hairpin ON4t; pH = 5.5. S74
Figure S63. UV melting profile of 1.0 µM hairpin ON4s; pH = 5.5. S75
Figure S64. UV melting profile of 1.0 µM hairpin ON6a; pH = 5.5. S76
Figure S65. UV melting profile of 1.0 µM hairpin ON6c; pH = 5.5. S76
Figure S66. UV melting profile of 1.0 µM hairpin ON6g; pH = 5.5. S77
Figure S67. UV melting profile of 1.0 µM hairpin ON6u; pH = 5.5. S77
Figure S68. UV melting profile of 1.0 µM hairpin ON6s; pH = 5.5. S78
Figure S69. UV melting profile of 1.0 µM hairpin ON8a; pH = 5.5. S79
Figure S70. UV melting profile of 1.0 µM hairpin ON8c; pH = 5.5. S79
Figure S71. UV melting profile of 1.0 µM hairpin ON8g; pH = 5.5. S80
Figure S72. UV melting profile of 1.0 µM hairpin ON8u; pH = 5.5. S80
Figure S73. UV melting profile of 1.0 µM hairpin ON8s; pH = 5.5. S81
Figure S74. UV melting profile of 1.0 µM triplex ON11t•ON12a*ON9; pH = 5.5. S82
Figure S75. UV melting profile of 1.0 µM triplex ON11g•ON12c*ON9; pH = 5.5. S82
Figure S76. UV melting profile of 1.0 µM triplex ON11c•ON12g*ON9; pH = 5.5. S83
Figure S77. UV melting profile of 1.0 µM triplex ON11a•ON12t*ON9; pH = 5.5. S83
Figure S78. UV melting profile of 1.0 µM triplex ON11t•ON12a*ON10; pH = 5.5. S84
Figure S79. UV melting profile of 1.0 µM triplex ON11g•ON12c*ON10; pH = 5.5. S84

Contents (continued)

Figure S80. UV melting profile of 1.0 µM triplex ON11c•ON12g*ON10; pH = 5.5.	S85
Figure S81. UV melting profile of 1.0 µM triplex ON11a•ON12t*ON10; pH = 5.5.	S85
Figure S82. Time-dependent mole fraction of oligonucleotide ON2t and its 9-formylmethyladenine adduct.	S86
Figure S83. Time-dependent mole fraction of oligonucleotide ON4t and its 9-formylmethyladenine adduct.	S86
Figure S84. Time-dependent mole fraction of oligonucleotide ON6u and its 9-formylmethyladenine adduct.	S87
Figure S85. Time-dependent mole fraction of oligonucleotide ON8u and its 9-formylmethyladenine adduct.	S87
Figure S86. UV and extracted ion RP-UPLC traces of the DCC product mixture of ON1.	S88
Figure S87. UV and extracted ion RP-UPLC traces of the DCC product mixture of ON2a.	S89
Figure S88. UV and extracted ion RP-UPLC traces of the DCC product mixture of ON2c.	S90
Figure S89. UV and extracted ion RP-UPLC traces of the DCC product mixture of ON2g.	S91
Figure S90. UV and extracted ion RP-UPLC traces of the DCC product mixture of ON2t.	S92
Figure S91. UV and extracted ion RP-UPLC traces of the DCC product mixture of ON2s.	S93
Figure S92. UV and extracted ion RP-UPLC traces of the DCC product mixture of ON3.	S94
Figure S93. UV and extracted ion RP-UPLC traces of the DCC product mixture of ON4a.	S95
Figure S94. UV and extracted ion RP-UPLC traces of the DCC product mixture of ON4c.	S96
Figure S95. UV and extracted ion RP-UPLC traces of the DCC product mixture of ON4g.	S97
Figure S96. UV and extracted ion RP-UPLC traces of the DCC product mixture of ON4t.	S98
Figure S97. UV and extracted ion RP-UPLC traces of the DCC product mixture of ON4s.	S99
Figure S98. UV and extracted ion RP-UPLC traces of the DCC product mixture of ON5.	S100
Figure S99. UV and extracted ion RP-UPLC traces of the DCC product mixture of ON6a.	S101
Figure S100. UV and extracted ion RP-UPLC traces of the DCC product mixture of ON6c.	S102
Figure S101. UV and extracted ion RP-UPLC traces of the DCC product mixture of ON6g.	S103
Figure S102. UV and extracted ion RP-UPLC traces of the DCC product mixture of ON6u.	S104
Figure S103. UV and extracted ion RP-UPLC traces of the DCC product mixture of ON6s.	S105
Figure S104. UV and extracted ion RP-UPLC traces of the DCC product mixture of ON7.	S106
Figure S105. UV and extracted ion RP-UPLC traces of the DCC product mixture of ON8a.	S107
Figure S106. UV and extracted ion RP-UPLC traces of the DCC product mixture of ON8c.	S108
Figure S107. UV and extracted ion RP-UPLC traces of the DCC product mixture of ON8g.	S109
Figure S108. UV and extracted ion RP-UPLC traces of the DCC product mixture of ON8u.	S110
Figure S109. UV and extracted ion RP-UPLC traces of the DCC product mixture of ON8s.	S111
Figure S110. UV and extracted ion RP-UPLC traces of the DCC product mixture of 9.	S112
Figure S111. UV and extracted ion RP-UPLC traces of the DCC product mixture of triplex ON11t•ON12a*ON9.	S113
Figure S112. UV and extracted ion RP-UPLC traces of the DCC product mixture of triplex ON11g•ON12c*ON9.	S114
Figure S113. UV and extracted ion RP-UPLC traces of the DCC product mixture of triplex ON11c•ON12g*ON9.	S115
Figure S114. UV and extracted ion RP-UPLC traces of the DCC product mixture of triplex ON11a•ON12t*ON9.	S116
Figure S115. UV and extracted ion RP-UPLC traces of the DCC product mixture of 10.	S117
Figure S116. UV and extracted ion RP-UPLC traces of the DCC product mixture of triplex ON11t•ON12a*ON10.	S118
Figure S117. UV and extracted ion RP-UPLC traces of the DCC product mixture of triplex ON11g•ON12c*ON10.	S119

Contents (continued)

Figure S118. UV and extracted ion RP-UPLC traces of the DCC product mixture of triplex ON11c•ON12g*ON10. S120
Figure S119. UV and extracted ion RP-UPLC traces of the DCC product mixture of triplex ON11a•ON12t*ON10. S121
Figure S120. UV melting profile of 1.0 µM hairpin ON2a naked and functionalized with aldehyde fmA; pH = 7.4. S122
Figure S121. UV melting profile of 1.0 µM hairpin ON2c naked and functionalized with aldehyde fmA; pH = 7.4. S122
Figure S122. UV melting profile of 1.0 µM hairpin ON2g naked and functionalized with aldehyde fmA; pH = 7.4. S123
Figure S123. UV melting profile of 1.0 µM hairpin ON2t naked and functionalized with aldehyde fmA; pH = 7.4. S123
Figure S124. UV melting profile of 1.0 µM hairpin ON2s naked and functionalized with aldehyde fmA; pH = 7.4. S124
Figure S125. UV melting profile of 1.0 µM hairpin ON4a naked and functionalized with aldehyde fmA; pH = 7.4. S125
Figure S126. UV melting profile of 1.0 µM hairpin ON4c naked and functionalized with aldehyde fmA; pH = 7.4. S125
Figure S127. UV melting profile of 1.0 µM hairpin ON4g naked and functionalized with aldehyde fmA; pH = 7.4. S126
Figure S128. UV melting profile of 1.0 µM hairpin ON4t naked and functionalized with aldehyde fmA; pH = 7.4. S126
Figure S129. UV melting profile of 1.0 µM hairpin ON4s naked and functionalized with aldehyde fmA; pH = 7.4. S127
Figure S130. UV melting profile of 1.0 µM hairpin ON6a; pH = 7.4. S128
Figure S131. UV melting profile of 1.0 µM hairpin ON6c; pH = 7.4. S128
Figure S132. UV melting profile of 1.0 µM hairpin ON6g; pH = 7.4. S129
Figure S133. UV melting profile of 1.0 µM hairpin ON6u; pH = 7.4. S129
Figure S134. UV melting profile of 1.0 µM hairpin ON6s; pH = 7.4. S130
Figure S135. UV melting profile of 1.0 µM hairpin ON8a; pH = 7.4. S131
Figure S136. UV melting profile of 1.0 µM hairpin ON8c; pH = 7.4. S131
Figure S137. UV melting profile of 1.0 µM hairpin ON8g; pH = 7.4. S132
Figure S138. UV melting profile of 1.0 µM hairpin ON8u; pH = 7.4. S132
Figure S139. UV melting profile of 1.0 µM hairpin ON8s; pH = 7.4. S133

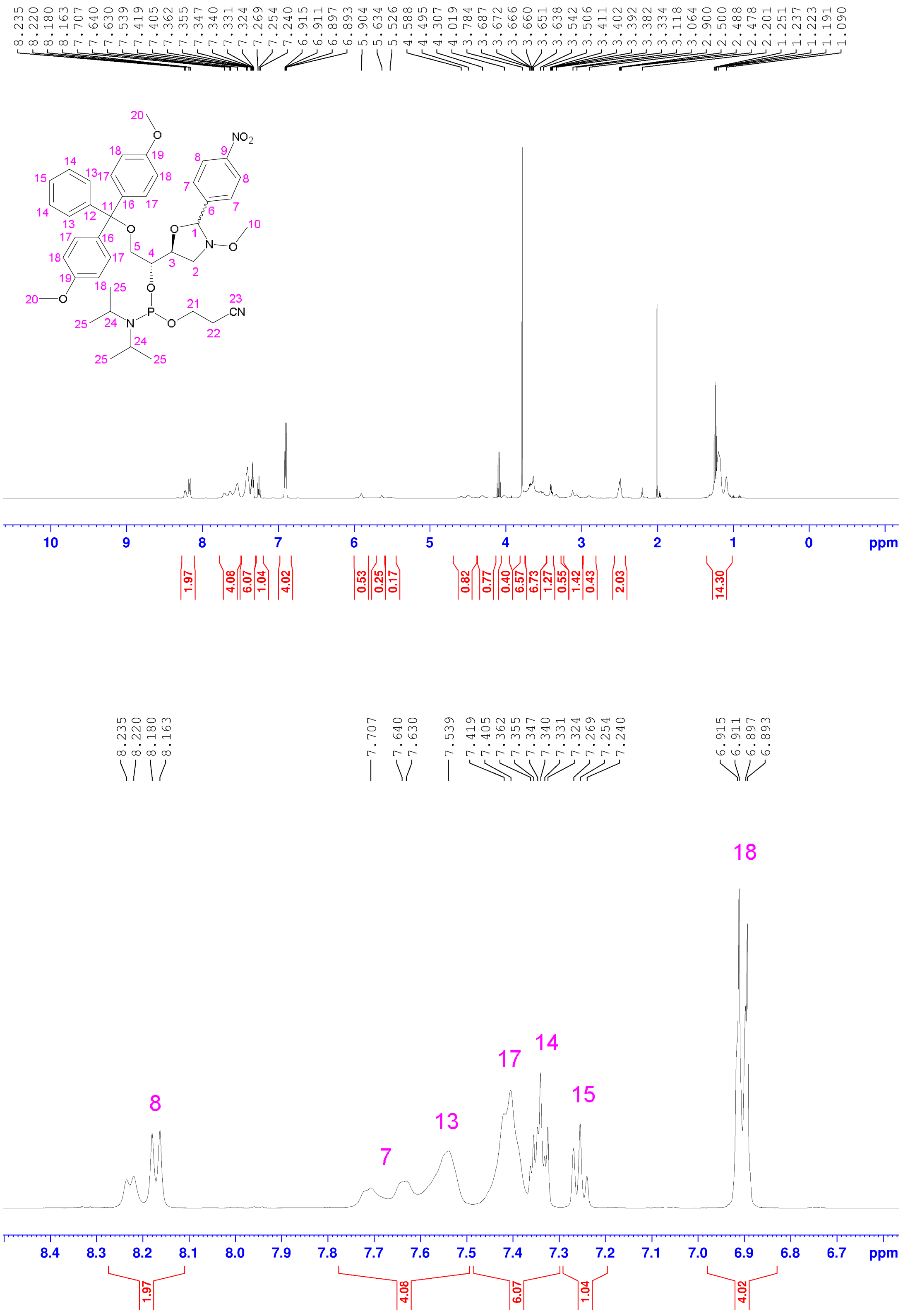

Figure S1. ^{1}H NMR spectrum of compound 1a (faster-eluting diastereomer, 500 MHz, CD_3CN).

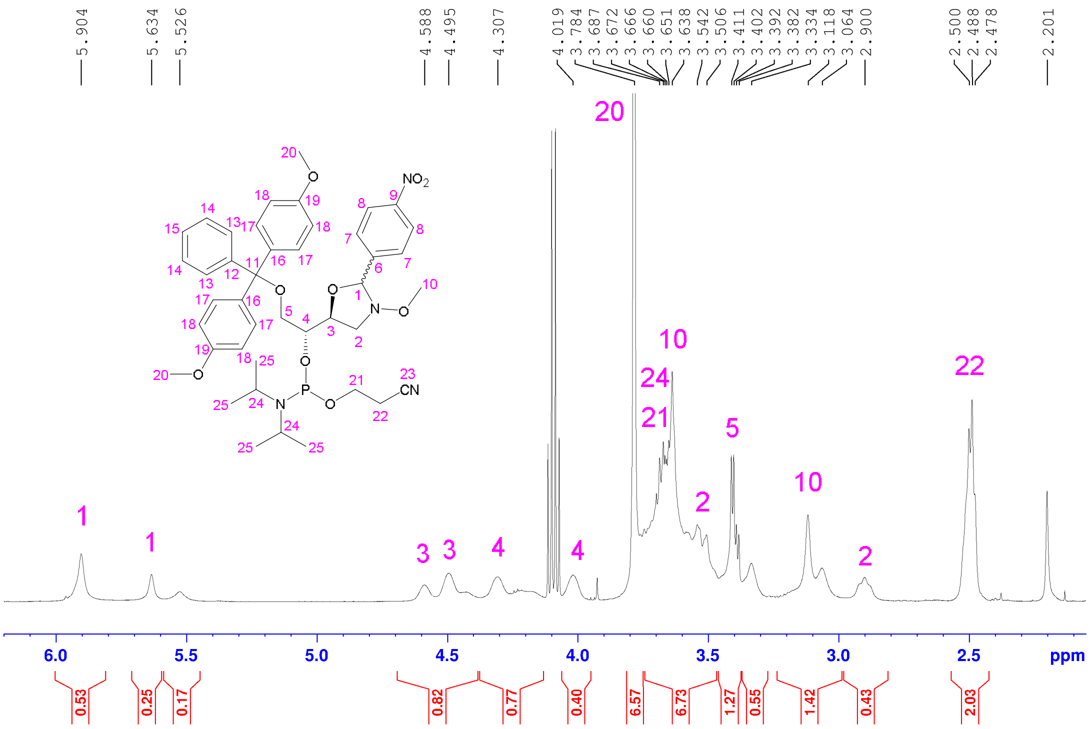

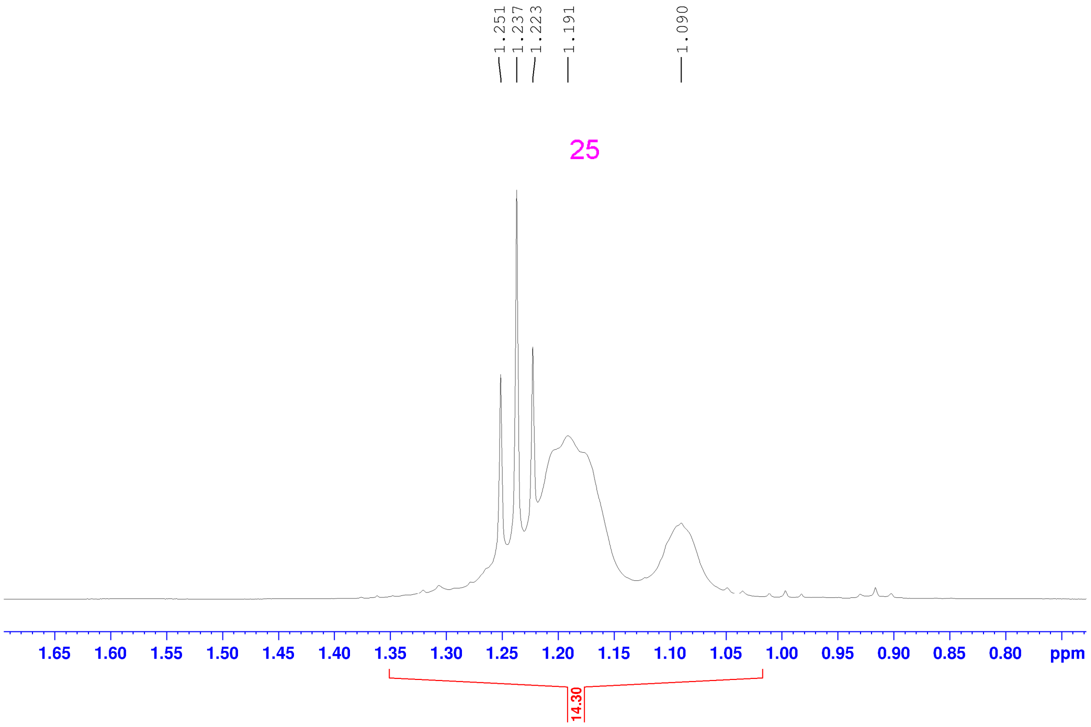

Figure S1 (continued). ^{1}H NMR spectrum of compound 1a (faster-eluting diastereomer, 500 MHz, CD_3CN).

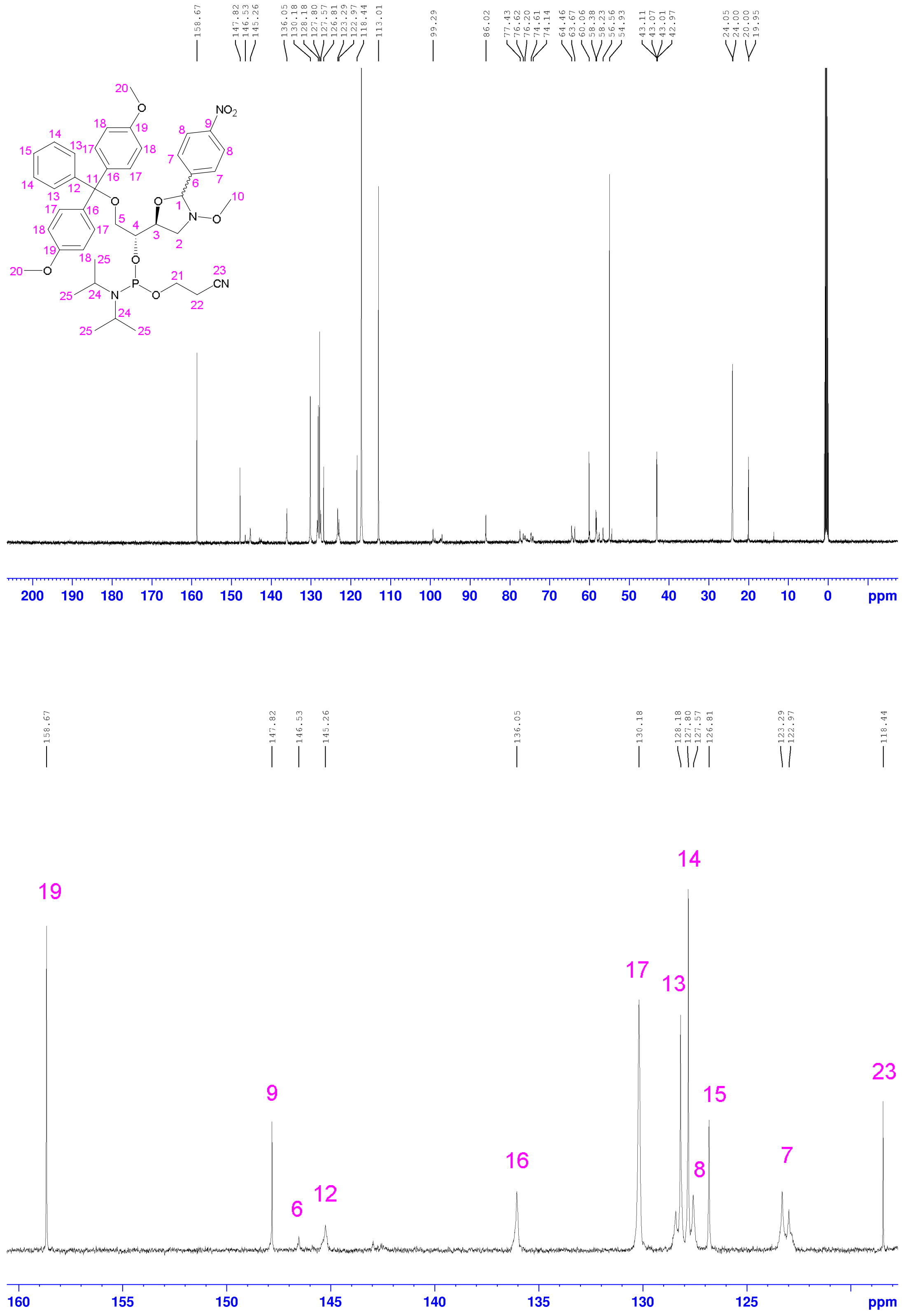

Figure S2. ^{13}C NMR spectrum of compound 1a (faster-eluting diastereomer, 126 MHz, CD_3CN).

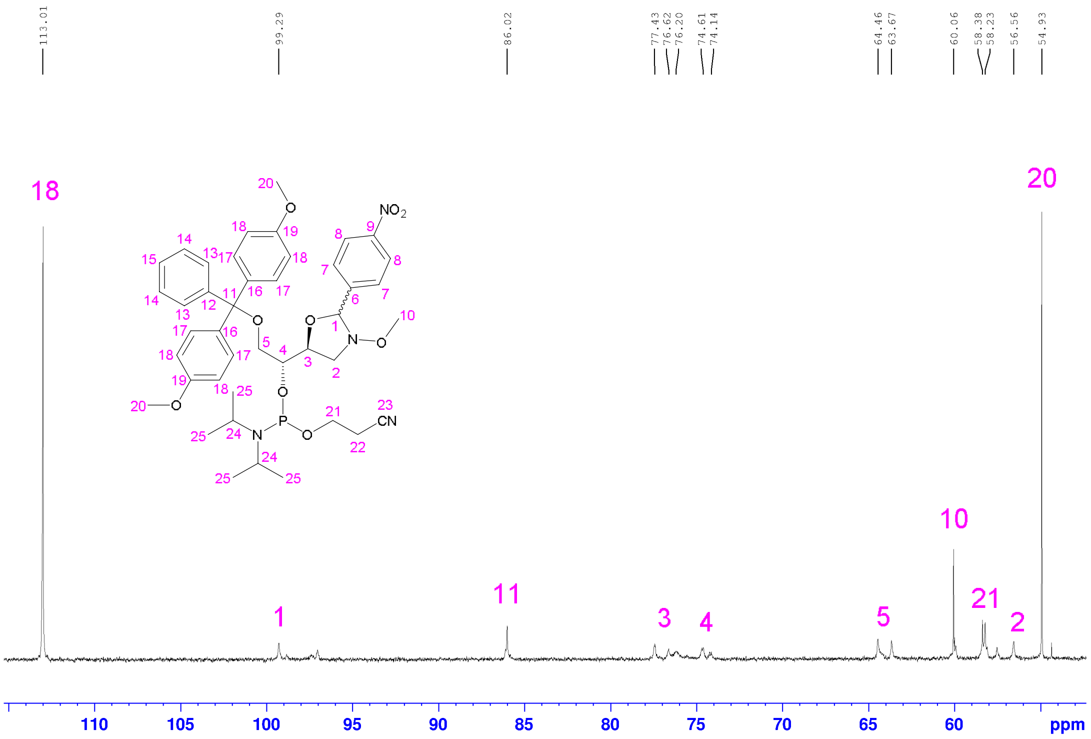

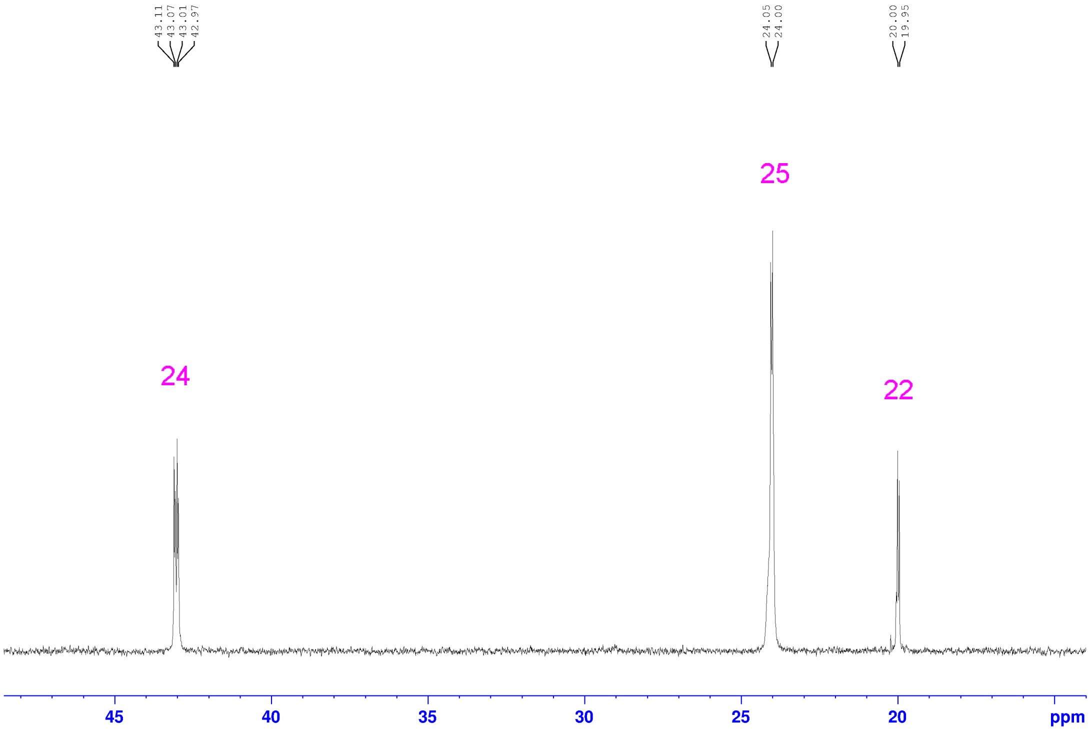

Figure S2 (continued). ^{13}C NMR spectrum of compound 1a (faster-eluting diastereomer, 126 MHz, CD_3CN).

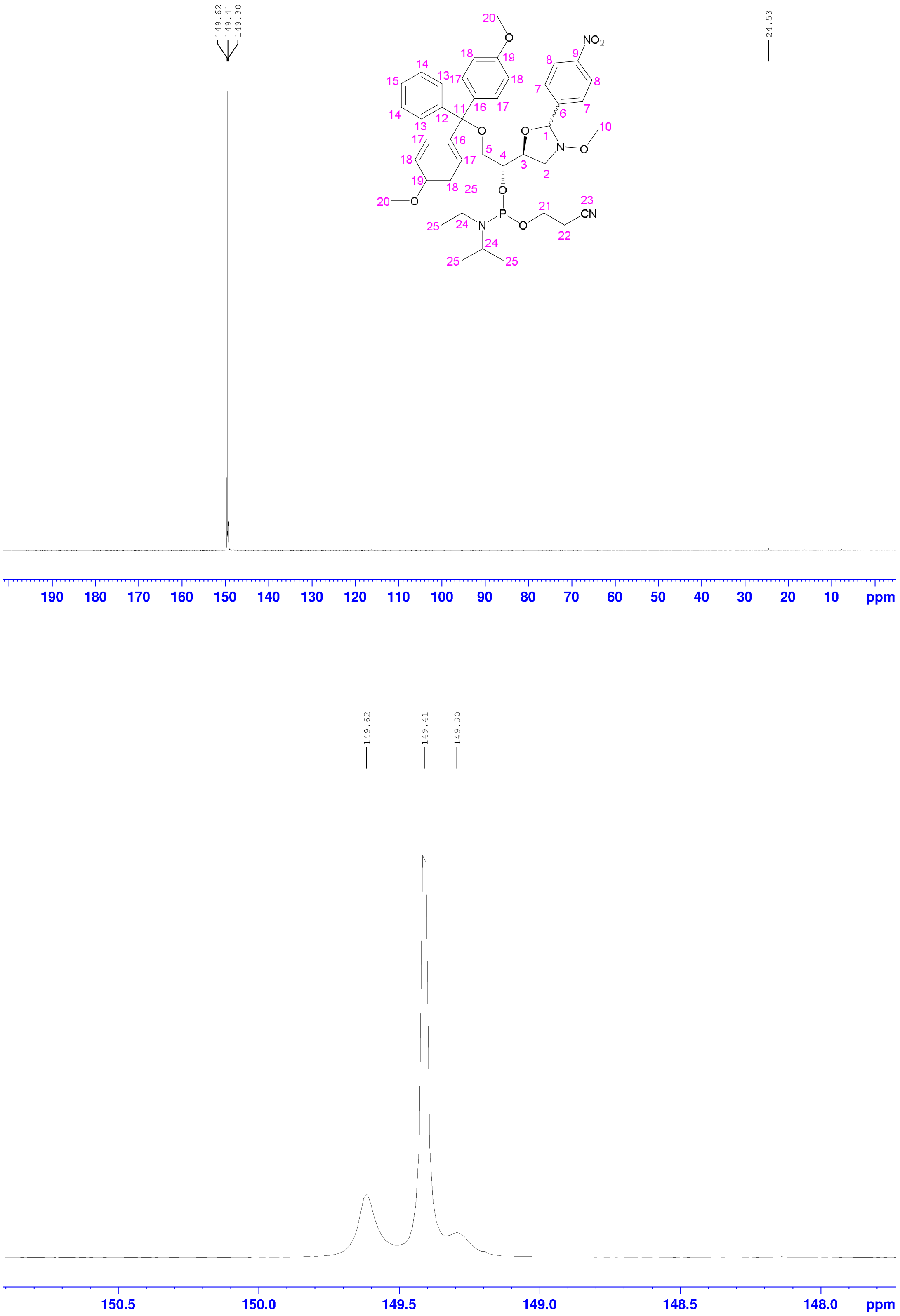

Figure S3. ^{31}P NMR spectrum of compound 1a (faster-eluting diastereomer, 202 MHz, CD_3CN).

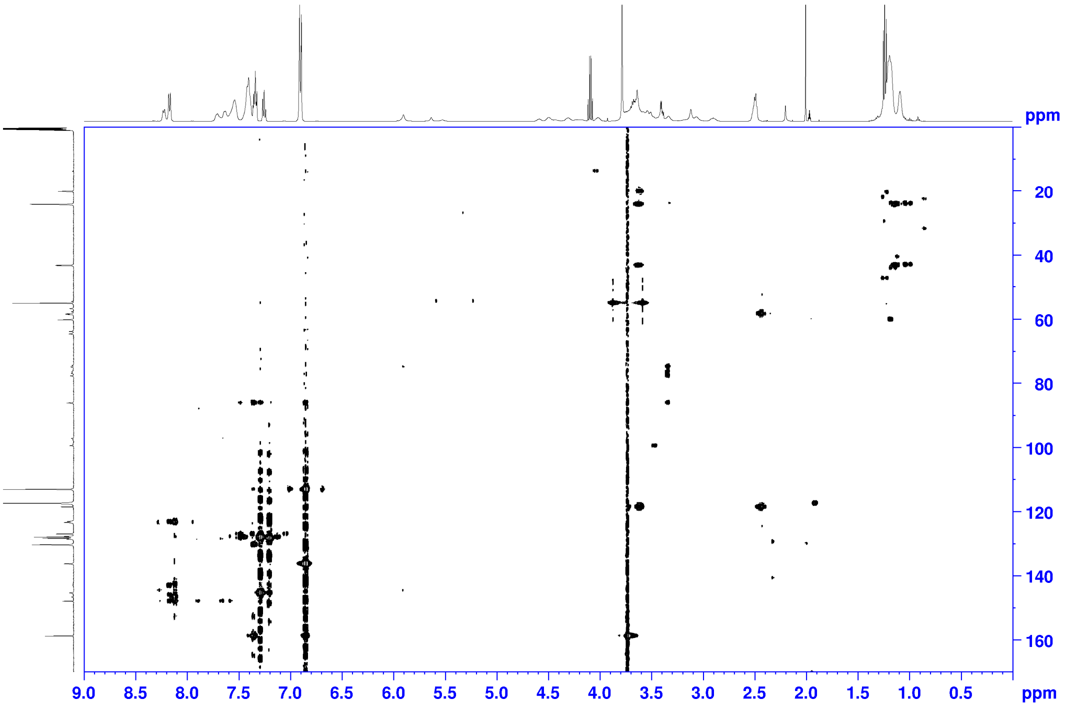

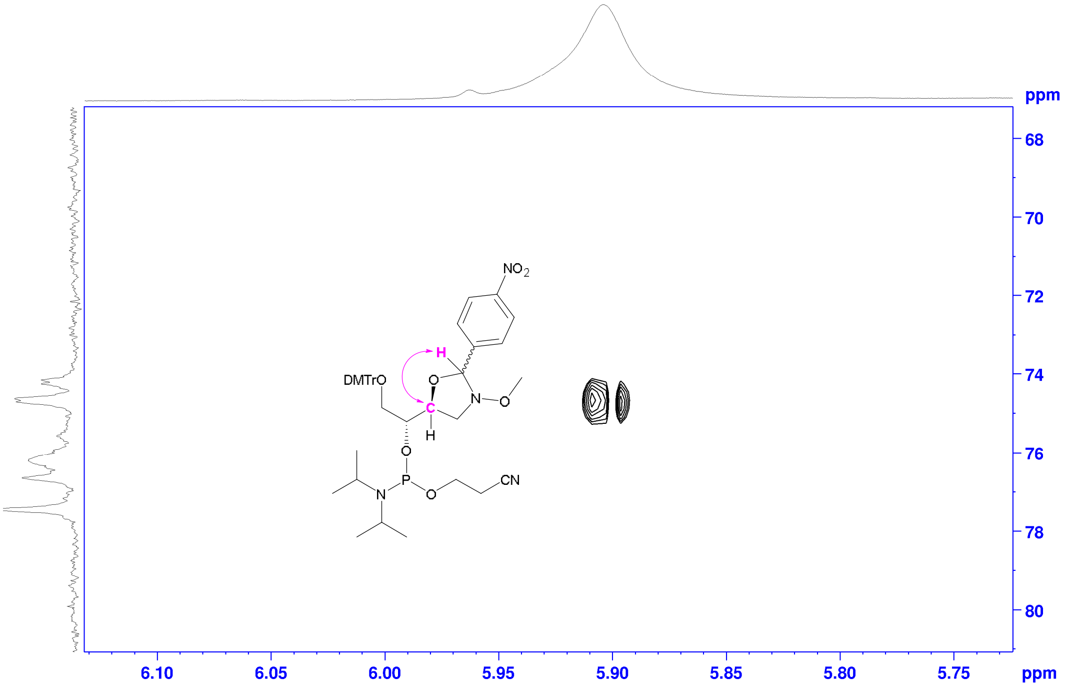

Figure S4. HMBC spectrum of compound 1a (faster-eluting diastereomer, CD_3CN).

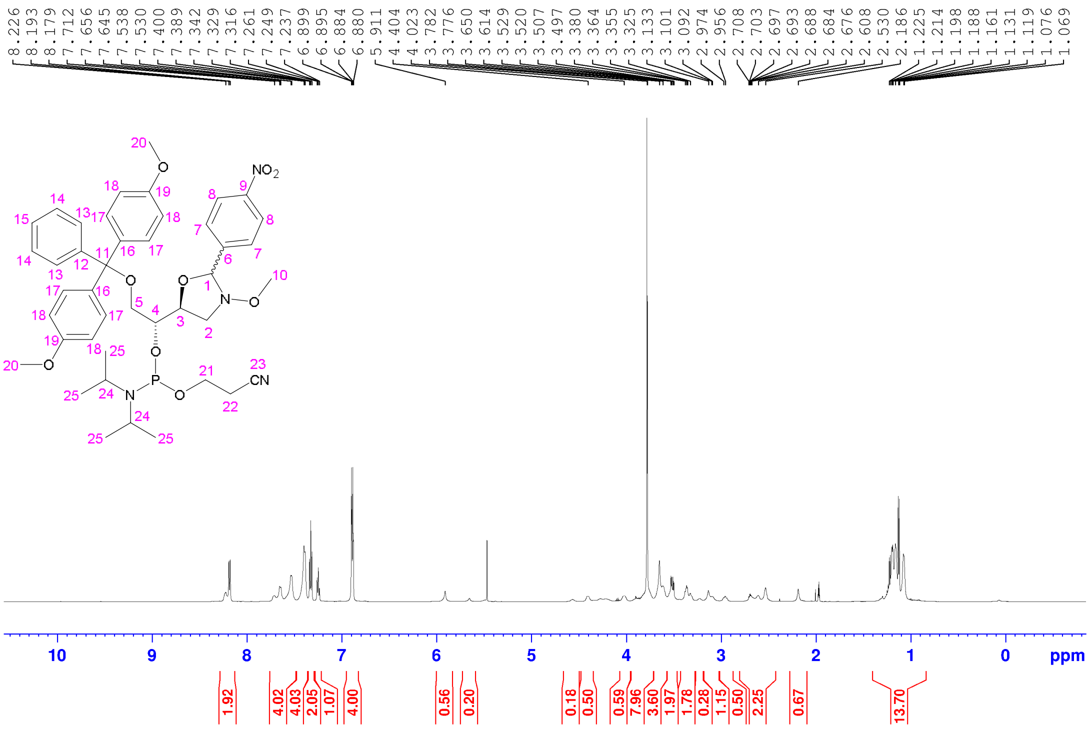

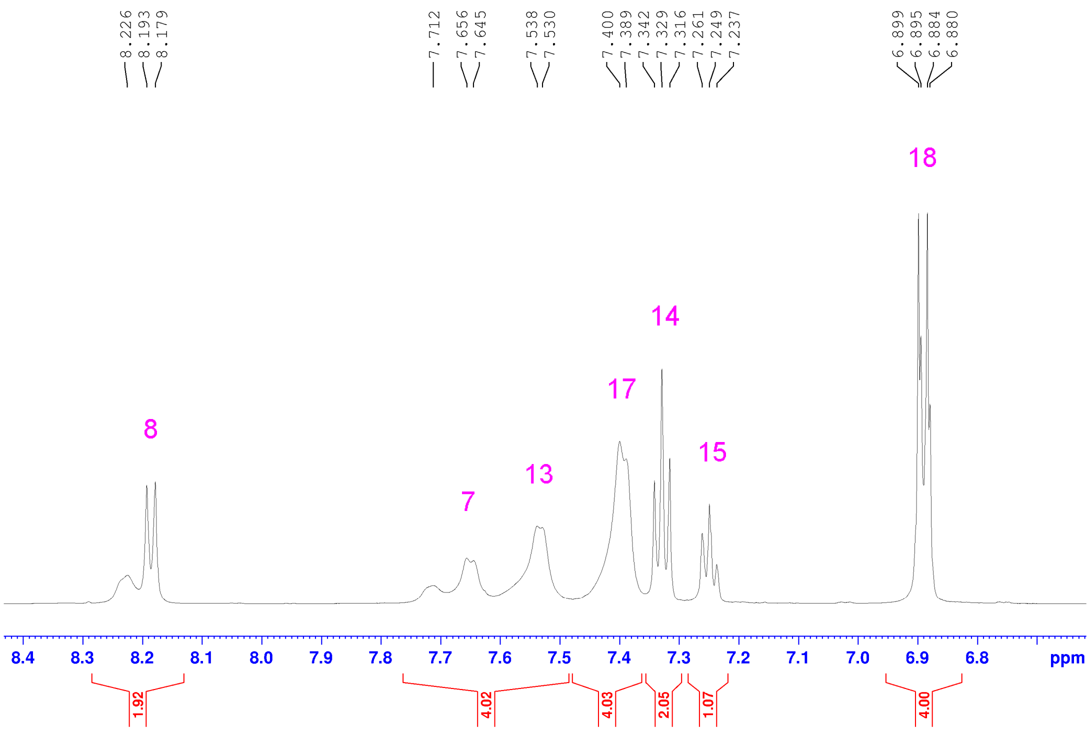

Figure S5. ^{1}H NMR spectrum of compound 1a (slower-eluting diastereomer, 500 MHz, CD_3CN).

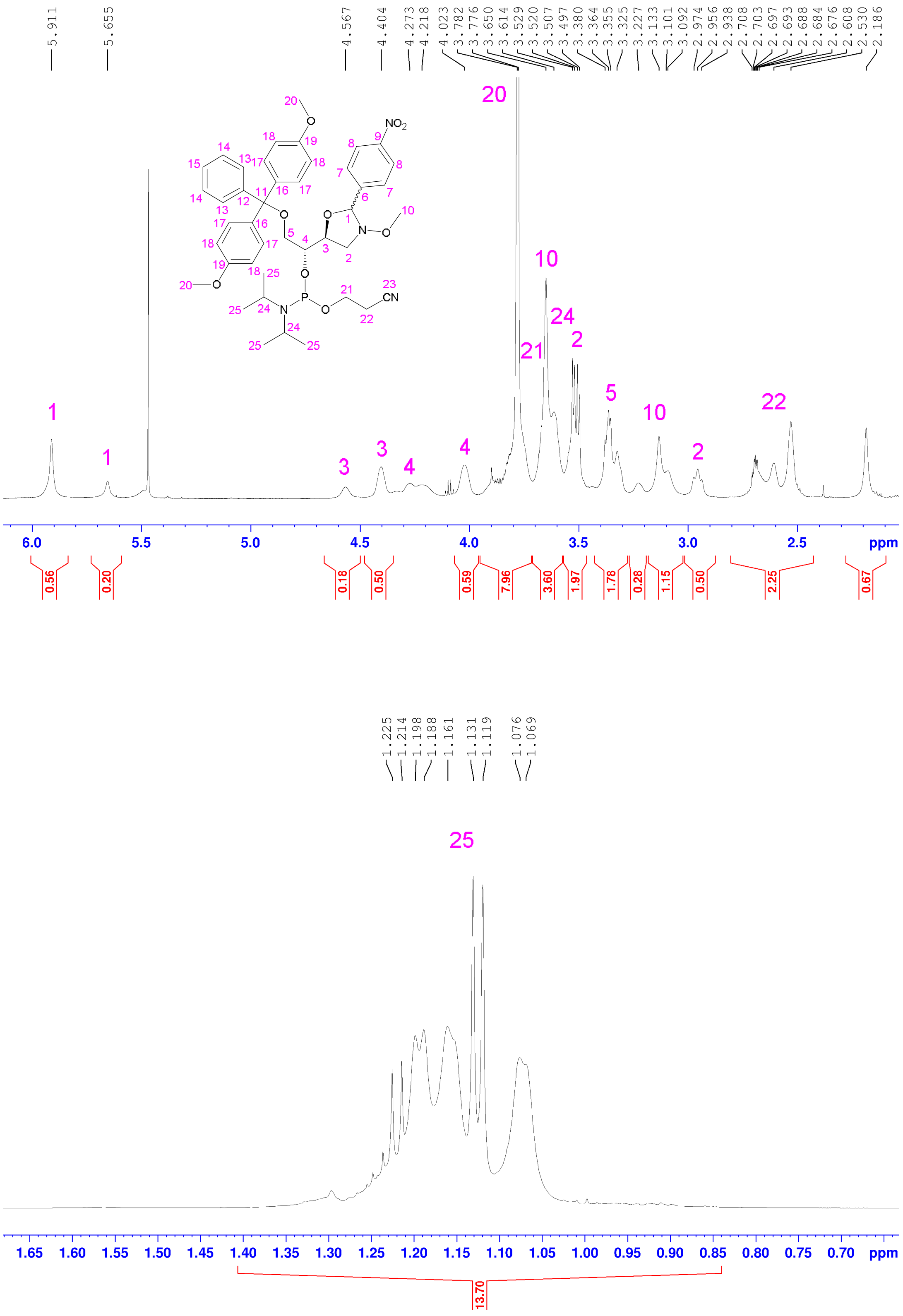

Figure S5 (continued). ^{1}H NMR spectrum of compound 1a (slower-eluting diastereomer, 500 MHz, CD_3CN).

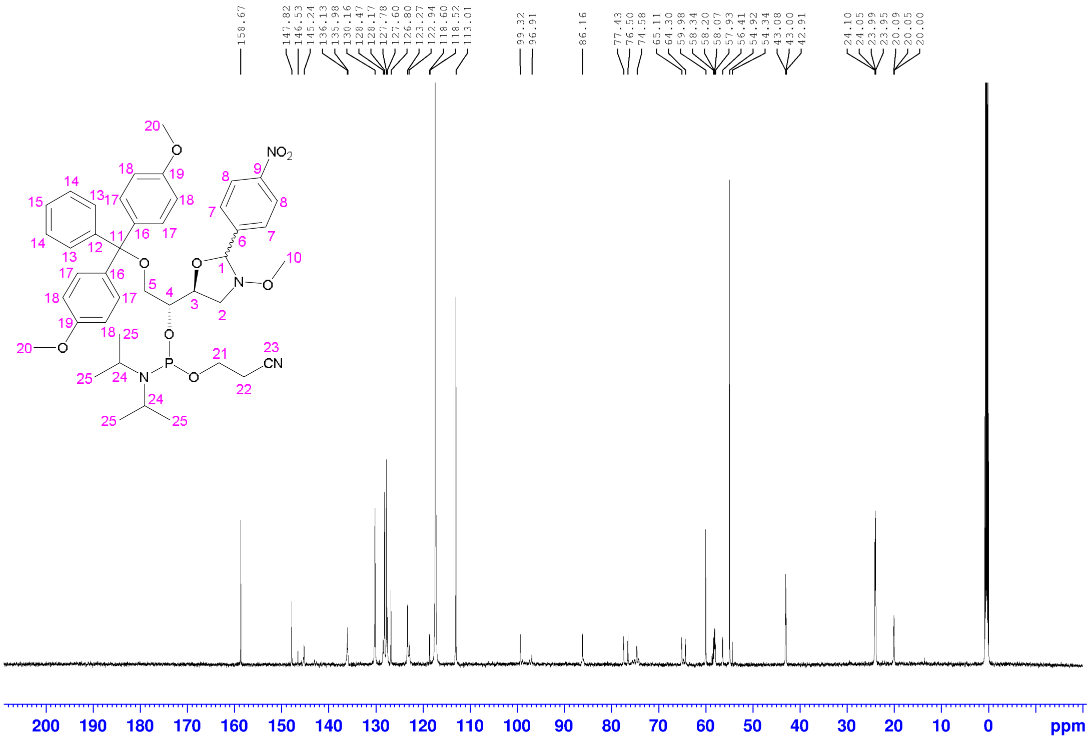

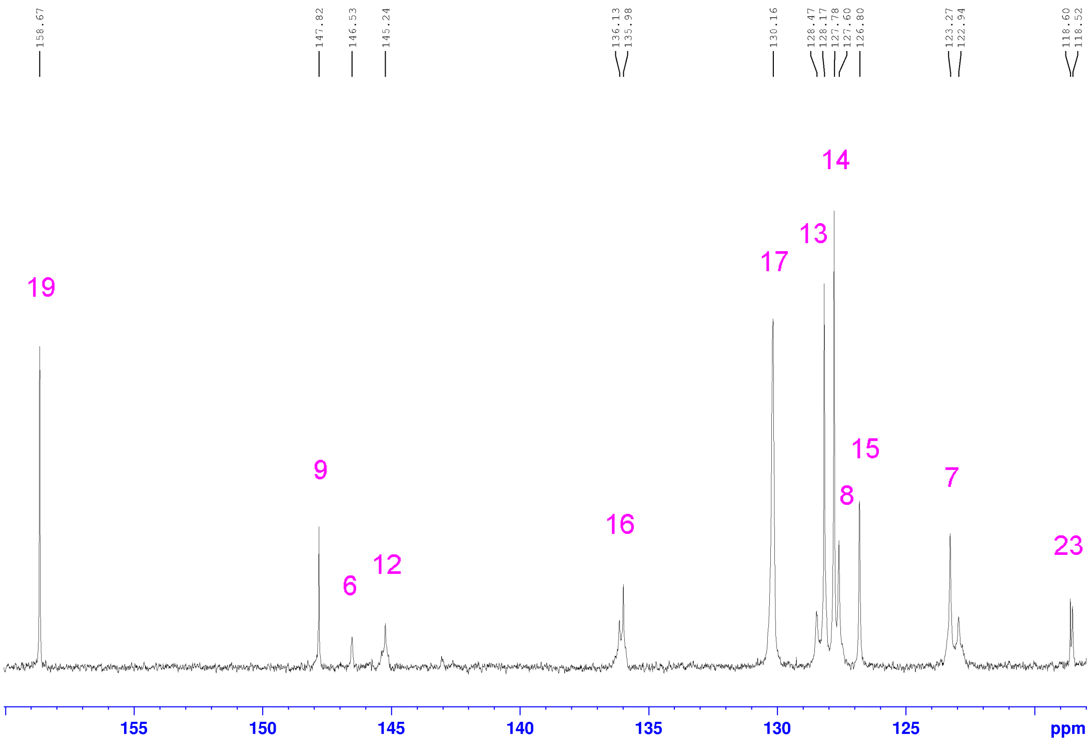

Figure S6. ^{13}C NMR spectrum of compound 1a (slower-eluting diastereomer, 126 MHz, CD_3CN).

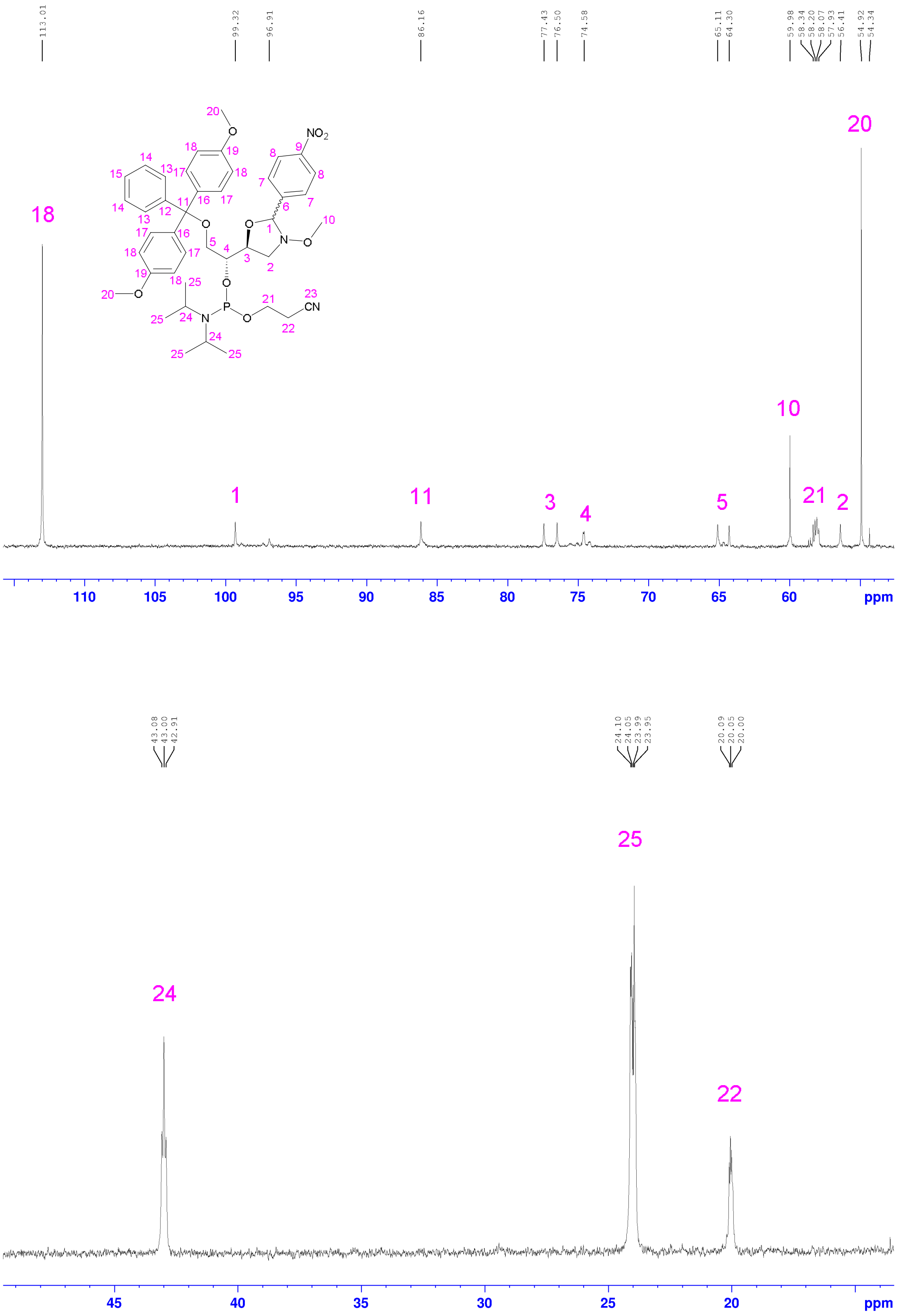

Figure S6 (continued). ^{13}C NMR spectrum of compound 1a (slower-eluting diastereomer, 126 MHz, CD_3CN).

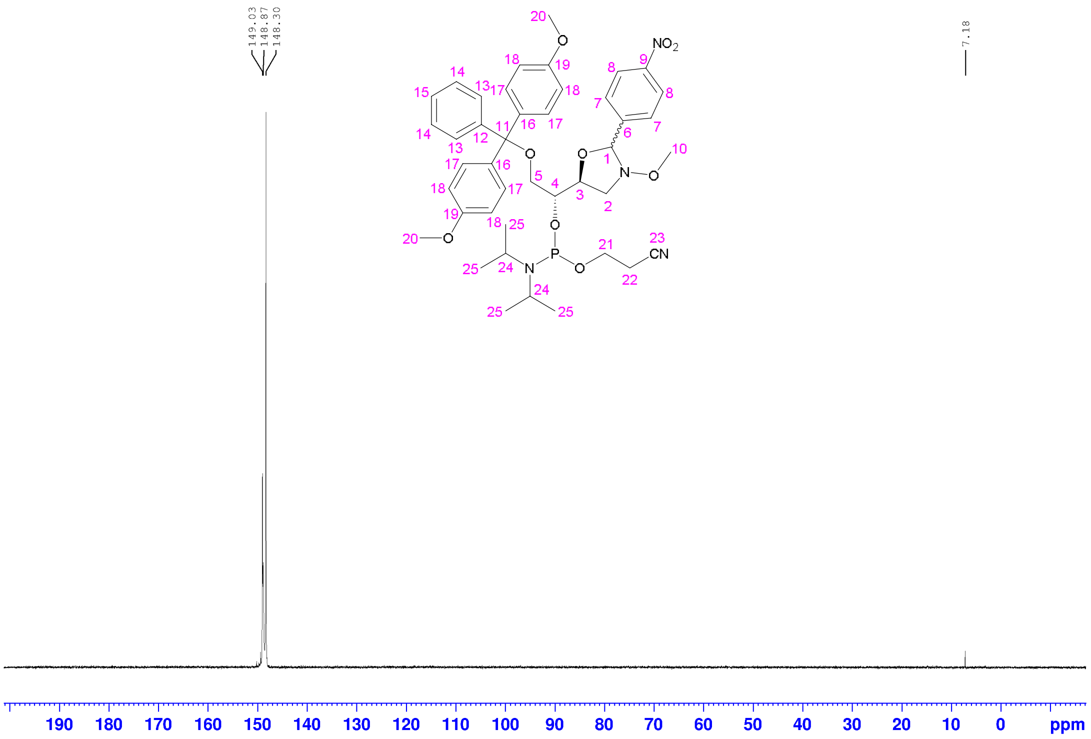

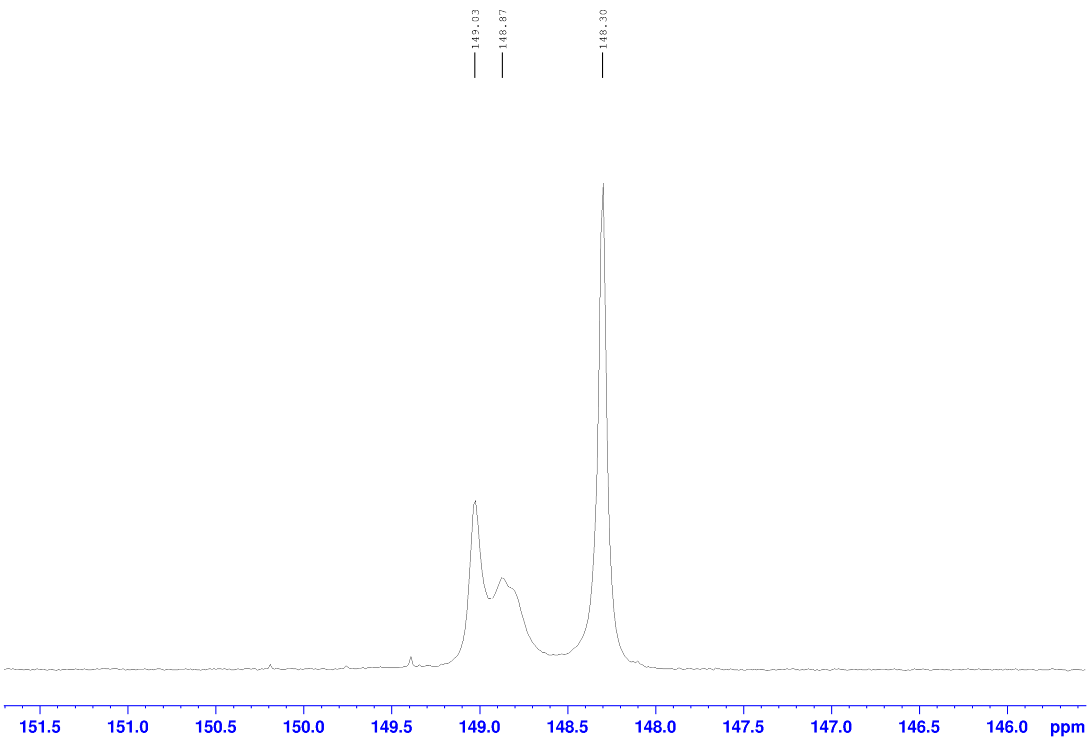

Figure S7. ^{31}P NMR spectrum of compound 1a (slower-eluting diastereomer, 202 MHz, CD_3CN).

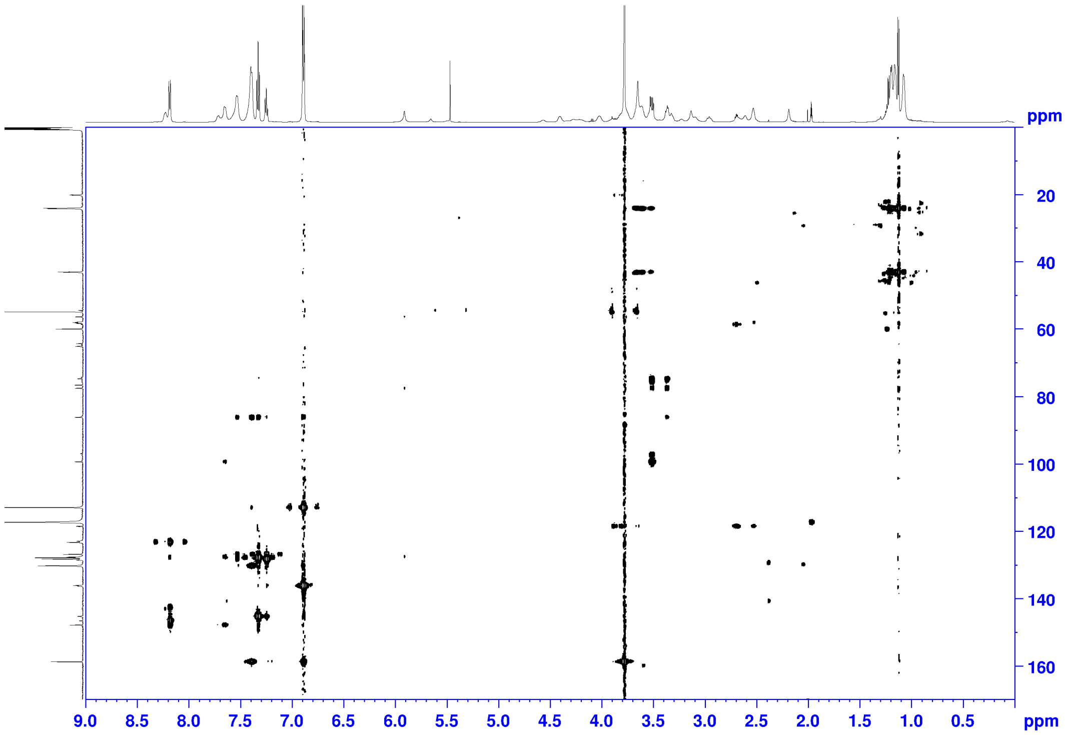

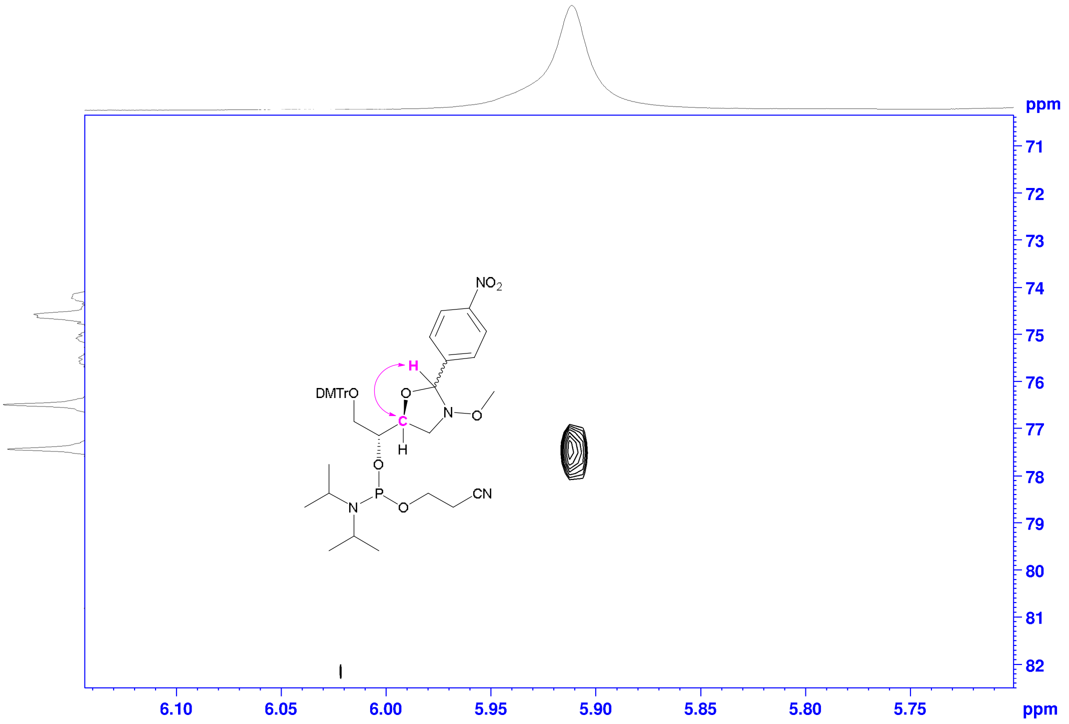

Figure S8. HMBC spectrum of compound 1a (slower-eluting diastereomer, CD_3CN).

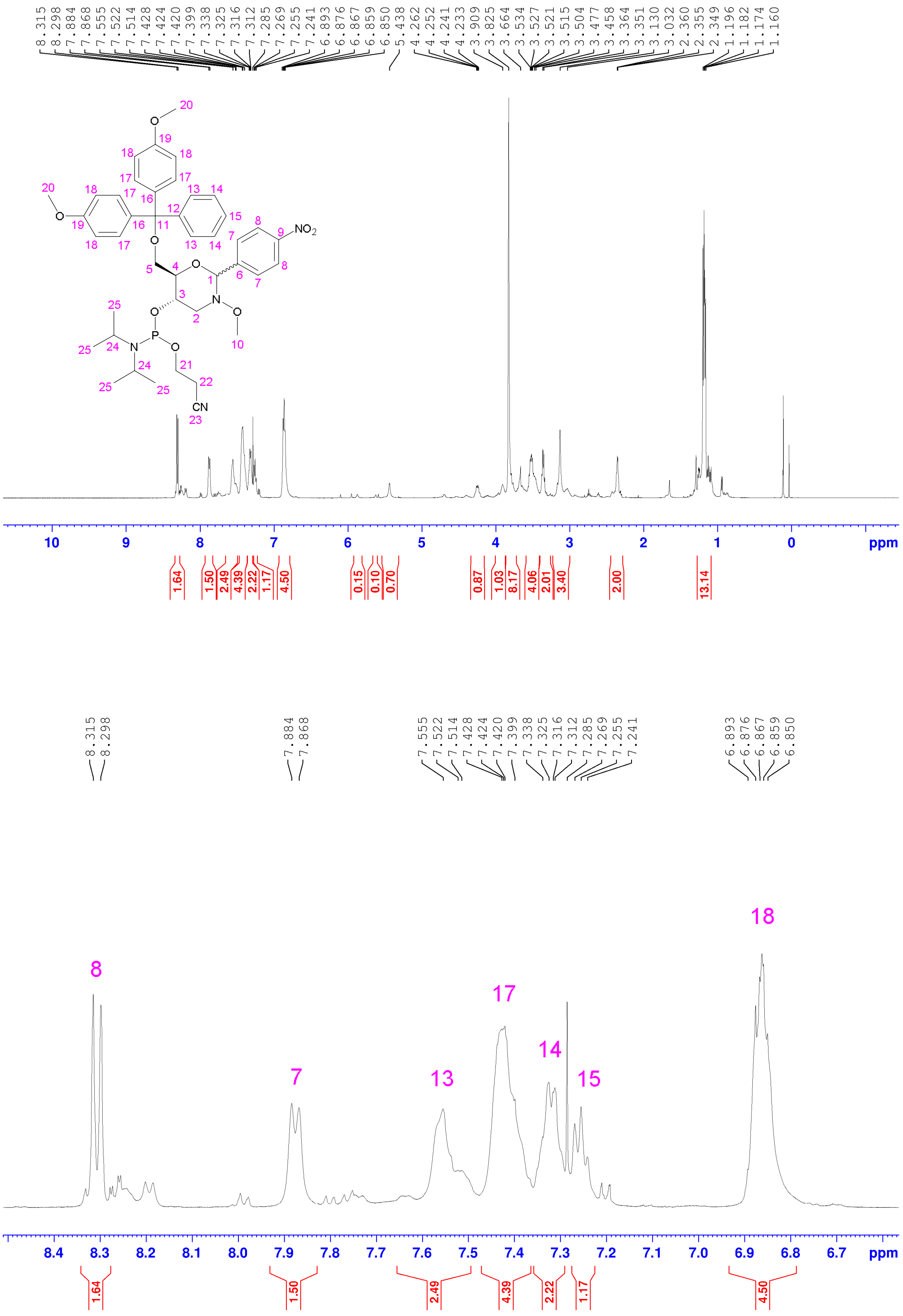

Figure S9. ^{1}H NMR spectrum of compound 1b (faster-eluting diastereomer, 500 MHz, $CDCl_3$).

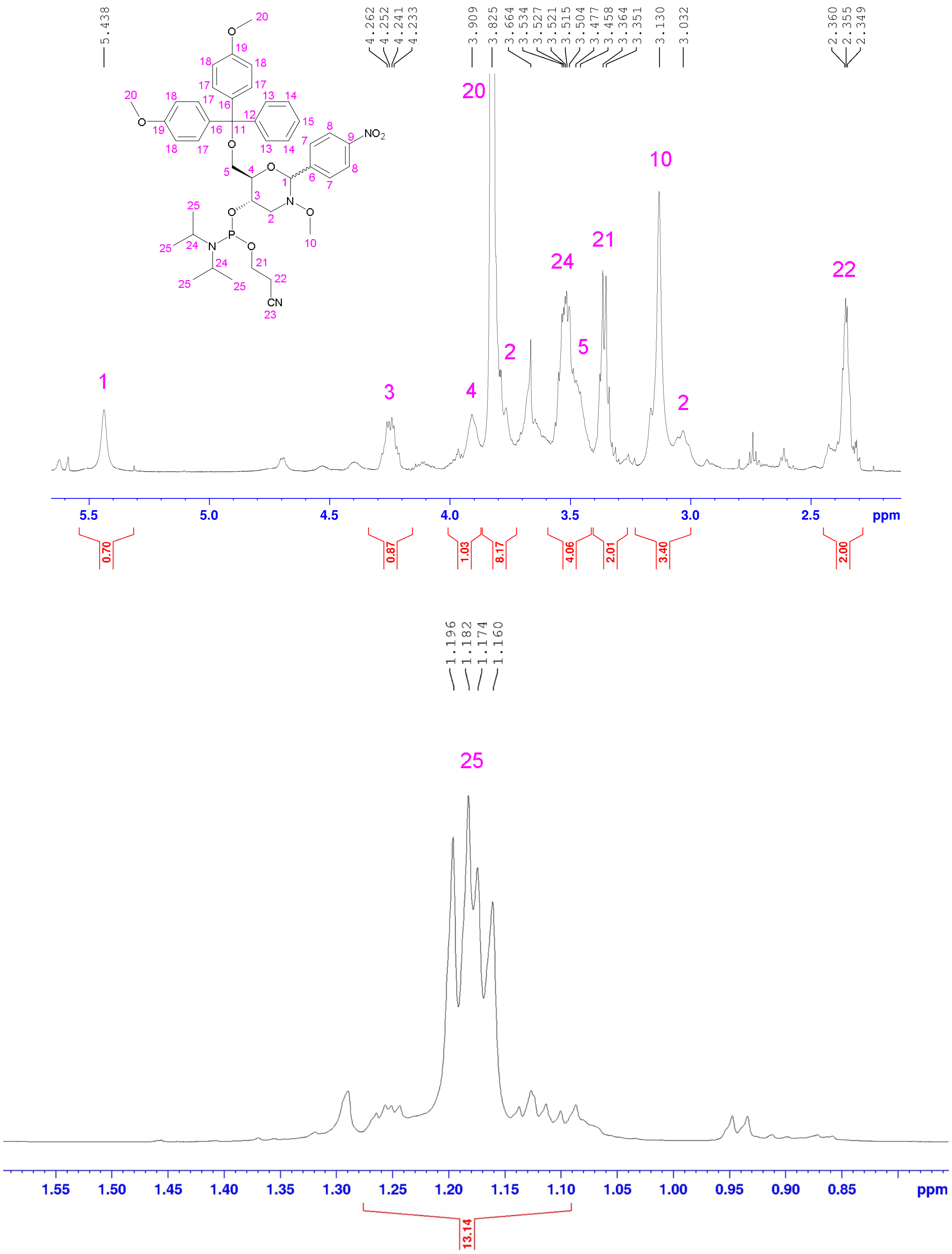

Figure S9 (continued). ^{1}H NMR spectrum of compound 1b (faster-eluting diastereomer, 500 MHz, $CDCl_3$).

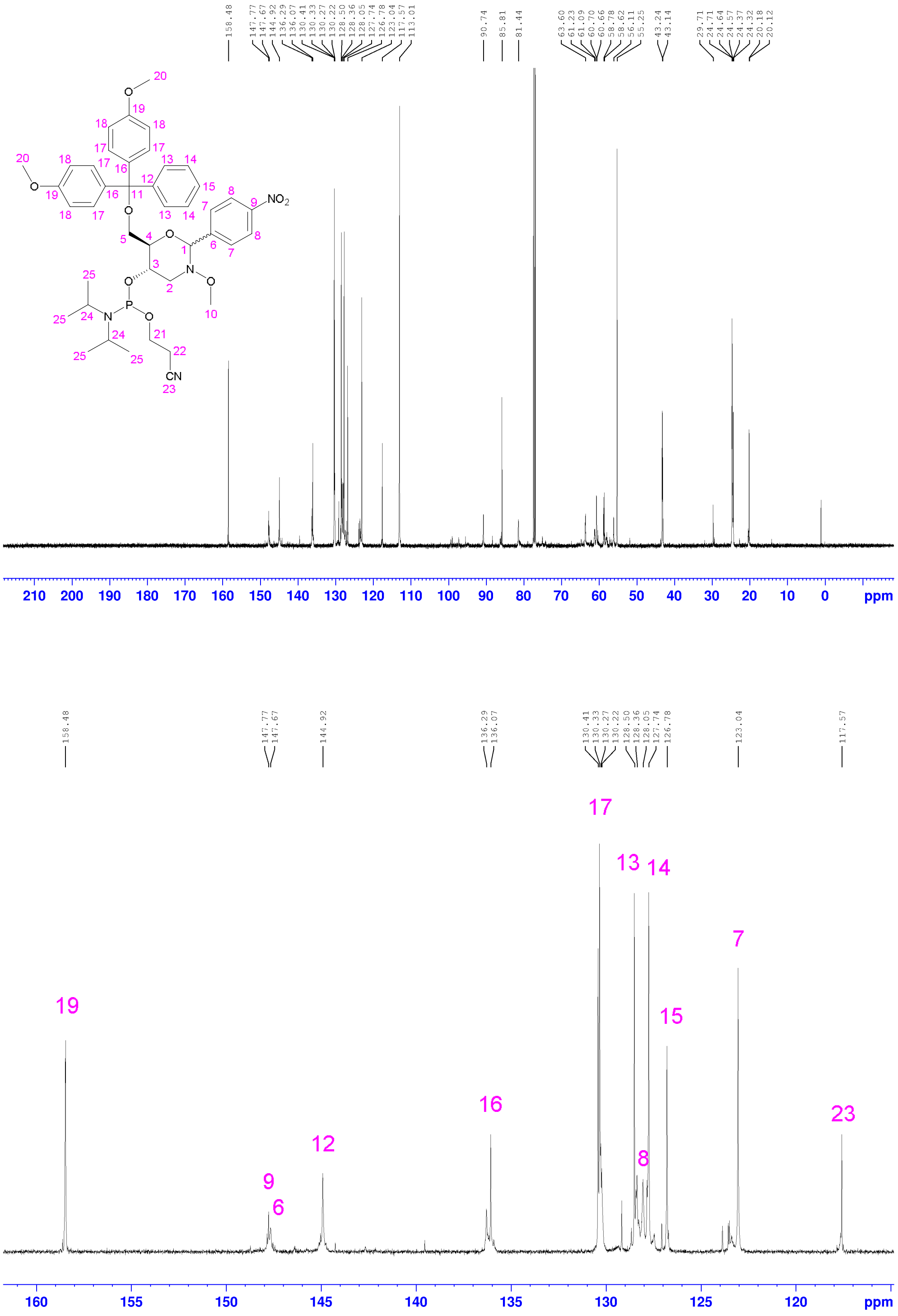

Figure S10. ^{13}C NMR spectrum of compound 1b (faster-eluting diastereomer, 126 MHz, $CDCl_3$).

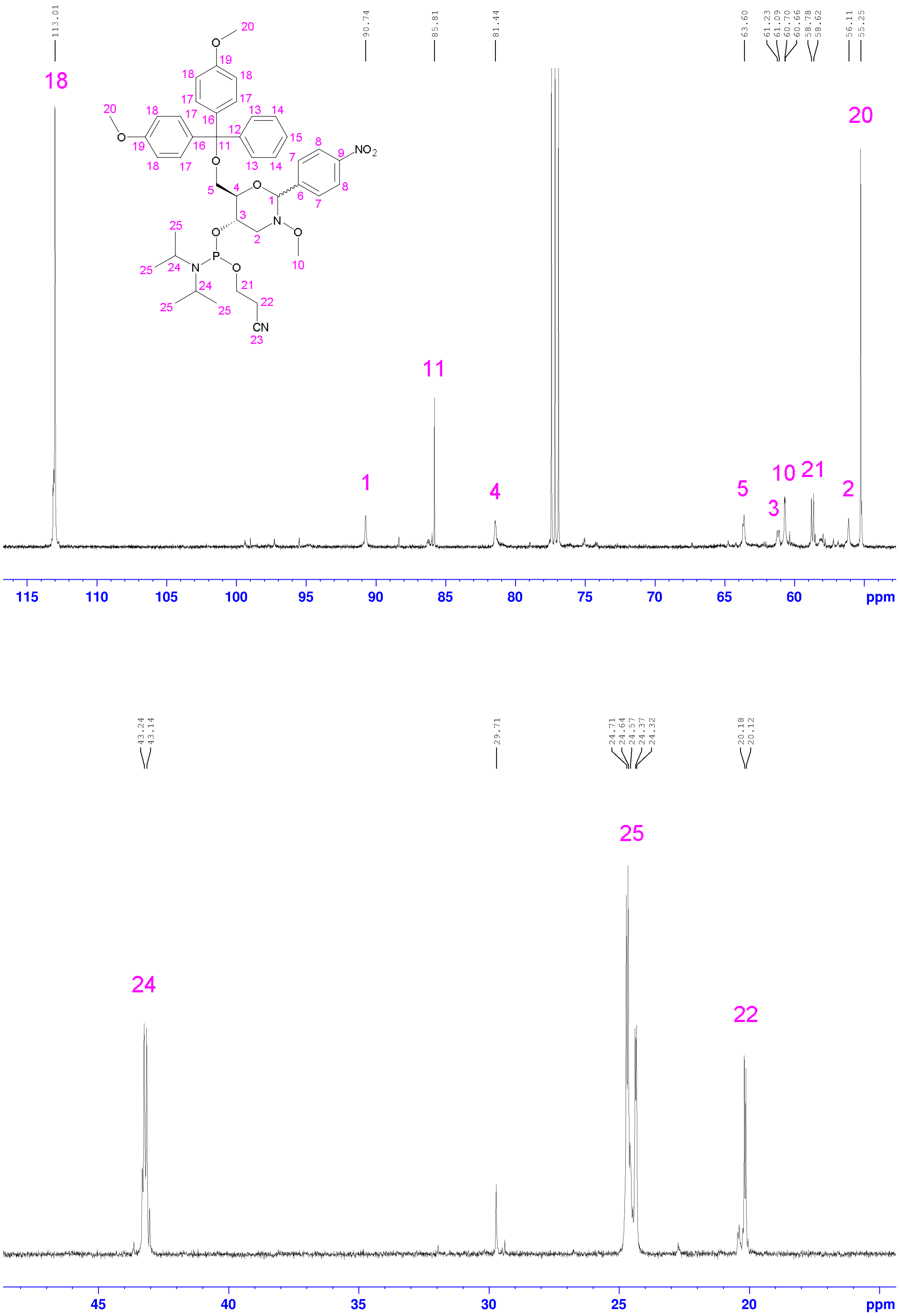

Figure S10 (continued). ^{13}C NMR spectrum of compound 1b (faster-eluting diastereomer, 126 MHz, $CDCl_3$).

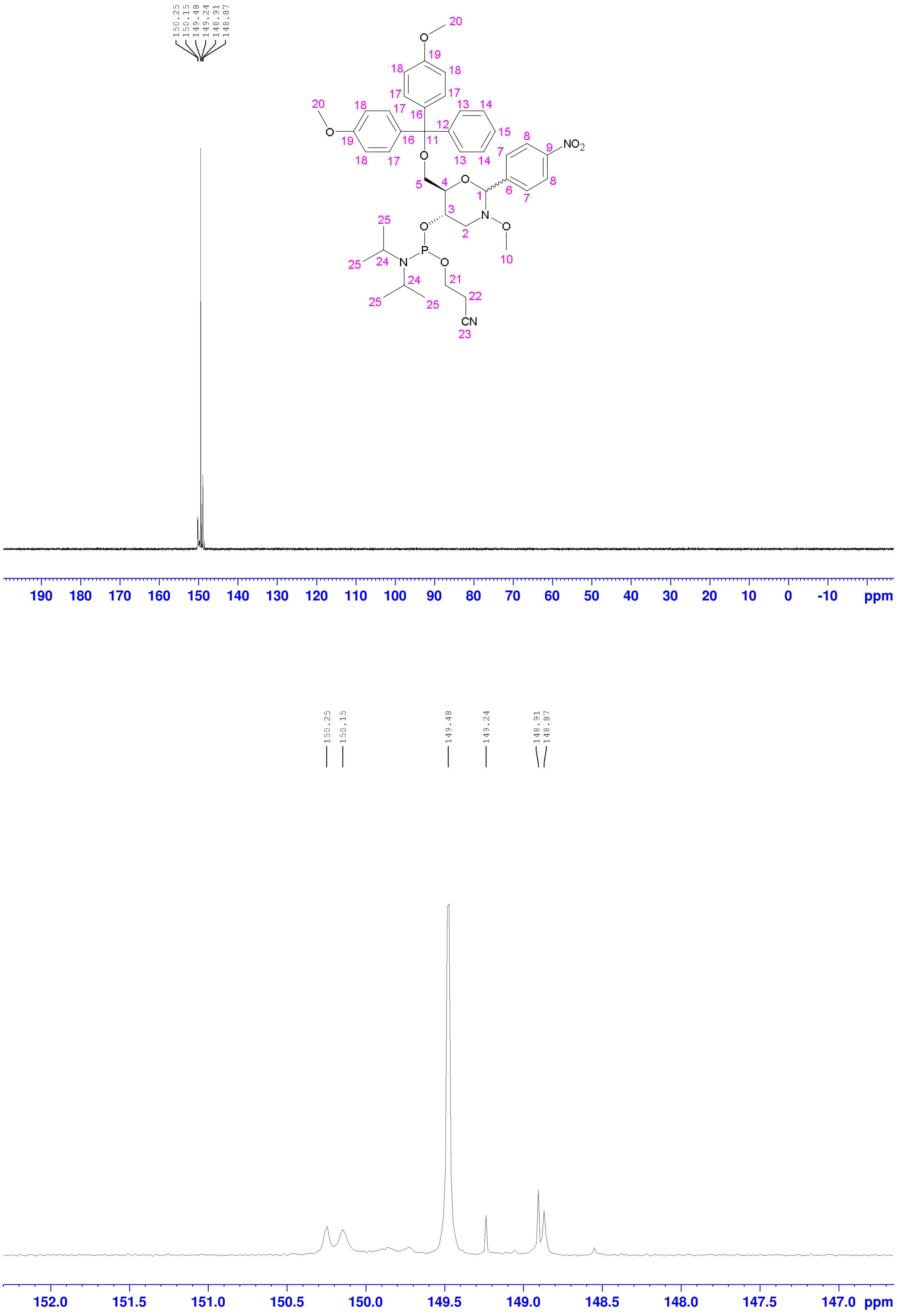

Figure S11. ^{31}P NMR spectrum of compound 1b (faster-eluting diastereomer, 202 MHz, $CDCl_3$).

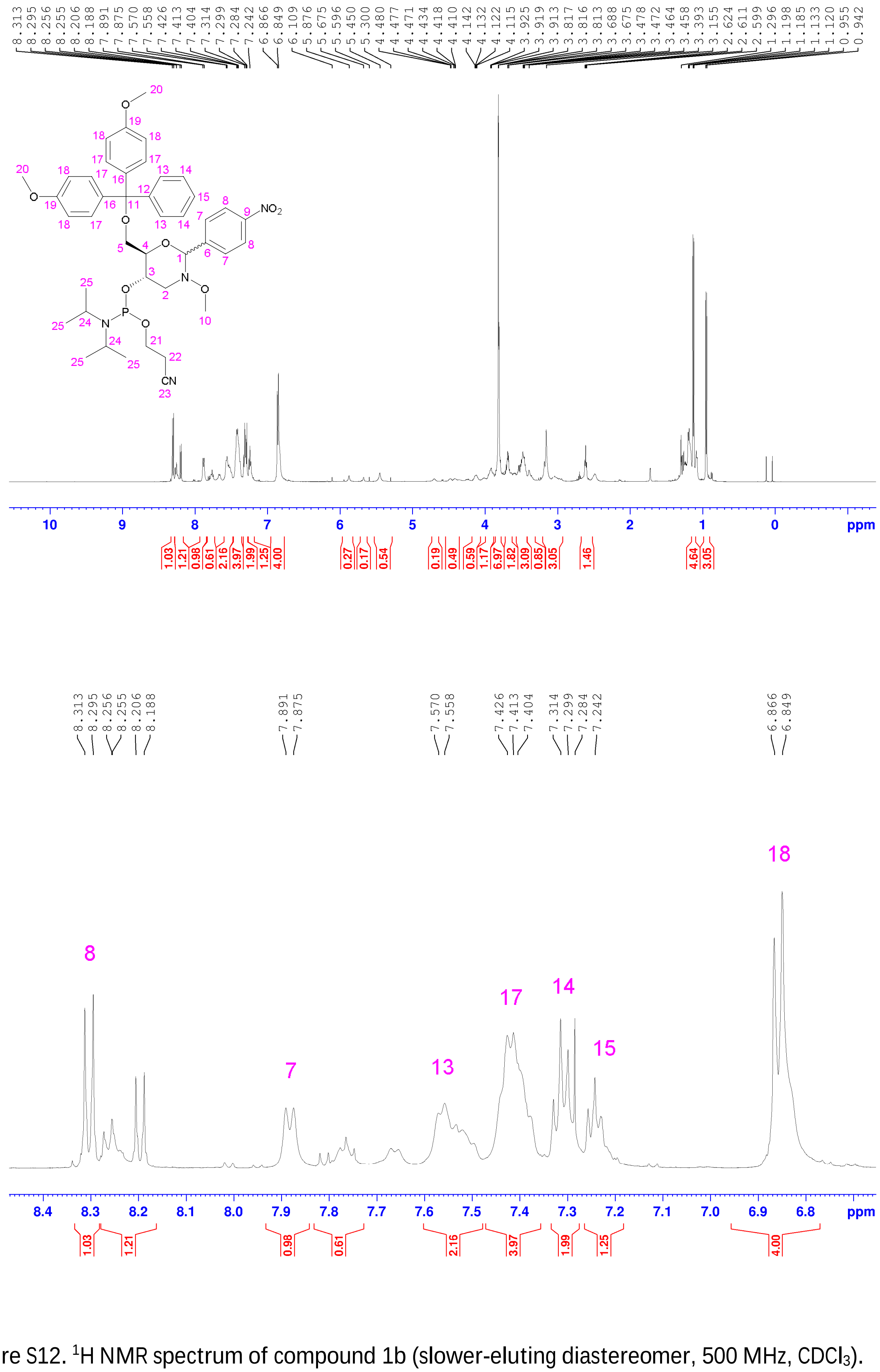

Figure S12. ^{1}H NMR spectrum of compound 1b (slower-eluting diastereomer, 500 MHz, $CDCl_3$).

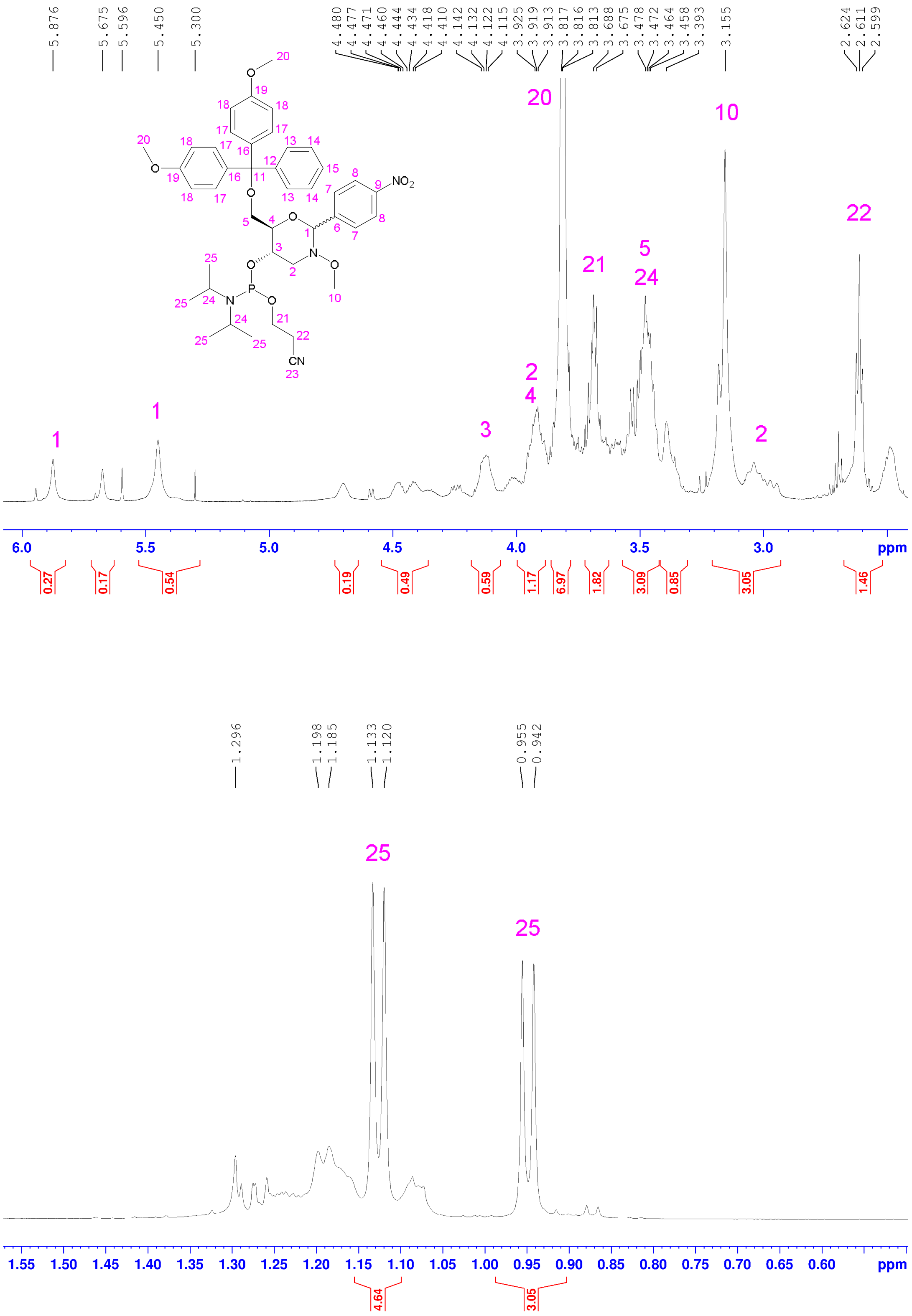

Figure S12 (continued). ^1H NMR spectrum of compound 1b (slower-eluting diastereomer, 500 MHz, $CDCl_3$).

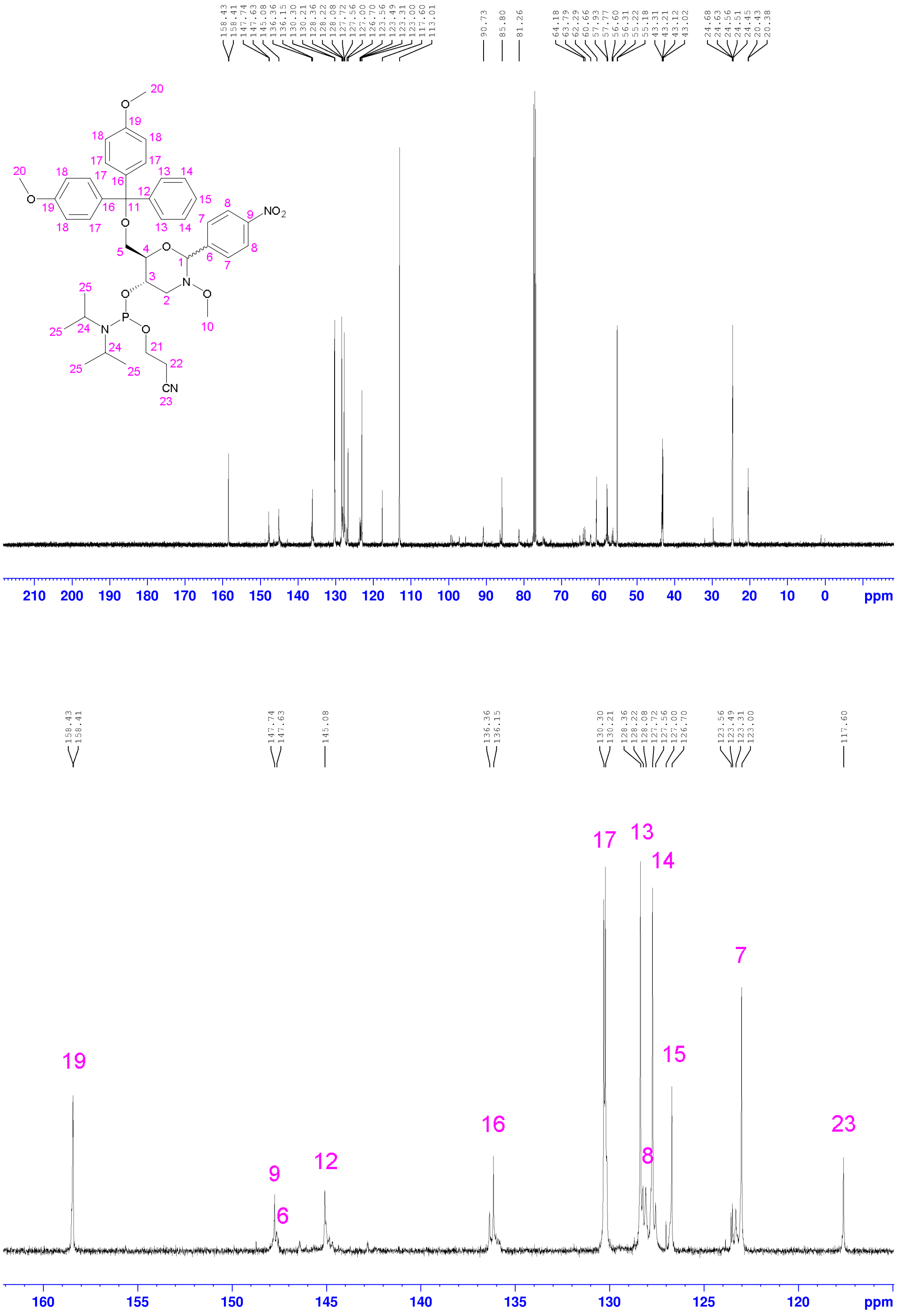

Figure S13. ^{13}C NMR spectrum of compound 1b (slower-eluting diastereomer, 126 MHz, $CDCl_3$).

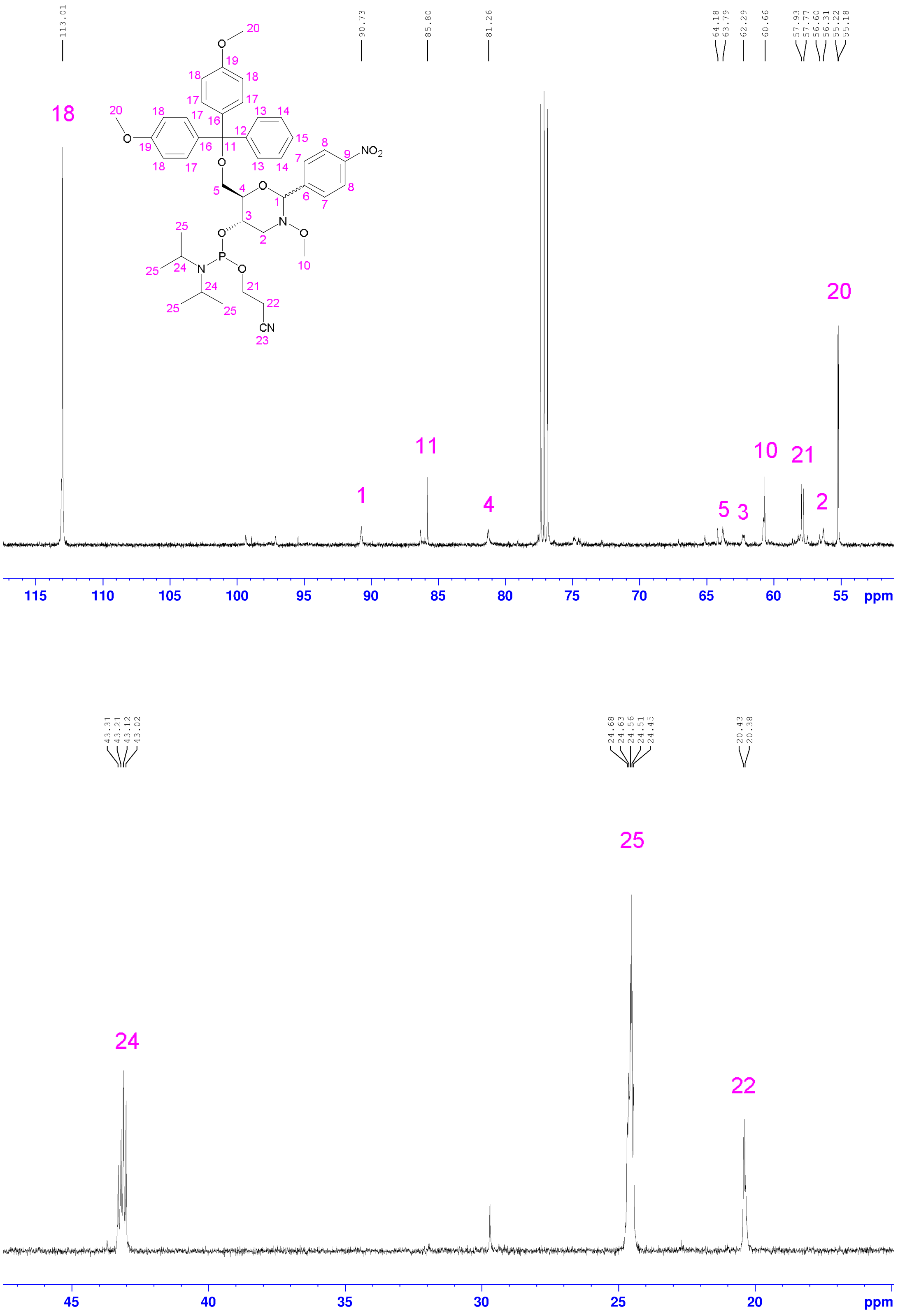

Figure S13 (continued). ^{13}C NMR spectrum of compound 1b (slower-eluting diastereomer, 126 MHz, $CDCl_3$).

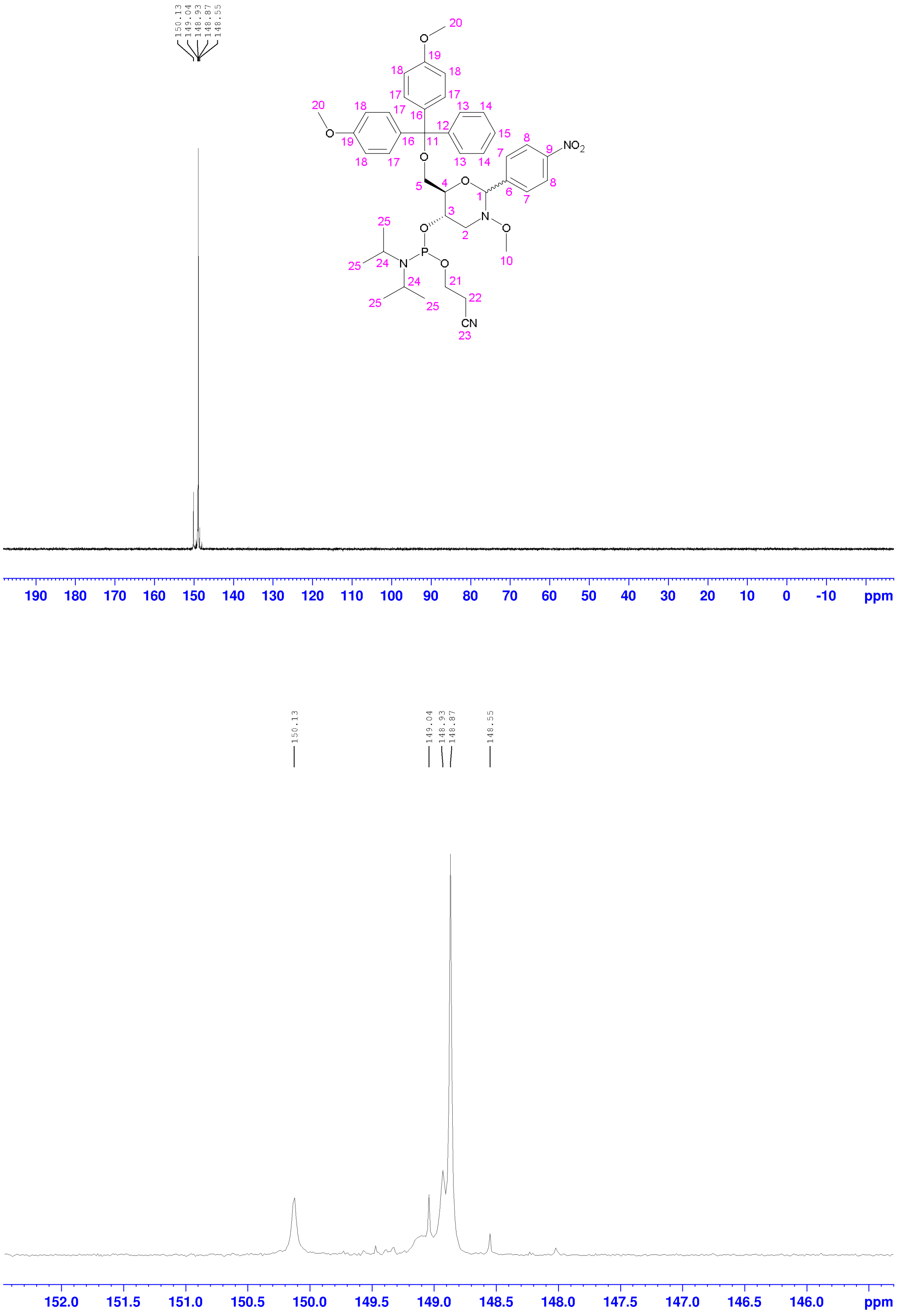

Figure S14. ^{31}P NMR spectrum of compound 1b (slower-eluting diastereomer, 202 MHz, $CDCl_3$).

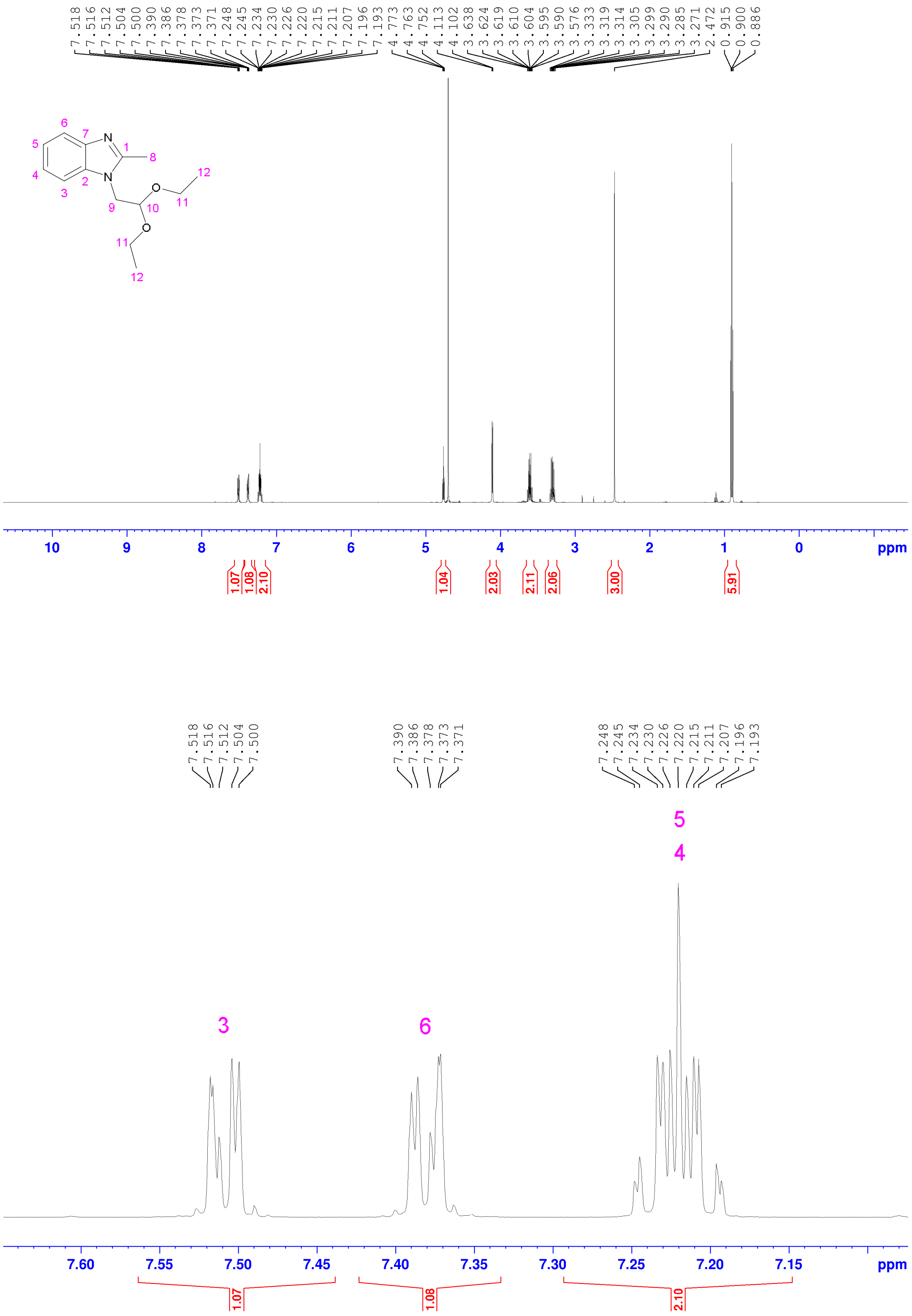

Figure S15. ^{1}H NMR spectrum of compound 6 (600 MHz, D_2O).

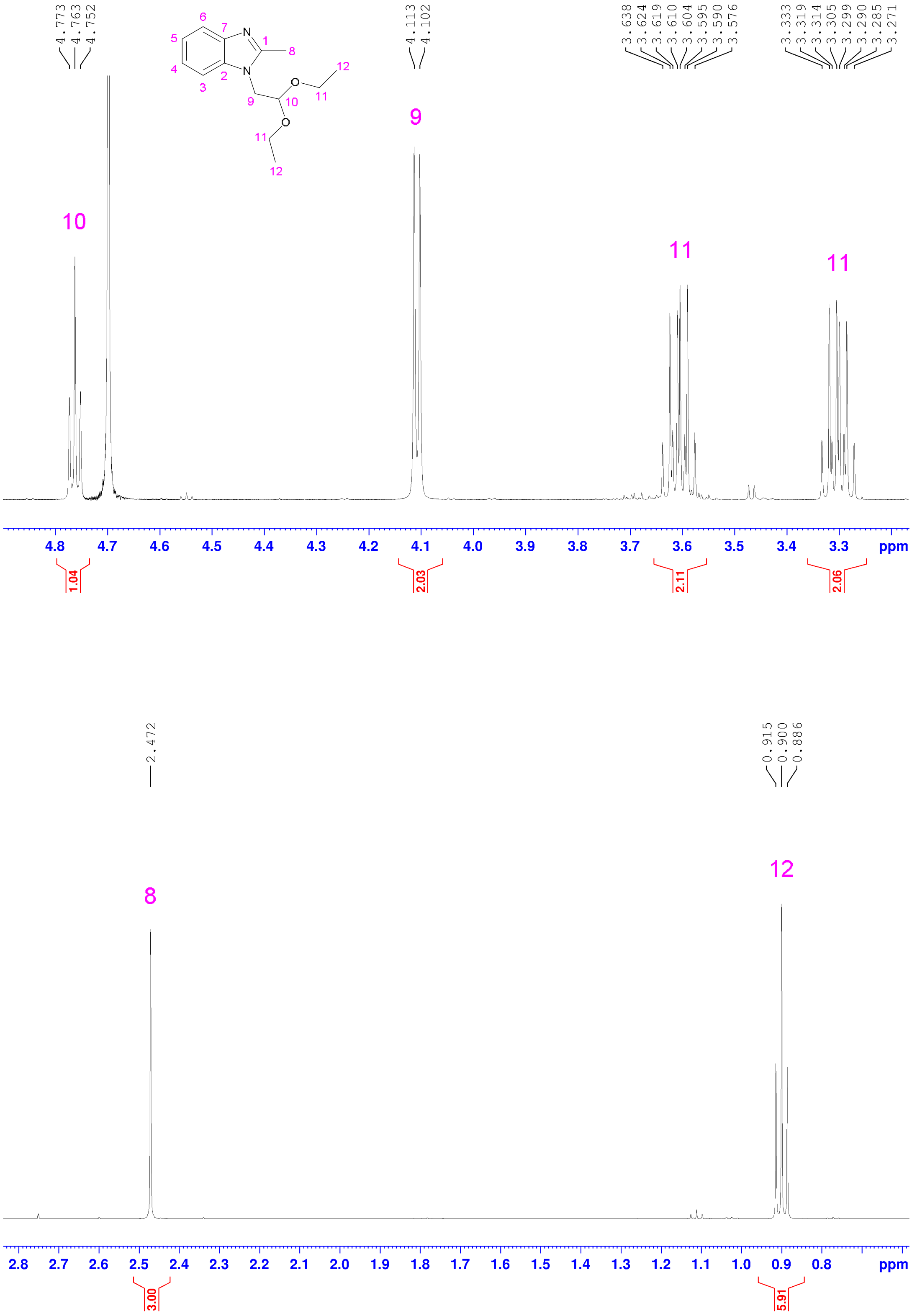

Figure S15 (continued). ^{1}H NMR spectrum of compound 6 (600 MHz, D_2O).

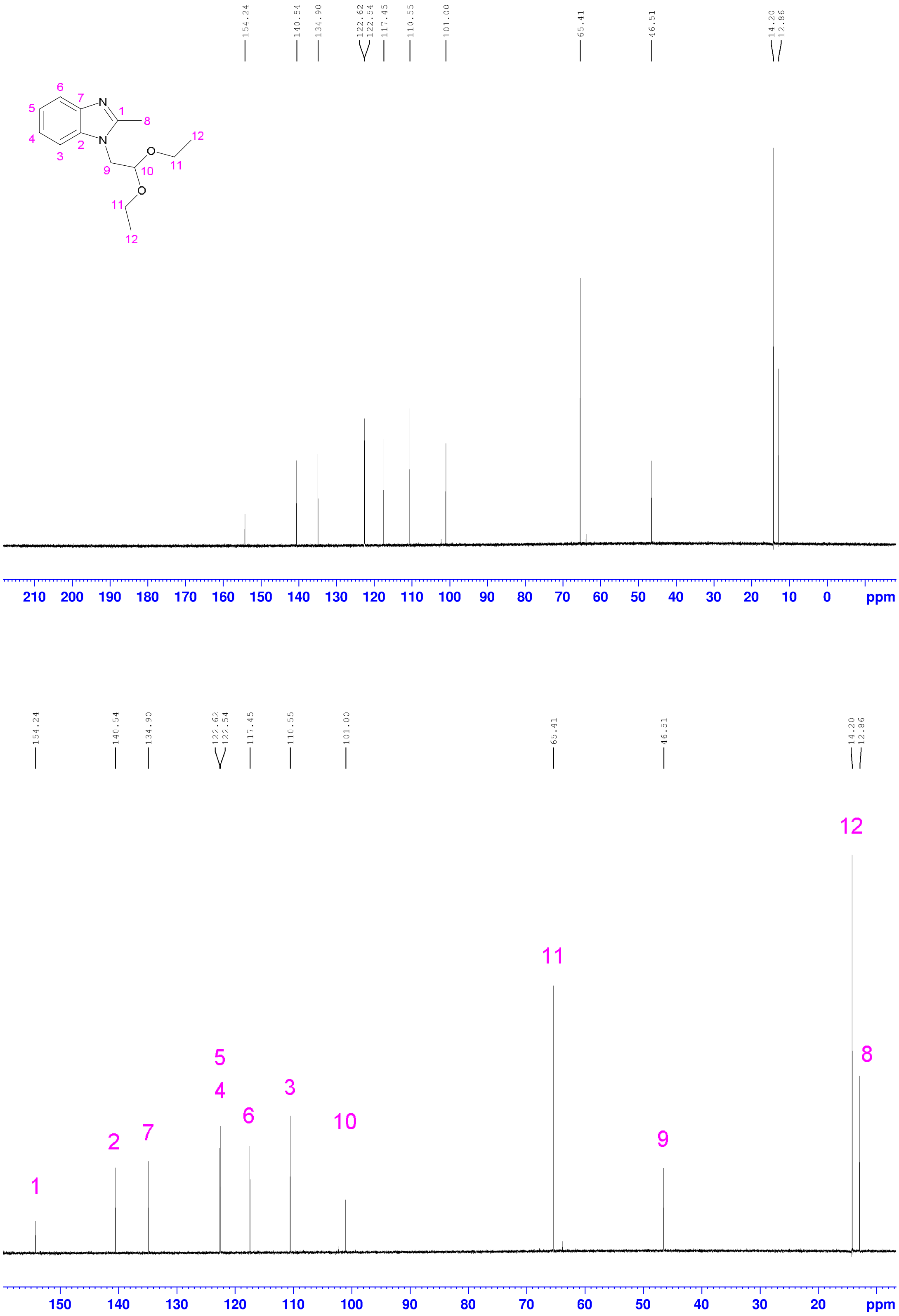

Figure S16. ^{13}C NMR spectrum of compound 6 (150 MHz, D_2O).

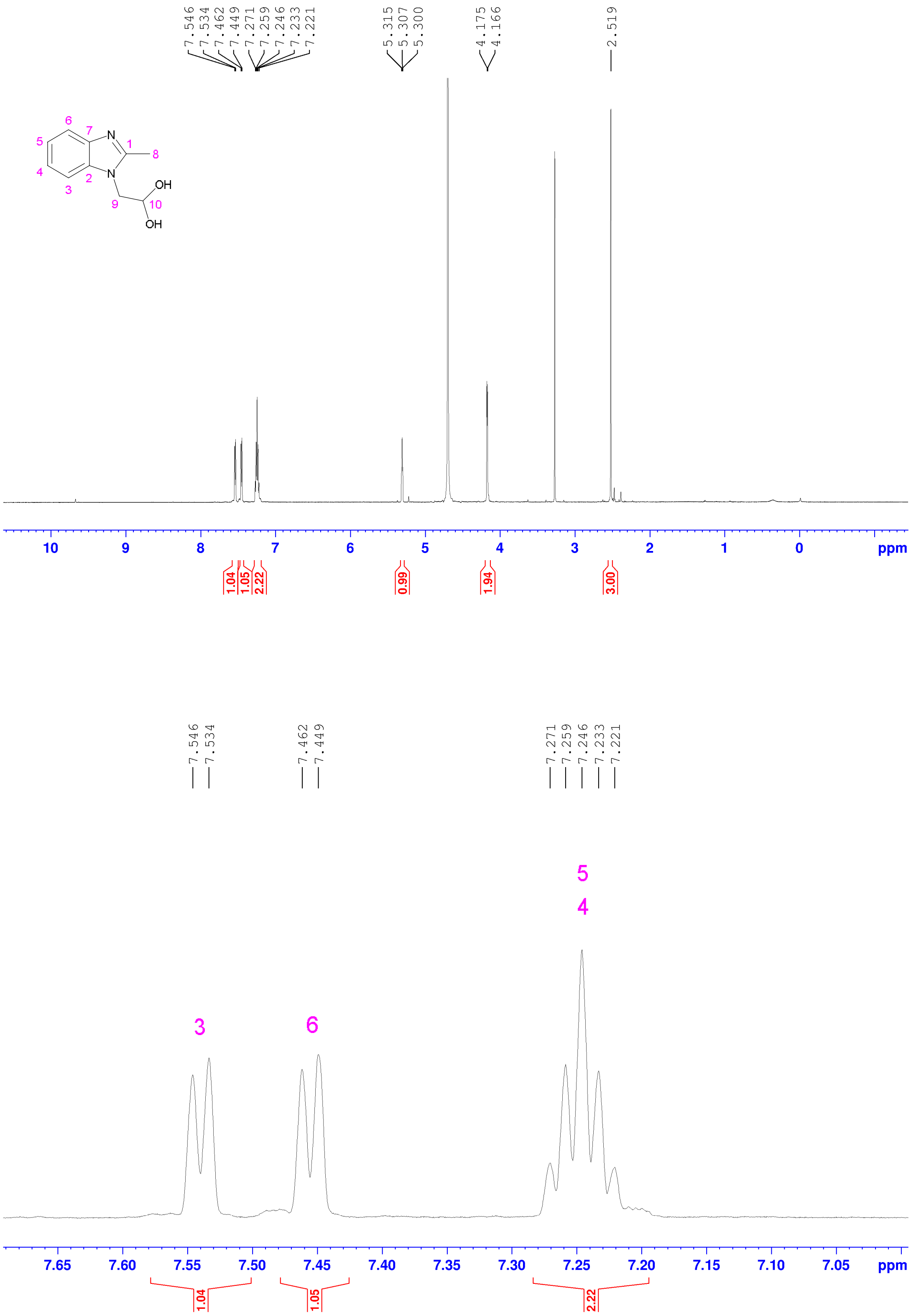

Figure S17. ^{1}H NMR spectrum of compound fmB (600 MHz, D_2O).

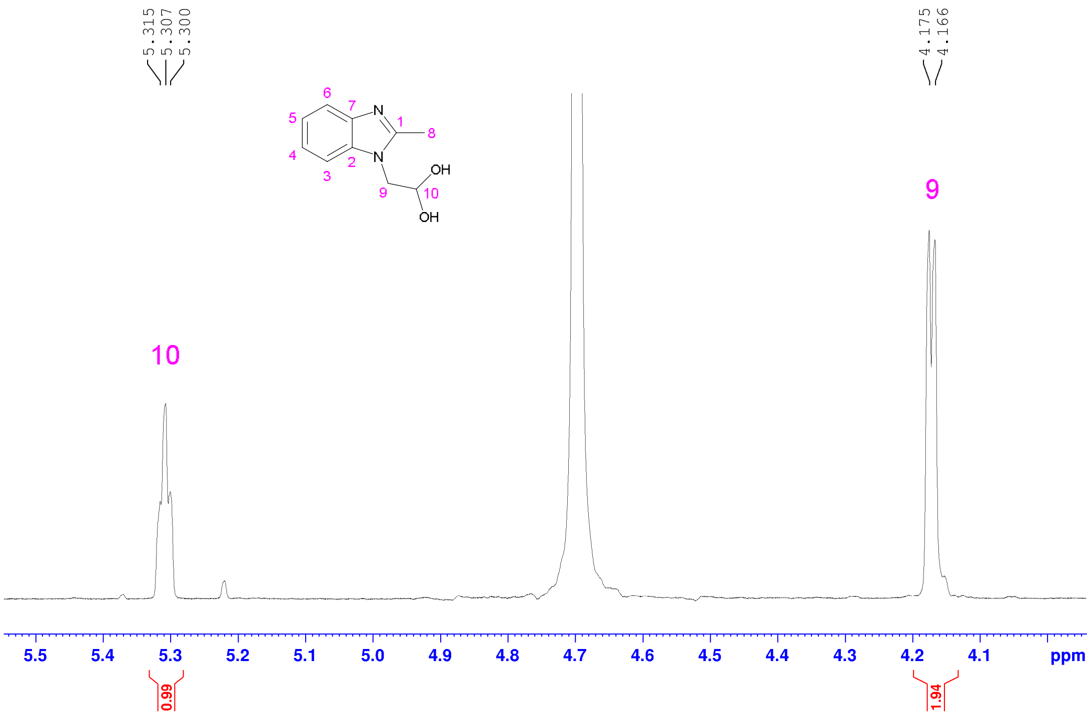

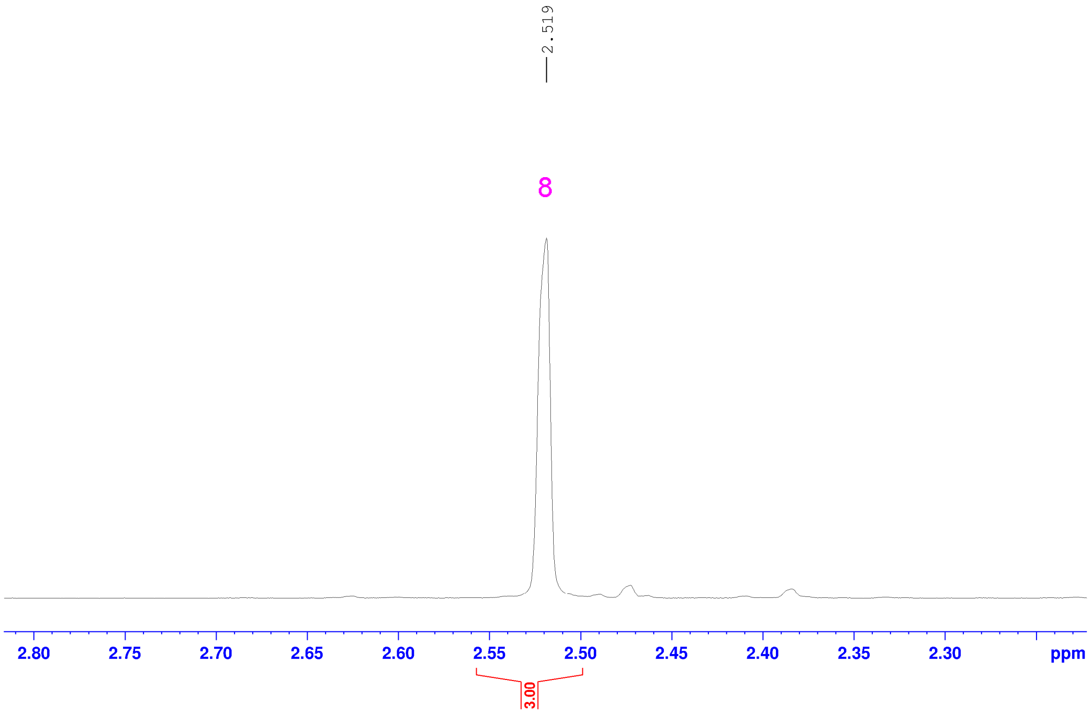

Figure S17 (continued). ^{1}H NMR spectrum of compound fmB (600 MHz, D_2O).

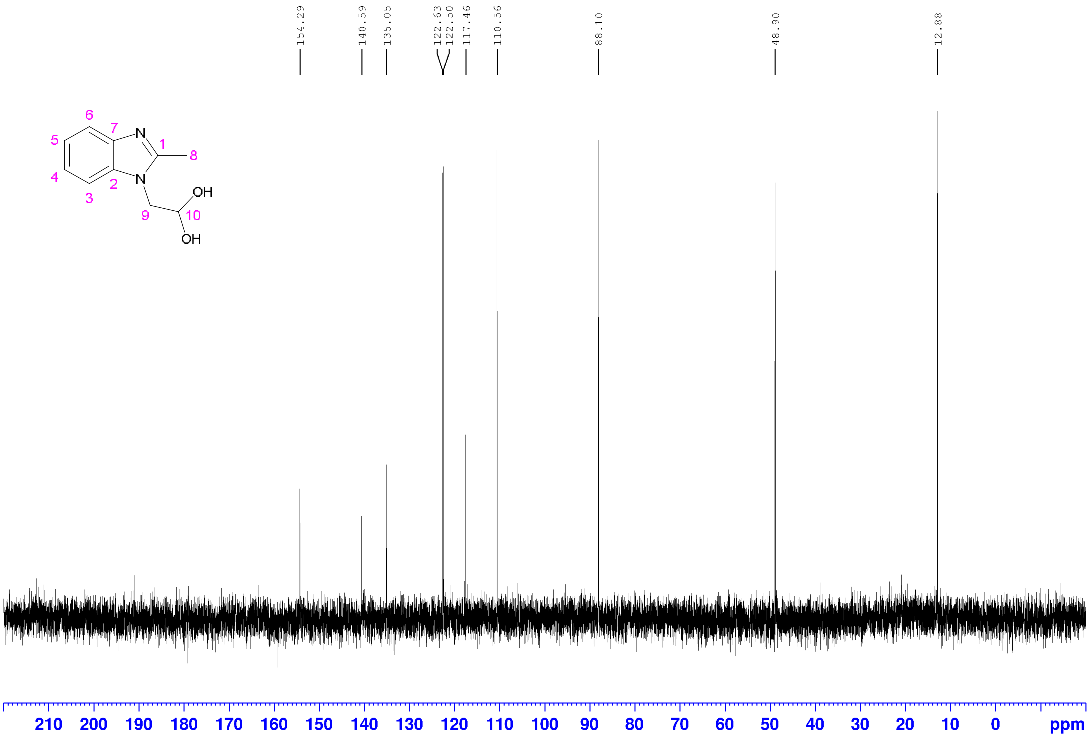

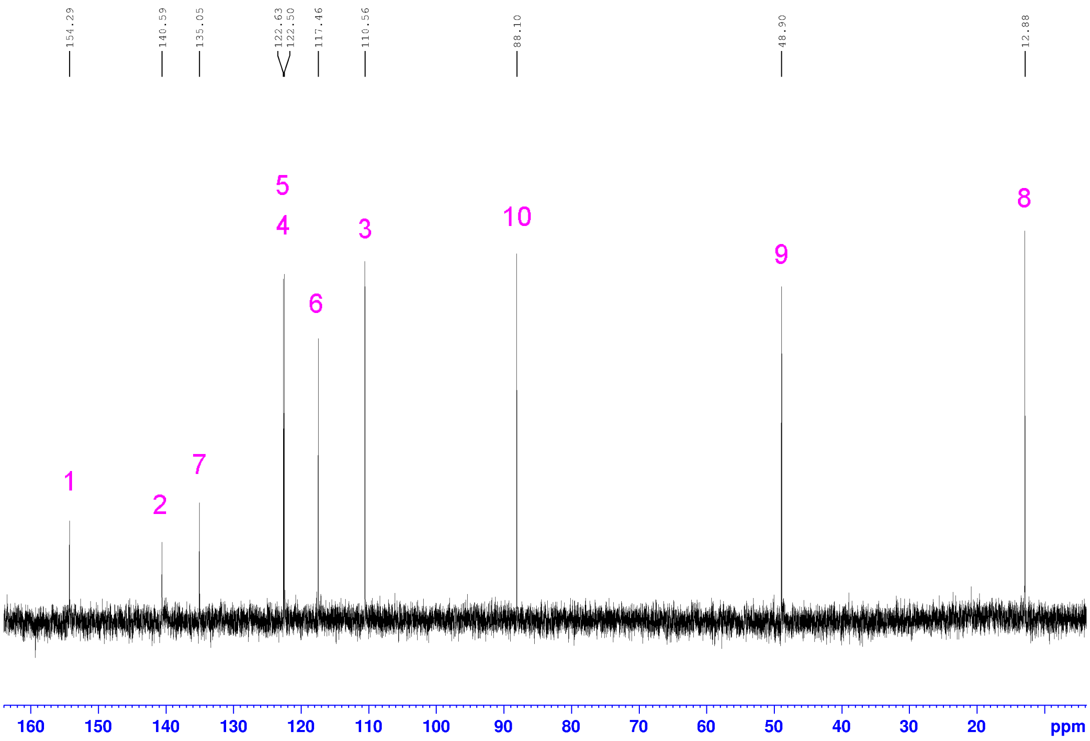

Figure S18. ^{13}C NMR spectrum of compound fmB (150 MHz, D_2O).

A **B**

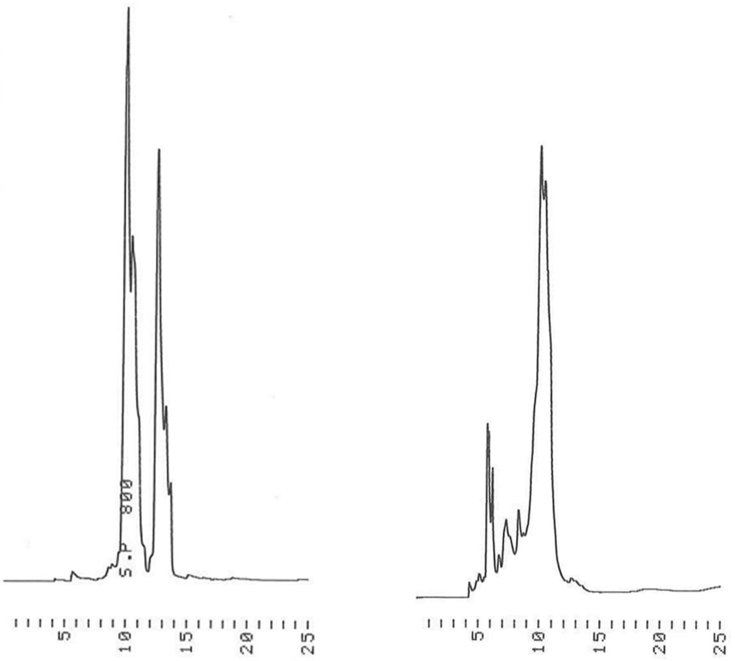

Figure S19. RP-HPLC traces of A) crude product mixture of the synthesis of oligonucleotide ON3 and B) the purified product after treatment with acetic acid; Thermo Scientific ODS Hypersil column (250 × 10 mm, 5 μM); flow rate = 3.0 mL min^{-1}; linear gradient (10—40 % over 25 min) of MeCN in 50 mM aqueous triethylammonium acetate; λ = 260 nm.

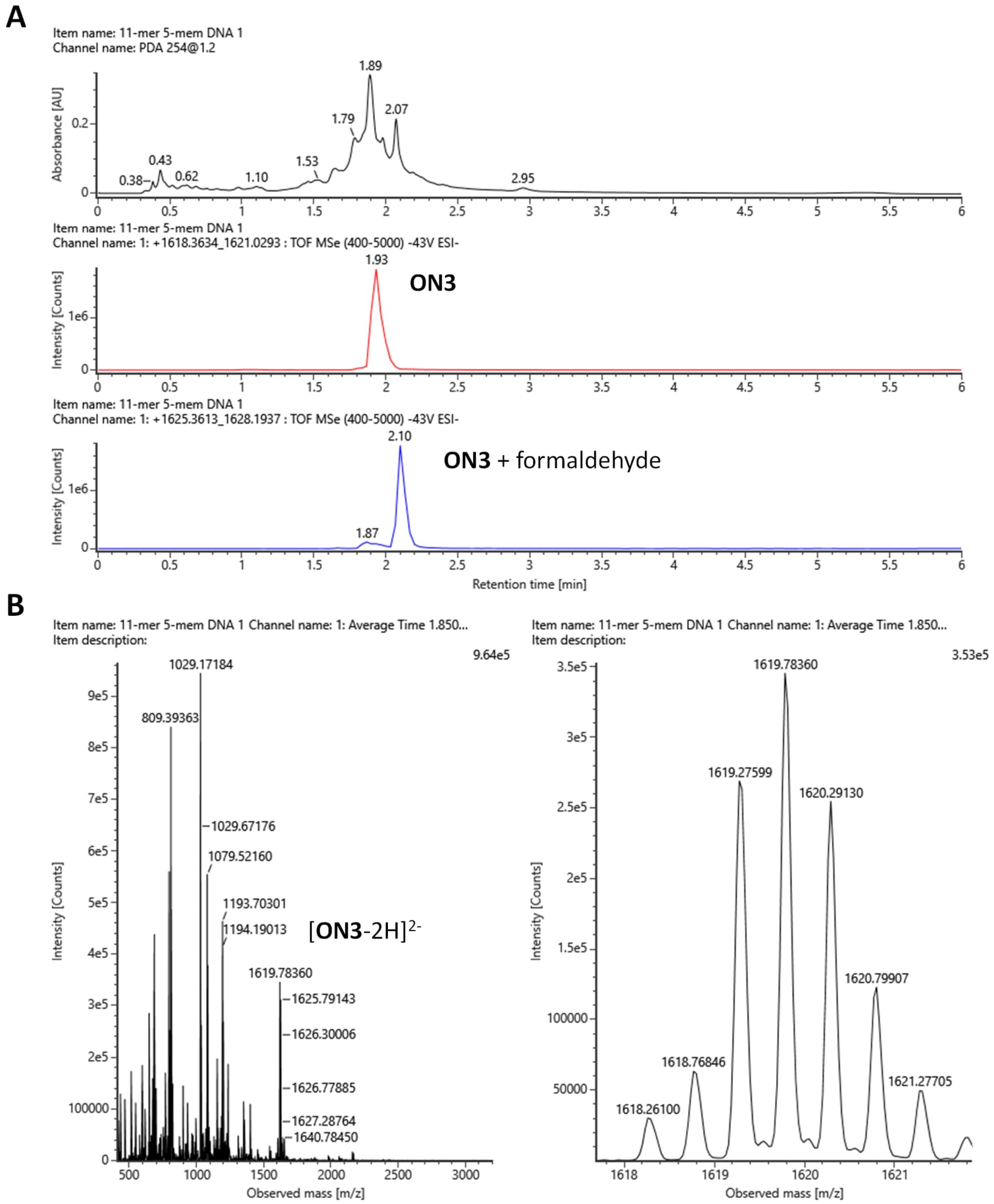

Figure S20. A) UV and extracted ion UPLC traces and B) mass spectra of oligonucleotide ON3; ACQUITY Premier OST column (50 × 2.1 mm, 1.7 µm); flow rate 0.4 mL min^{-1}; linear gradient (5—25 % over 4 min) of MeOH in aqueous solution of hexafluoroisopropanol (40 mM) and triethylamine (7 mM); λ = 254 nm; T = 60 °C. Besides naked ON3, a peak for the reversible adduct with formaldehyde was observed.

A B

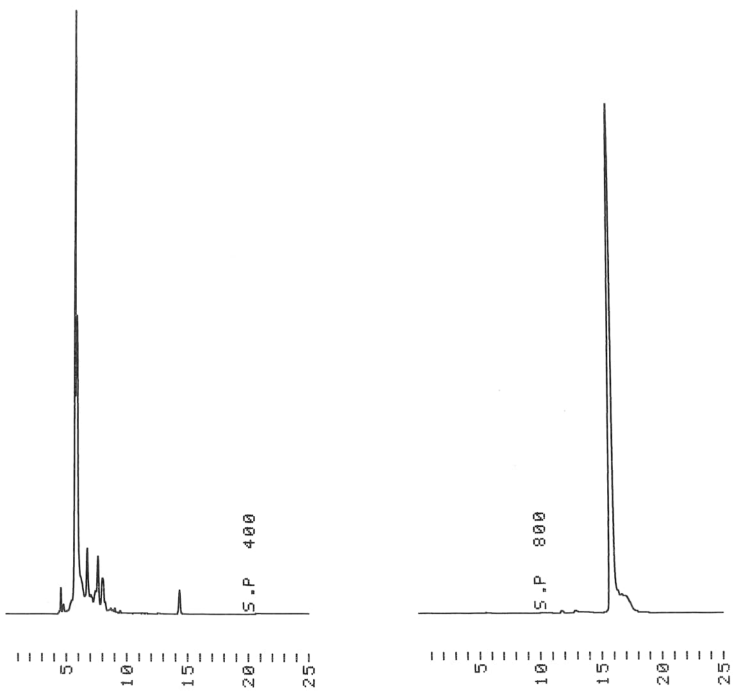

Figure S21. RP-HPLC traces of A) crude product mixture of the synthesis of oligonucleotide ON4a and B) the purified product after treatment with acetic acid; Thermo Scientific ODS Hypersil column (250 × 10 mm, 5 µM); flow rate = 3.0 mL min^{-1}; linear gradient (A: 10—40 % over 25 min and B: 7.5—15 % over 25 min) of MeCN in 50 mM aqueous triethylammonium acetate; λ = 260 nm.

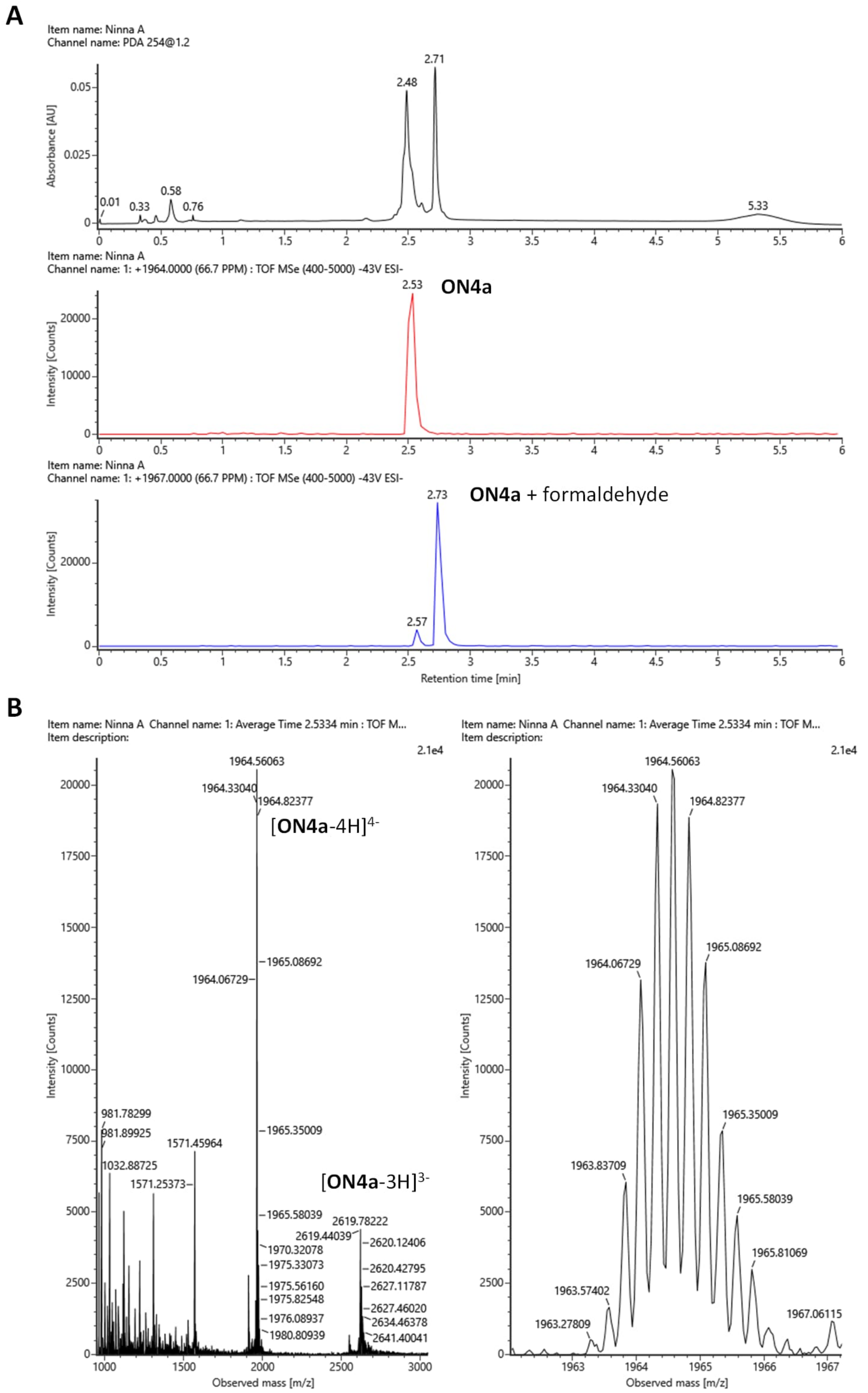

Figure S22. A) UV and extracted ion UPLC traces and B) mass spectra of oligonucleotide ON4a; ACQUITY Premier OST column (50 × 2.1 mm, 1.7 µm); flow rate 0.4 mL min^{-1}; linear gradient (5—25 % over 4 min) of MeOH in aqueous solution of hexafluoroisopropanol (40 mM) and triethylamine (7 mM); λ = 254 nm; T = 60 °C. Besides naked ON4a, a peak for the reversible adduct with formaldehyde was observed.

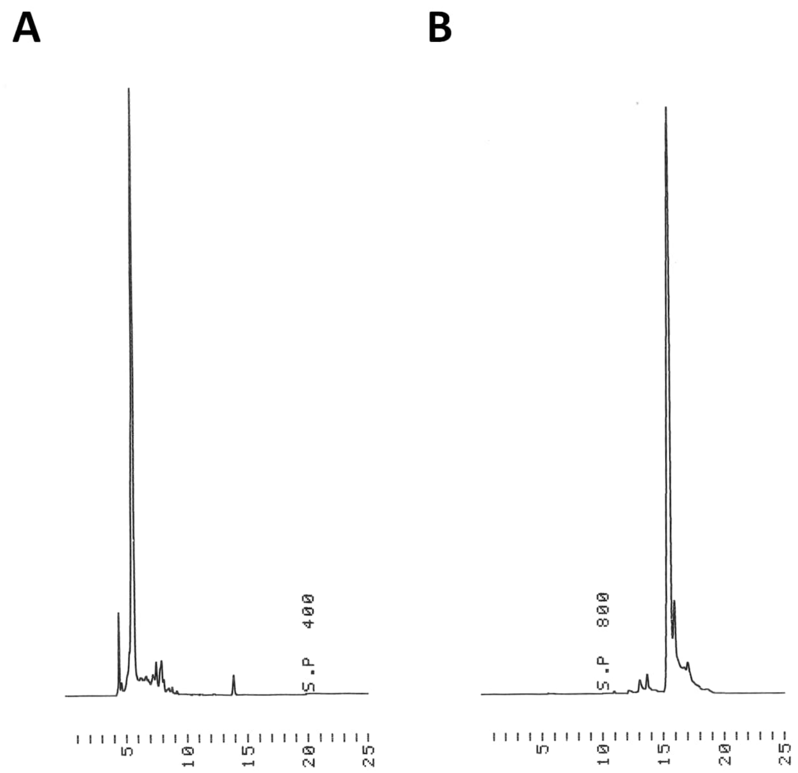

Figure S23. RP-HPLC traces of A) crude product mixture of the synthesis of oligonucleotide ON4c and B) the purified product after treatment with acetic acid; Thermo Scientific ODS Hypersil column (250 × 10 mm, 5 µM); flow rate = 3.0 mL min^{-1}; linear gradient (A: 10—40 % over 25 min and B: 7.5—15 % over 25 min) of MeCN in 50 mM aqueous triethylammonium acetate; λ = 260 nm.

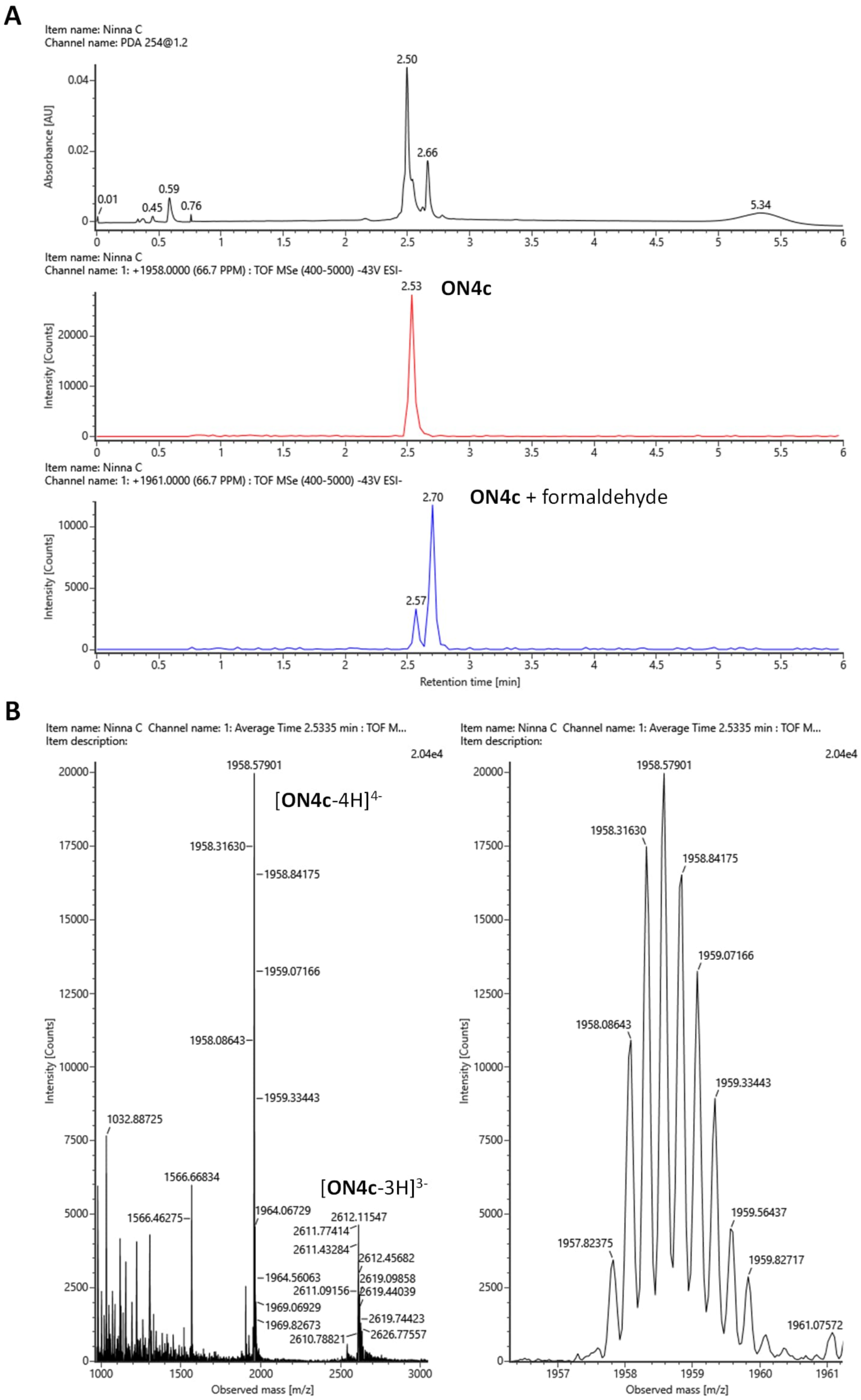

Figure S24. A) UV and extracted ion UPLC traces and B) mass spectra of oligonucleotide ON4c; ACQUITY Premier OST column (50 × 2.1 mm, 1.7 µm); flow rate 0.4 mL min^{-1}; linear gradient (5—25 % over 4 min) of MeOH in aqueous solution of hexafluoroisopropanol (40 mM) and triethylamine (7 mM); λ = 254 nm; T = 60 °C. Besides naked ON4c, a peak for the reversible adduct with formaldehyde was observed.

A **B**

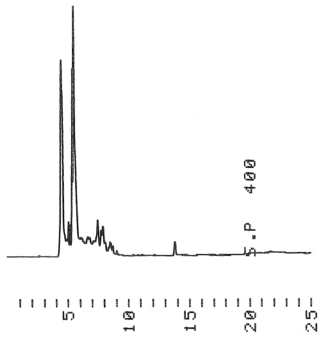

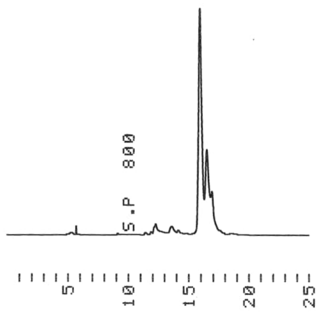

Figure S25. RP-HPLC traces of A) crude product mixture of the synthesis of oligonucleotide ON4g and B) the purified product after treatment with acetic acid; Thermo Scientific ODS Hypersil column (250 × 10 mm, 5 µM); flow rate = 3.0 mL min^{-1}; linear gradient (A: 10—40 % over 25 min and B: 7.5—15 % over 25 min) of MeCN in 50 mM aqueous triethylammonium acetate; λ = 260 nm.

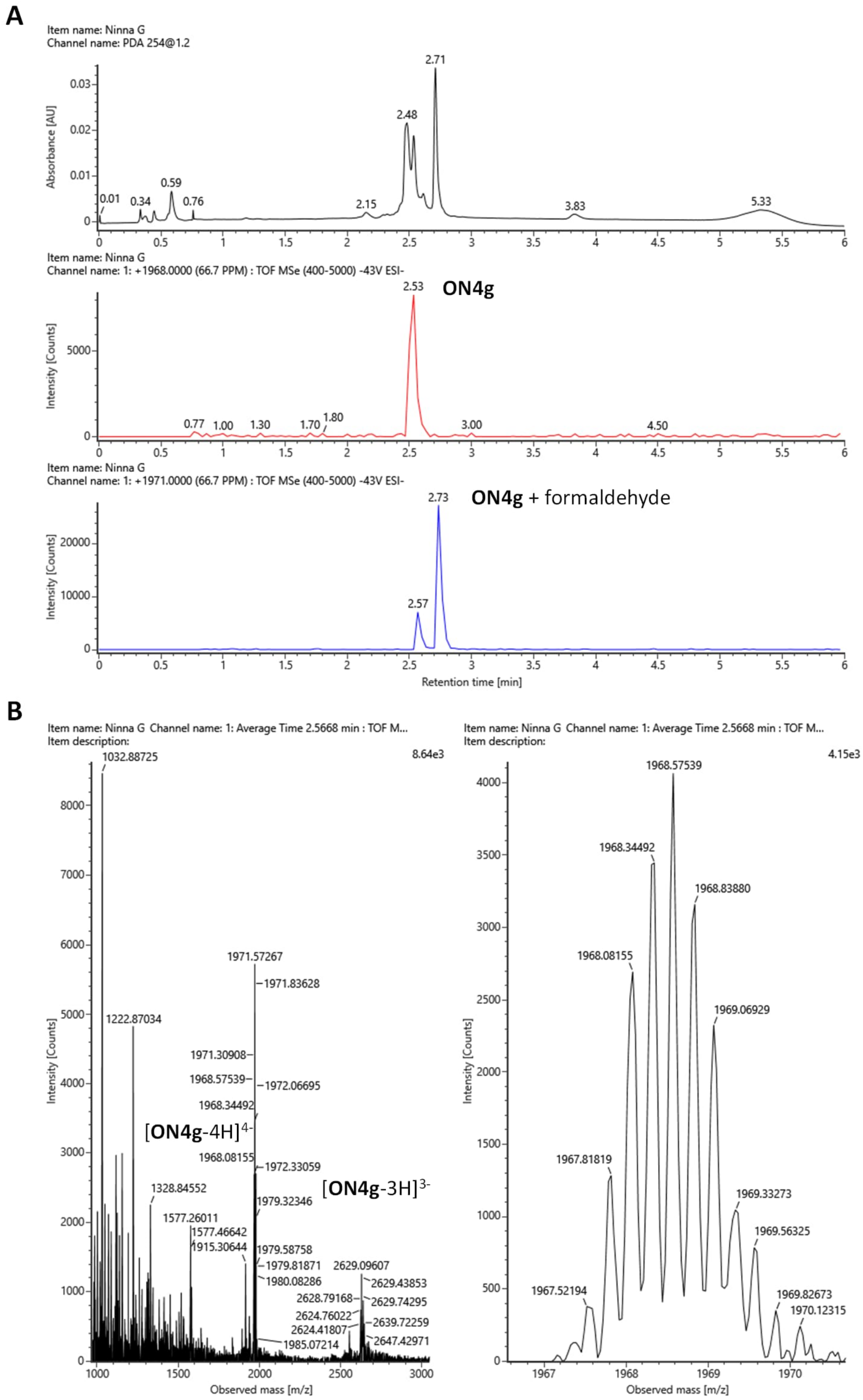

Figure S26. A) UV and extracted ion UPLC traces and B) mass spectra of oligonucleotide ON4g; ACQUITY Premier OST column (50 × 2.1 mm, 1.7 µm); flow rate 0.4 mL min^{-1}; linear gradient (5—25 % over 4 min) of MeOH in aqueous solution of hexafluoroisopropanol (40 mM) and triethylamine (7 mM); λ = 254 nm; T = 60 °C. Besides naked ON4g, a peak for the reversible adduct with formaldehyde was observed.

A **B**

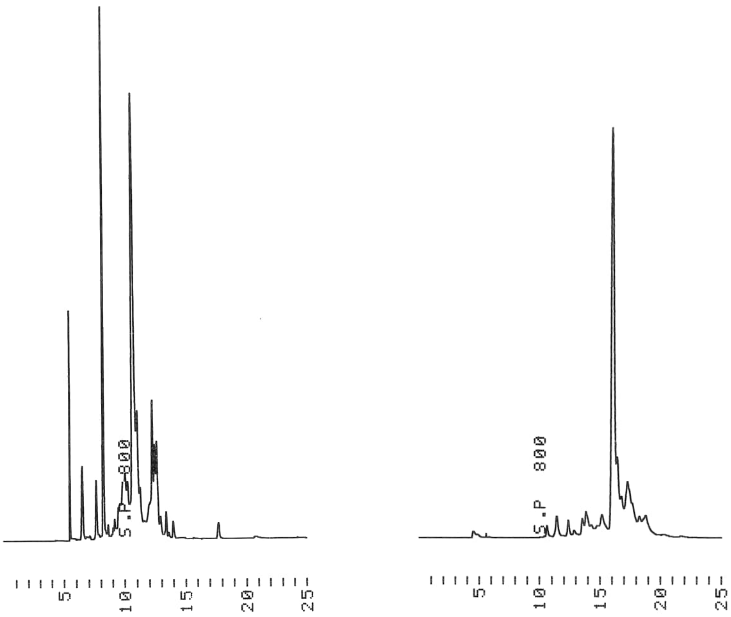

Figure S27. RP-HPLC traces of A) crude product mixture of the synthesis of oligonucleotide ON4t and B) the purified product after treatment with acetic acid; Thermo Scientific ODS Hypersil column (250 × 10 mm, 5 µM); flow rate = 3.0 mL min^{-1}; linear gradient (A: 7.5—30 % over 25 min and B: 7.5—15 % over 25 min) of MeCN in 50 mM aqueous triethylammonium acetate; λ = 260 nm.

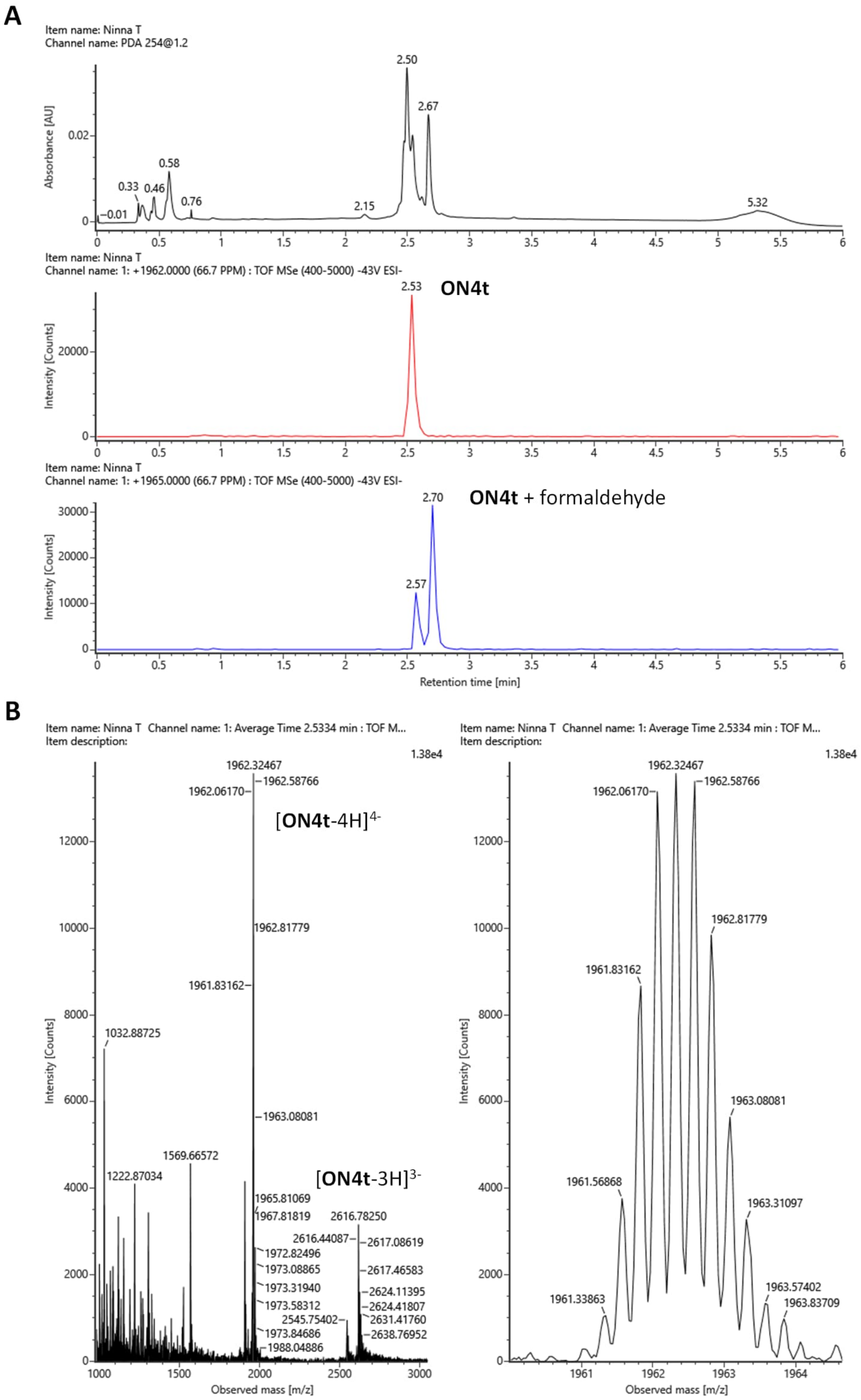

Figure S28. A) UV and extracted ion UPLC traces and B) mass spectra of oligonucleotide ON4t; ACQUITY Premier OST column (50 × 2.1 mm, 1.7 µm); flow rate 0.4 mL min^{-1}; linear gradient (5—25 % over 4 min) of MeOH in aqueous solution of hexafluoroisopropanol (40 mM) and triethylamine (7 mM); λ = 254 nm; T = 60 °C. Besides naked ON4t, a peak for the reversible adduct with formaldehyde was observed.

A **B**

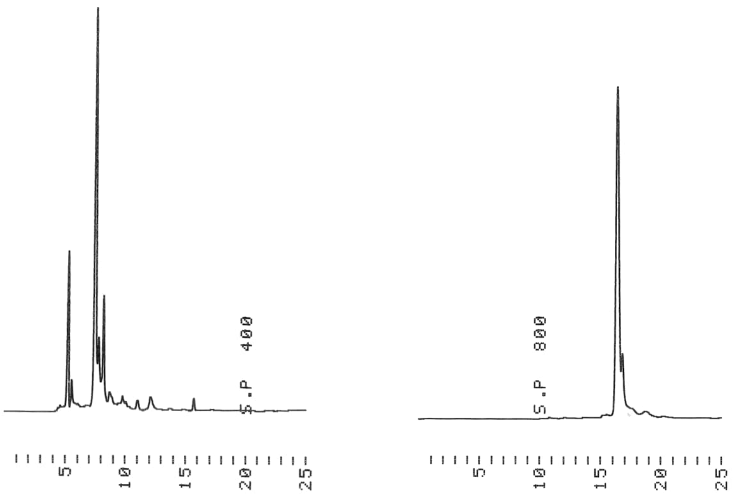

Figure S29. RP-HPLC traces of A) crude product mixture of the synthesis of oligonucleotide ON4s and B) the purified product after treatment with acetic acid; Thermo Scientific ODS Hypersil column (250 × 10 mm, 5 µM); flow rate = 3.0 mL min^{-1}; linear gradient (A: 7.5—30 % over 25 min and B: 7.5—15 % over 25 min) of MeCN in 50 mM aqueous triethylammonium acetate; λ = 260 nm.

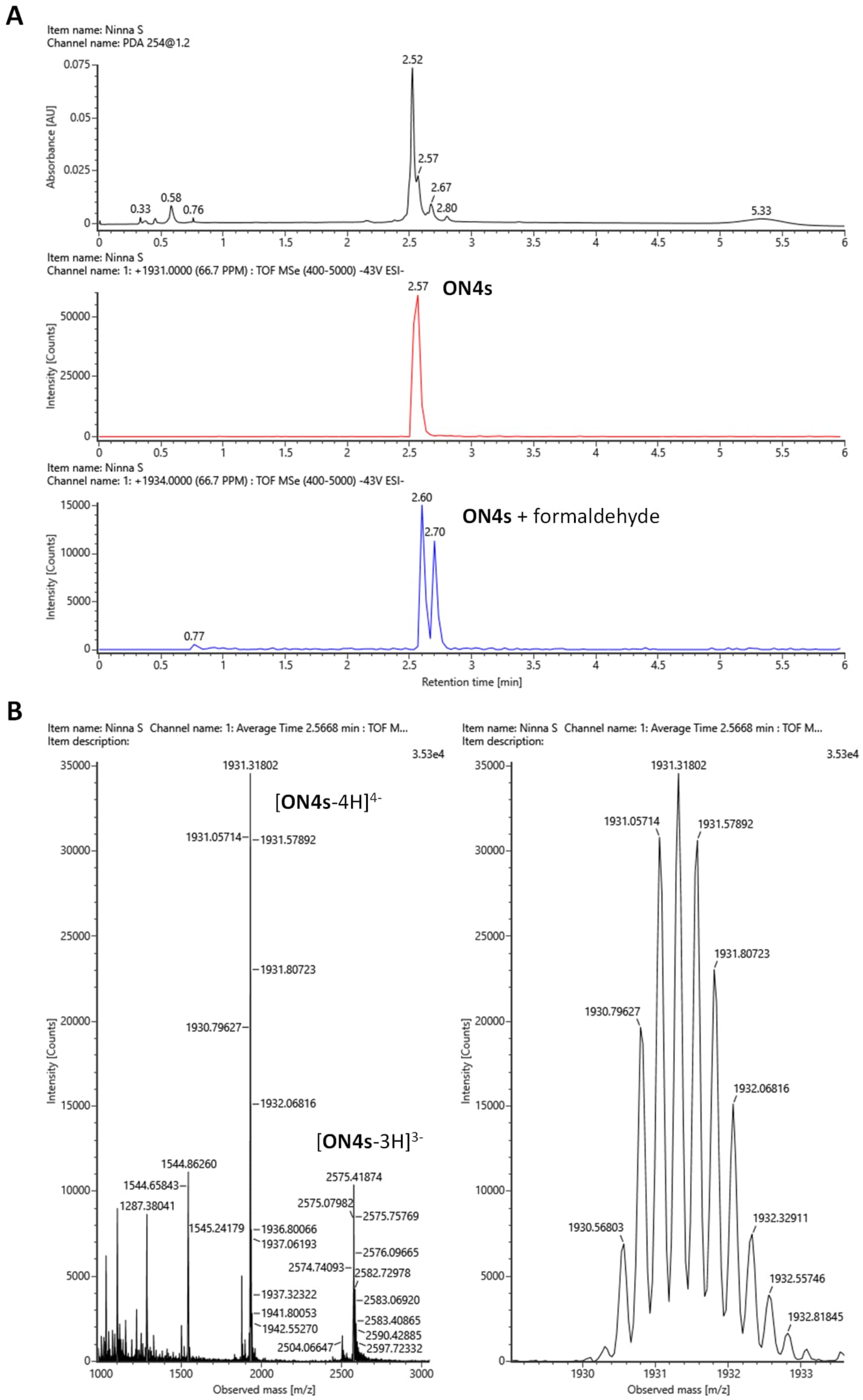

Figure S30. A) UV and extracted ion UPLC traces and B) mass spectra of oligonucleotide ON4s; ACQUITY Premier OST column (50 × 2.1 mm, 1.7 µm); flow rate 0.4 mL min^{-1}; linear gradient (5—25 % over 4 min) of MeOH in aqueous solution of hexafluoroisopropanol (40 mM) and triethylamine (7 mM); λ = 254 nm; T = 60 °C. Besides naked ON4s, a peak for the reversible adduct with formaldehyde was observed.

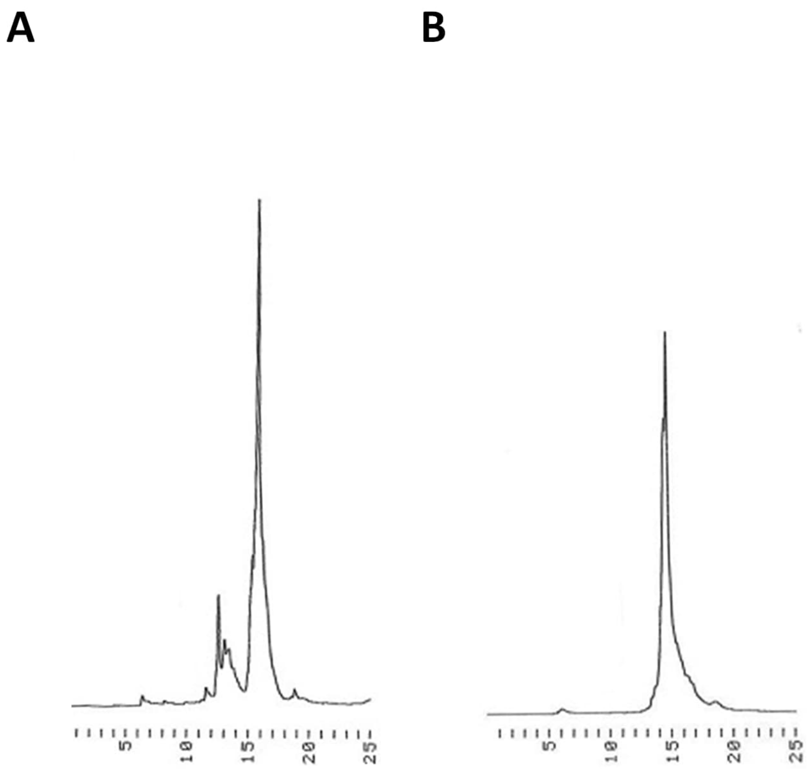

Figure S31. RP-HPLC traces of A) crude product mixture of the synthesis of oligonucleotide ON5 and B) the purified product after treatment with acetic acid; Thermo Scientific ODS Hypersil column (250 × 10 mm, 5 µM); flow rate = 3.0 mL min^{-1}; linear gradient (10—40 % over 25 min) of MeCN in 50 mM aqueous triethylammonium acetate; λ = 260 nm.

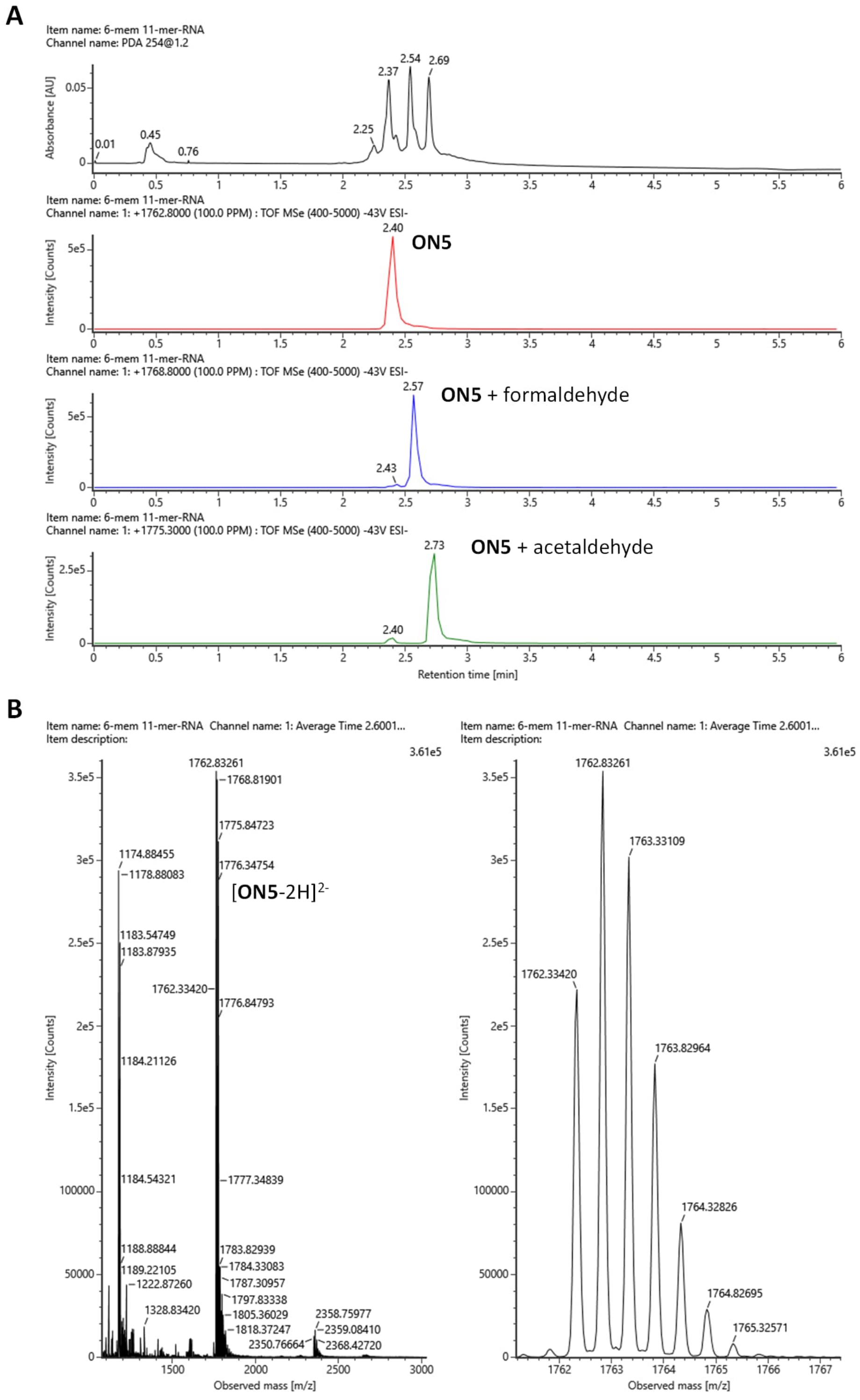

Figure S32. A) UV and extracted ion UPLC traces and B) mass spectra of oligonucleotide ON5; ACQUITY Premier OST column (50 × 2.1 mm, 1.7 µm); flow rate 0.4 mL min^{-1}; linear gradient (5—25 % over 4 min) of MeOH in aqueous solution of hexafluoroisopropanol (40 mM) and triethylamine (7 mM); λ = 254 nm; T = 60 °C. Besides naked ON5, peaks for the reversible adducts with formaldehyde and acetaldehyde were observed.

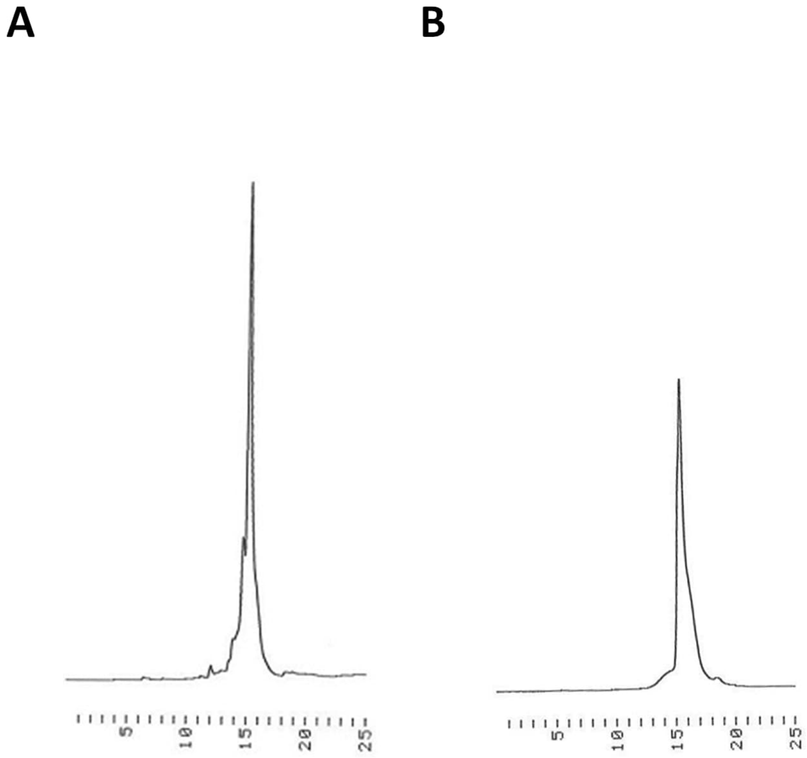

Figure S33. RP-HPLC traces of A) crude product mixture of the synthesis of oligonucleotide ON6a and B) the purified product after treatment with acetic acid; Thermo Scientific ODS Hypersil column (250 × 10 mm, 5 µM); flow rate = 3.0 mL min^{-1}; linear gradient (10—40 % over 25 min) of MeCN in 50 mM aqueous triethylammonium acetate; λ = 260 nm.

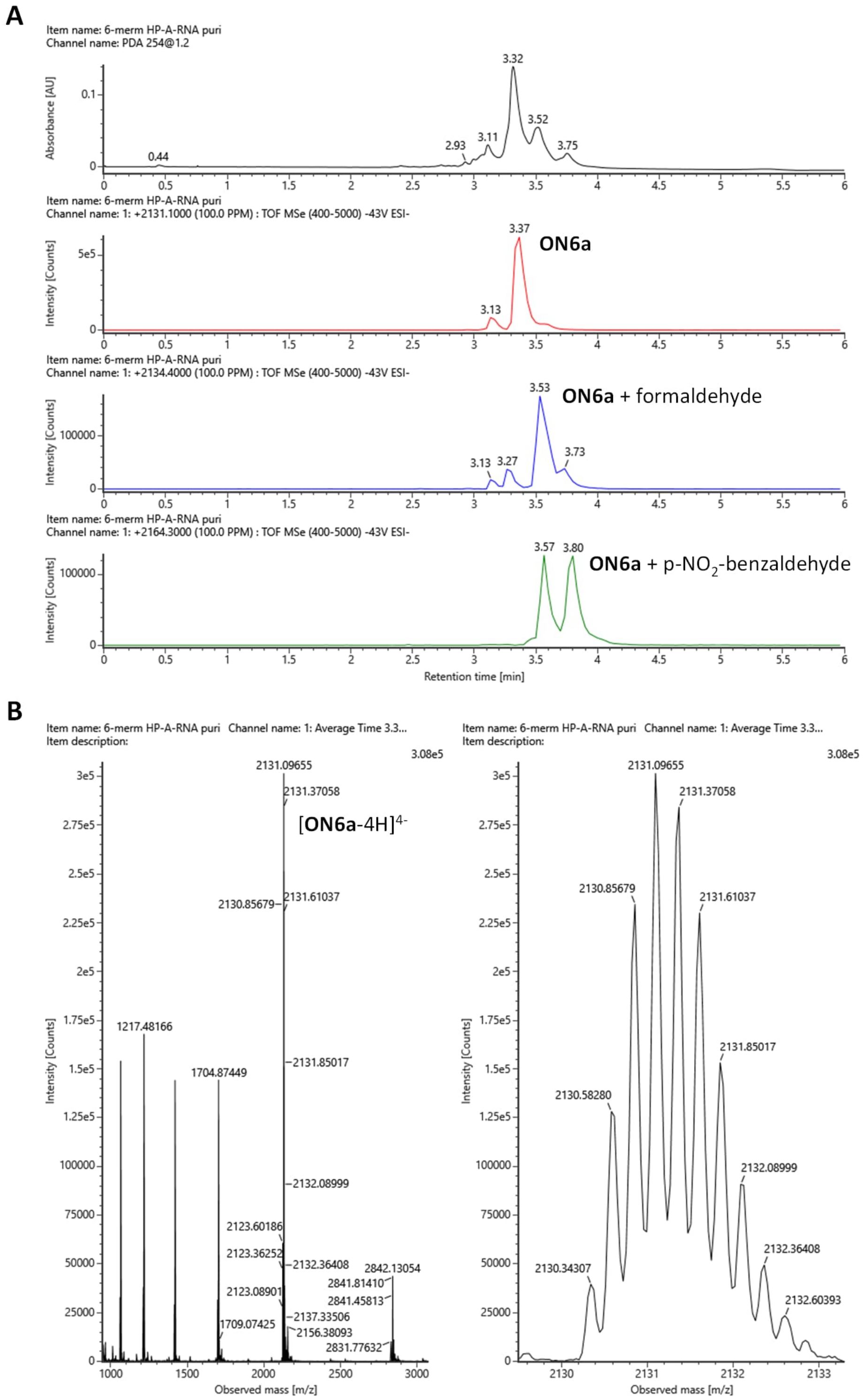

Figure S34. A) UV and extracted ion UPLC traces and B) mass spectra of oligonucleotide ON6a; ACQUITY Premier OST column (50 × 2.1 mm, 1.7 µm); flow rate 0.4 mL min^{-1}; linear gradient (5—25 % over 4 min) of MeOH in aqueous solution of hexafluoroisopropanol (40 mM) and triethylamine (7 mM); λ = 254 nm; T = 60 °C. Besides naked ON6a, peaks for the reversible adducts with formaldehyde and p-nitrobenzaldehyde (residual protecting group) were observed.

A B

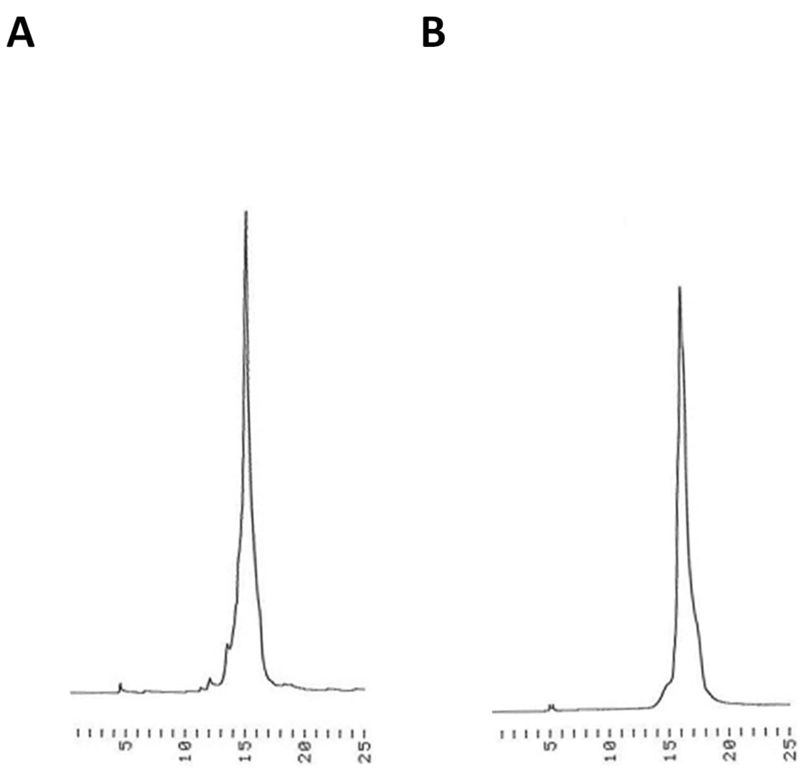

Figure S35. RP-HPLC traces of A) crude product mixture of the synthesis of oligonucleotide ON6c and B) the purified product after treatment with acetic acid; Thermo Scientific ODS Hypersil column (250 × 10 mm, 5 μM); flow rate = 3.0 mL min^{-1}; linear gradient (10—40 % over 25 min) of MeCN in 50 mM aqueous triethylammonium acetate; λ = 260 nm.

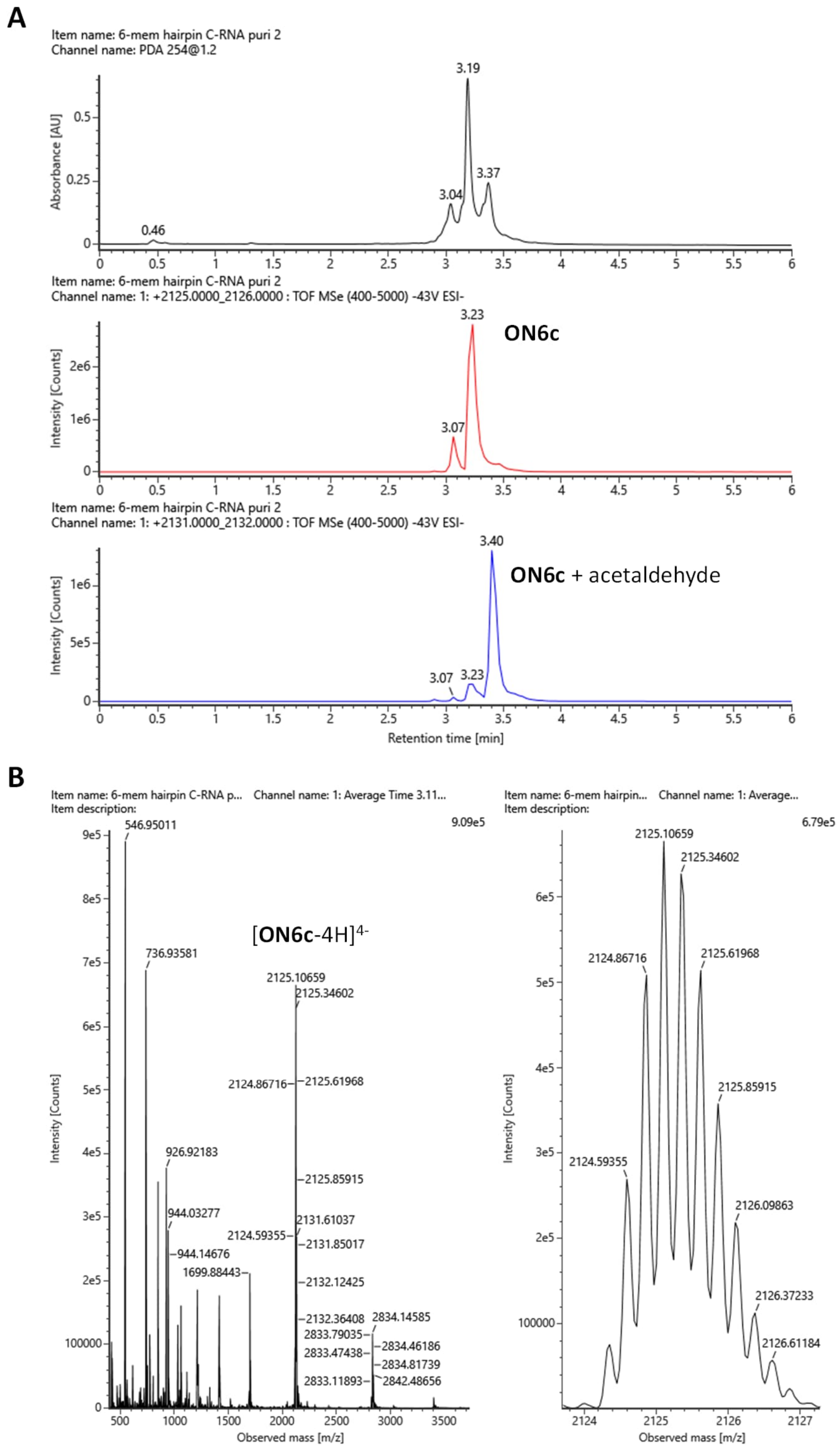

Figure S36. A) UV and extracted ion UPLC traces and B) mass spectra of oligonucleotide ON6c; ACQUITY Premier OST column (50 × 2.1 mm, 1.7 µm); flow rate 0.4 mL min^{-1}; linear gradient (5—25 % over 4 min) of MeOH in aqueous solution of hexafluoroisopropanol (40 mM) and triethylamine (7 mM); λ = 254 nm; T = 60 °C. Besides naked ON6c, a peak for the reversible adduct with acetaldehyde was observed.

A B

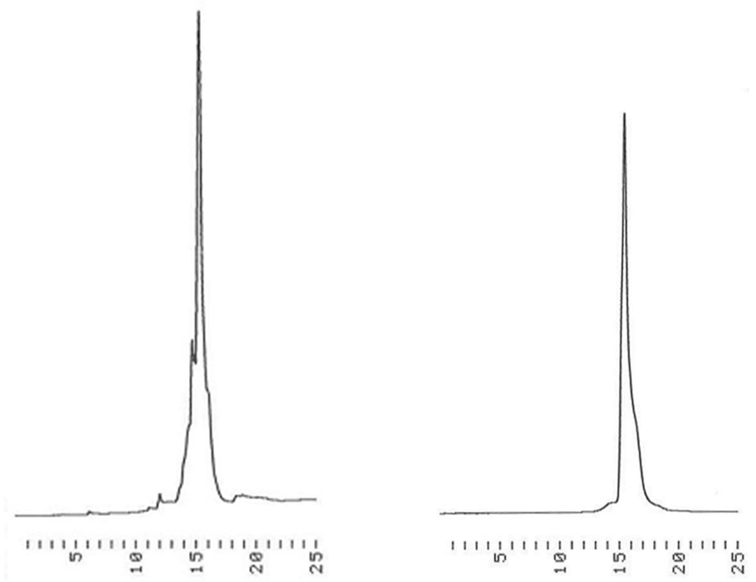

Figure S37. RP-HPLC traces of A) crude product mixture of the synthesis of oligonucleotide ON6g and B) the purified product after treatment with acetic acid; Thermo Scientific ODS Hypersil column (250 × 10 mm, 5 µM); flow rate = 3.0 mL min^{-1}; linear gradient (10—40 % over 25 min) of MeCN in 50 mM aqueous triethylammonium acetate; λ = 260 nm.

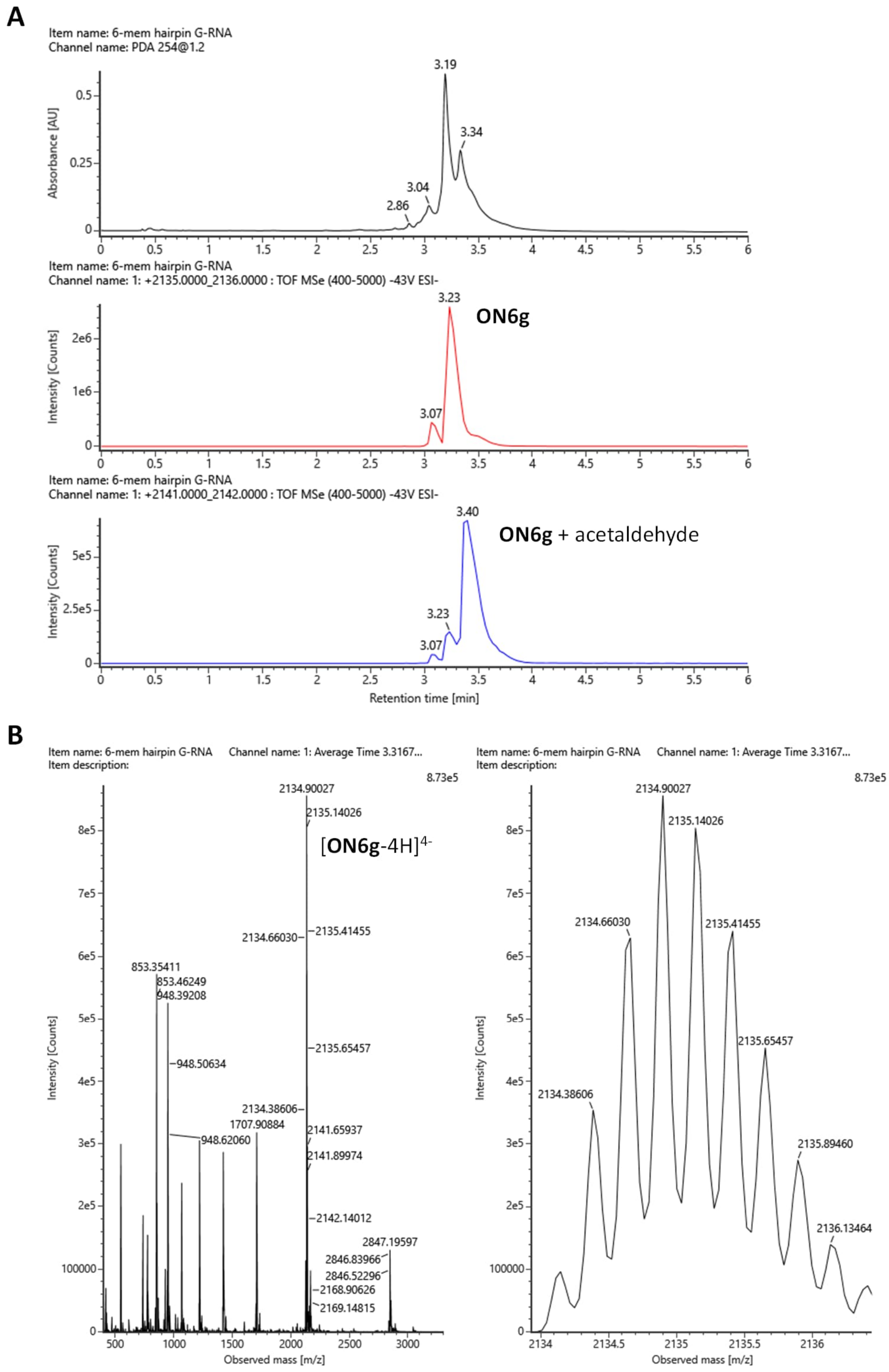

Figure S38. A) UV and extracted ion UPLC traces and B) mass spectra of oligonucleotide ON6g; ACQUITY Premier OST column (50 × 2.1 mm, 1.7 µm); flow rate 0.4 mL min^{-1}; linear gradient (5—25 % over 4 min) of MeOH in aqueous solution of hexafluoroisopropanol (40 mM) and triethylamine (7 mM); λ = 254 nm; T = 60 °C. Besides naked ON6g, a peak for the reversible adduct with acetaldehyde was observed.

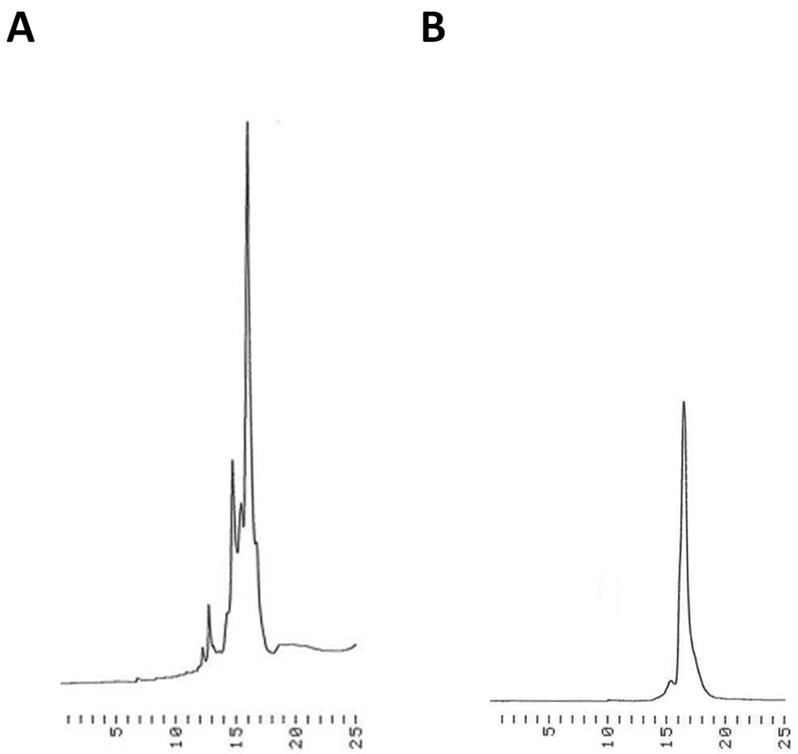

Figure S39. RP-HPLC traces of A) crude product mixture of the synthesis of oligonucleotide ON6u and B) the purified product after treatment with acetic acid; Thermo Scientific ODS Hypersil column (250 × 10 mm, 5 µM); flow rate = 3.0 mL min^{-1}; linear gradient (10—40 % over 25 min) of MeCN in 50 mM aqueous triethylammonium acetate; λ = 260 nm.

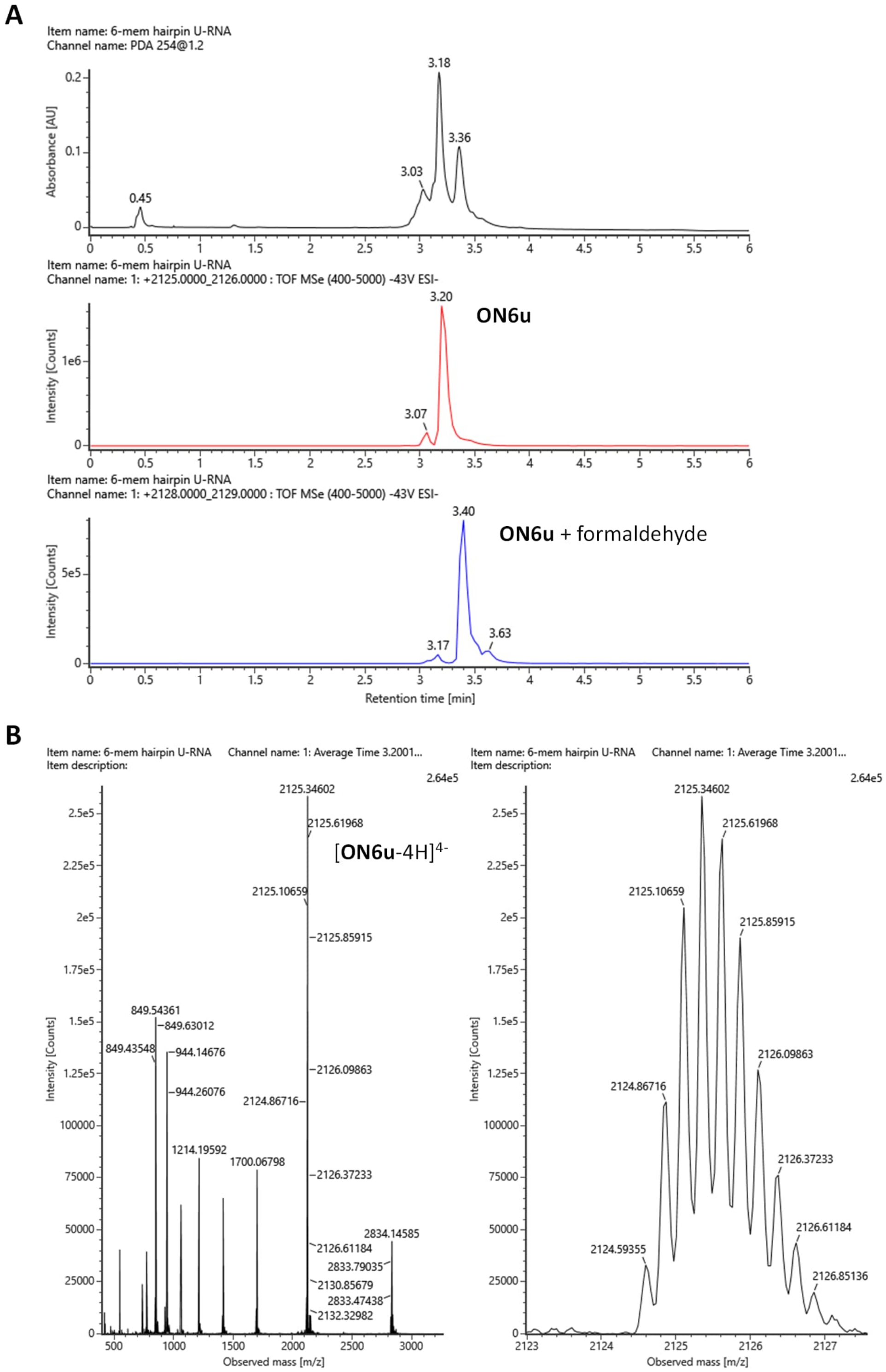

Figure S40. A) UV and extracted ion UPLC traces and B) mass spectra of oligonucleotide ON6u; ACQUITY Premier OST column (50 × 2.1 mm, 1.7 µm); flow rate 0.4 mL min^{-1}; linear gradient (5—25 % over 4 min) of MeOH in aqueous solution of hexafluoroisopropanol (40 mM) and triethylamine (7 mM); λ = 254 nm; T = 60 °C. Besides naked ON6u, a peak for the reversible adduct with formaldehyde was observed.

A **B**

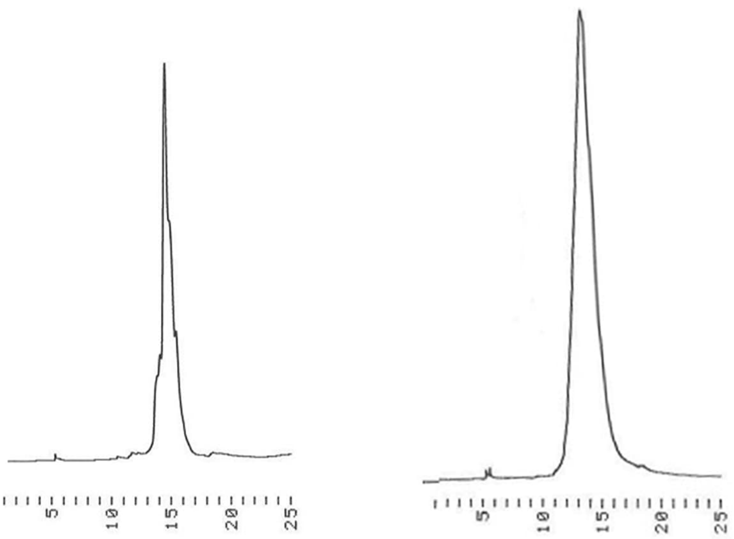

Figure S41. RP-HPLC traces of A) crude product mixture of the synthesis of oligonucleotide ON6s and B) the purified product after treatment with acetic acid; Thermo Scientific ODS Hypersil column (250 × 10 mm, 5 µM); flow rate = 3.0 mL min^{-1}; linear gradient (10—40 % over 25 min) of MeCN in 50 mM aqueous triethylammonium acetate; λ = 260 nm.

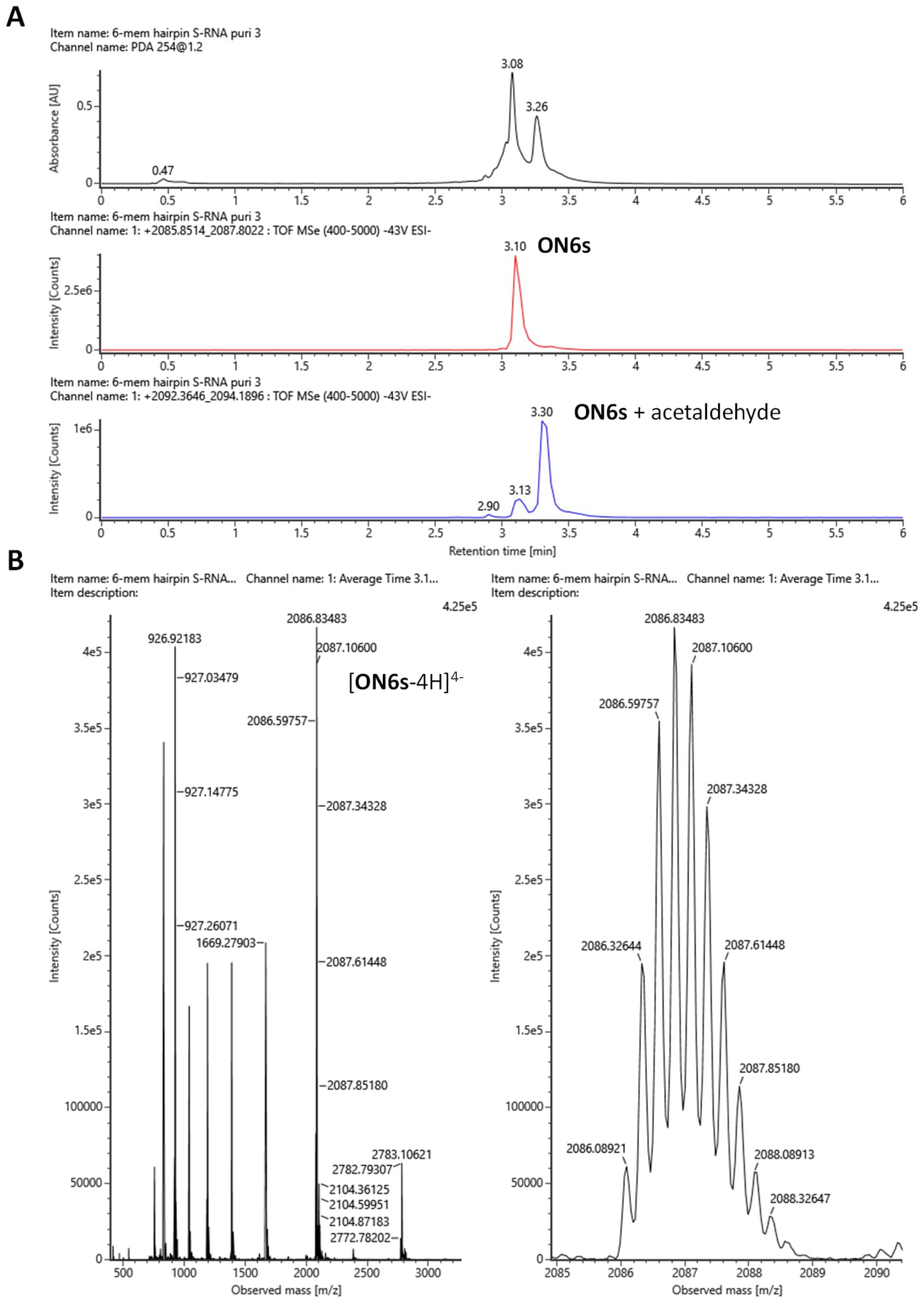

Figure S42. A) UV and extracted ion UPLC traces and B) mass spectra of oligonucleotide ON6s; ACQUITY Premier OST column (50 × 2.1 mm, 1.7 µm); flow rate 0.4 mL min^{-1}; linear gradient (5—25 % over 4 min) of MeOH in aqueous solution of hexafluoroisopropanol (40 mM) and triethylamine (7 mM); λ = 254 nm; T = 60 °C. Besides naked ON6s, a peak for the reversible adduct with formaldehyde was observed.

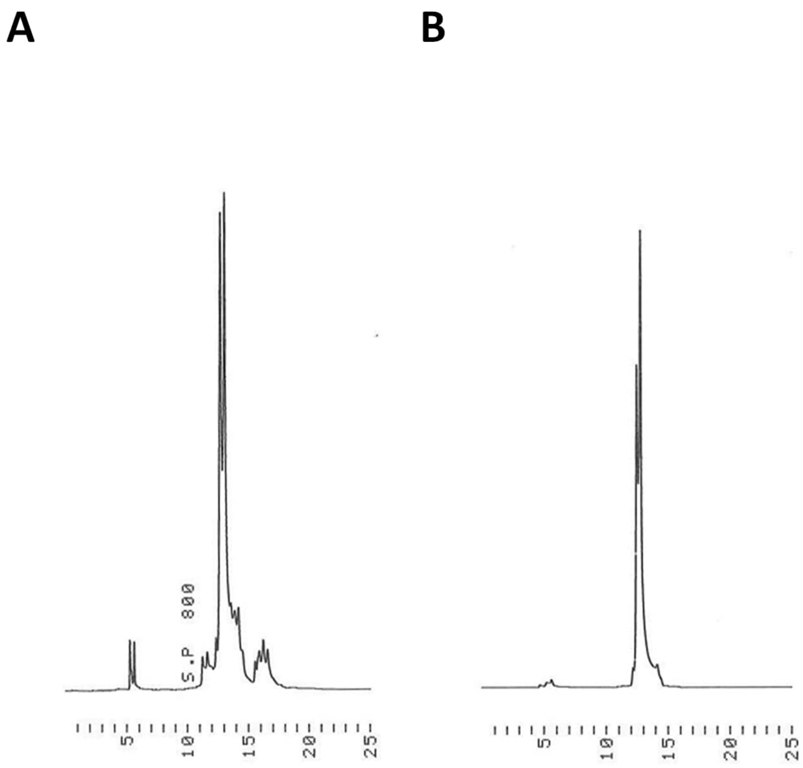

Figure S43. RP-HPLC traces of A) crude product mixture of the synthesis of oligonucleotide ON7 and B) the purified product after treatment with acetic acid; Thermo Scientific ODS Hypersil column (250 × 10 mm, 5 μM); flow rate = 3.0 mL min^{-1}; linear gradient (10—40 % over 25 min) of MeCN in 50 mM aqueous triethylammonium acetate; λ = 260 nm.

A

B

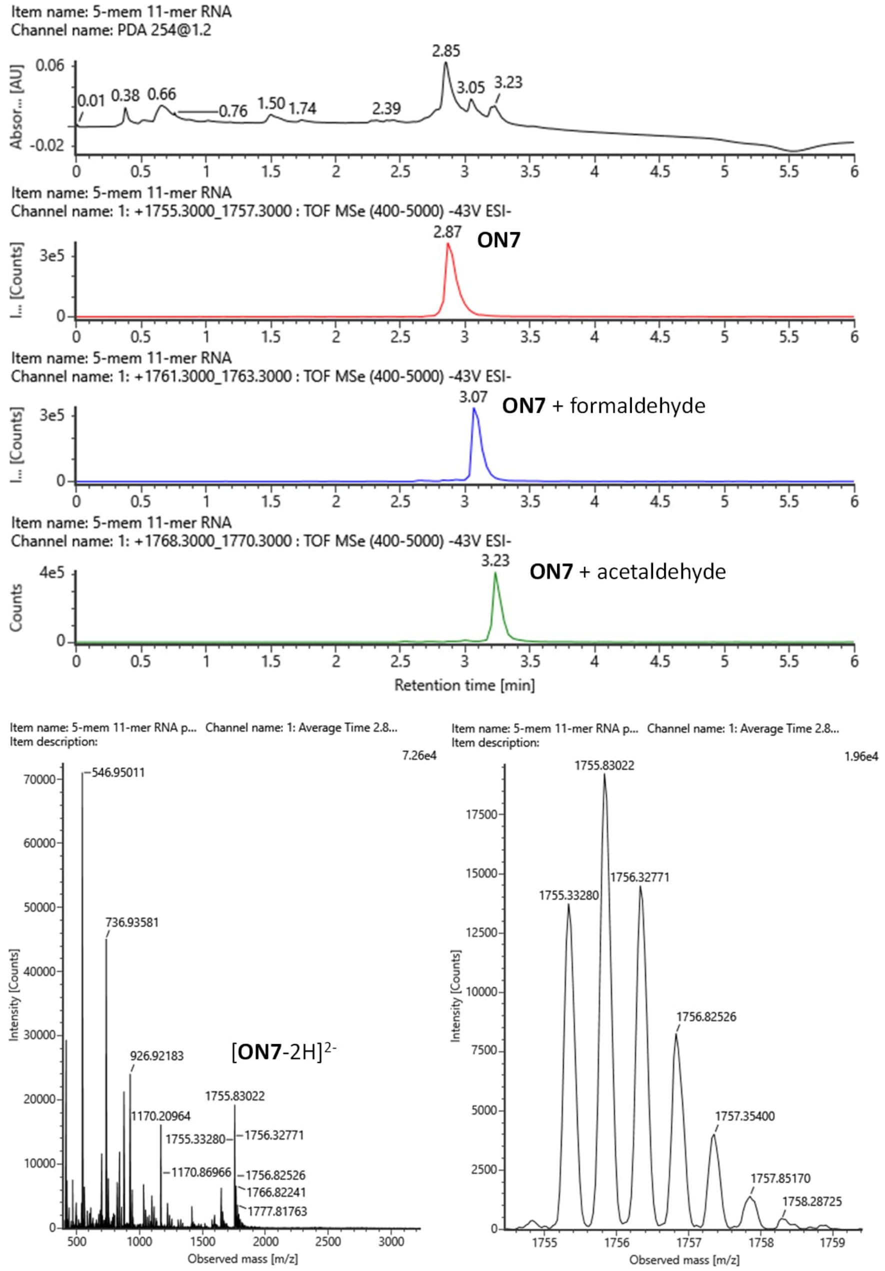

Figure S44. A) UV and extracted ion UPLC traces and B) mass spectra of oligonucleotide ON7; ACQUITY Premier OST column (50 × 2.1 mm, 1.7 µm); flow rate 0.4 mL min^{-1}; linear gradient (5—25 % over 4 min) of MeOH in aqueous solution of hexafluoroisopropanol (40 mM) and triethylamine (7 mM); λ = 254 nm; T = 60 °C. Besides naked ON7, a peak for the reversible adduct with formaldehyde was observed.

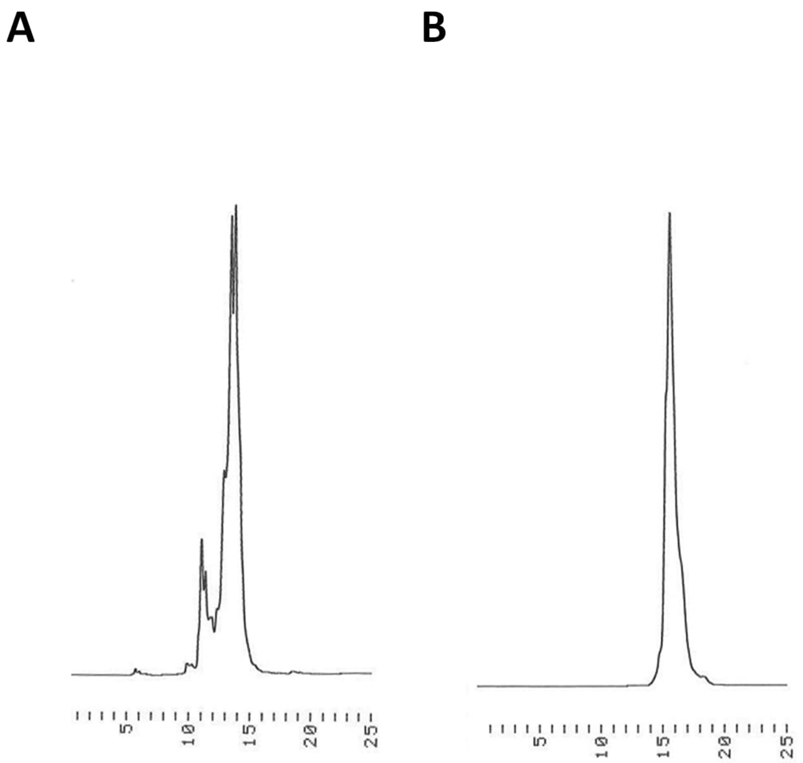

Figure S45. RP-HPLC traces of A) crude product mixture of the synthesis of oligonucleotide ON8a and B) the purified product after treatment with acetic acid; Thermo Scientific ODS Hypersil column (250 × 10 mm, 5 µM); flow rate = 3.0 mL min^{-1}; linear gradient (10—40 % over 25 min) of MeCN in 50 mM aqueous triethylammonium acetate; λ = 260 nm.

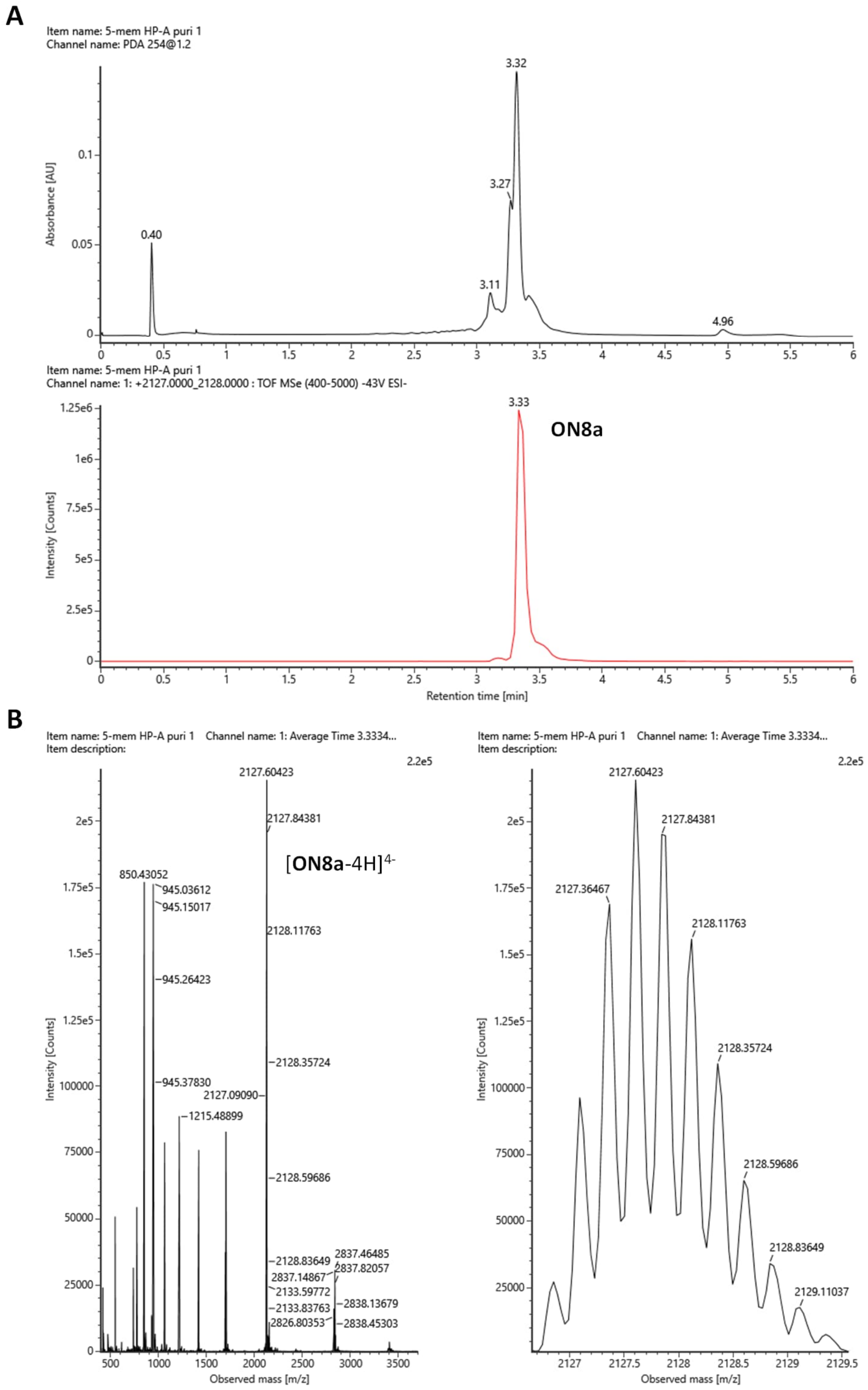

Figure S46. A) UV and extracted ion UPLC traces and B) mass spectra of oligonucleotide ON8a; ACQUITY Premier OST column (50 × 2.1 mm, 1.7 µm); flow rate 0.4 mL min^{-1}; linear gradient (5—25 % over 4 min) of MeOH in aqueous solution of hexafluoroisopropanol (40 mM) and triethylamine (7 mM); λ = 254 nm; T = 60 °C.

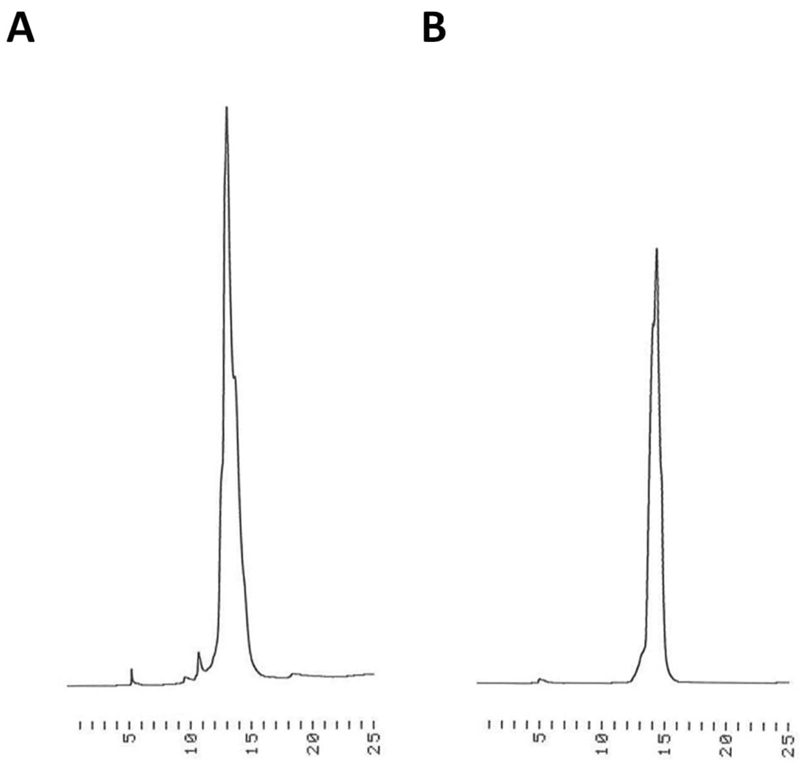

Figure S47. RP-HPLC traces of A) crude product mixture of the synthesis of oligonucleotide ON8c and B) the purified product after treatment with acetic acid; Thermo Scientific ODS Hypersil column (250 × 10 mm, 5 µM); flow rate = 3.0 mL min^{-1}; linear gradient (10—40 % over 25 min) of MeCN in 50 mM aqueous triethylammonium acetate; λ = 260 nm.

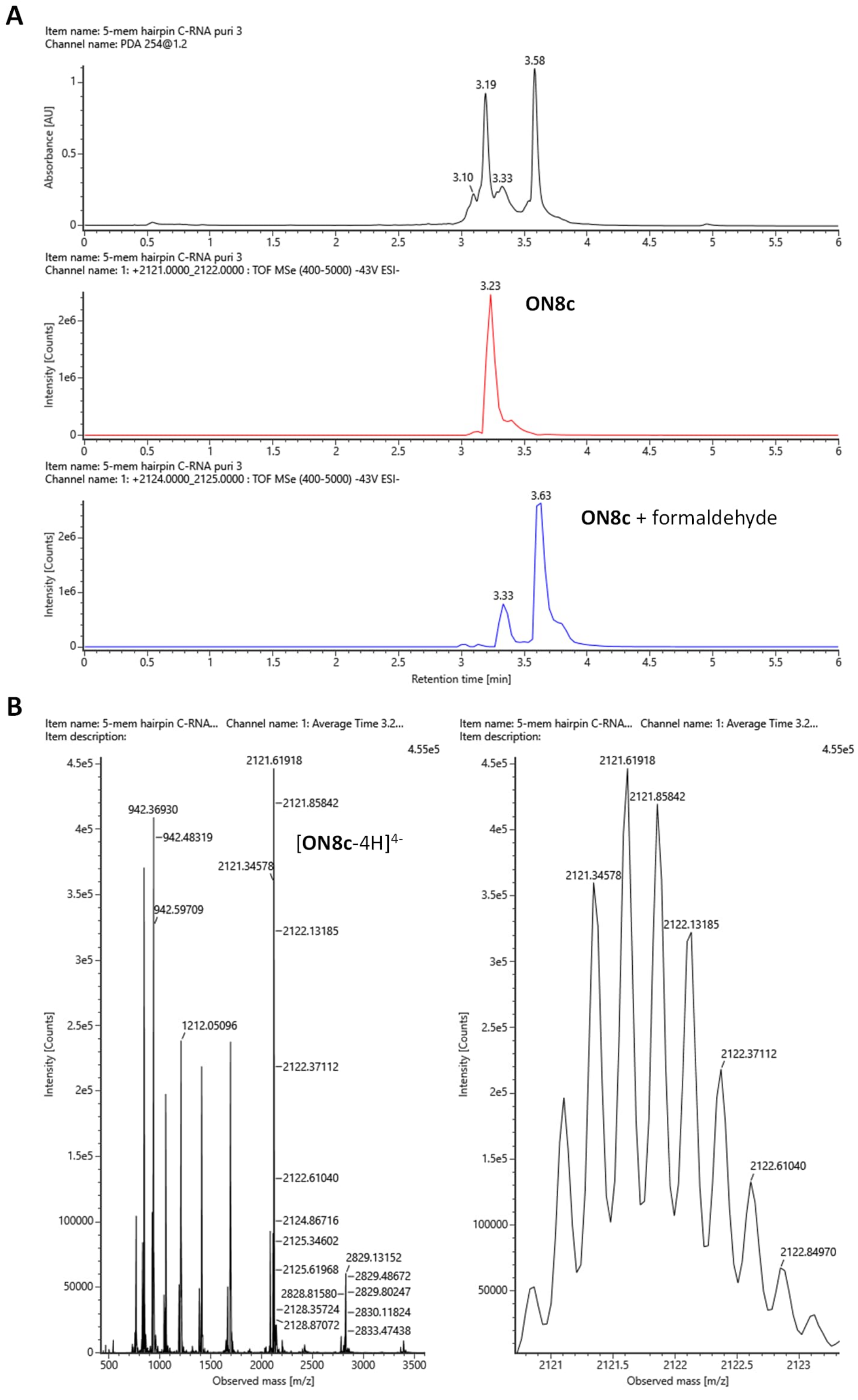

Figure S48. A) UV and extracted ion UPLC traces and B) mass spectra of oligonucleotide ON8c; ACQUITY Premier OST column (50 × 2.1 mm, 1.7 µm); flow rate 0.4 mL min^{-1}; linear gradient (5—25 % over 4 min) of MeOH in aqueous solution of hexafluoroisopropanol (40 mM) and triethylamine (7 mM); λ = 254 nm; T = 60 °C. Besides naked ON8c, a peak for the reversible adduct with formaldehyde was observed.

A B

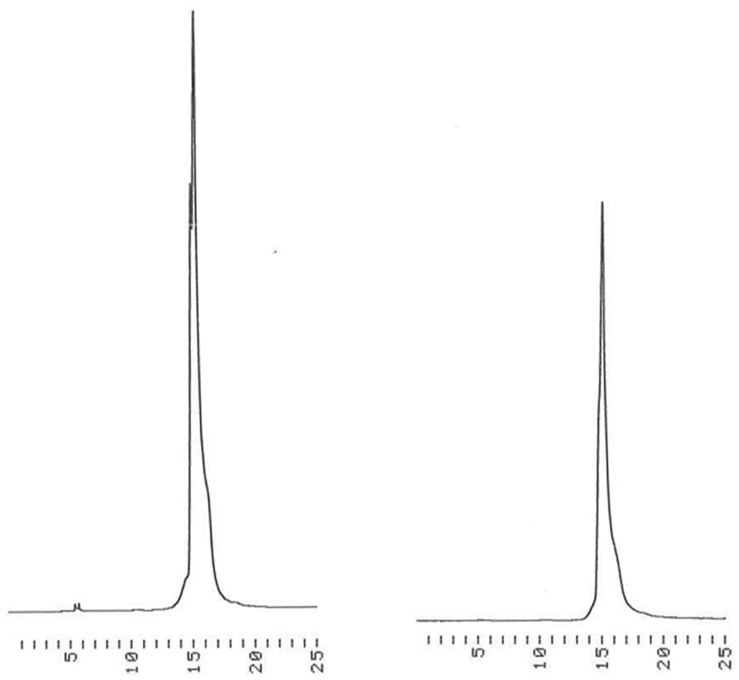

Figure S49. RP-HPLC traces of A) crude product mixture of the synthesis of oligonucleotide ON8g and B) the purified product after treatment with acetic acid; Thermo Scientific ODS Hypersil column (250 × 10 mm, 5 µM); flow rate = 3.0 mL min^{-1}; linear gradient (10—40 % over 25 min) of MeCN in 50 mM aqueous triethylammonium acetate; λ = 260 nm.

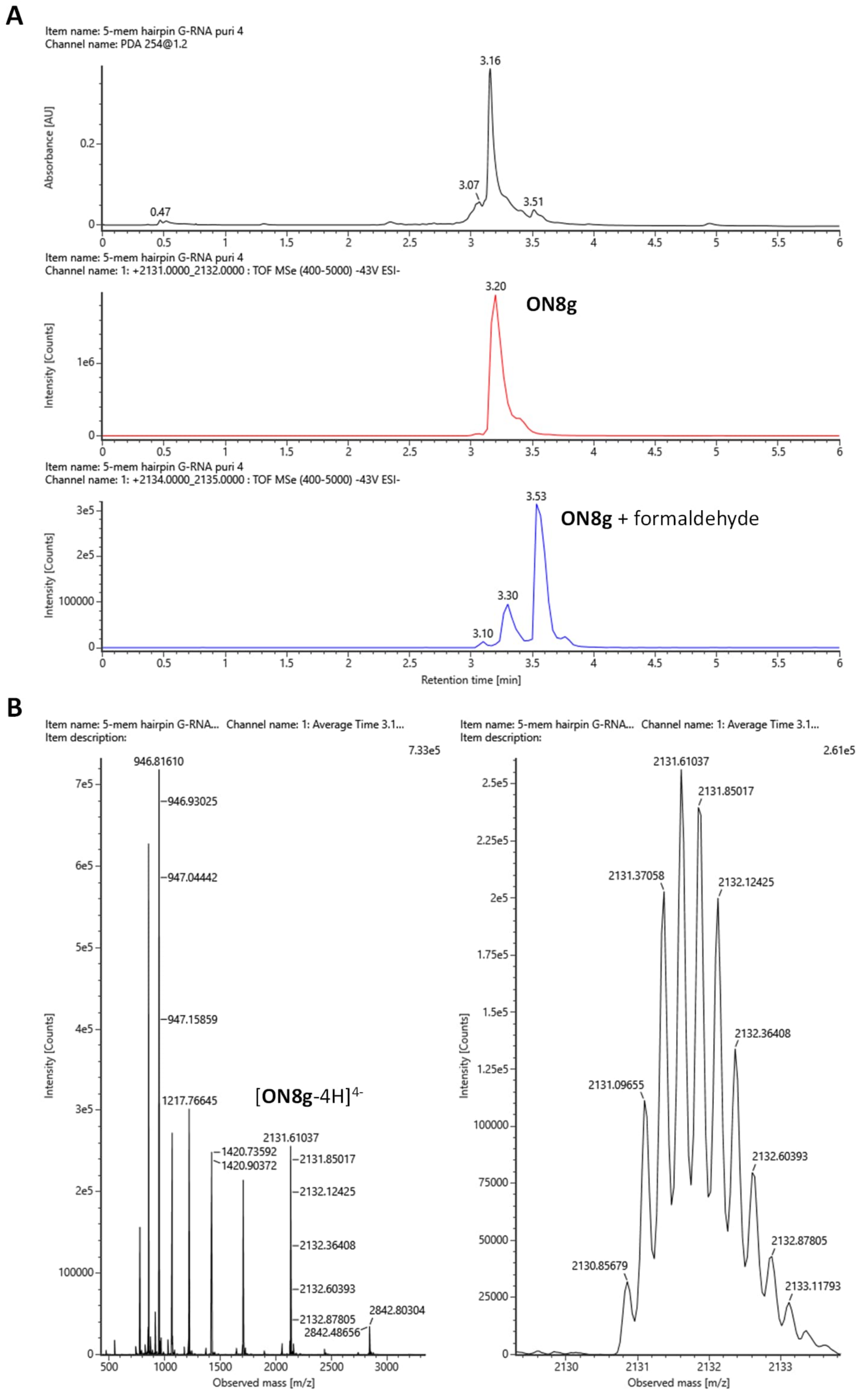

Figure S50. A) UV and extracted ion UPLC traces and B) mass spectra of oligonucleotide ON8g; ACQUITY Premier OST column (50 × 2.1 mm, 1.7 µm); flow rate 0.4 mL min^{-1}; linear gradient (5—25 % over 4 min) of MeOH in aqueous solution of hexafluoroisopropanol (40 mM) and triethylamine (7 mM); λ = 254 nm; T = 60 °C. Besides naked ON8g, a peak for the reversible adduct with formaldehyde was observed.

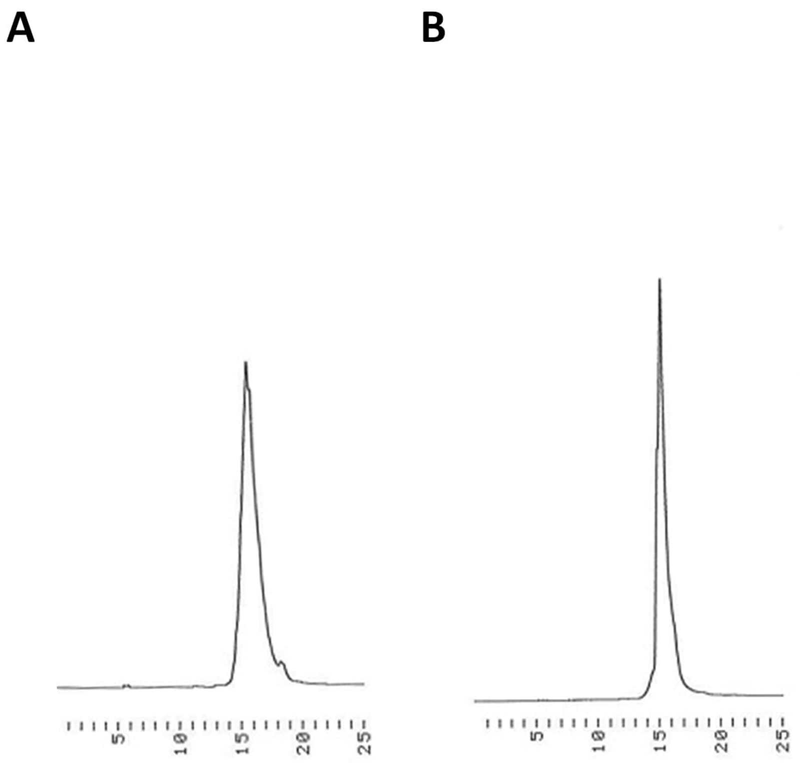

Figure S51. RP-HPLC traces of A) crude product mixture of the synthesis of oligonucleotide ON8u and B) the purified product after treatment with acetic acid; Thermo Scientific ODS Hypersil column (250 × 10 mm, 5 µM); flow rate = 3.0 mL min^{-1}; linear gradient (10—40 % over 25 min) of MeCN in 50 mM aqueous triethylammonium acetate; λ = 260 nm.

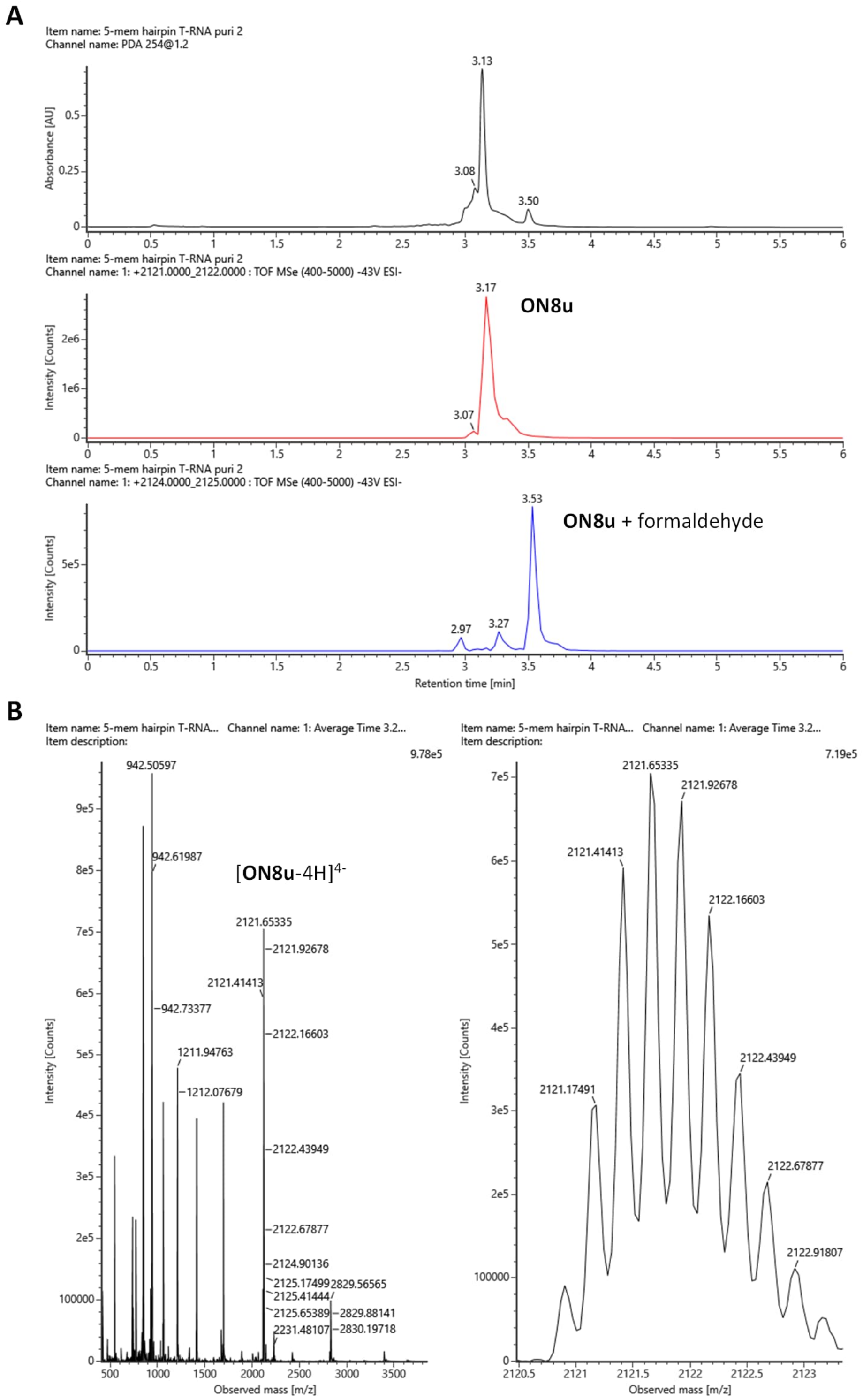

Figure S52. A) UV and extracted ion UPLC traces and B) mass spectra of oligonucleotide ON8u; ACQUITY Premier OST column (50 × 2.1 mm, 1.7 µm); flow rate 0.4 mL min^{-1}; linear gradient (5—25 % over 4 min) of MeOH in aqueous solution of hexafluoroisopropanol (40 mM) and triethylamine (7 mM); λ = 254 nm; T = 60 °C. Besides naked ON8u, a peak for the reversible adduct with formaldehyde was observed.

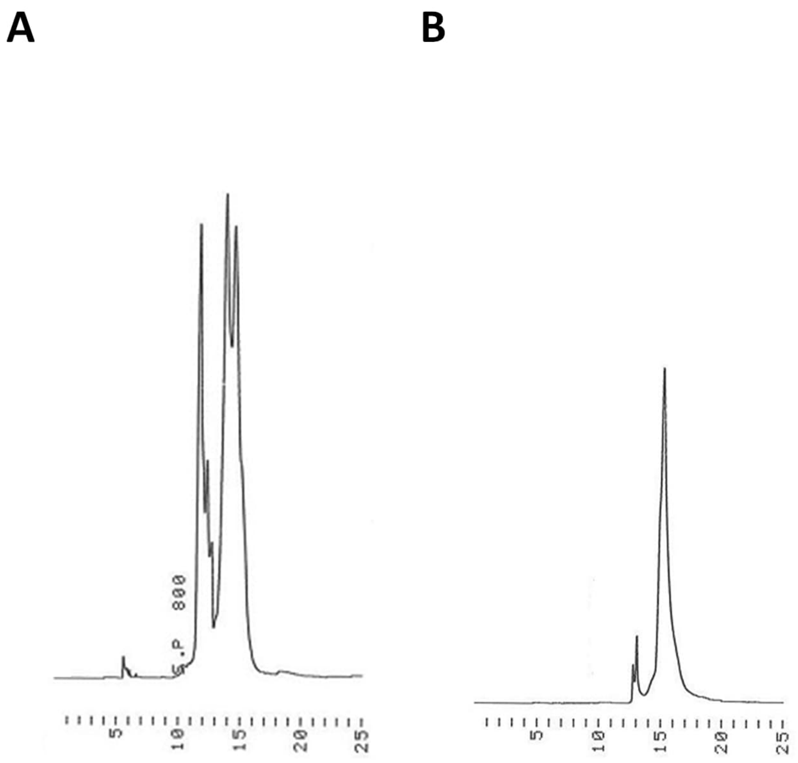

Figure S53. RP-HPLC traces of A) crude product mixture of the synthesis of oligonucleotide ON8s and B) the purified product after treatment with acetic acid; Thermo Scientific ODS Hypersil column (250 × 10 mm, 5 µM); flow rate = 3.0 mL min^{-1}; linear gradient (10—40 % over 25 min) of MeCN in 50 mM aqueous triethylammonium acetate; λ = 260 nm.

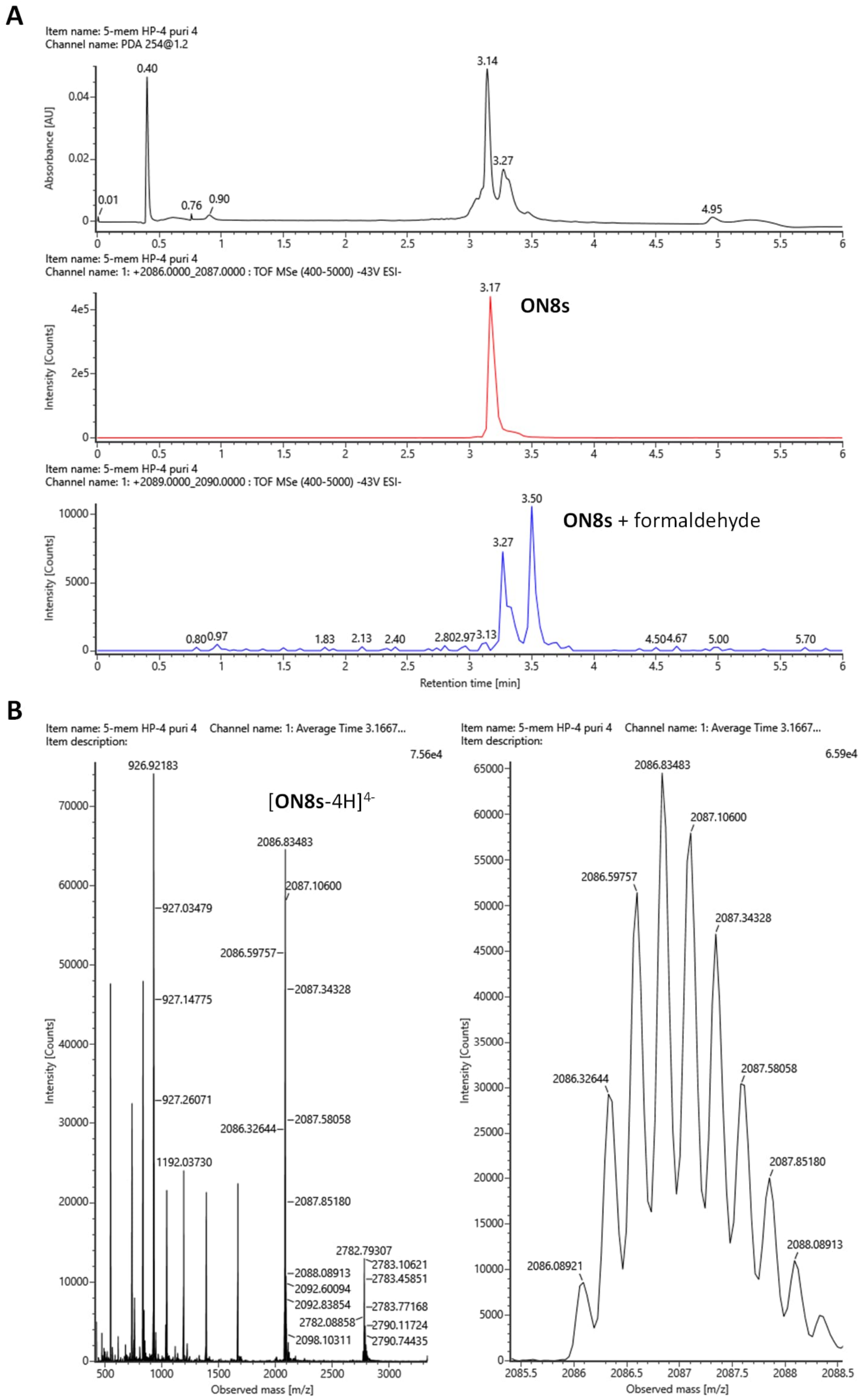

Figure S54. A) UV and extracted ion UPLC traces and B) mass spectra of oligonucleotide ON8s; ACQUITY Premier OST column (50 × 2.1 mm, 1.7 µm); flow rate 0.4 mL min^{-1}; linear gradient (5—25 % over 4 min) of MeOH in aqueous solution of hexafluoroisopropanol (40 mM) and triethylamine (7 mM); λ = 254 nm; T = 60 °C. Besides naked ON8s, a peak for the reversible adduct with formaldehyde was observed.

A B

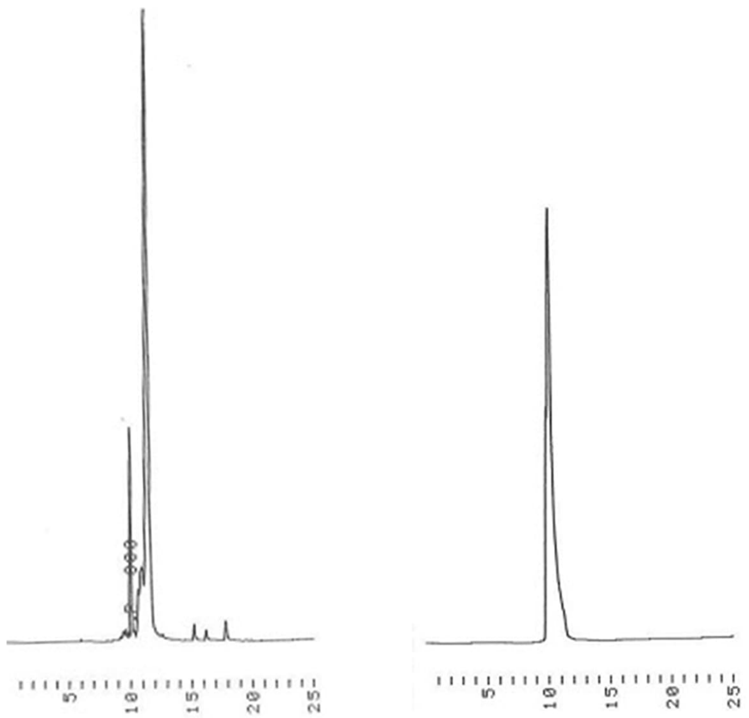

Figure S55. RP-HPLC traces of A) crude product mixture of the synthesis of oligonucleotide ON9 and B) the purified product after treatment with acetic acid; Thermo Scientific ODS Hypersil column (250 × 10 mm, 5 µM); flow rate = 3.0 mL min^{-1}; linear gradient (10—40 % over 25 min) of MeCN in 50 mM aqueous triethylammonium acetate; λ = 260 nm.

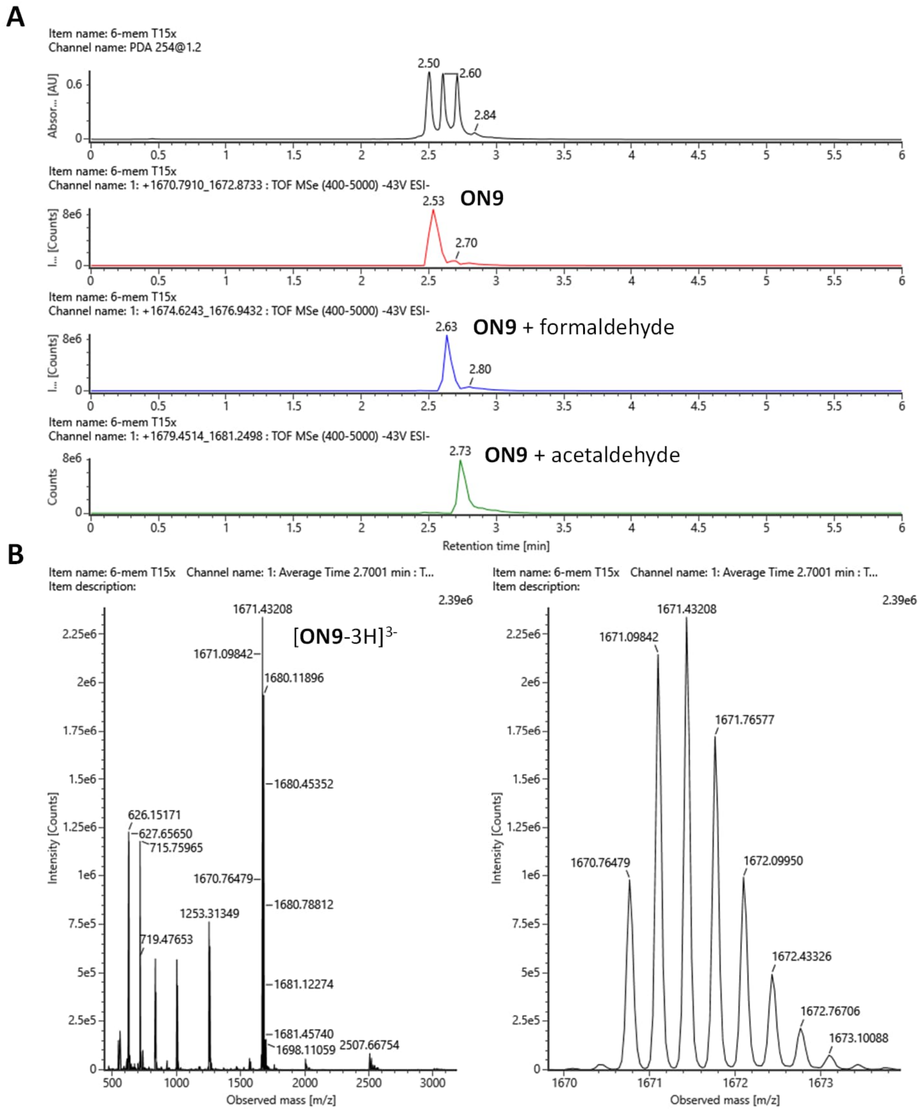

Figure S56. A) UV and extracted ion UPLC traces and B) mass spectra of oligonucleotide ON9; ACQUITY Premier OST column (50 × 2.1 mm, 1.7 µm); flow rate 0.4 mL min^{-1}; linear gradient (5—25 % over 4 min) of MeOH in aqueous solution of hexafluoroisopropanol (40 mM) and triethylamine (7 mM); λ = 254 nm; T = 60 °C. Besides naked ON9, peaks for the reversible adducts with formaldehyde and acetaldehyde were observed.

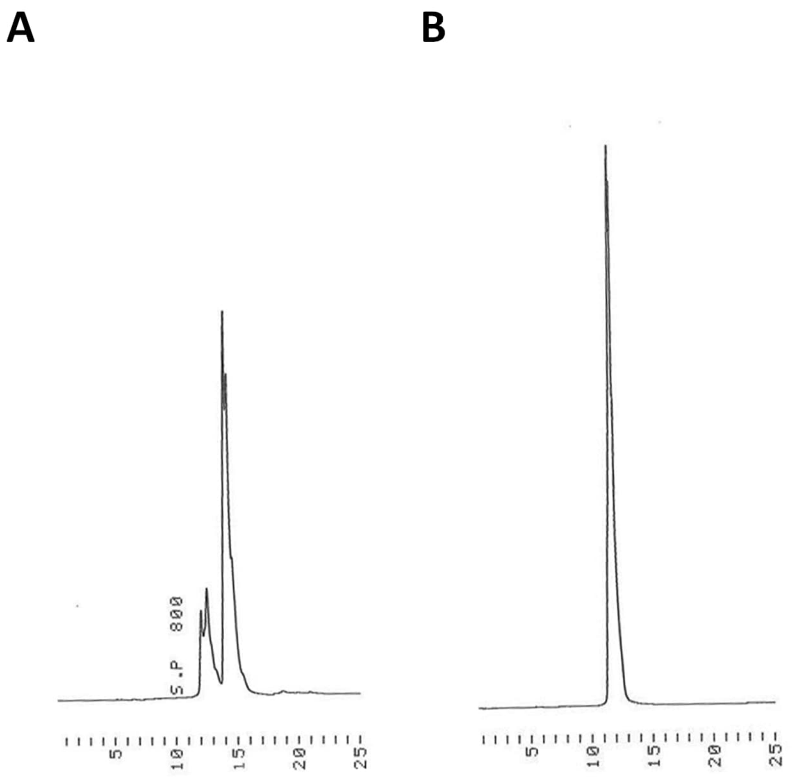

Figure S57. RP-HPLC traces of A) crude product mixture of the synthesis of oligonucleotide ON10 and B) the purified product after treatment with acetic acid; Thermo Scientific ODS Hypersil column (250 × 10 mm, 5 µM); flow rate = 3.0 mL min^{-1}; linear gradient (10—40 % over 25 min) of MeCN in 50 mM aqueous triethylammonium acetate; λ = 260 nm.

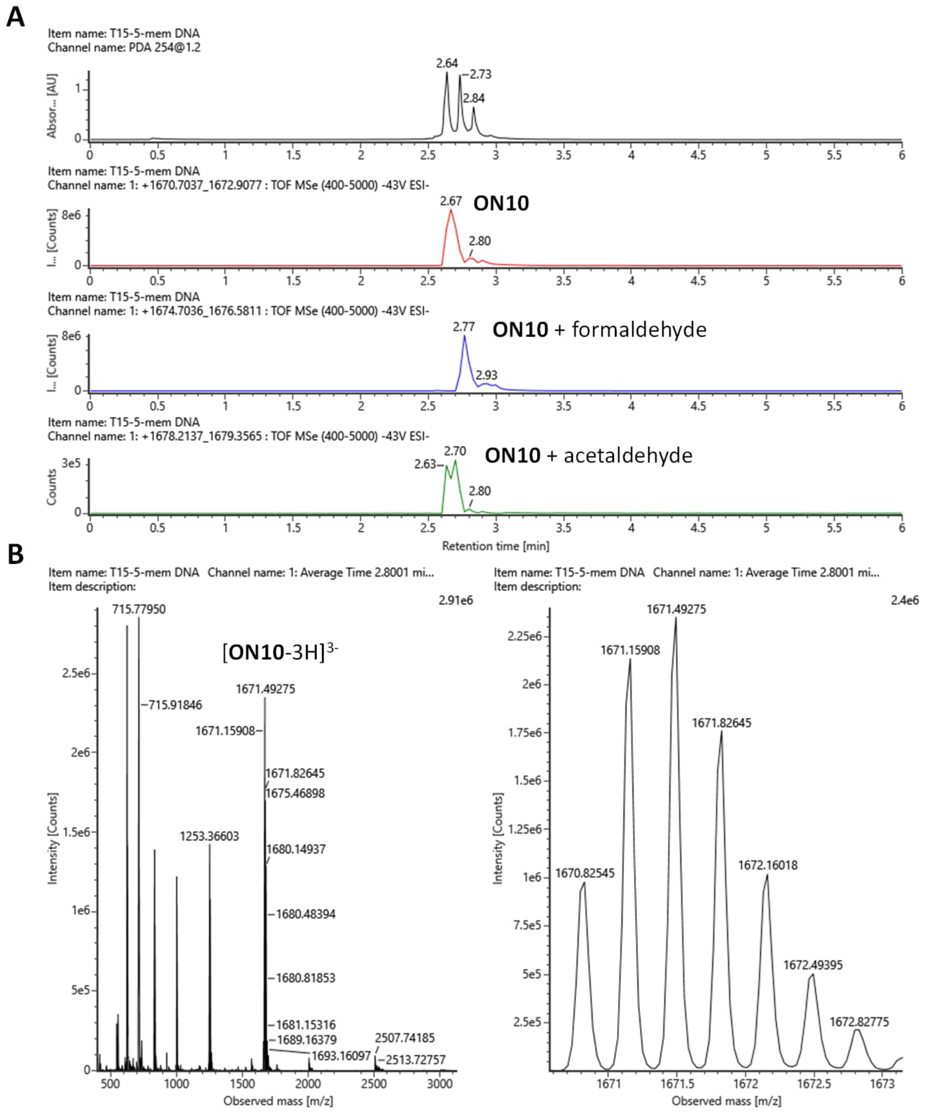

Figure S58. A) UV and extracted ion UPLC traces and B) mass spectra of oligonucleotide ON10; ACQUITY Premier OST column (50 × 2.1 mm, 1.7 µm); flow rate 0.4 mL min^{-1}; linear gradient (5—25 % over 4 min) of MeOH in aqueous solution of hexafluoroisopropanol (40 mM) and triethylamine (7 mM); λ = 254 nm; T = 60 °C. Besides naked ON10, peaks for the reversible adducts with formaldehyde and acetaldehyde were observed.

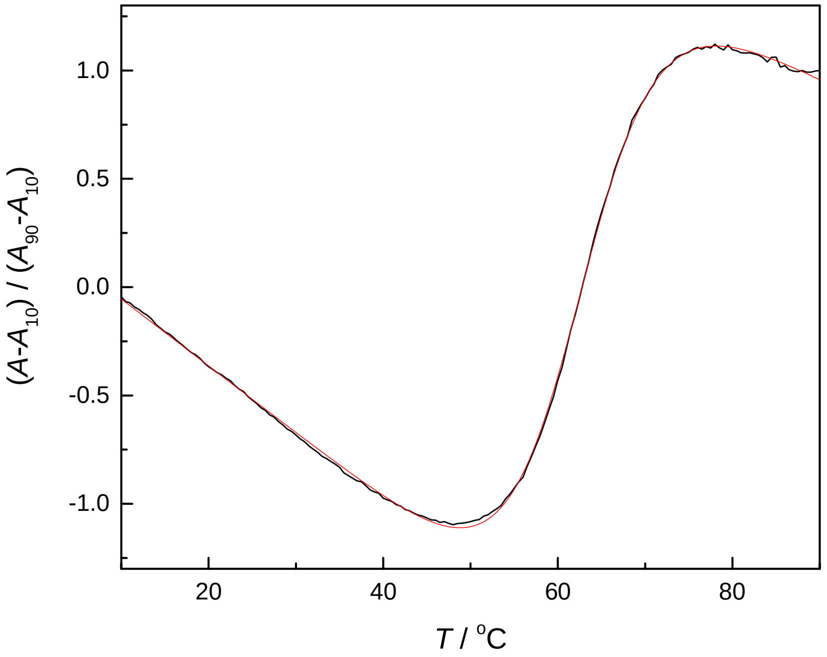

Figure S59. UV melting profile of 1.0 µM hairpin ON4a; pH = 5.5 (20 mM cacodylate buffer); $I(NaClO_4)$ = 0.10.

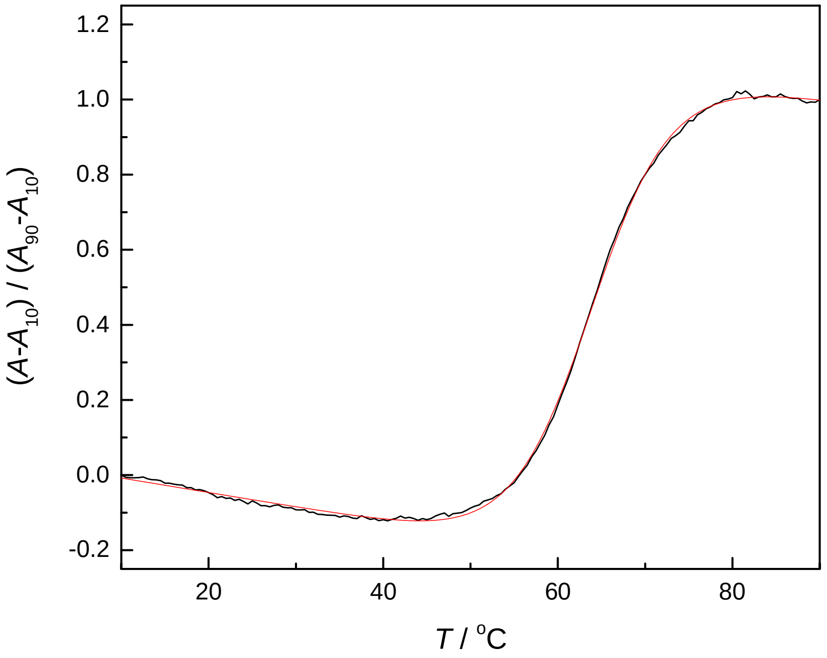

Figure S60. UV melting profile of 1.0 µM hairpin ON4c; pH = 5.5 (20 mM cacodylate buffer); $I(NaClO_4)$ = 0.10.

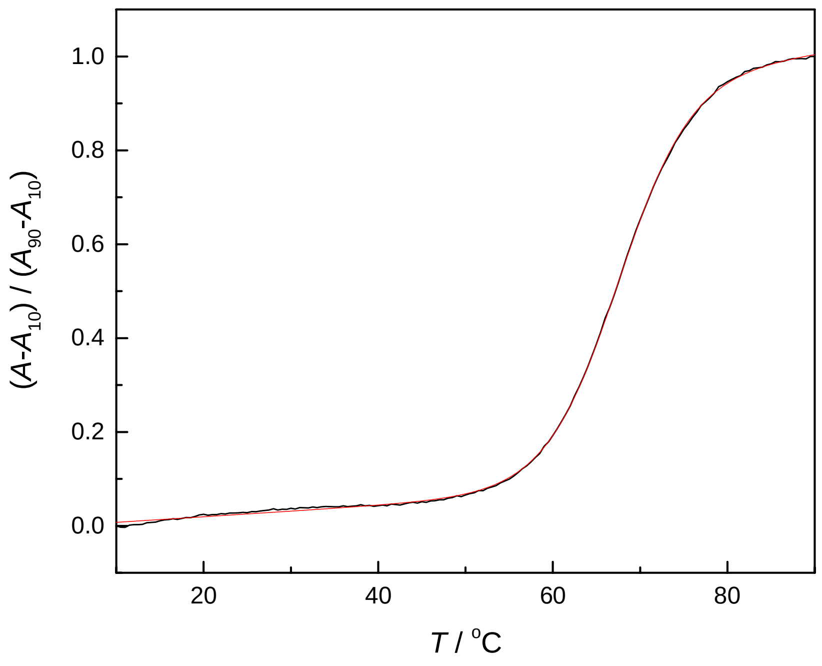

Figure S61. UV melting profile of 1.0 µM hairpin ON4g; pH = 5.5 (20 mM cacodylate buffer); $I(NaClO_4)$ = 0.10.

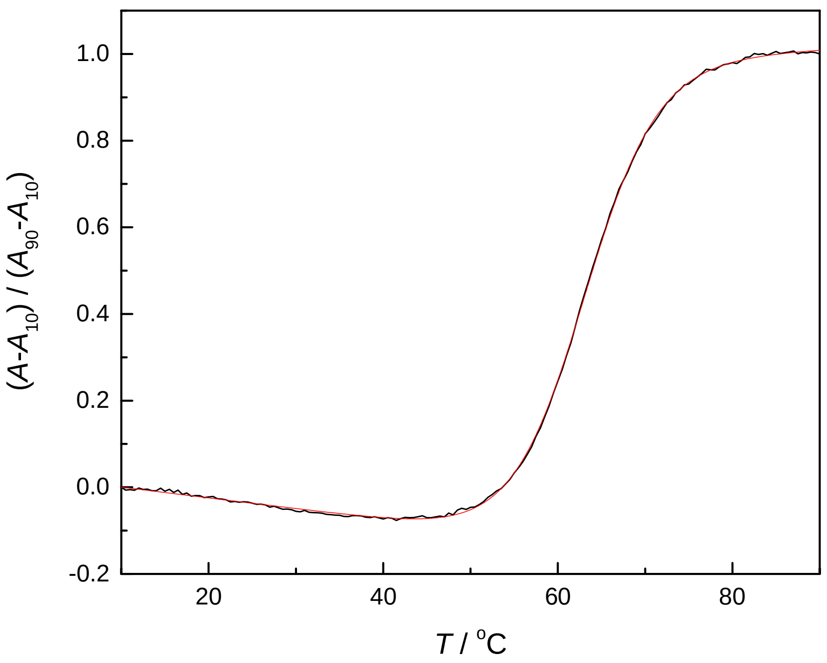

Figure S62. UV melting profile of 1.0 µM hairpin ON4t; pH = 5.5 (20 mM cacodylate buffer); $I(NaClO_4)$ = 0.10.

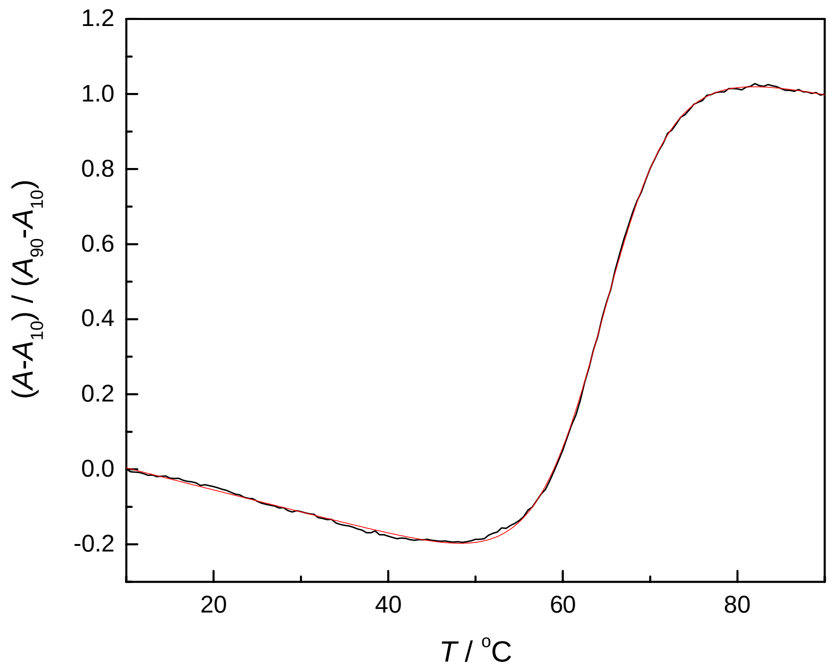

Figure S63. UV melting profile of 1.0 µM hairpin ON4s; pH = 5.5 (20 mM cacodylate buffer); *I*($NaClO_4$) = 0.10.

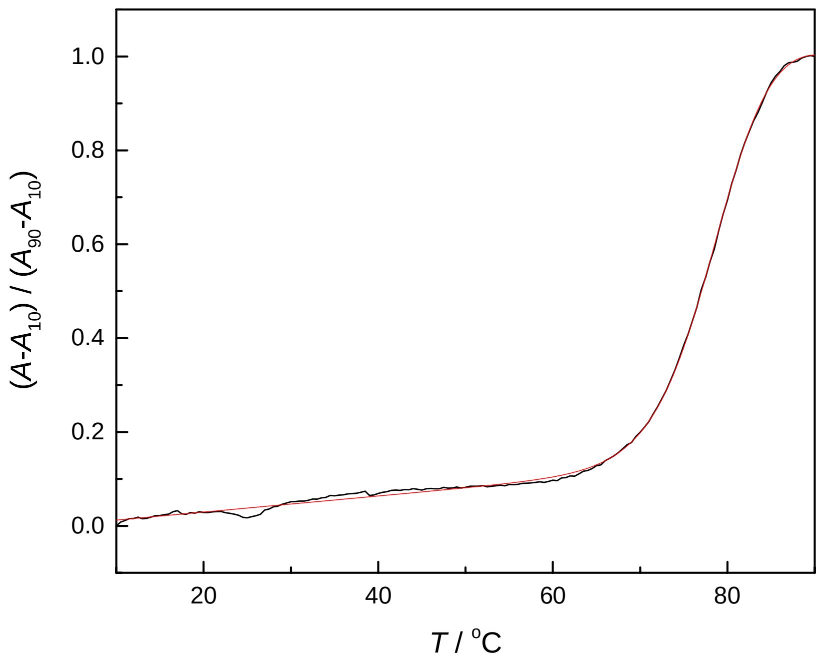

Figure S64. UV melting profile of 1.0 µM hairpin ON6a; pH = 5.5 (20 mM cacodylate buffer); *I*($NaClO_4$) = 0.10.

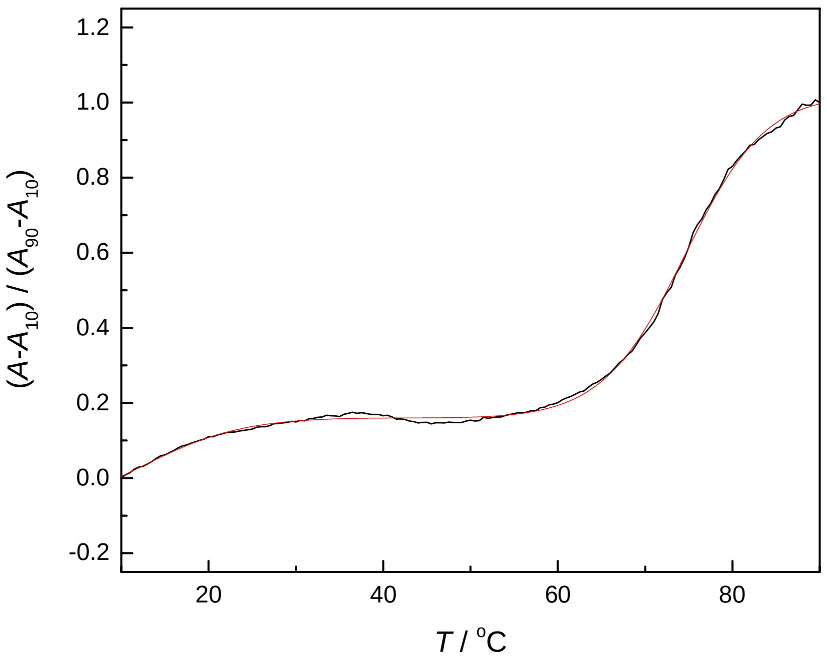

Figure S65. UV melting profile of 1.0 µM hairpin ON6c; pH = 5.5 (20 mM cacodylate buffer); *I*($NaClO_4$) = 0.10.

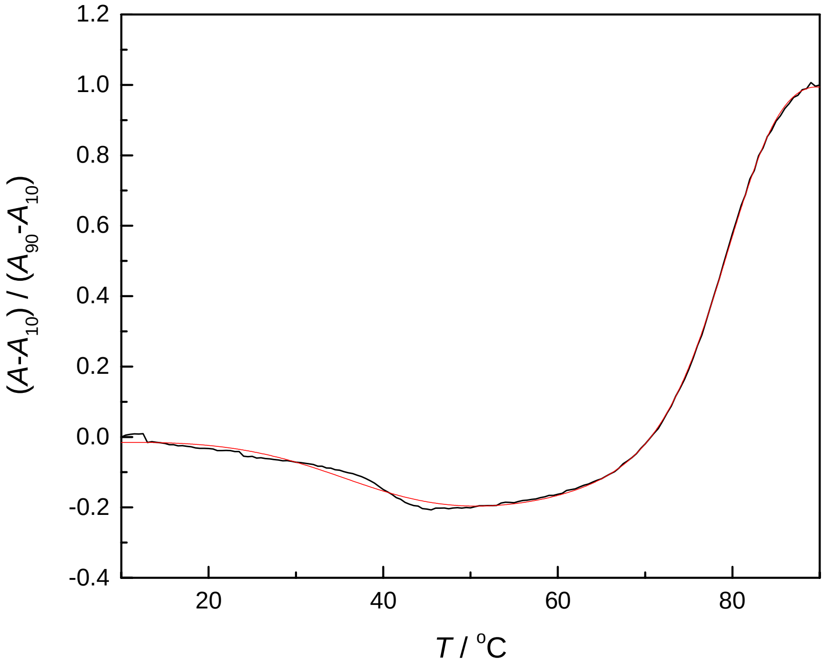

Figure S66. UV melting profile of 1.0 µM hairpin ON6g; pH = 5.5 (20 mM cacodylate buffer); *I*($NaClO_4$) = 0.10.

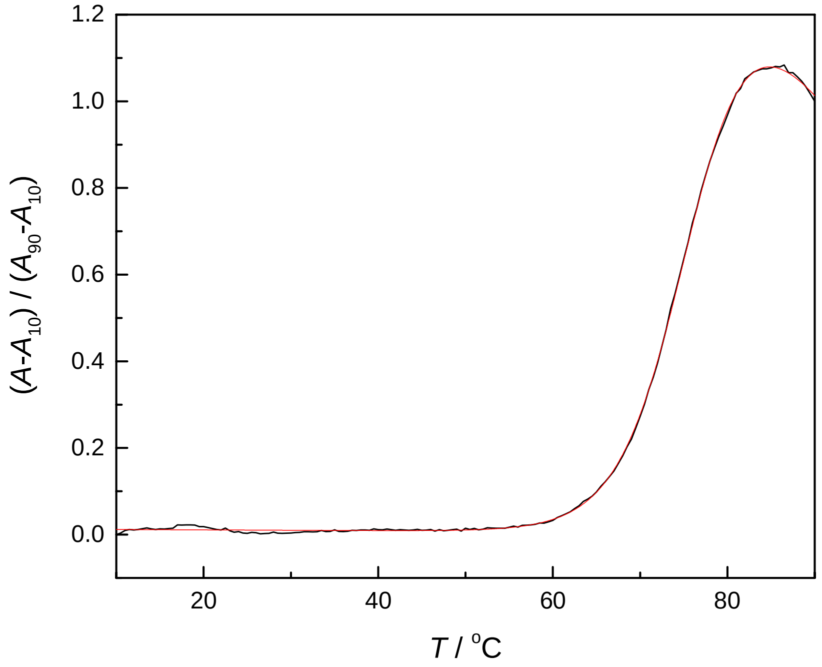

Figure S67. UV melting profile of 1.0 µM hairpin ON6u; pH = 5.5 (20 mM cacodylate buffer); *I*($NaClO_4$) = 0.10.

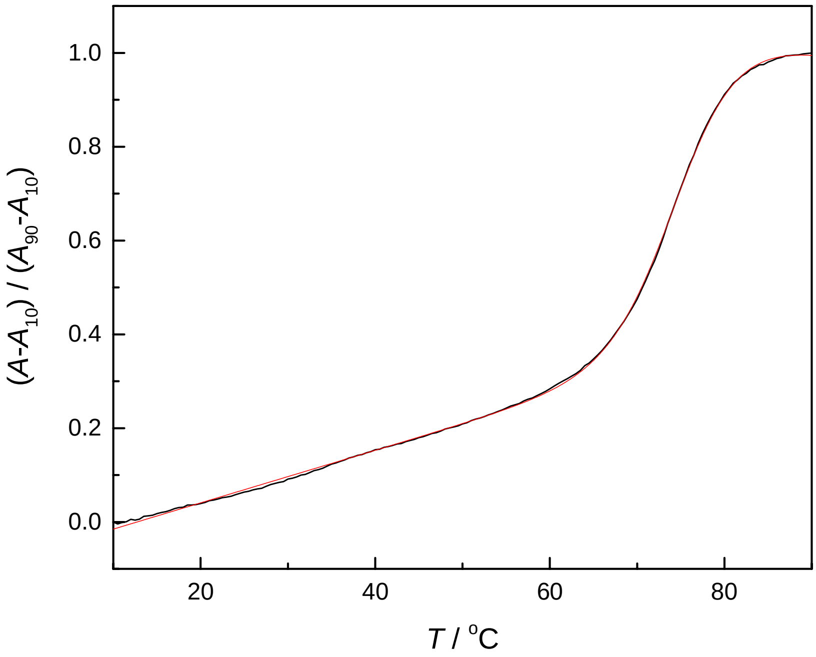

Figure S68. UV melting profile of 1.0 µM hairpin ON6s; pH = 5.5 (20 mM cacodylate buffer); *I*($NaClO_4$) = 0.10.

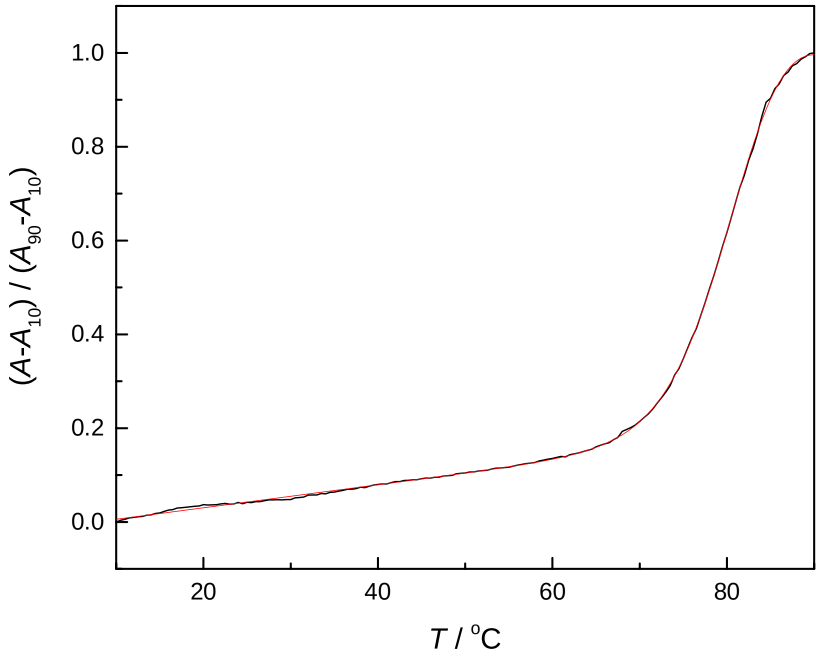

Figure S69. UV melting profile of 1.0 µM hairpin ON8a; pH = 5.5 (20 mM cacodylate buffer); *I*($NaClO_4$) = 0.10.

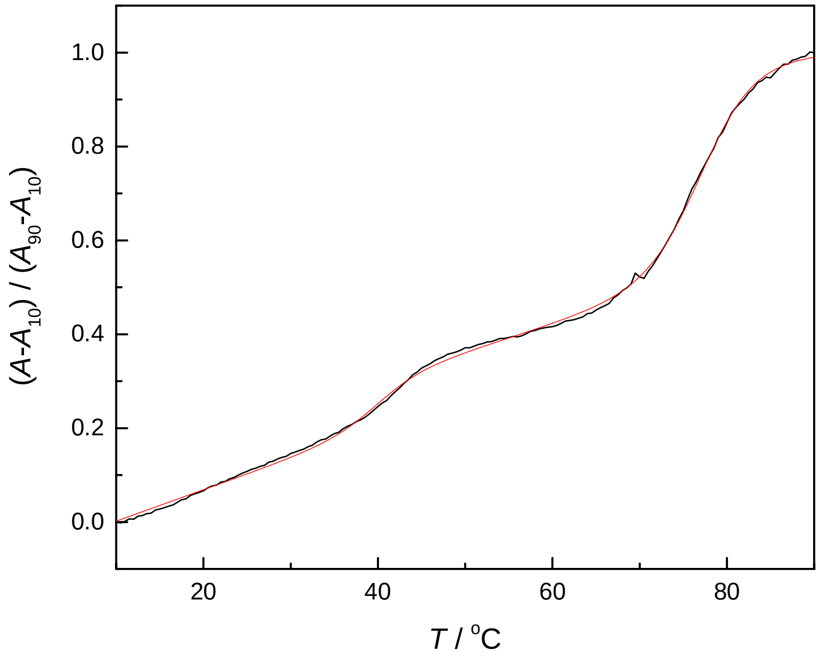

Figure S70. UV melting profile of 1.0 µM hairpin ON8c; pH = 5.5 (20 mM cacodylate buffer); *I*($NaClO_4$) = 0.10.

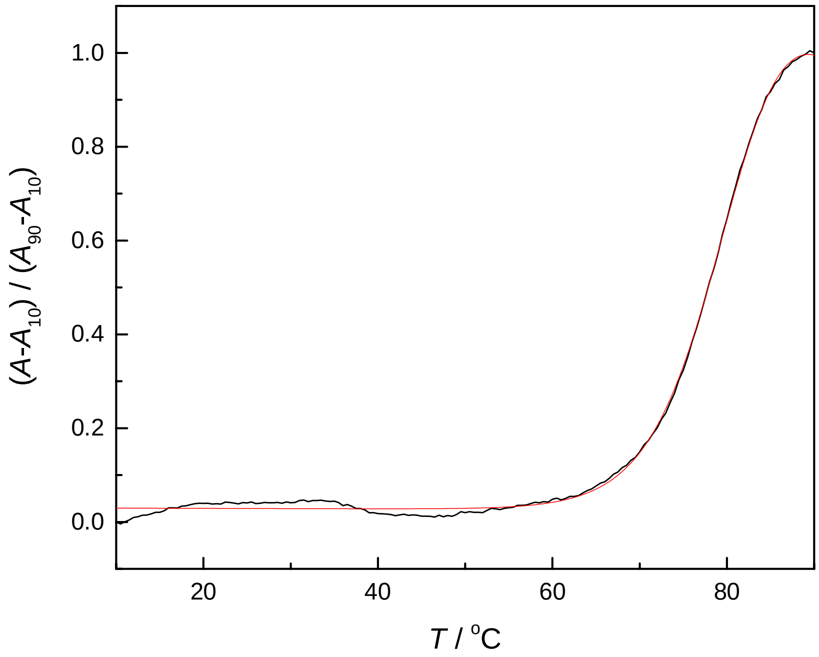

Figure S71. UV melting profile of 1.0 μM hairpin ON8g; pH = 5.5 (20 mM cacodylate buffer); *I*($NaClO_4$) = 0.10.

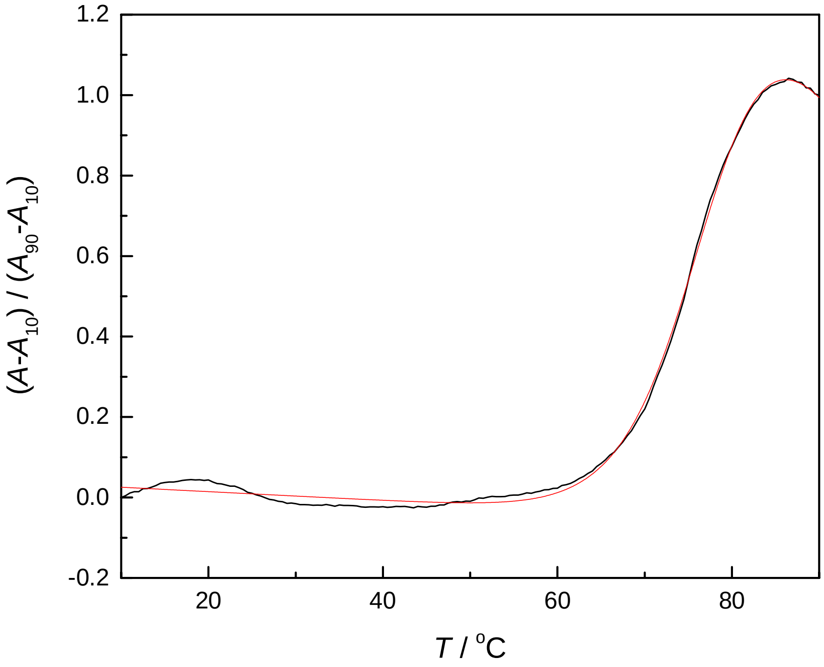

Figure S72. UV melting profile of 1.0 μM hairpin ON8u; pH = 5.5 (20 mM cacodylate buffer); *I*($NaClO_4$) = 0.10.

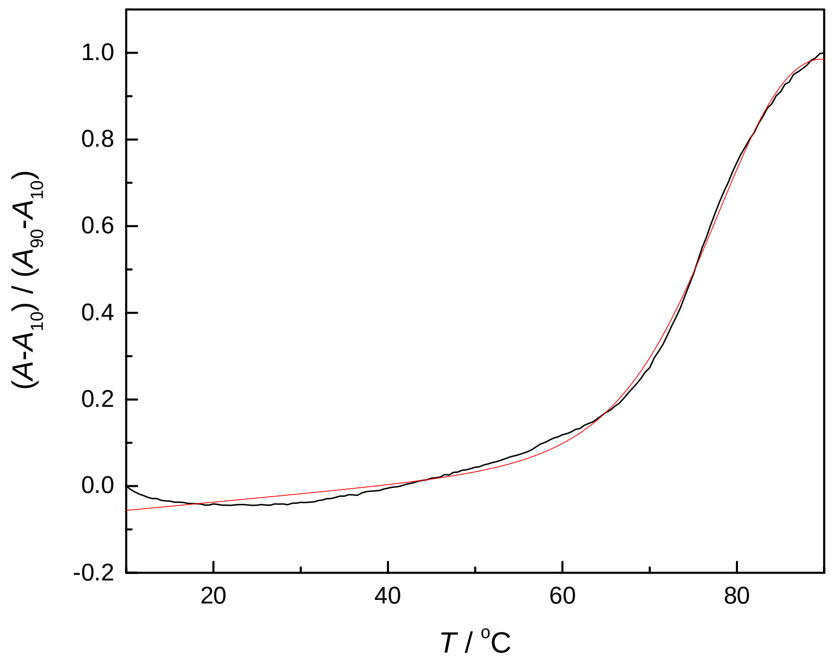

Figure S73. UV melting profile of 1.0 µM hairpin ON8s; pH = 5.5 (20 mM cacodylate buffer); *I*($NaClO_4$) = 0.10.

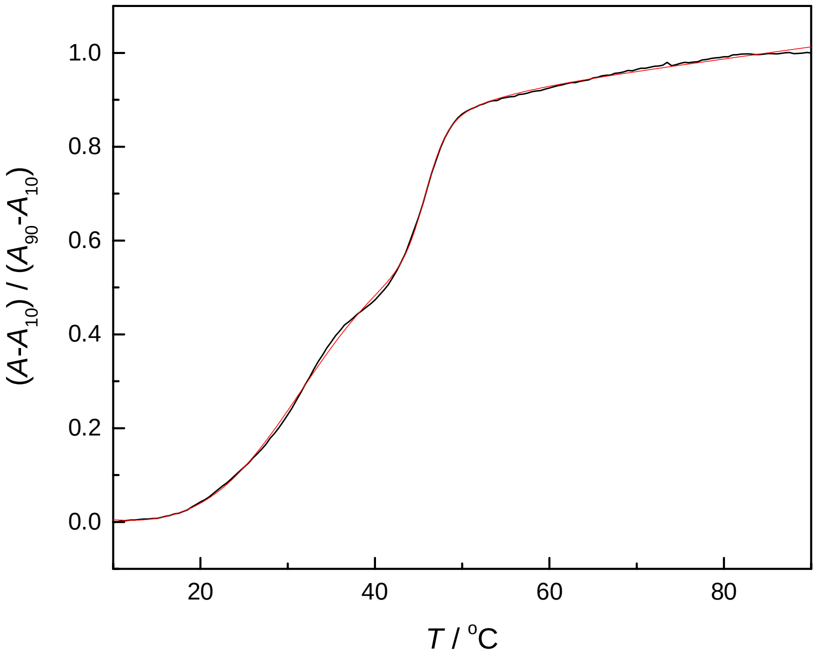

Figure S74. UV melting profile of 1.0 µM triplex ON11t•ON12a*ON9; pH = 5.5 (20 mM cacodylate buffer); $I(NaClO_4)$ = 0.10.

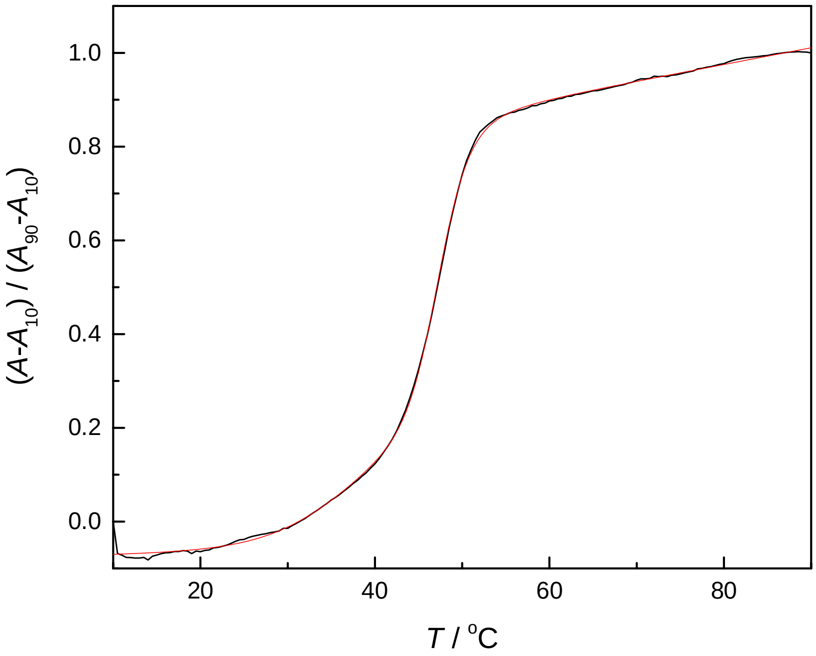

Figure S75. UV melting profile of 1.0 µM triplex ON11g•ON12c*ON9; pH = 5.5 (20 mM cacodylate buffer); $I(NaClO_4)$ = 0.10.

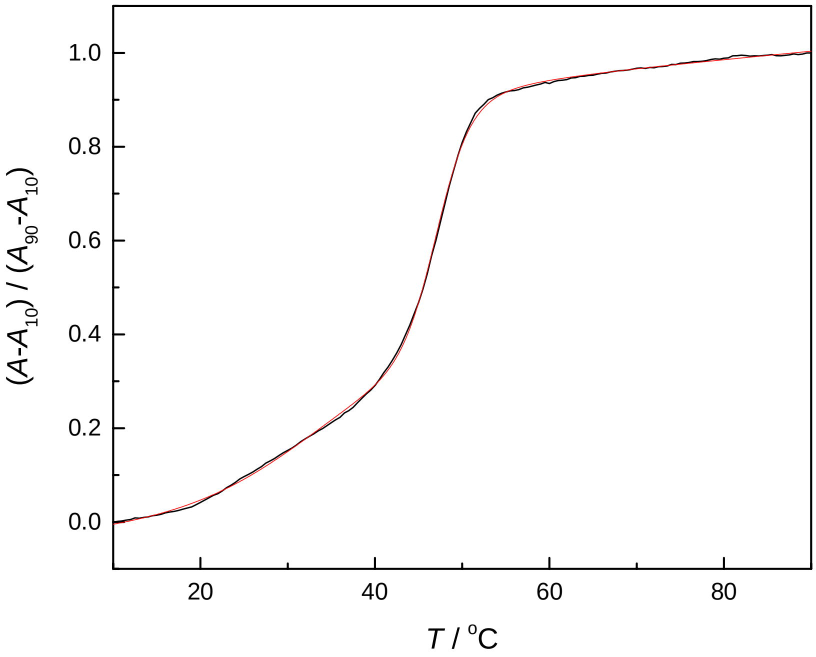

Figure S76. UV melting profile of 1.0 µM triplex ON11c•ON12g*ON9; pH = 5.5 (20 mM cacodylate buffer); *I*($NaClO_4$) = 0.10.

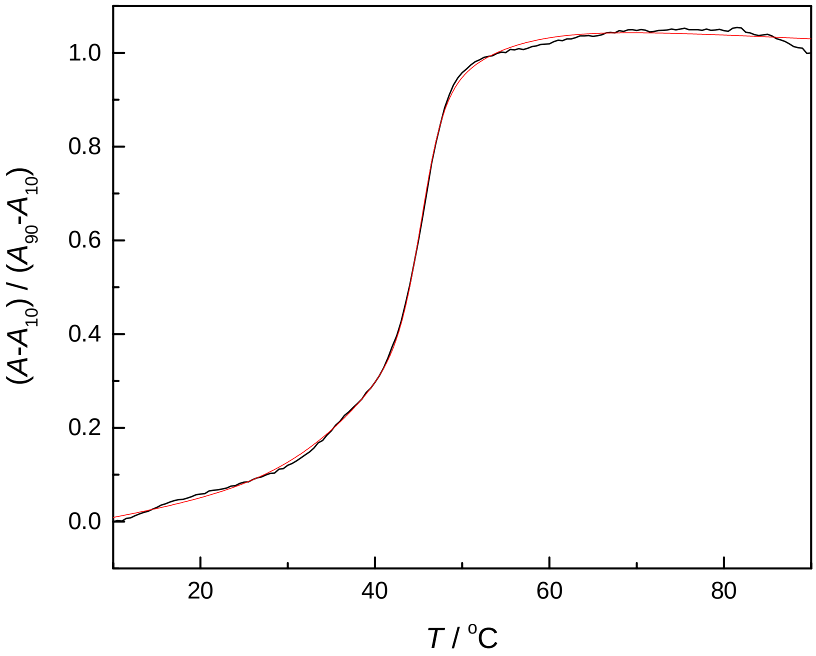

Figure S77. UV melting profile of 1.0 µM triplex ON11a•ON12t*ON9; pH = 5.5 (20 mM cacodylate buffer); *I*($NaClO_4$) = 0.10.

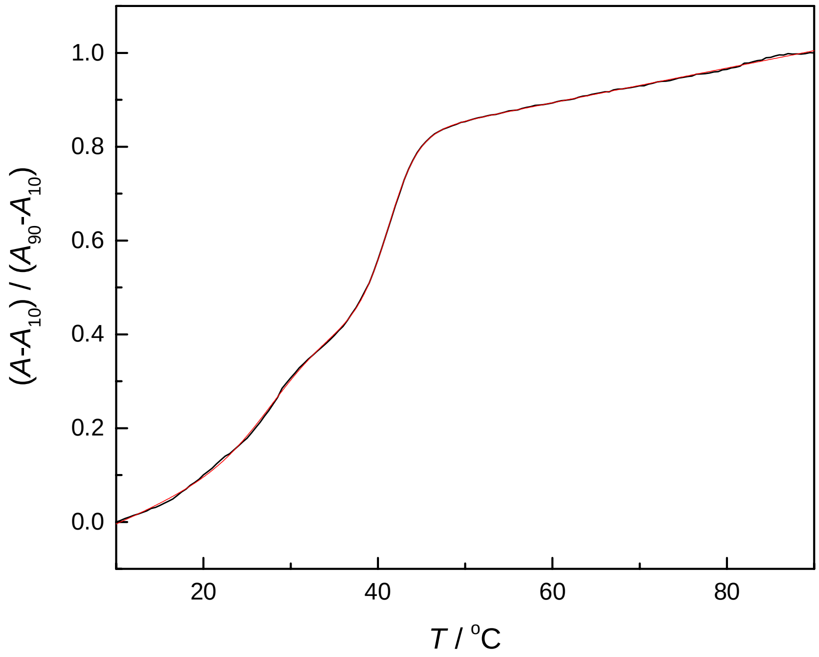

Figure S78. UV melting profile of 1.0 μM triplex ON11t•ON12a*ON10; pH = 5.5 (20 mM cacodylate buffer); *I*($NaClO_4$) = 0.10.

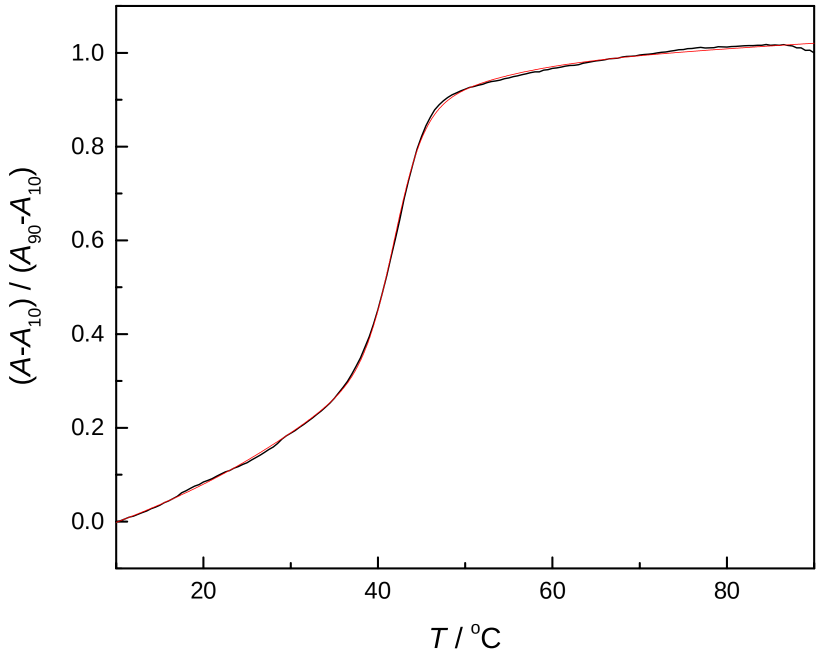

Figure S79. UV melting profile of 1.0 μM triplex ON11g•ON12c*ON10; pH = 5.5 (20 mM cacodylate buffer); *I*($NaClO_4$) = 0.10.

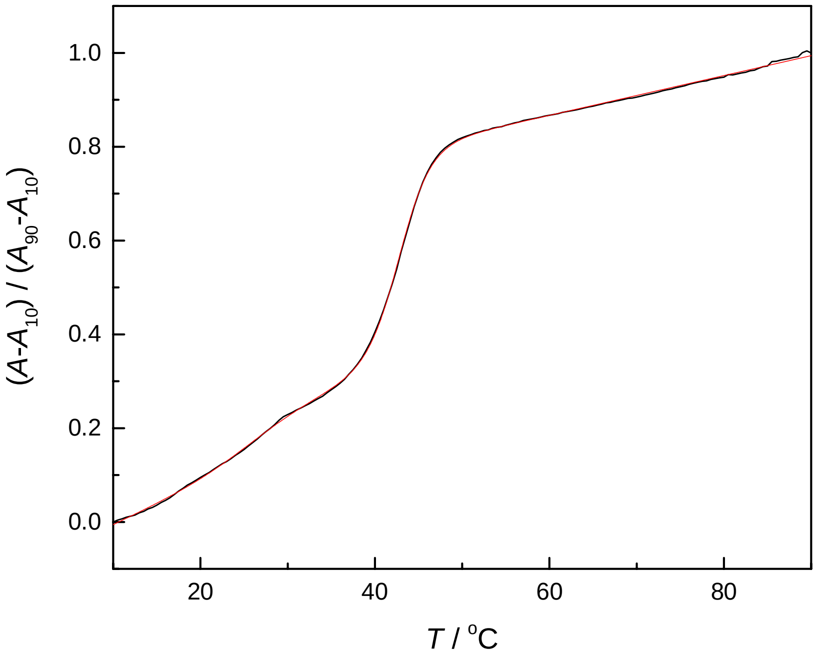

Figure S80. UV melting profile of 1.0 μM triplex ON11c•ON12g*ON10; pH = 5.5 (20 mM cacodylate buffer); *I*($NaClO_4$) = 0.10.

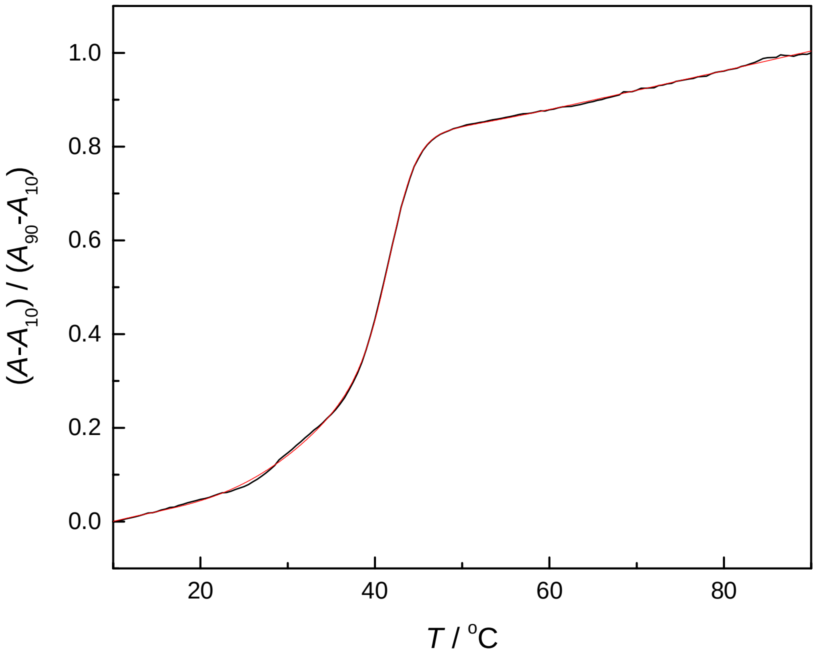

Figure S81. UV melting profile of 1.0 μM triplex ON11a•ON12t*ON10; pH = 5.5 (20 mM cacodylate buffer); *I*($NaClO_4$) = 0.10.

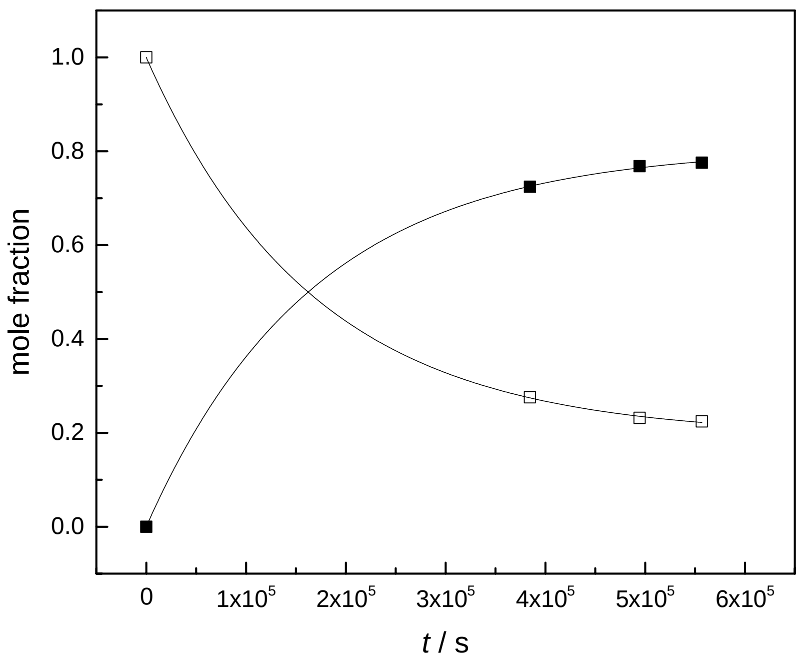

Figure S82. Time-dependent mole fraction of oligonucleotide ON2t (□) and its 9-formylmethyladenine adduct (■); pH = 5.5 (20 mM cacodylate buffer); *I*($NaClO_4$) = 0.10; [oligonucleotides] = 1.0 µM; [aldehydes] = 2.0 µM.

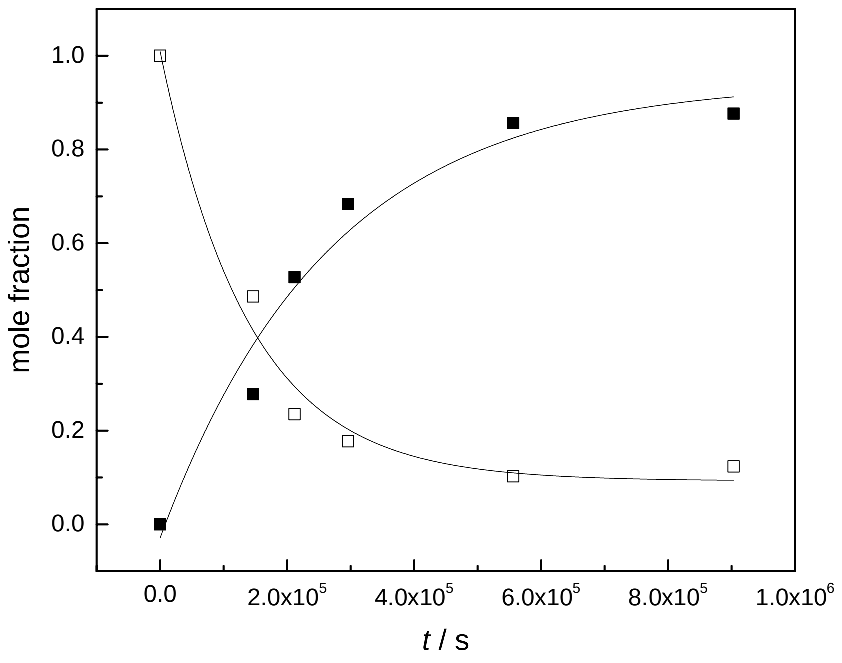

Figure S83. Time-dependent mole fraction of oligonucleotide ON4t (□) and its 9-formylmethyladenine adduct (■); pH = 5.5 (20 mM cacodylate buffer); *I*($NaClO_4$) = 0.10; [oligonucleotides] = 1.0 µM; [aldehydes] = 2.0 µM.

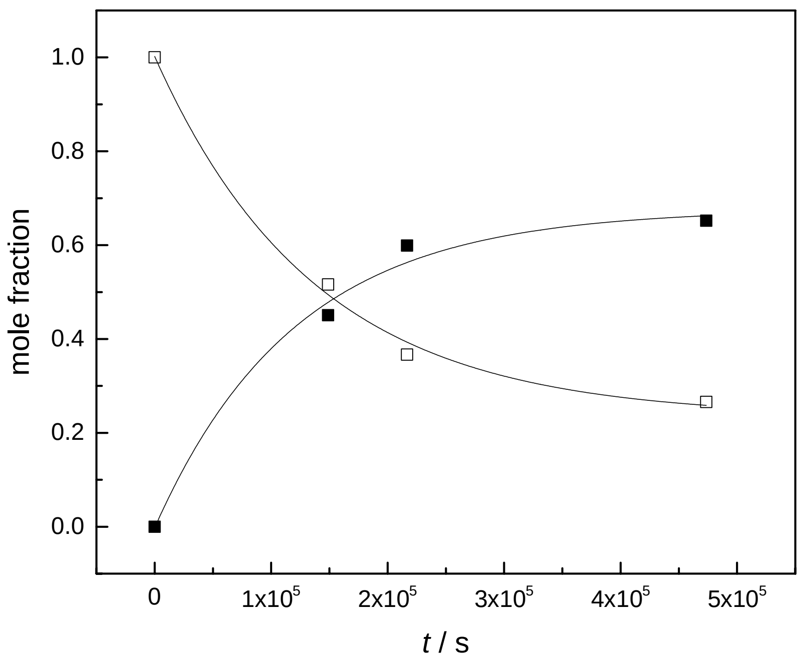

Figure S84. Time-dependent mole fraction of oligonucleotide ON6u (□) and its 9-formylmethyladenine adduct (■); pH = 5.5 (20 mM cacodylate buffer); $I(NaClO_4)$ = 0.10; [oligonucleotides] = 1.0 µM; [aldehydes] = 2.0 µM.

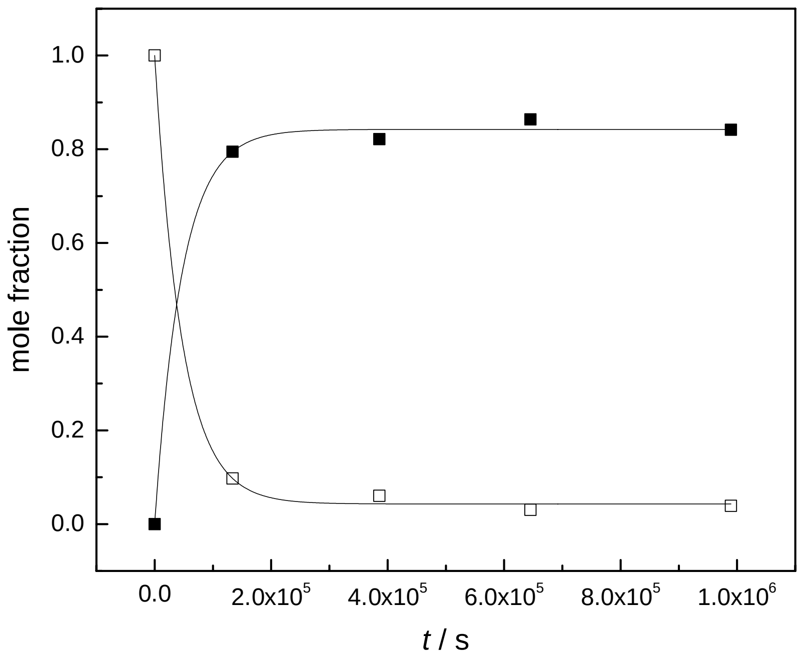

Figure S85. Time-dependent mole fraction of oligonucleotide ON8u (□) and its 9-formylmethyladenine adduct (■); pH = 5.5 (20 mM cacodylate buffer); $I(NaClO_4)$ = 0.10; [oligonucleotides] = 1.0 µM; [aldehydes] = 2.0 µM.

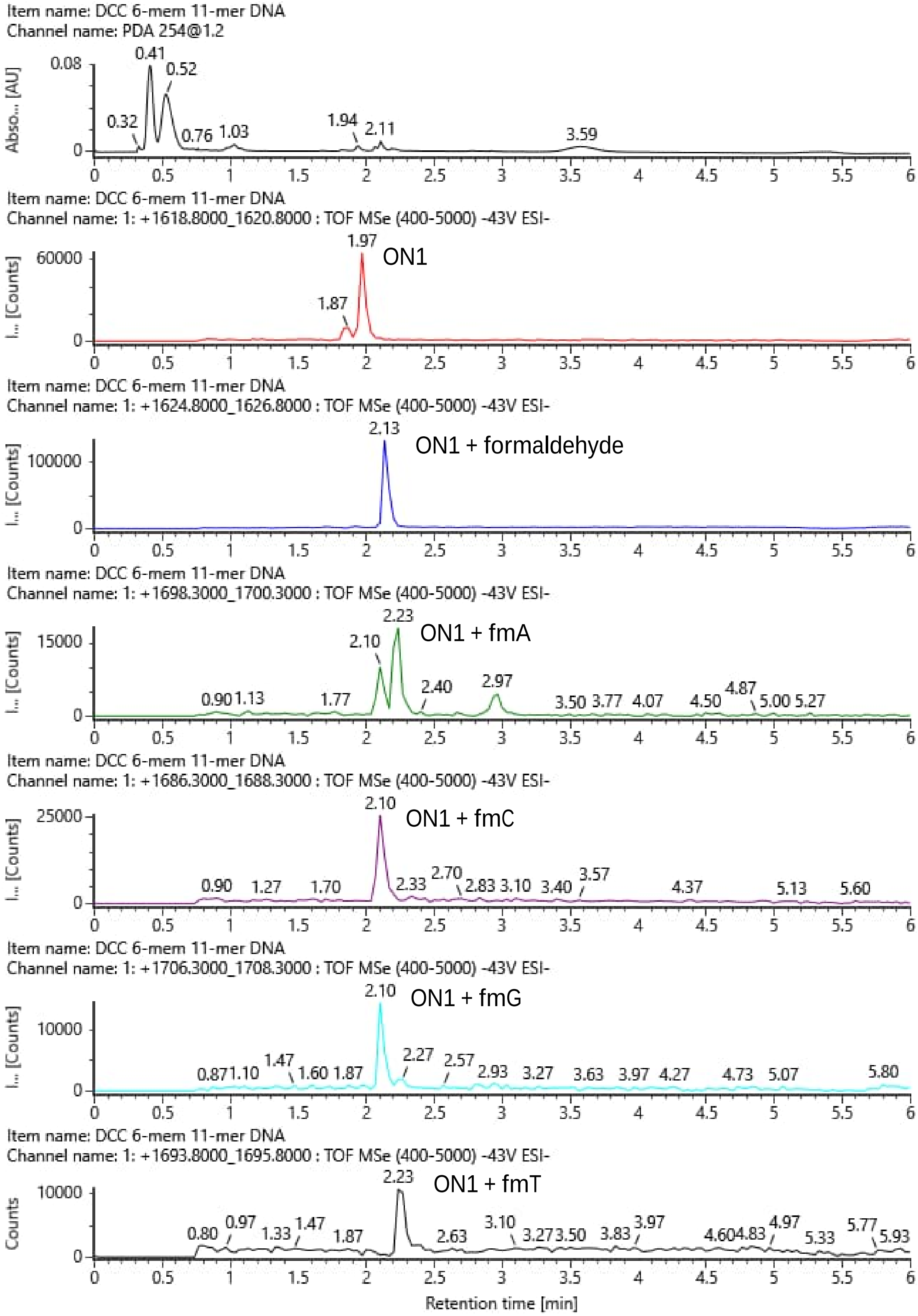

Figure S86. UV (top panel) and extracted ion RP-UPLC traces of the DCC product mixture on incubation of the single-stranded oligonucleotide ON1 (1.0 µM) with a mixture of aldehydes fmA, fmC, fmG, fmT and fmB (20 µM) at 23 °C, pH = 5.5 (20 mM cacodylate buffer) and $I(NaClO_4)$ = 0.10 M for 120 h; ACQUITY Premier OST column (50 × 2.1 mm, 1.7 µm); flow rate 0.4 mL min^{-1}; linear gradient (5—25% over 4 min) of MeOH in aqueous solution of hexafluoroisopropanol (40 mM) and triethylamine (7 mM); λ = 254 nm; T = 60 °C.

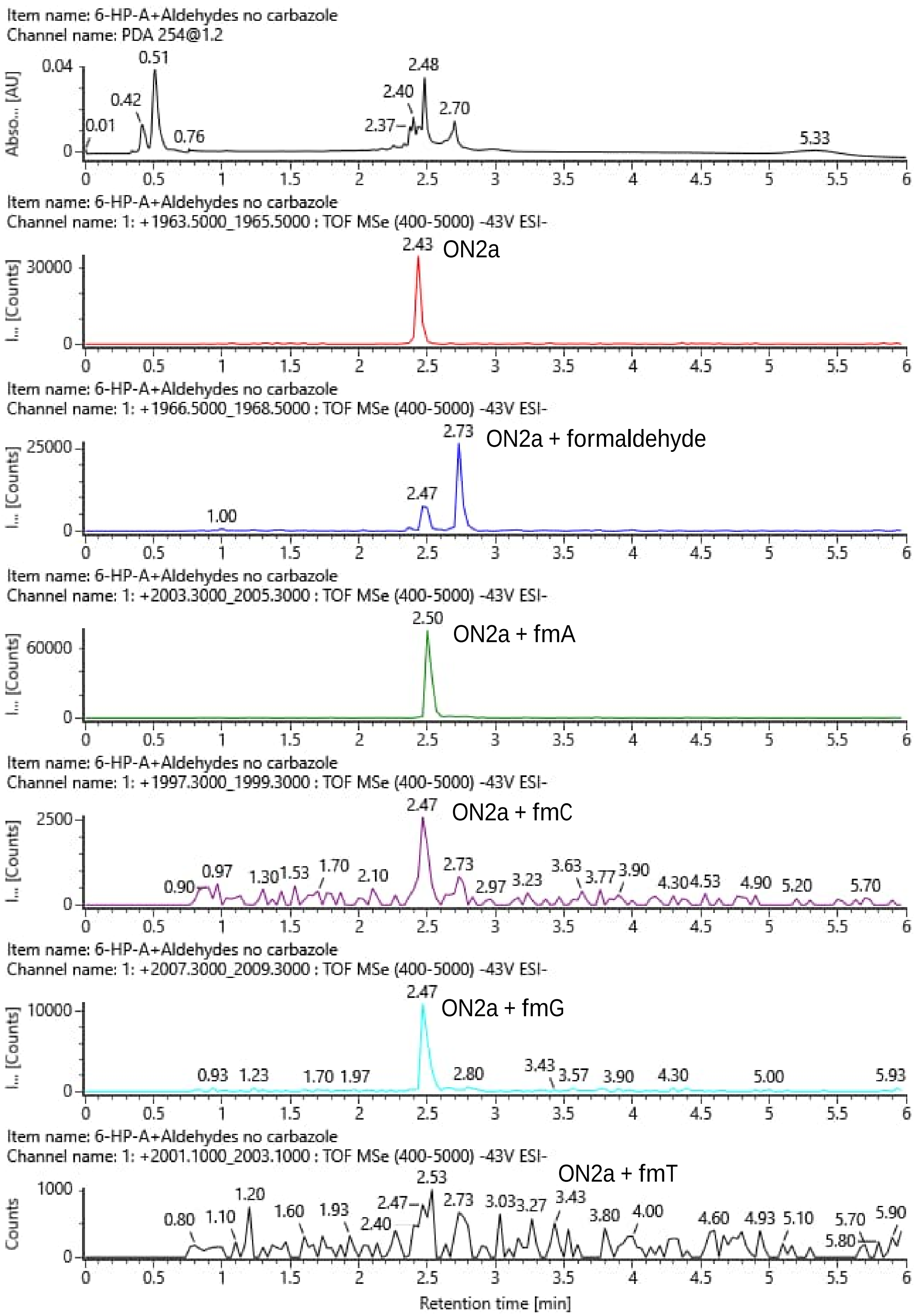

Figure S87. UV (top panel) and extracted ion RP-UPLC traces of the DCC product mixture on incubation of the hairpin oligonucleotide ON2a (1.0 µM) with a mixture of aldehydes fmA, fmC, fmG, fmT and fmB (2.0 µM) at 23 °C, pH = 5.5 (20 mM cacodylate buffer) and $I(NaClO_4)$ = 0.10 M for 120 h; ACQUITY Premier OST column (50 × 2.1 mm, 1.7 µm); flow rate 0.4 mL min^{-1}; linear gradient (5—25% over 4 min) of MeOH in aqueous solution of hexafluoroisopropanol (40 mM) and triethylamine (7 mM); λ = 254 nm; T = 60 °C.

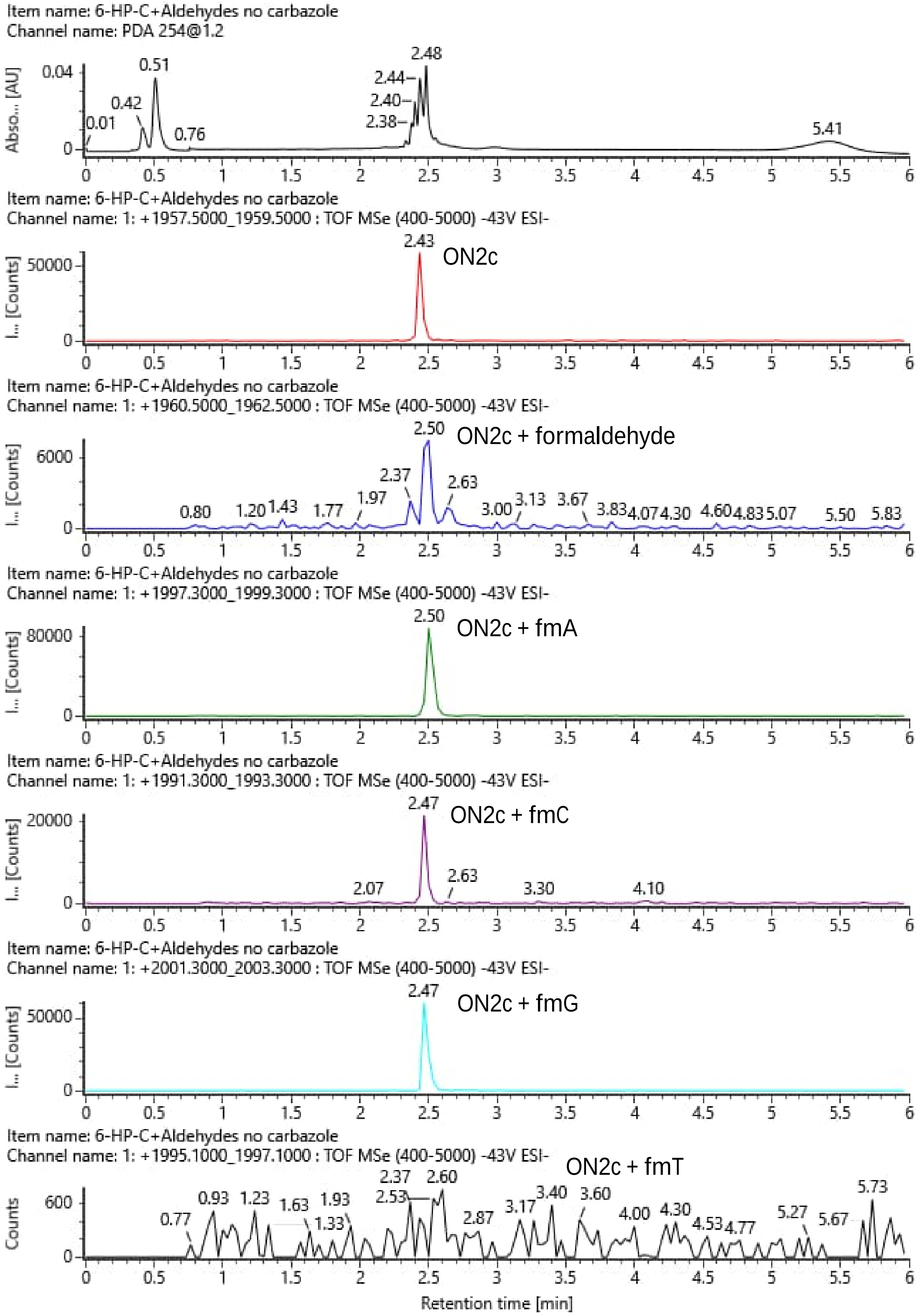

Figure S88. UV (top panel) and extracted ion RP-UPLC traces of the DCC product mixture on incubation of the hairpin oligonucleotide ON2c (1.0 µM) with a mixture of aldehydes fmA, fmC, fmG, fmT and fmB (2.0 µM) at 23 °C, pH = 5.5 (20 mM cacodylate buffer) and $I(NaClO_4)$ = 0.10 M for 120 h; ACQUITY Premier OST column (50 × 2.1 mm, 1.7 µm); flow rate 0.4 mL min^{-1}; linear gradient (5—25% over 4 min) of MeOH in aqueous solution of hexafluoroisopropanol (40 mM) and triethylamine (7 mM); λ = 254 nm; T = 60 °C.

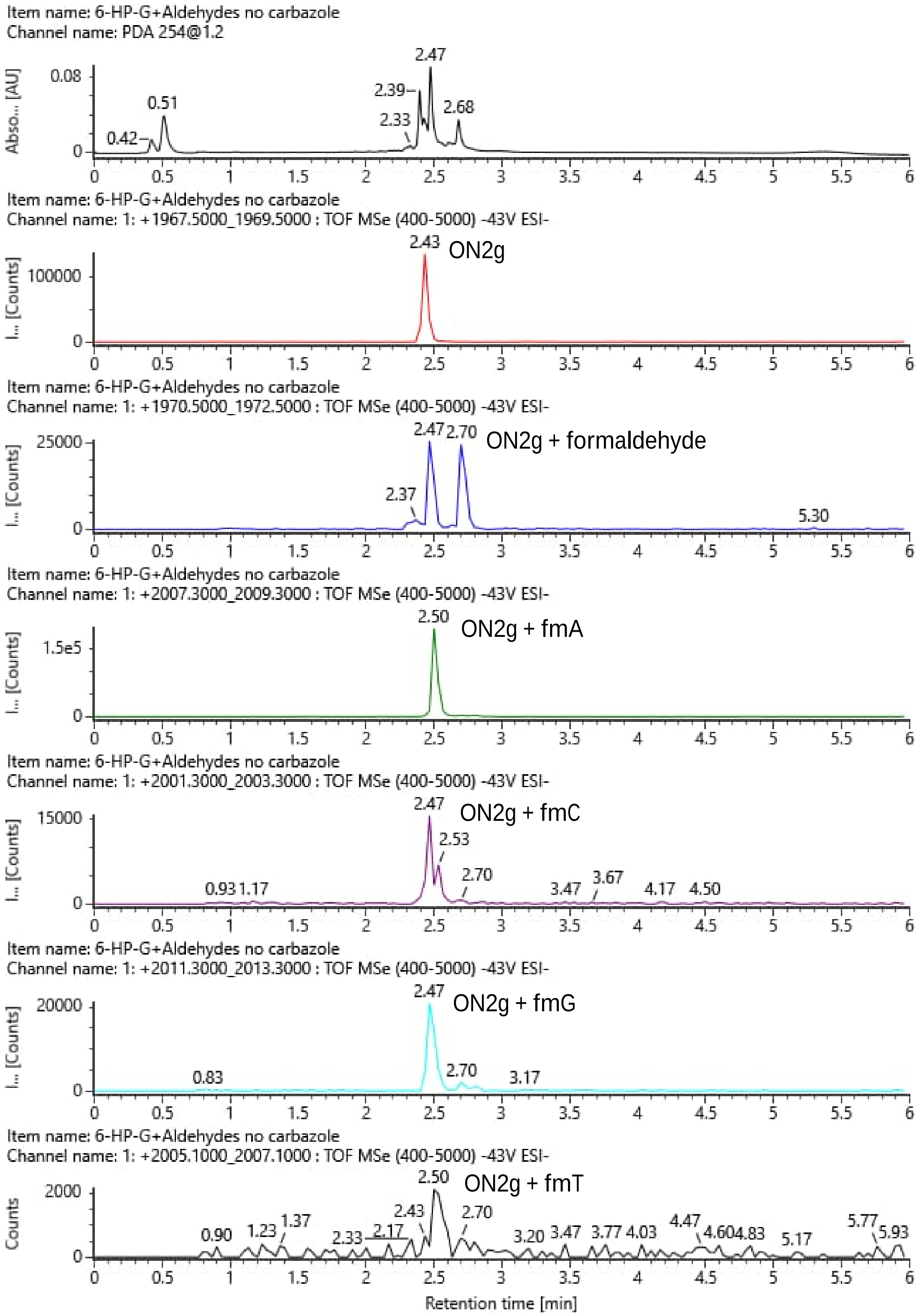

Figure S89. UV (top panel) and extracted ion RP-UPLC traces of the DCC product mixture on incubation of the hairpin oligonucleotide ON2g (1.0 µM) with a mixture of aldehydes fmA, fmC, fmG, fmT and fmB (2.0 µM) at 23 °C, pH = 5.5 (20 mM cacodylate buffer) and *I*($NaClO_4$) = 0.10 M for 120 h; ACQUITY Premier OST column (50 × 2.1 mm, 1.7 µm); flow rate 0.4 mL min^{-1}; linear gradient (5—25% over 4 min) of MeOH in aqueous solution of hexafluoroisopropanol (40 mM) and triethylamine (7 mM); λ = 254 nm; *T* = 60 °C.

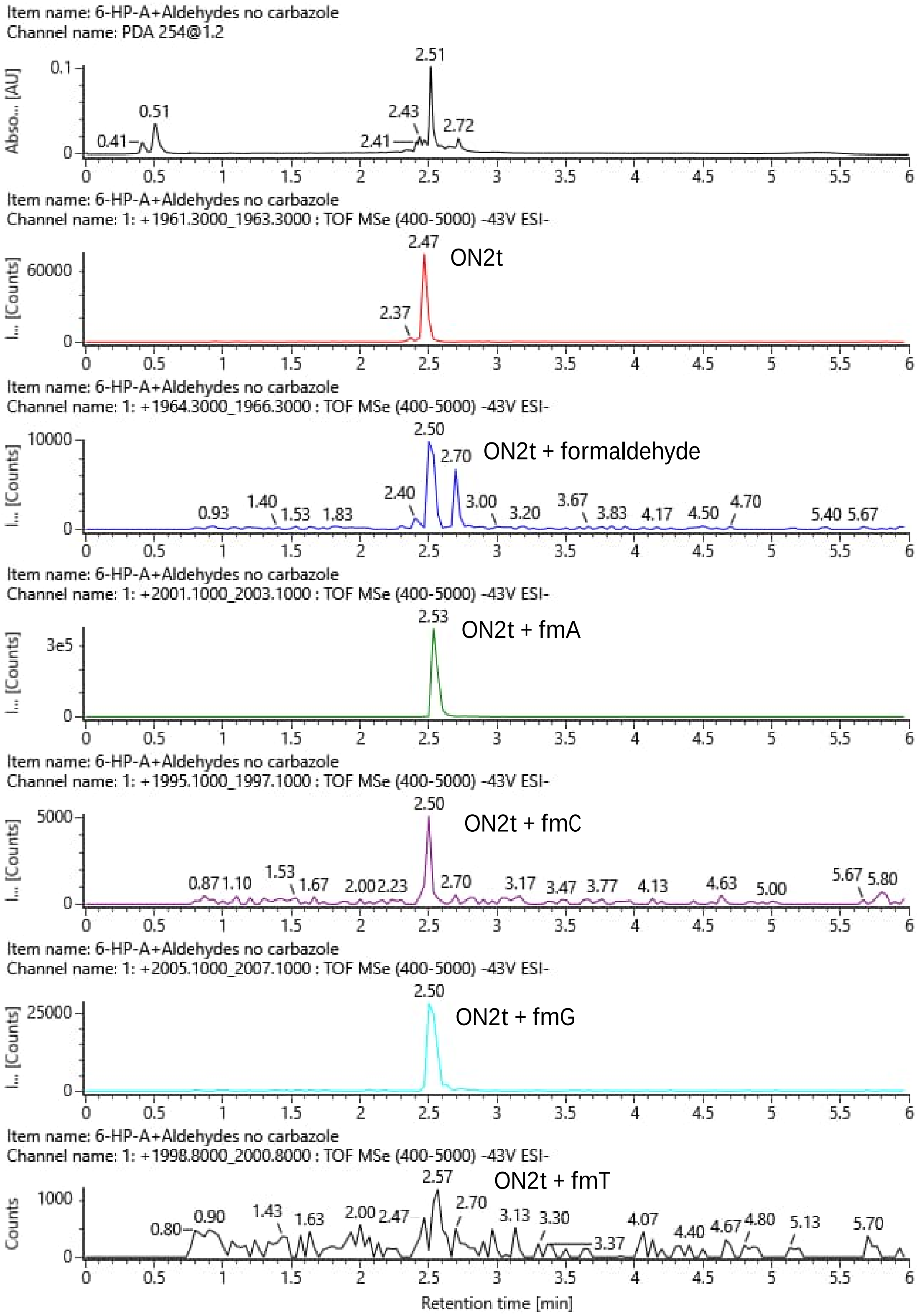

Figure S90. UV (top panel) and extracted ion RP-UPLC traces of the DCC product mixture on incubation of the hairpin oligonucleotide ON2t (1.0 µM) with a mixture of aldehydes fmA, fmC, fmG, fmT and fmB (2.0 µM) at 23 °C, pH = 5.5 (20 mM cacodylate buffer) and $I(NaClO_4)$ = 0.10 M for 120 h; ACQUITY Premier OST column (50 × 2.1 mm, 1.7 µm); flow rate 0.4 mL min^{-1}; linear gradient (5—25% over 4 min) of MeOH in aqueous solution of hexafluoroisopropanol (40 mM) and triethylamine (7 mM); λ = 254 nm; T = 60 °C.

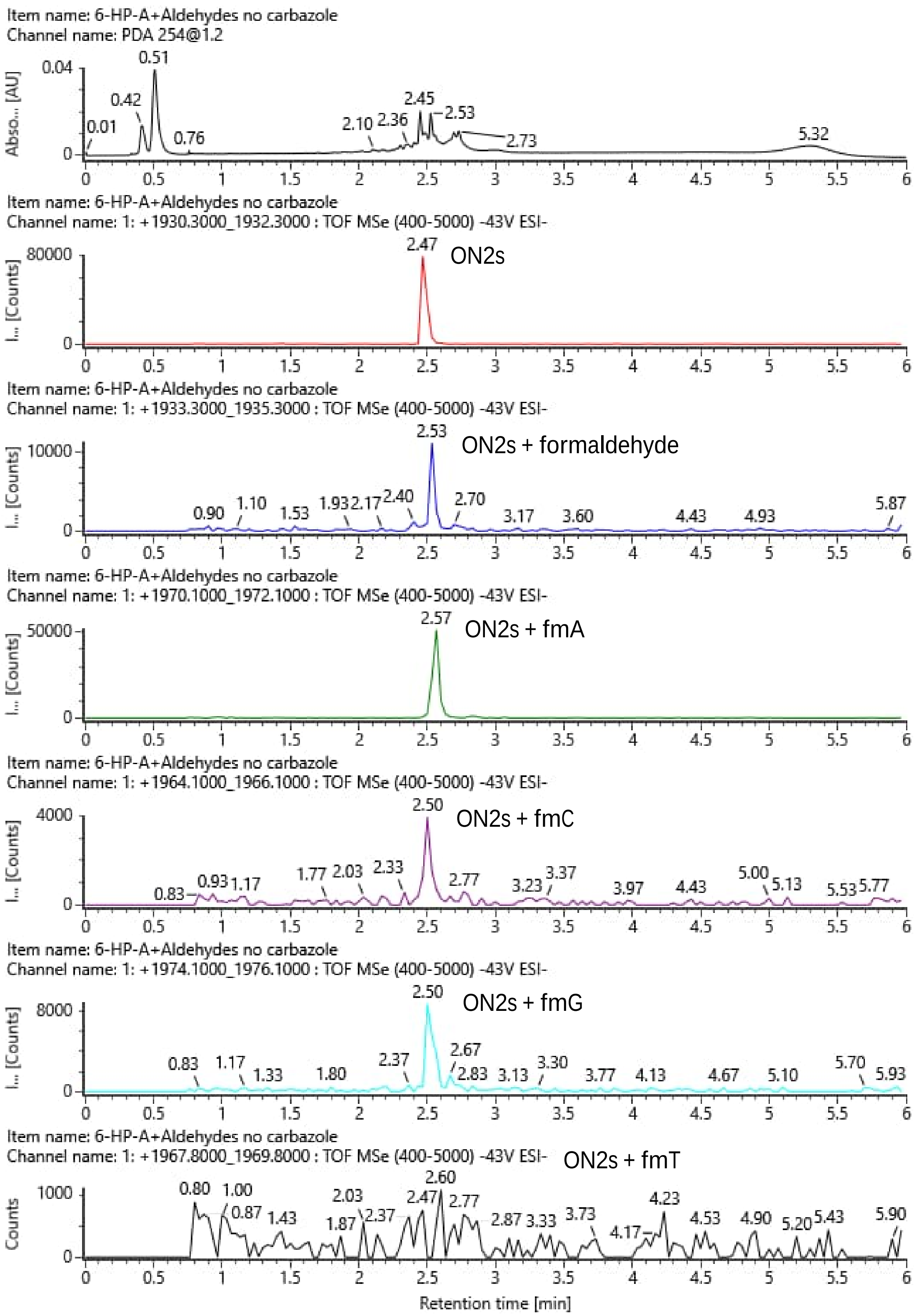

Figure S91. UV (top panel) and extracted ion RP-UPLC traces of the DCC product mixture on incubation of the hairpin oligonucleotide ON2s (1.0 µM) with a mixture of aldehydes fmA, fmC, fmG, fmT and fmB (2.0 µM) at 23 °C, pH = 5.5 (20 mM cacodylate buffer) and $I(NaClO_4)$ = 0.10 M for 120 h; ACQUITY Premier OST column (50 × 2.1 mm, 1.7 µm); flow rate 0.4 mL min^{-1}; linear gradient (5—25% over 4 min) of MeOH in aqueous solution of hexafluoroisopropanol (40 mM) and triethylamine (7 mM); λ = 254 nm; T = 60 °C.

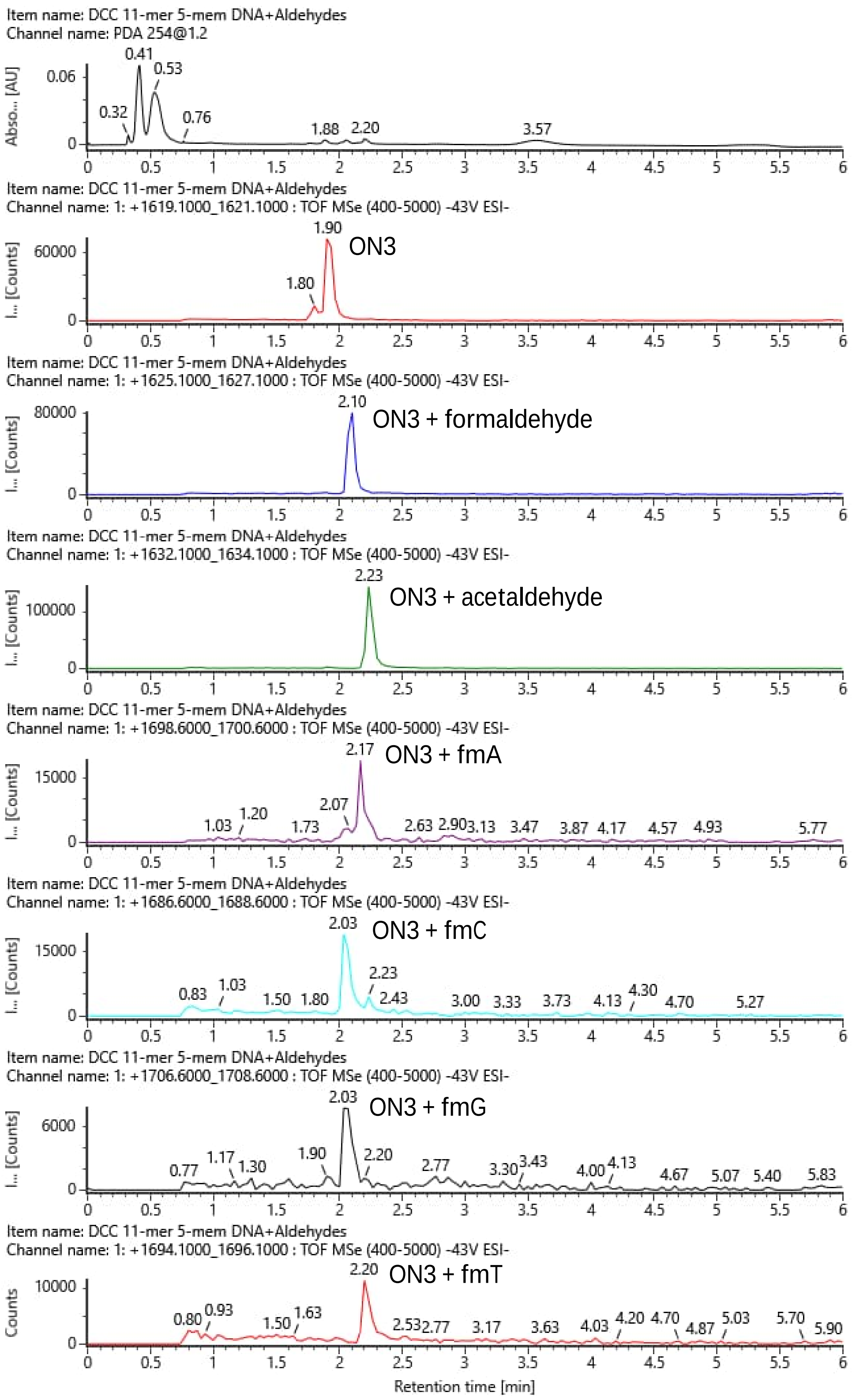

Figure S92. UV (top panel) and extracted ion RP-UPLC traces of the DCC product mixture on incubation of the single-stranded oligonucleotide ON3 (1.0 µM) with a mixture of aldehydes fmA, fmC, fmG, fmT and fmB (20 µM) at 23 °C, pH = 5.5 (20 mM cacodylate buffer) and $I(NaClO_4)$ = 0.10 M for 120 h; ACQUITY Premier OST column (50 × 2.1 mm, 1.7 µm); flow rate 0.4 mL min^{-1}; linear gradient (5—25% over 4 min) of MeOH in aqueous solution of hexafluoroisopropanol (40 mM) and triethylamine (7 mM); λ = 254 nm; T = 60 °C.

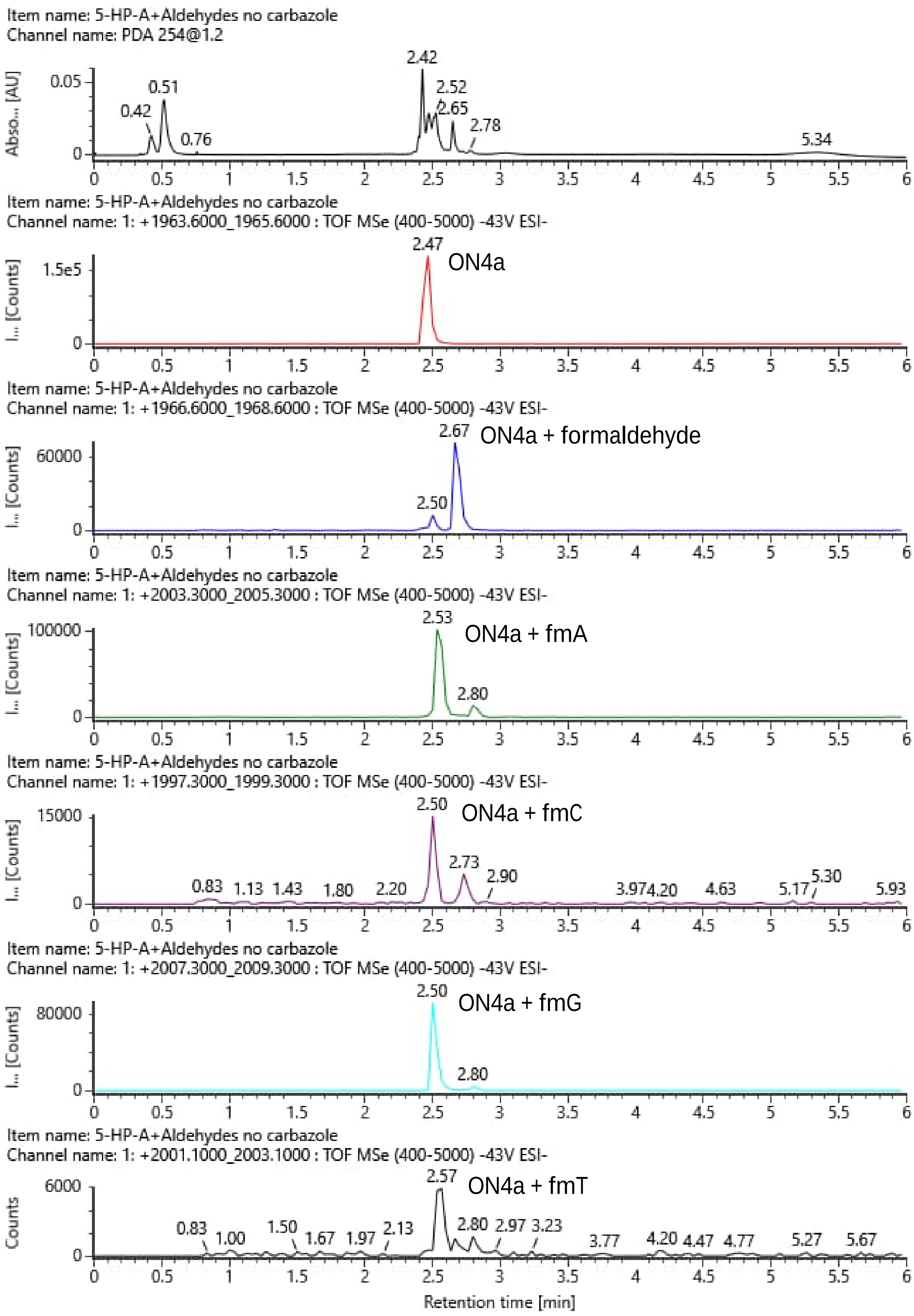

Figure S93. UV (top panel) and extracted ion RP-UPLC traces of the DCC product mixture on incubation of the hairpin oligonucleotide ON4a (1.0 µM) with a mixture of aldehydes fmA, fmC, fmG, fmT and fmB (2.0 µM) at 23 °C, pH = 5.5 (20 mM cacodylate buffer) and *I*($NaClO_4$) = 0.10 M for 120 h; ACQUITY Premier OST column (50 × 2.1 mm, 1.7 µm); flow rate 0.4 mL min^{-1}; linear gradient (5—25% over 4 min) of MeOH in aqueous solution of hexafluoroisopropanol (40 mM) and triethylamine (7 mM); λ = 254 nm; T = 60 °C.

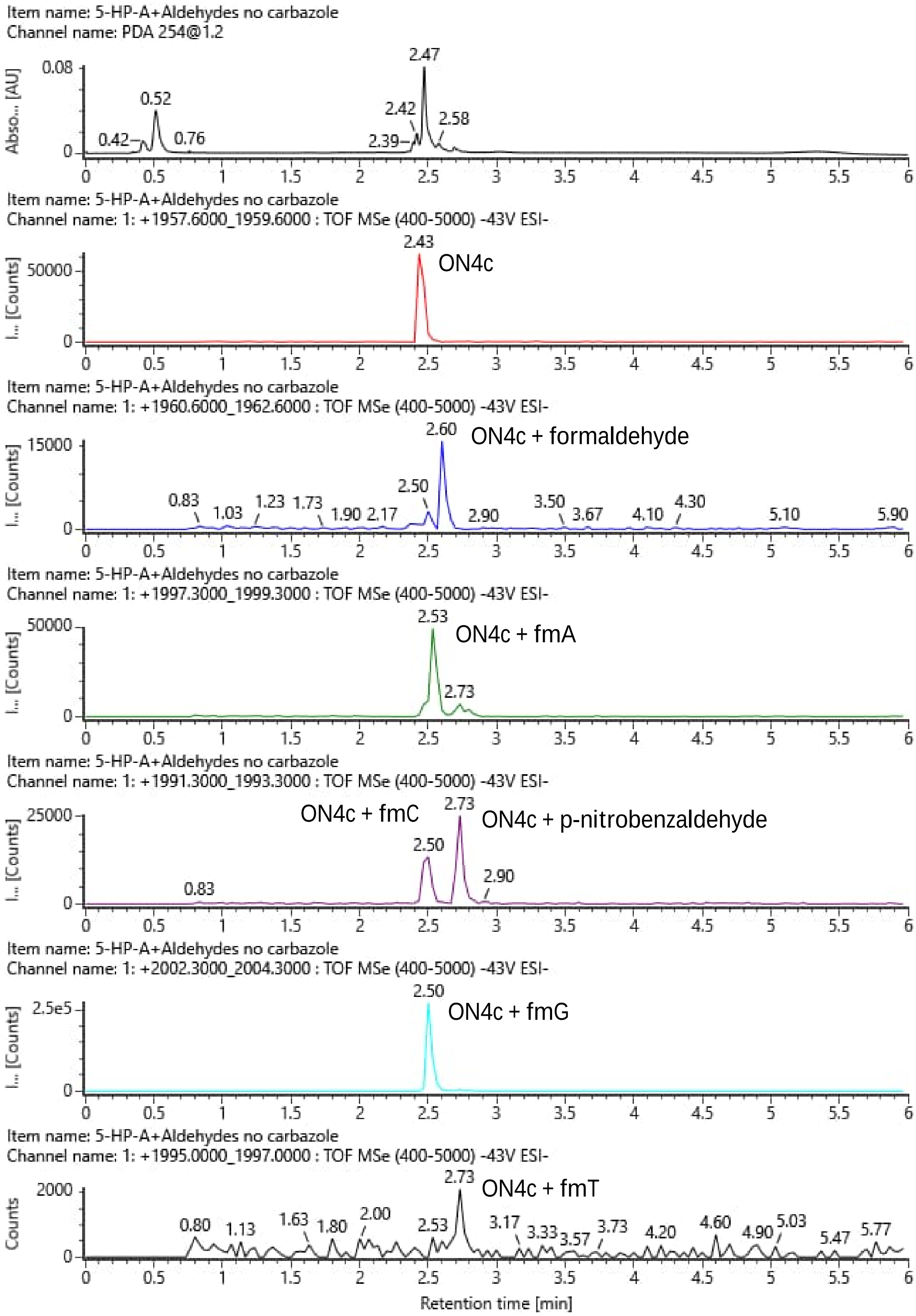

Figure S94. UV (top panel) and extracted ion RP-UPLC traces of the DCC product mixture on incubation of the hairpin oligonucleotide ON4c (1.0 µM) with a mixture of aldehydes fmA, fmC, fmG, fmT and fmB (2.0 µM) at 23 °C, pH = 5.5 (20 mM cacodylate buffer) and *I*($NaClO_4$) = 0.10 M for 120 h; ACQUITY Premier OST column (50 × 2.1 mm, 1.7 µm); flow rate 0.4 mL min^{-1}; linear gradient (5—25% over 4 min) of MeOH in aqueous solution of hexafluoroisopropanol (40 mM) and triethylamine (7 mM); λ = 254 nm; T = 60 °C.

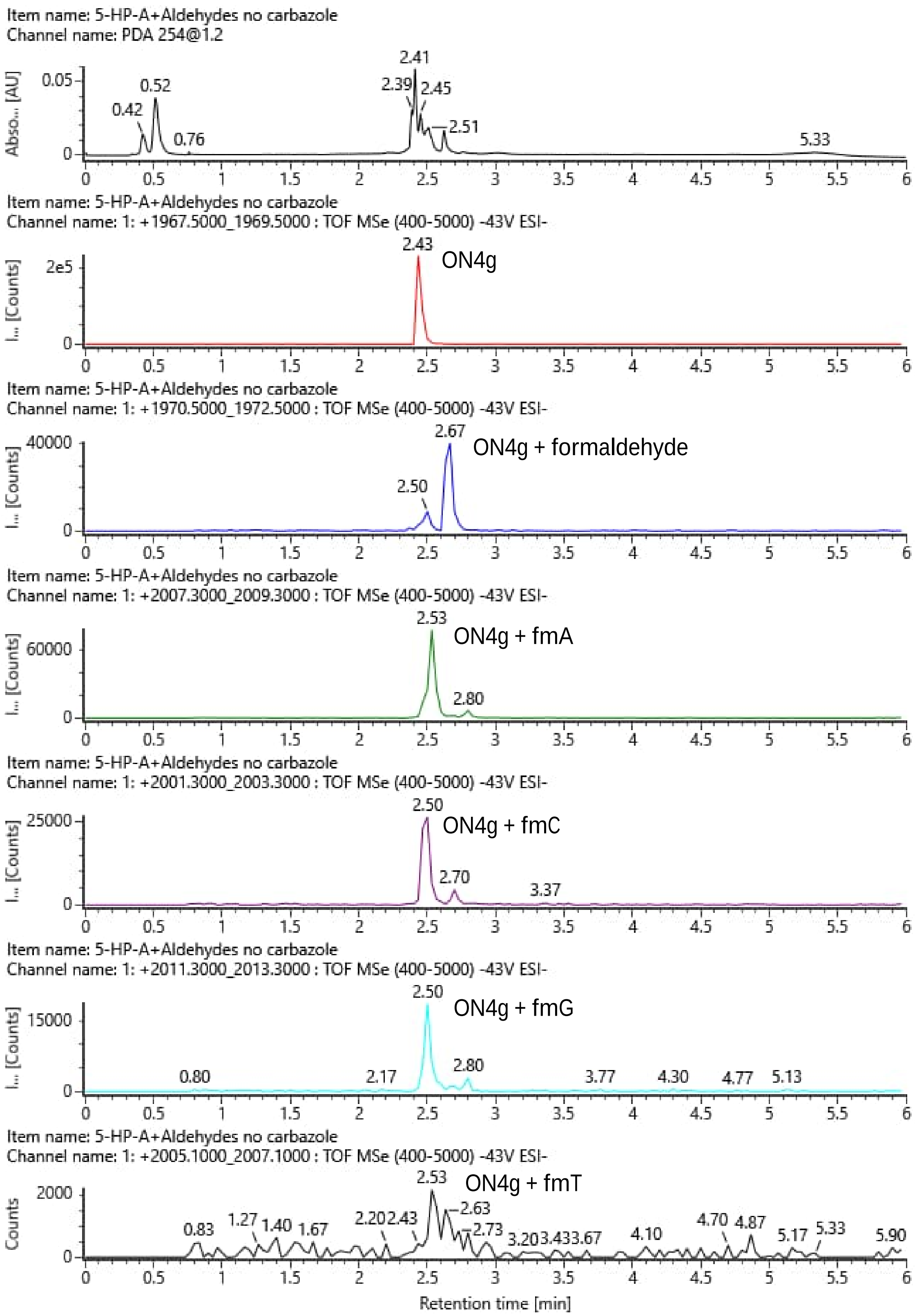

Figure S95. UV (top panel) and extracted ion RP-UPLC traces of the DCC product mixture on incubation of the hairpin oligonucleotide ON4g (1.0 µM) with a mixture of aldehydes fmA, fmC, fmG, fmT and fmB (2.0 µM) at 23 °C, pH = 5.5 (20 mM cacodylate buffer) and $I(NaClO_4)$ = 0.10 M for 120 h; ACQUITY Premier OST column (50 × 2.1 mm, 1.7 µm); flow rate 0.4 mL min^{-1}; linear gradient (5—25% over 4 min) of MeOH in aqueous solution of hexafluoroisopropanol (40 mM) and triethylamine (7 mM); λ = 254 nm; T = 60 °C.

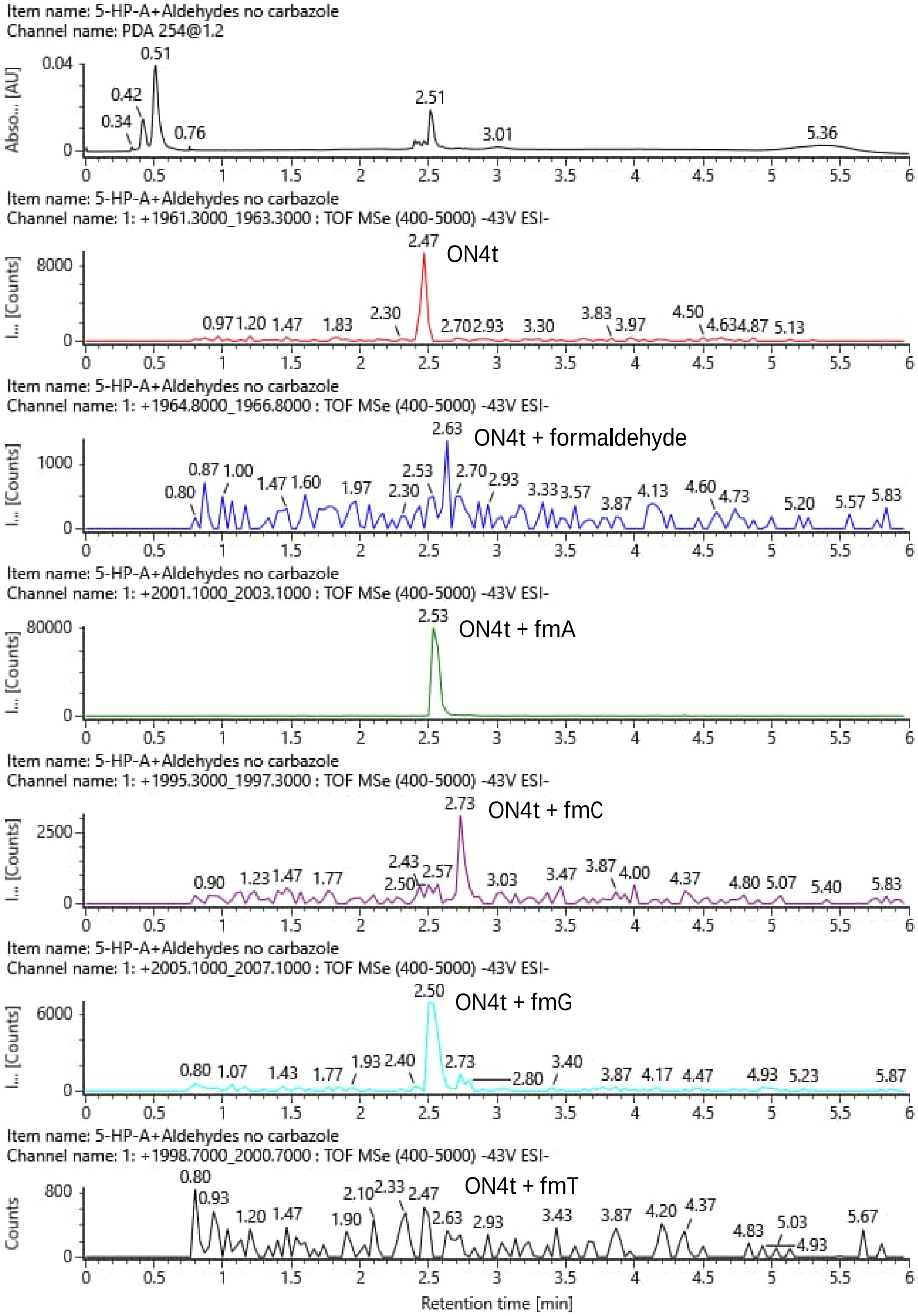

Figure S96. UV (top panel) and extracted ion RP-UPLC traces of the DCC product mixture on incubation of the hairpin oligonucleotide ON4t (1.0 μM) with a mixture of aldehydes fmA, fmC, fmG, fmT and fmB (2.0 μM) at 23 °C, pH = 5.5 (20 mM cacodylate buffer) and $I(NaClO_4)$ = 0.10 M for 120 h; ACQUITY Premier OST column (50 × 2.1 mm, 1.7 μm); flow rate 0.4 mL min^{-1}; linear gradient (5—25% over 4 min) of MeOH in aqueous solution of hexafluoroisopropanol (40 mM) and triethylamine (7 mM); λ = 254 nm; T = 60 °C.

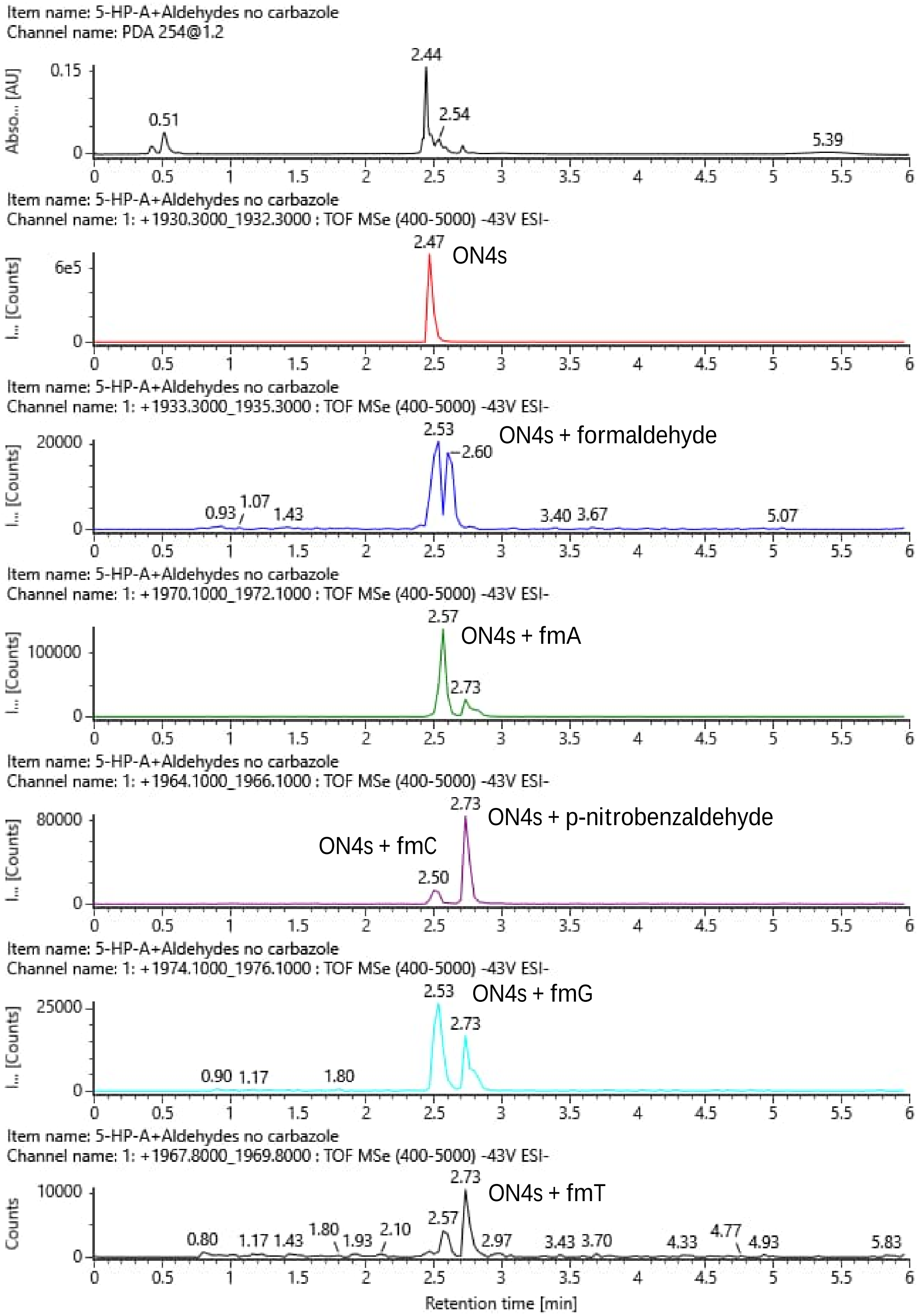

Figure S97. UV (top panel) and extracted ion RP-UPLC traces of the DCC product mixture on incubation of the hairpin oligonucleotide ON4s (1.0 µM) with a mixture of aldehydes fmA, fmC, fmG, fmT and fmB (2.0 µM) at 23 °C, pH = 5.5 (20 mM cacodylate buffer) and $I(NaClO_4)$ = 0.10 M for 120 h; ACQUITY Premier OST column (50 × 2.1 mm, 1.7 µm); flow rate 0.4 mL min^{-1}; linear gradient (5—25% over 4 min) of MeOH in aqueous solution of hexafluoroisopropanol (40 mM) and triethylamine (7 mM); λ = 254 nm; T = 60 °C.

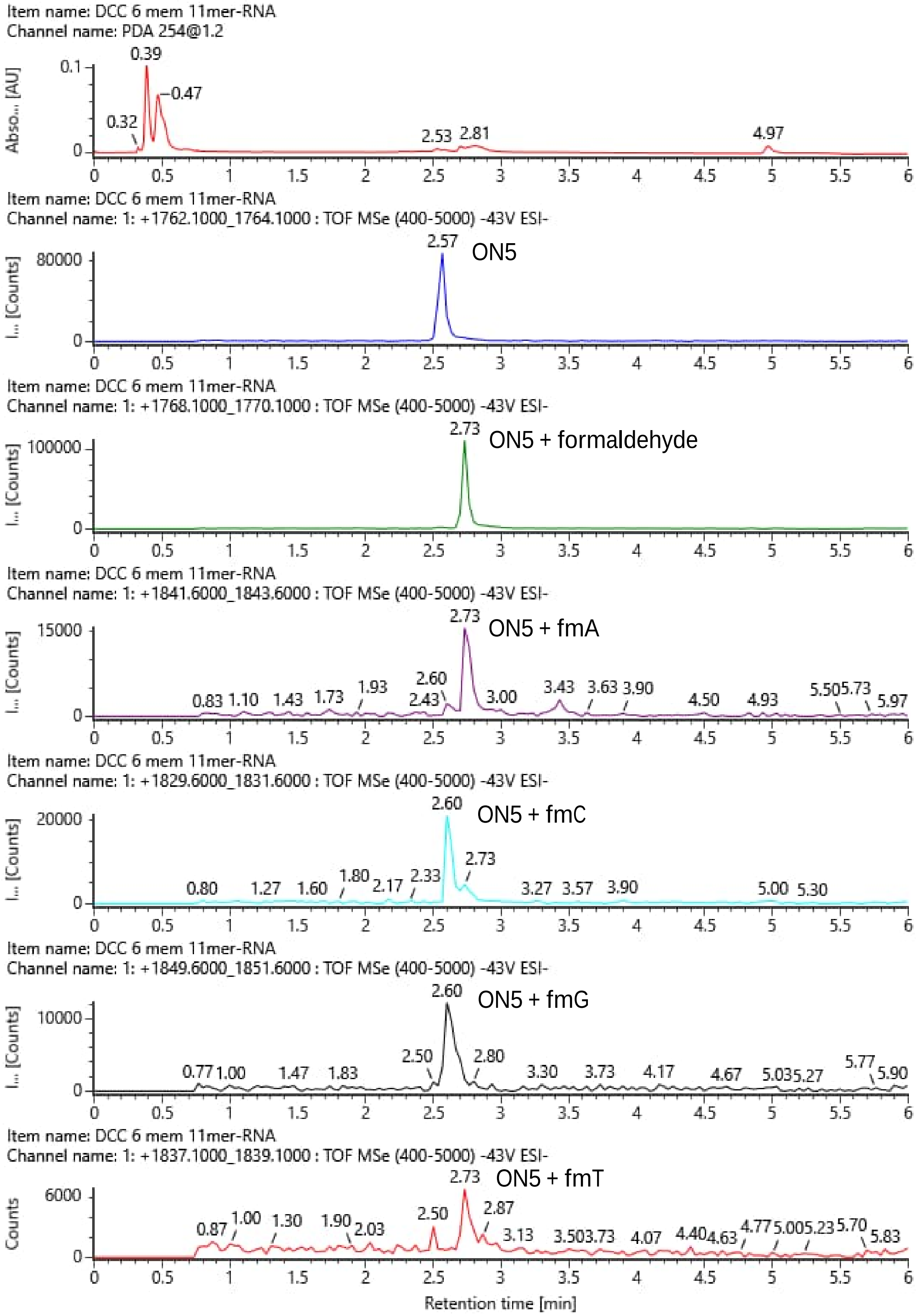

Figure S98. UV (top panel) and extracted ion RP-UPLC traces of the DCC product mixture on incubation of the single-stranded oligonucleotide ON5 (1.0 µM) with a mixture of aldehydes fmA, fmC, fmG, fmT and fmB (20 µM) at 23 °C, pH = 5.5 (20 mM cacodylate buffer) and $I(NaClO_4)$ = 0.10 M for 120 h; ACQUITY Premier OST column (50 × 2.1 mm, 1.7 µm); flow rate 0.4 mL min^{-1}; linear gradient (5—25% over 4 min) of MeOH in aqueous solution of hexafluoroisopropanol (40 mM) and triethylamine (7 mM); λ = 254 nm; T = 60 °C.

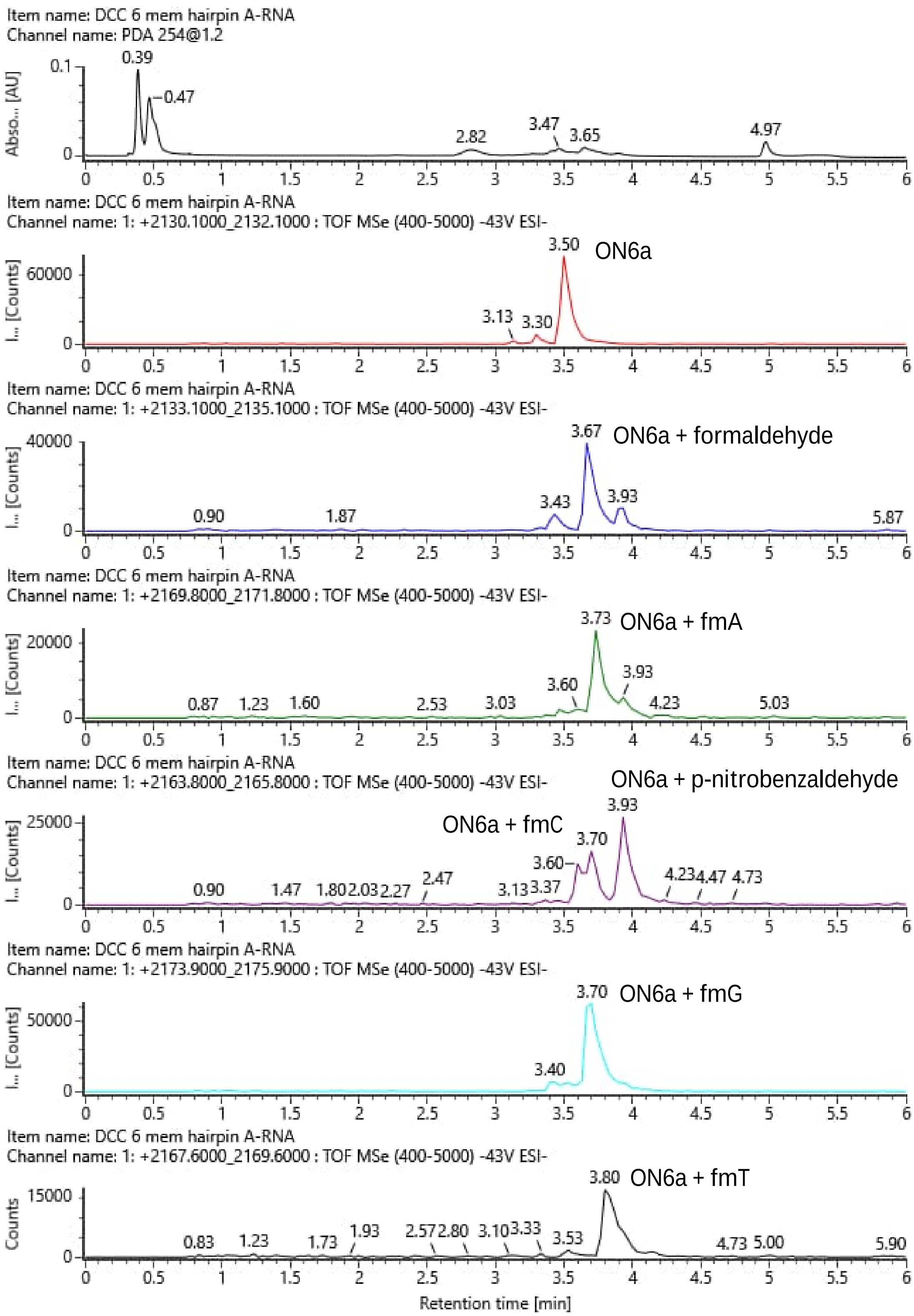

Figure S99. UV (top panel) and extracted ion RP-UPLC traces of the DCC product mixture on incubation of the hairpin oligonucleotide ON6a (1.0 µM) with a mixture of aldehydes fmA, fmC, fmG, fmT and fmB (2.0 µM) at 23 °C, pH = 5.5 (20 mM cacodylate buffer) and $I(NaClO_4)$ = 0.10 M for 120 h; ACQUITY Premier OST column (50 × 2.1 mm, 1.7 µm); flow rate 0.4 mL min^{-1}; linear gradient (5—25% over 4 min) of MeOH in aqueous solution of hexafluoroisopropanol (40 mM) and triethylamine (7 mM); λ = 254 nm; T = 60 °C.

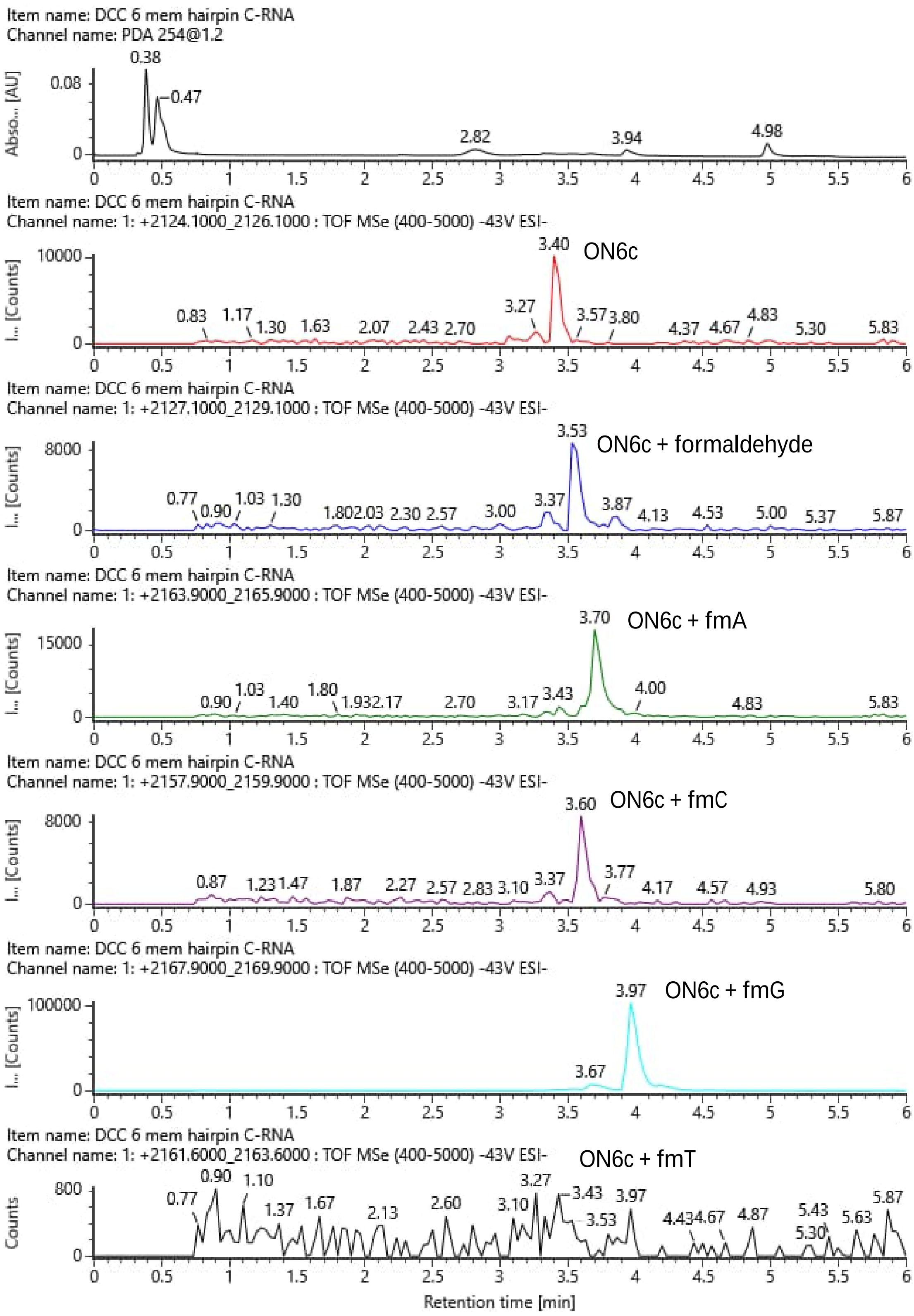

Figure S100. UV (top panel) and extracted ion RP-UPLC traces of the DCC product mixture on incubation of the hairpin oligonucleotide ON6c (1.0 µM) with a mixture of aldehydes fmA, fmC, fmG, fmT and fmB (2.0 µM) at 23 °C, pH = 5.5 (20 mM cacodylate buffer) and $I(NaClO_4)$ = 0.10 M for 120 h; ACQUITY Premier OST column (50 × 2.1 mm, 1.7 µm); flow rate 0.4 mL min^{-1}; linear gradient (5—25% over 4 min) of MeOH in aqueous solution of hexafluoroisopropanol (40 mM) and triethylamine (7 mM); λ = 254 nm; T = 60 °C.

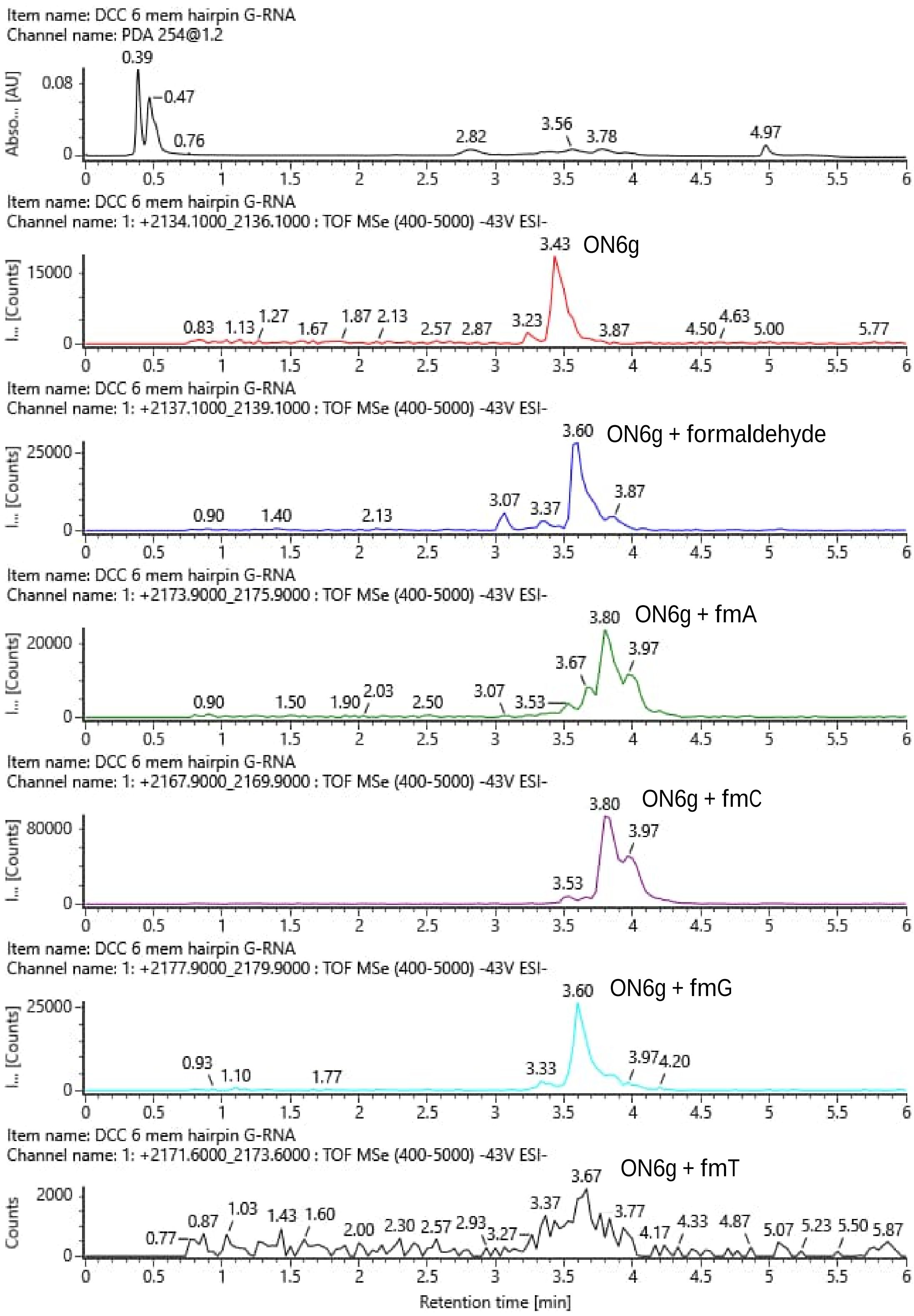

Figure S101. UV (top panel) and extracted ion RP-UPLC traces of the DCC product mixture on incubation of the hairpin oligonucleotide ON6g (1.0 µM) with a mixture of aldehydes fmA, fmC, fmG, fmT and fmB (2.0 µM) at 23 °C, pH = 5.5 (20 mM cacodylate buffer) and $I(NaClO_4)$ = 0.10 M for 120 h; ACQUITY Premier OST column (50 × 2.1 mm, 1.7 µm); flow rate 0.4 mL min^{-1}; linear gradient (5—25% over 4 min) of MeOH in aqueous solution of hexafluoroisopropanol (40 mM) and triethylamine (7 mM); λ = 254 nm; T = 60 °C.

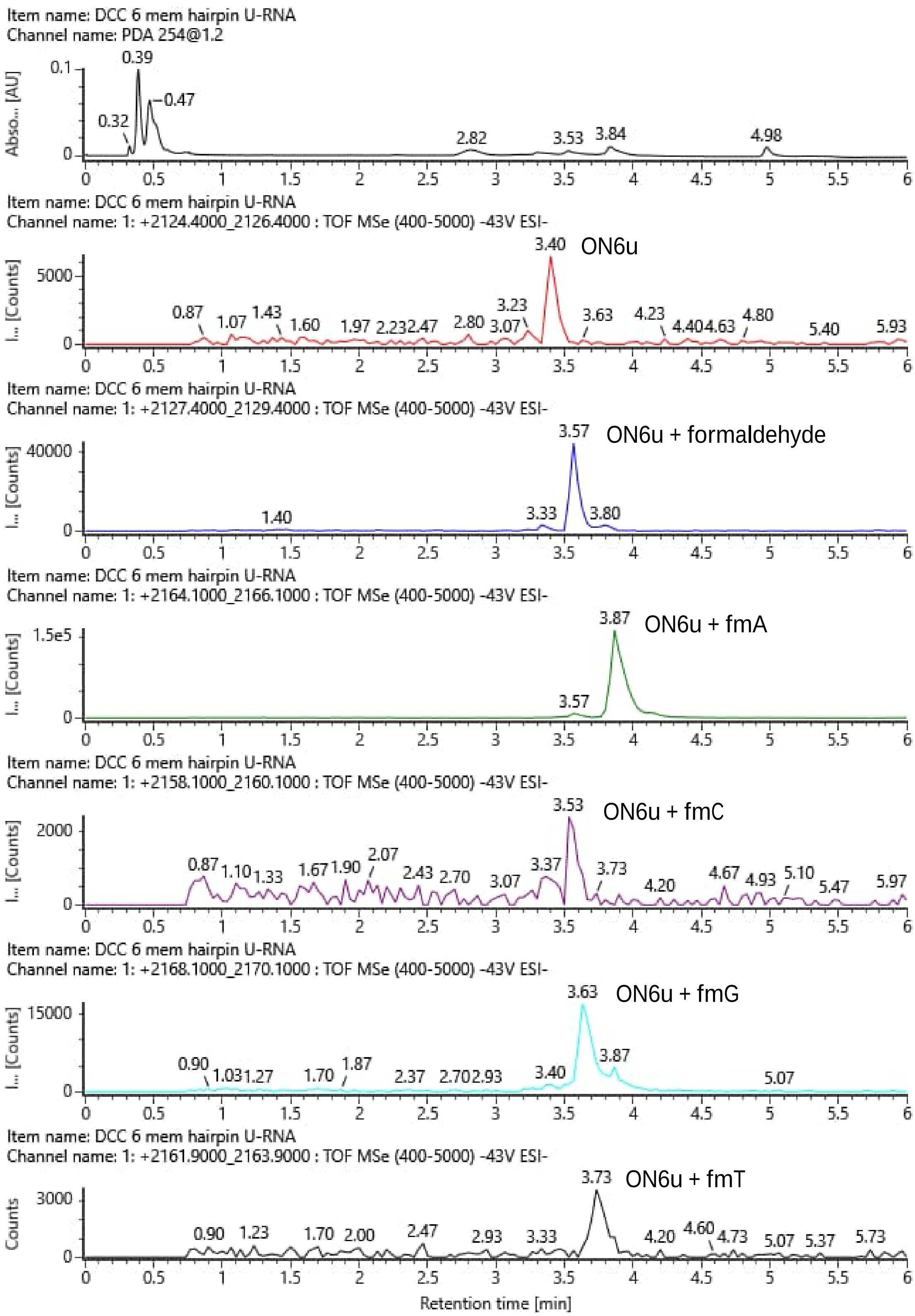

Figure S102. UV (top panel) and extracted ion RP-UPLC traces of the DCC product mixture on incubation of the hairpin oligonucleotide ON6u (1.0 µM) with a mixture of aldehydes fmA, fmC, fmG, fmT and fmB (2.0 µM) at 23 °C, pH = 5.5 (20 mM cacodylate buffer) and $I(NaClO_4)$ = 0.10 M for 120 h; ACQUITY Premier OST column (50 × 2.1 mm, 1.7 µm); flow rate 0.4 mL min^{-1}; linear gradient (5—25% over 4 min) of MeOH in aqueous solution of hexafluoroisopropanol (40 mM) and triethylamine (7 mM); λ = 254 nm; T = 60 °C.

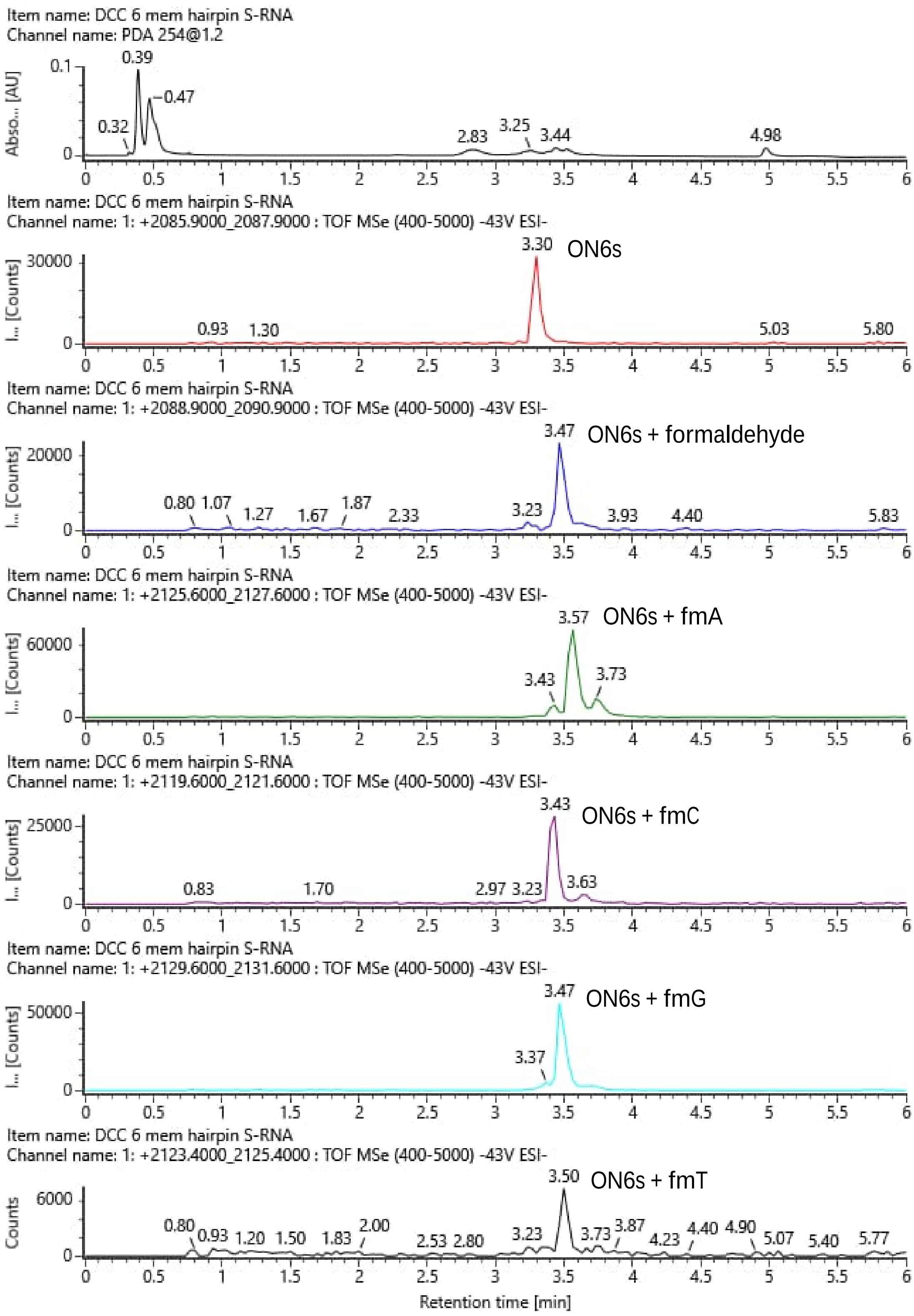

Figure S103. UV (top panel) and extracted ion RP-UPLC traces of the DCC product mixture on incubation of the hairpin oligonucleotide ON6s (1.0 µM) with a mixture of aldehydes fmA, fmC, fmG, fmT and fmB (2.0 µM) at 23 °C, pH = 5.5 (20 mM cacodylate buffer) and $I(NaClO_4)$ = 0.10 M for 120 h; ACQUITY Premier OST column (50 × 2.1 mm, 1.7 µm); flow rate 0.4 mL min^{-1}; linear gradient (5—25% over 4 min) of MeOH in aqueous solution of hexafluoroisopropanol (40 mM) and triethylamine (7 mM); λ = 254 nm; T = 60 °C.

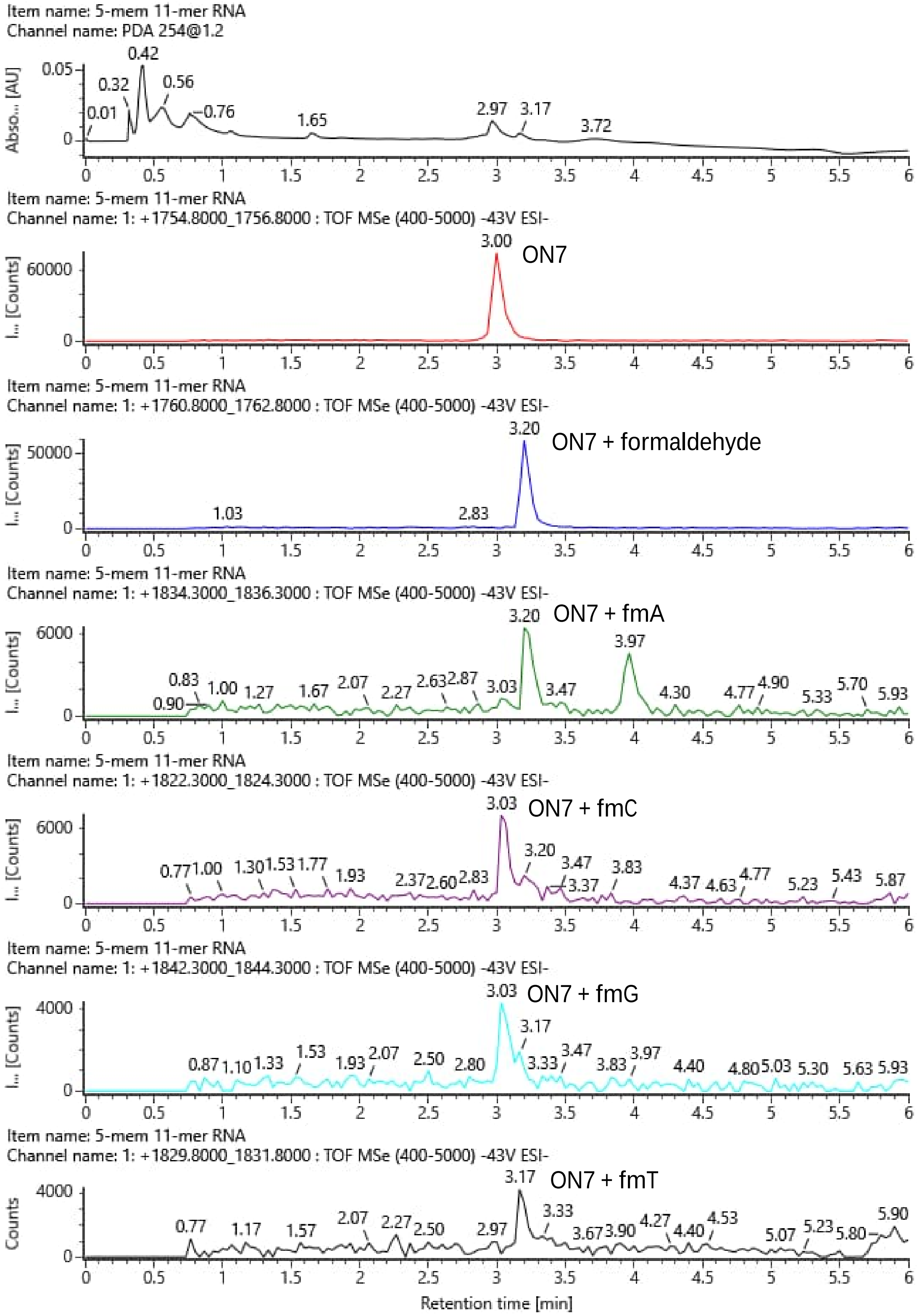

Figure S104. UV (top panel) and extracted ion RP-UPLC traces of the DCC product mixture on incubation of the single-stranded oligonucleotide ON7 (1.0 µM) with a mixture of aldehydes fmA, fmC, fmG, fmT and fmB (20 µM) at 23 °C, pH = 5.5 (20 mM cacodylate buffer) and $I(NaClO_4)$ = 0.10 M for 120 h; ACQUITY Premier OST column (50 × 2.1 mm, 1.7 µm); flow rate 0.4 mL min^{-1}; linear gradient (5—25% over 4 min) of MeOH in aqueous solution of hexafluoroisopropanol (40 mM) and triethylamine (7 mM); λ = 254 nm; T = 60 °C.

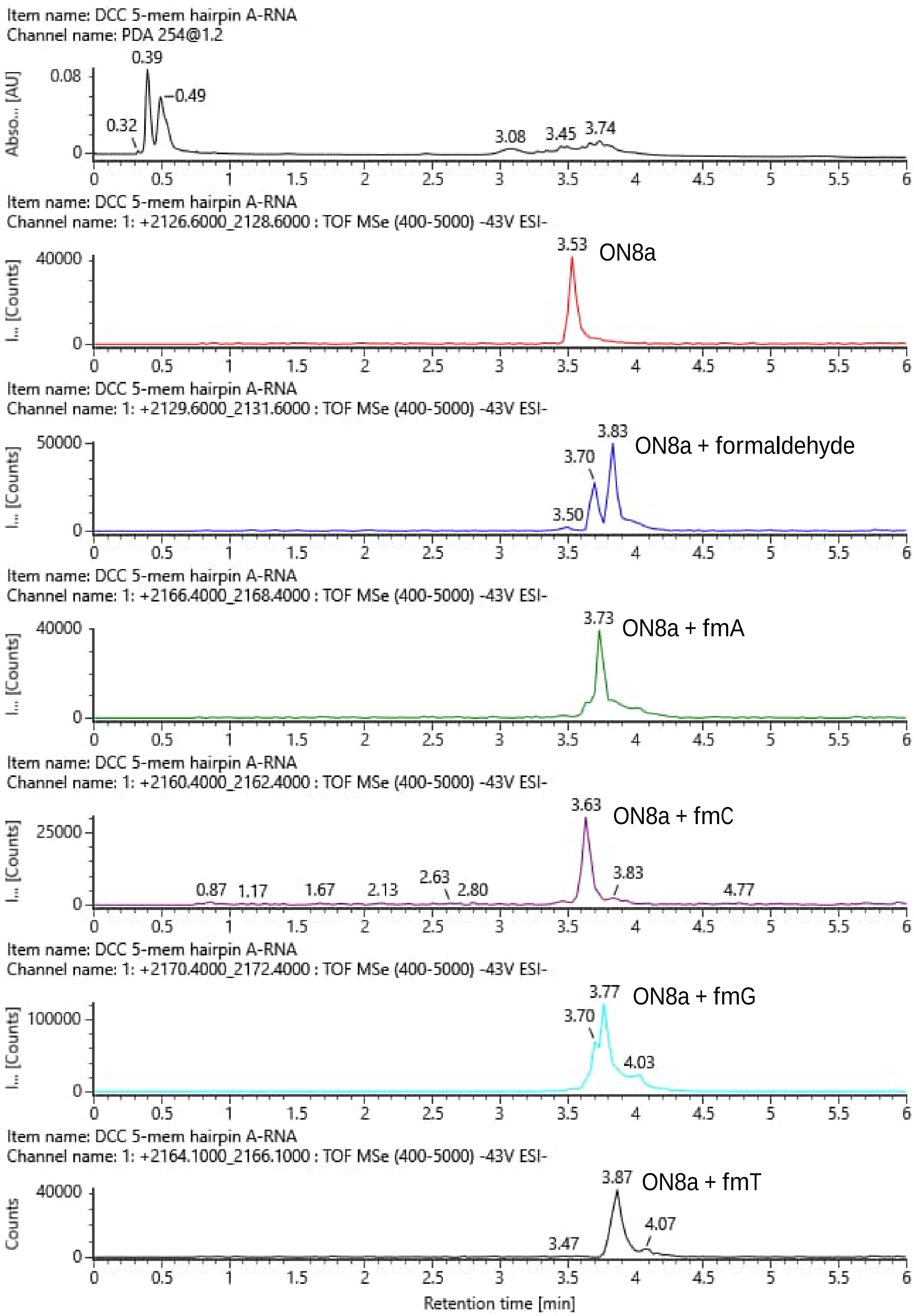

Figure S105. UV (top panel) and extracted ion RP-UPLC traces of the DCC product mixture on incubation of the hairpin oligonucleotide ON8a (1.0 µM) with a mixture of aldehydes fmA, fmC, fmG, fmT and fmB (2.0 µM) at 23 °C, pH = 5.5 (20 mM cacodylate buffer) and $I(NaClO_4)$ = 0.10 M for 120 h; ACQUITY Premier OST column (50 × 2.1 mm, 1.7 µm); flow rate 0.4 mL min^{-1}; linear gradient (5—25% over 4 min) of MeOH in aqueous solution of hexafluoroisopropanol (40 mM) and triethylamine (7 mM); λ = 254 nm; T = 60 °C.

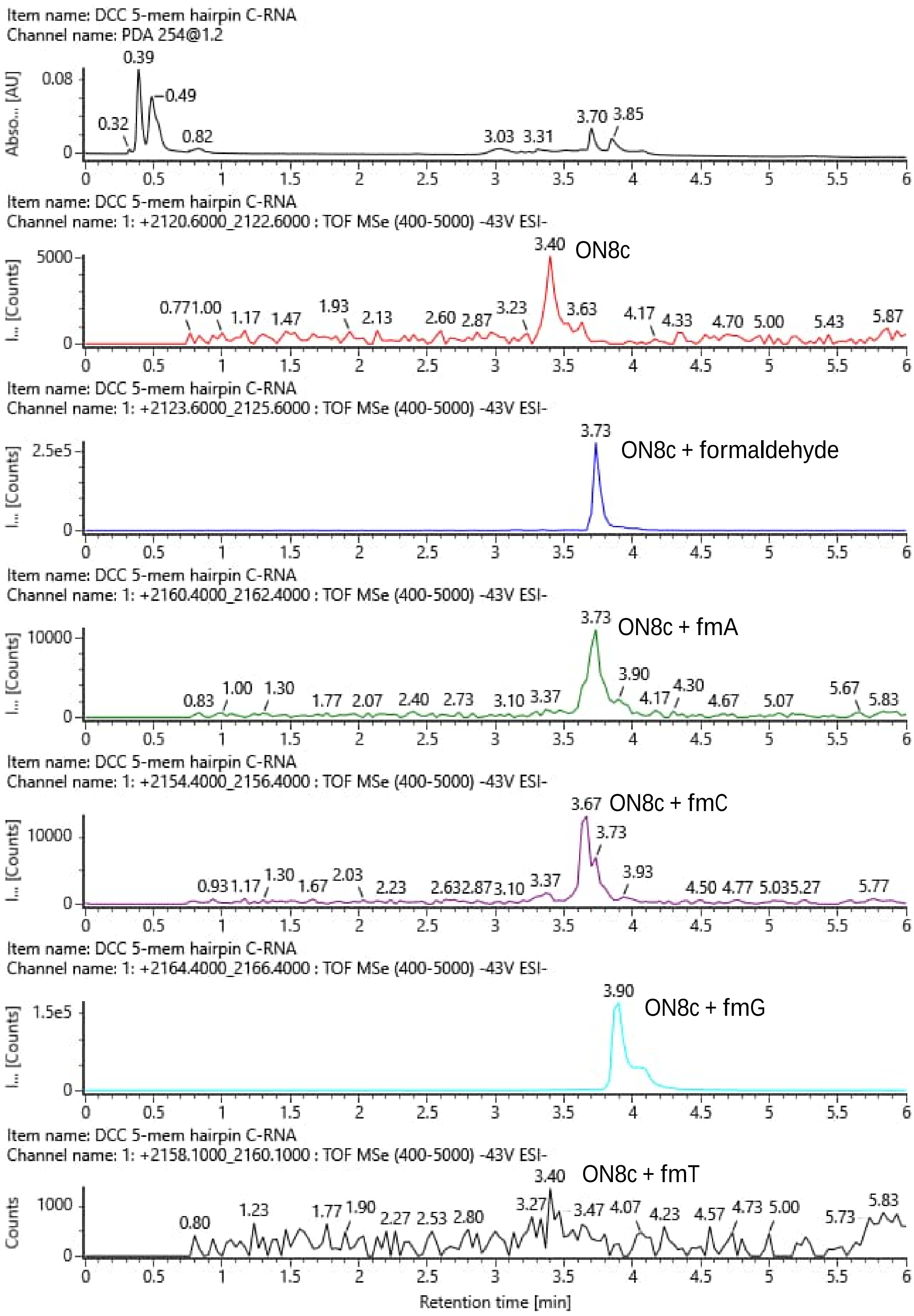

Figure S106. UV (top panel) and extracted ion RP-UPLC traces of the DCC product mixture on incubation of the hairpin oligonucleotide ON8c (1.0 µM) with a mixture of aldehydes fmA, fmC, fmG, fmT and fmB (2.0 µM) at 23 °C, pH = 5.5 (20 mM cacodylate buffer) and *I*($NaClO_4$) = 0.10 M for 120 h; ACQUITY Premier OST column (50 × 2.1 mm, 1.7 µm); flow rate 0.4 mL min^{-1}; linear gradient (5—25% over 4 min) of MeOH in aqueous solution of hexafluoroisopropanol (40 mM) and triethylamine (7 mM); λ = 254 nm; T = 60 °C.

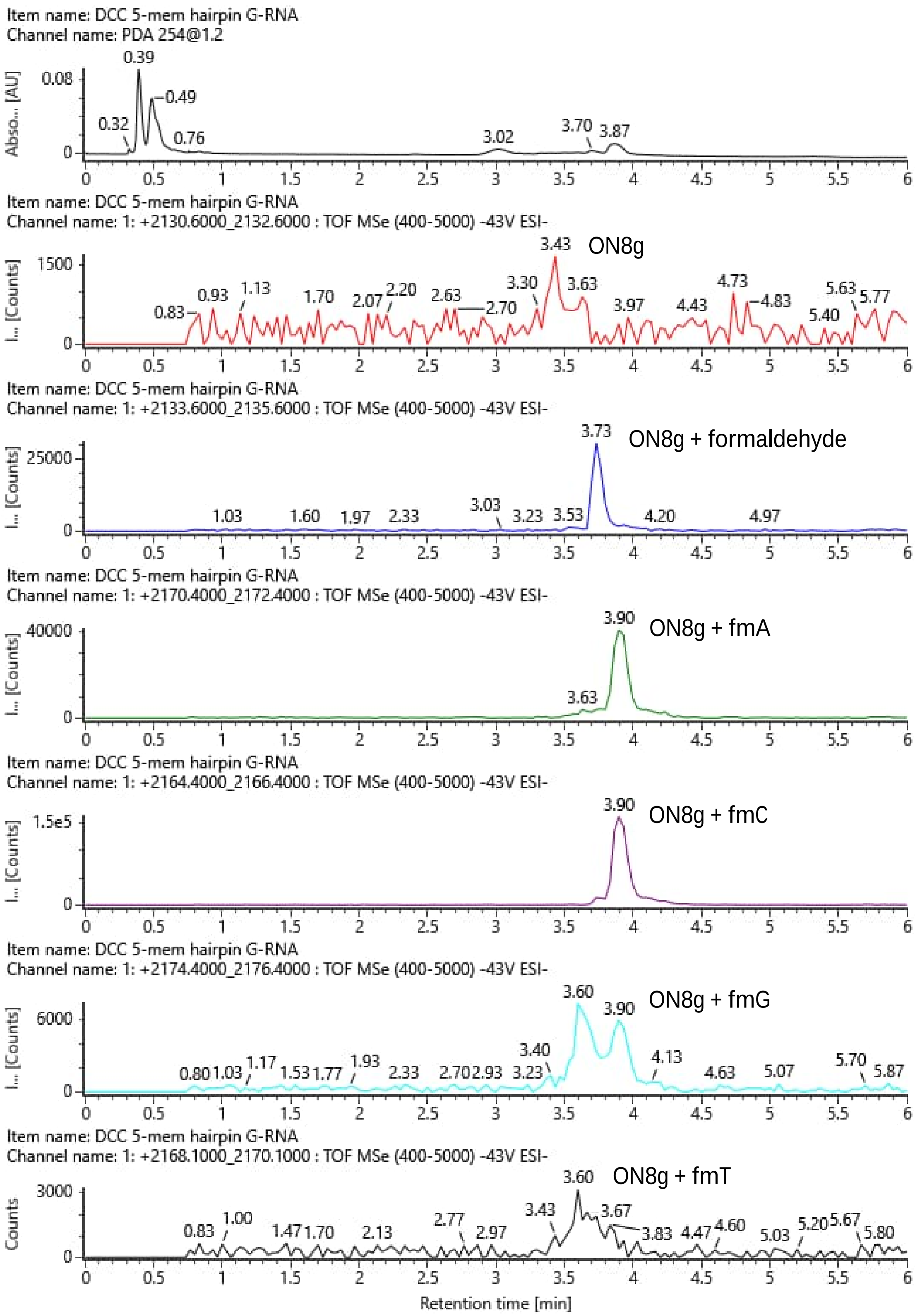

Figure S107. UV (top panel) and extracted ion RP-UPLC traces of the DCC product mixture on incubation of the hairpin oligonucleotide ON8g (1.0 µM) with a mixture of aldehydes fmA, fmC, fmG, fmT and fmB (2.0 µM) at 23 °C, pH = 5.5 (20 mM cacodylate buffer) and $I(NaClO_4)$ = 0.10 M for 120 h; ACQUITY Premier OST column (50 × 2.1 mm, 1.7 µm); flow rate 0.4 mL min^{-1}; linear gradient (5—25% over 4 min) of MeOH in aqueous solution of hexafluoroisopropanol (40 mM) and triethylamine (7 mM); λ = 254 nm; T = 60 °C.

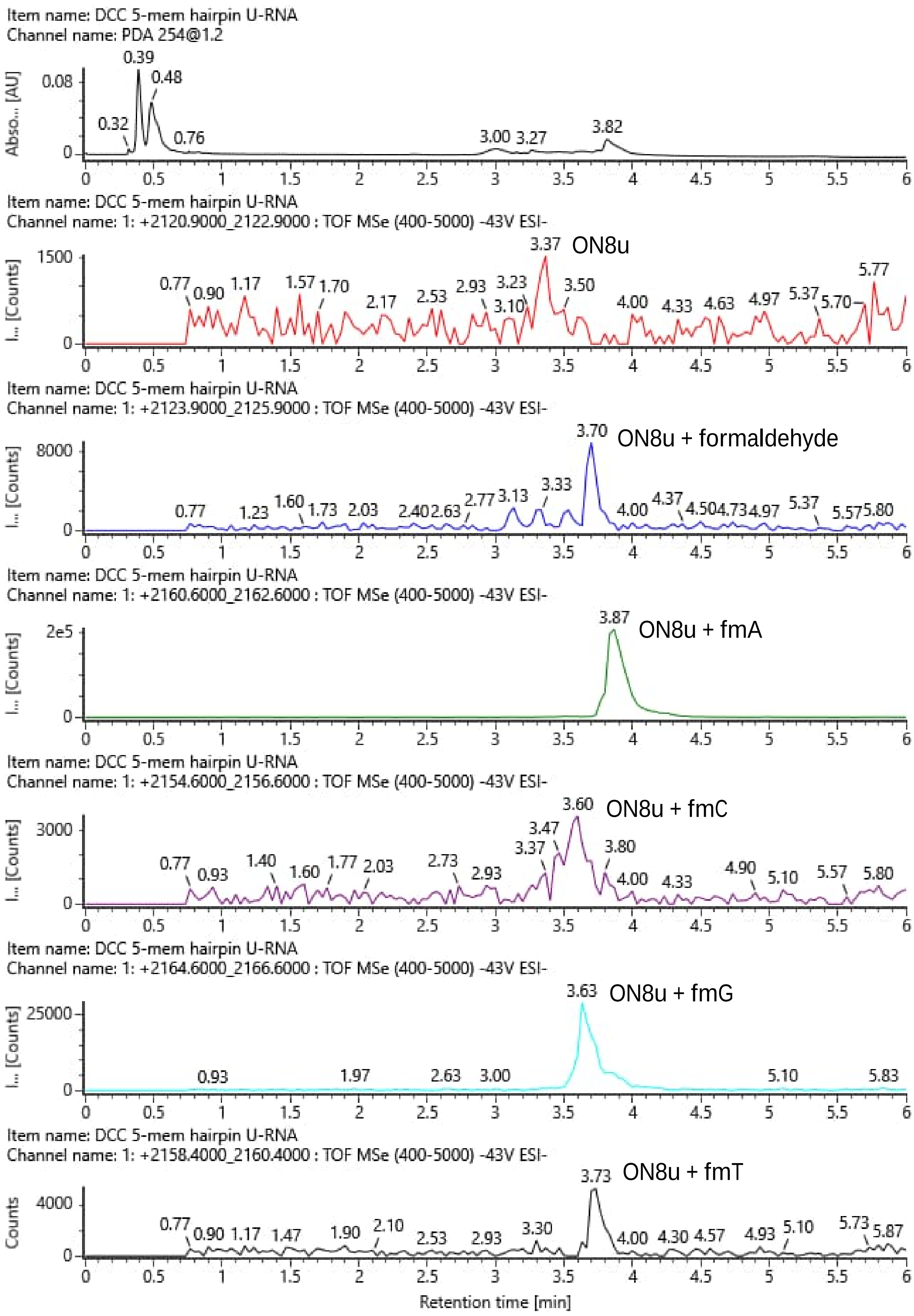

Figure S108. UV (top panel) and extracted ion RP-UPLC traces of the DCC product mixture on incubation of the hairpin oligonucleotide ON8u (1.0 µM) with a mixture of aldehydes fmA, fmC, fmG, fmT and fmB (2.0 µM) at 23 °C, pH = 5.5 (20 mM cacodylate buffer) and *I*($NaClO_4$) = 0.10 M for 120 h; ACQUITY Premier OST column (50 × 2.1 mm, 1.7 µm); flow rate 0.4 mL min^{-1}; linear gradient (5—25% over 4 min) of MeOH in aqueous solution of hexafluoroisopropanol (40 mM) and triethylamine (7 mM); λ = 254 nm; T = 60 °C.

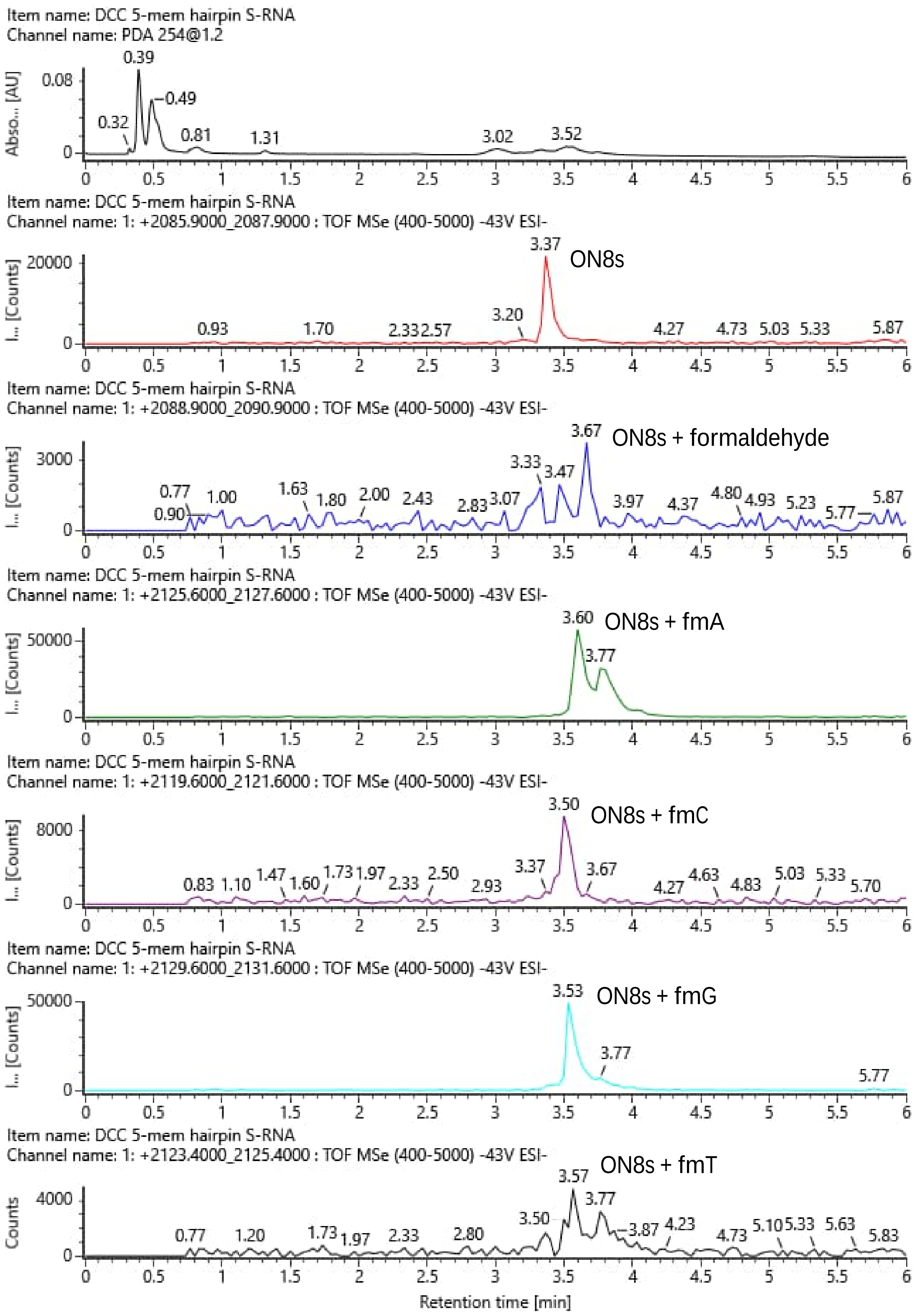

Figure S109. UV (top panel) and extracted ion RP-UPLC traces of the DCC product mixture on incubation of the hairpin oligonucleotide ON8s (1.0 µM) with a mixture of aldehydes fmA, fmC, fmG, fmT and fmB (2.0 µM) at 23 °C, pH = 5.5 (20 mM cacodylate buffer) and $I(NaClO_4)$ = 0.10 M for 120 h; ACQUITY Premier OST column (50 × 2.1 mm, 1.7 µm); flow rate 0.4 mL min^{-1}; linear gradient (5—25% over 4 min) of MeOH in aqueous solution of hexafluoroisopropanol (40 mM) and triethylamine (7 mM); λ = 254 nm; T = 60 °C.

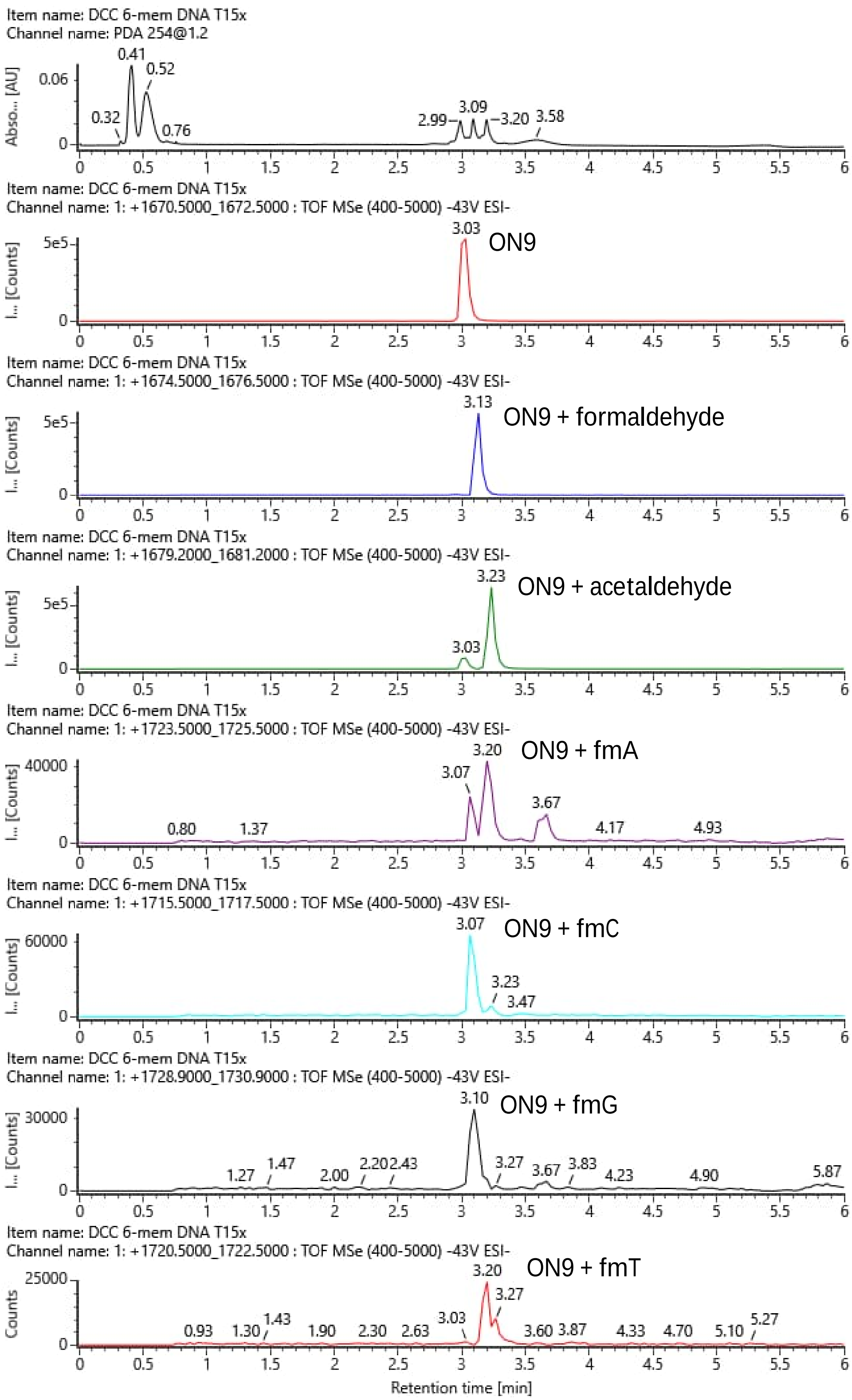

Figure S110. UV (top panel) and extracted ion RP-UPLC traces of the DCC product mixture on incubation of the single-stranded oligonucleotide ON9 (1.0 µM) with a mixture of aldehydes fmA, fmC, fmG, fmT and fmB (20 µM) at 23 °C, pH = 5.5 (20 mM cacodylate buffer) and $I(NaClO_4)$ = 0.10 M for 120 h; ACQUITY Premier OST column (50 × 2.1 mm, 1.7 µm); flow rate 0.4 mL min^{-1}; linear gradient (5—25% over 4 min) of MeOH in aqueous solution of hexafluoroisopropanol (40 mM) and triethylamine (7 mM); λ = 254 nm; T = 60 °C.

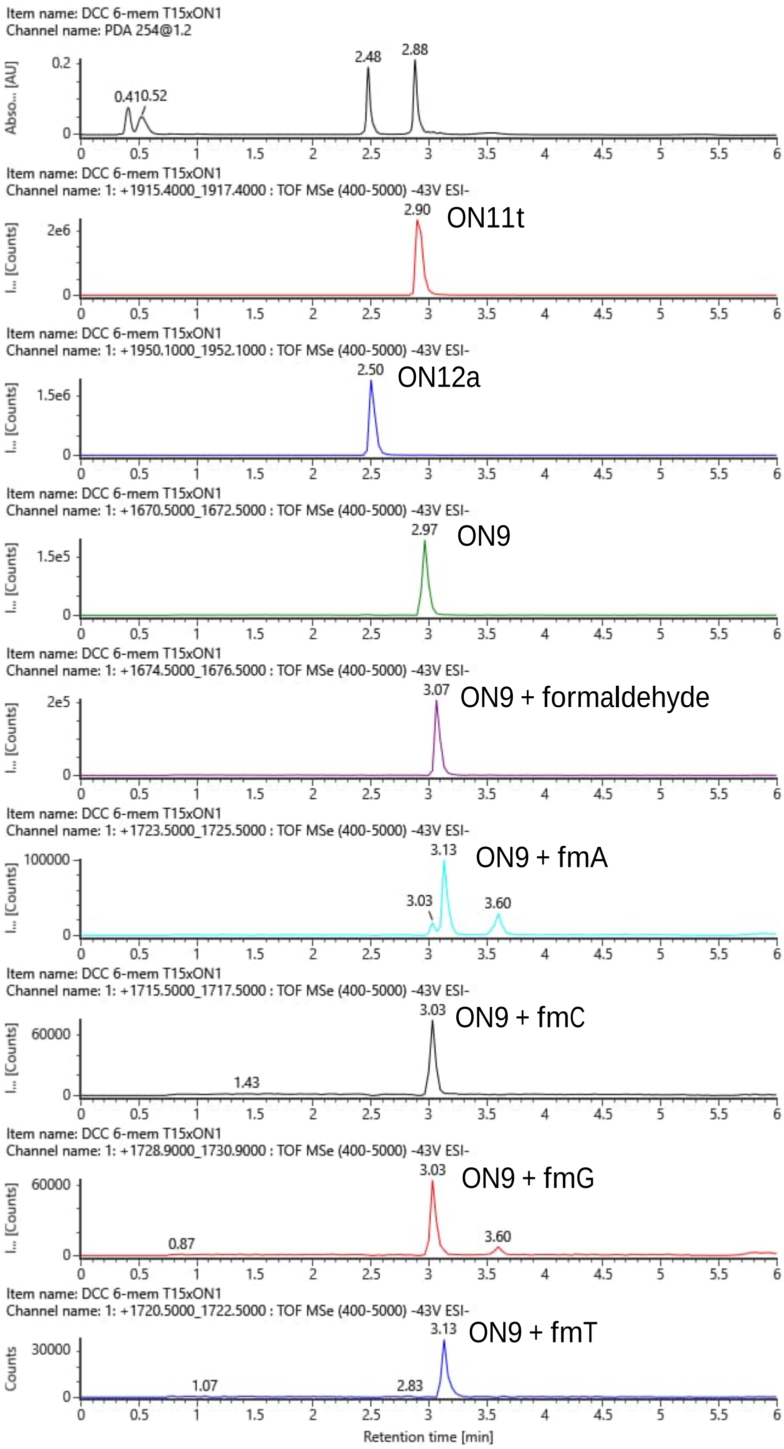

Figure S111. UV (top panel) and extracted ion RP-UPLC traces of the DCC product mixture on incubation of the oligonucleotide triplex ON11t•ON12a*ON9 (1.0 µM) with a mixture of aldehydes fmA, fmC, fmG, fmT and fmB (2.0 µM) at 23 °C, pH = 5.5 (20 mM cacodylate buffer) and *I*($NaClO_4$) = 0.10 M for 120 h; ACQUITY Premier OST column (50 × 2.1 mm, 1.7 µm); flow rate 0.4 mL min^{-1}; linear gradient (5—25% over 4 min) of MeOH in aqueous solution of hexafluoroisopropanol (40 mM) and triethylamine (7 mM); λ = 254 nm; T = 60 °C.

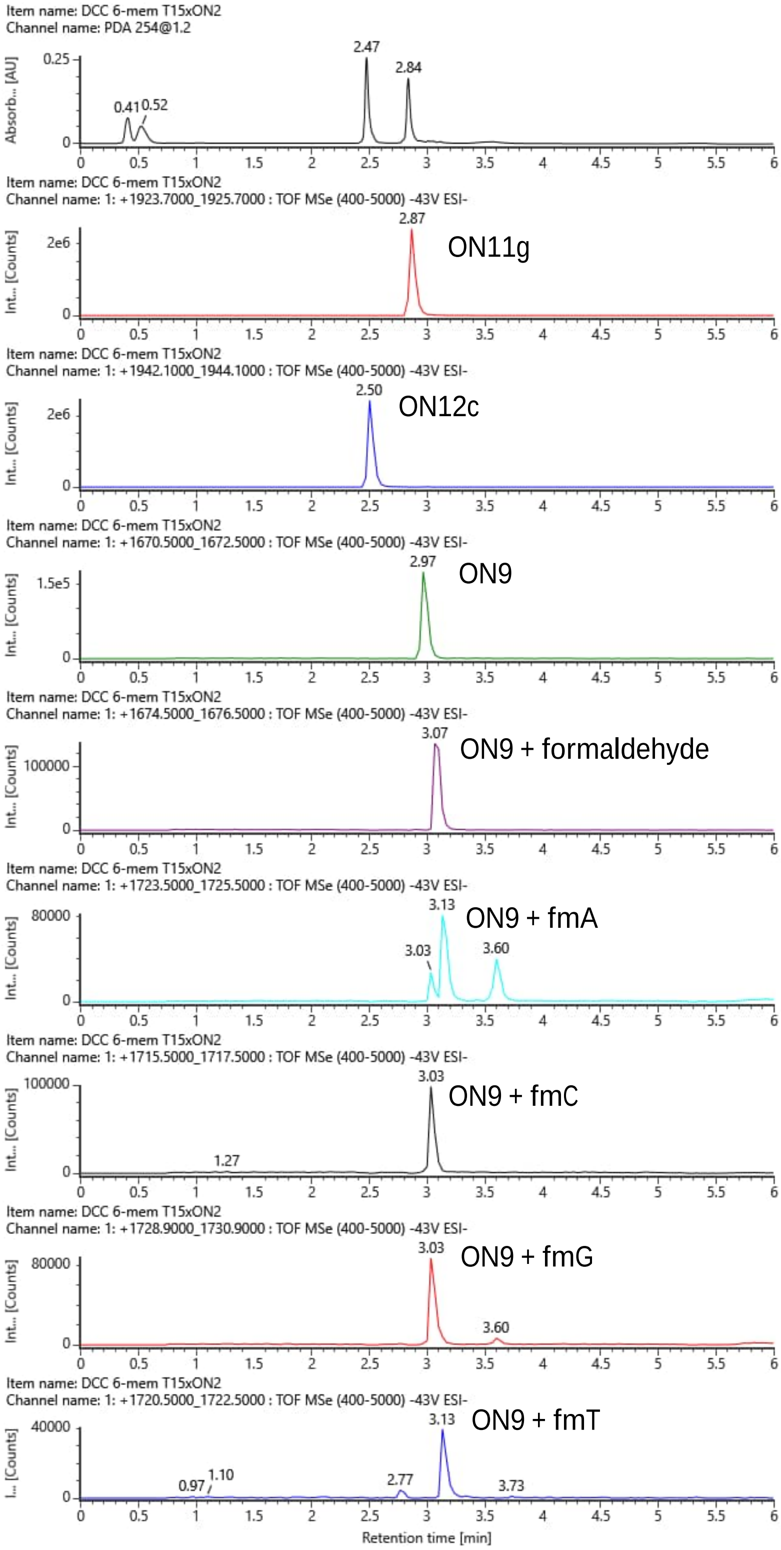

Figure S112. UV (top panel) and extracted ion RP-UPLC traces of the DCC product mixture on incubation of the oligonucleotide triplex ON11g•ON12c*ON9 (1.0 µM) with a mixture of aldehydes fmA, fmC, fmG, fmT and fmB (2.0 µM) at 23 °C, pH = 5.5 (20 mM cacodylate buffer) and $I(NaClO_4)$ = 0.10 M for 120 h; ACQUITY Premier OST column (50 × 2.1 mm, 1.7 µm); flow rate 0.4 mL min^{-1}; linear gradient (5—25% over 4 min) of MeOH in aqueous solution of hexafluoroisopropanol (40 mM) and triethylamine (7 mM); λ = 254 nm; T = 60 °C.

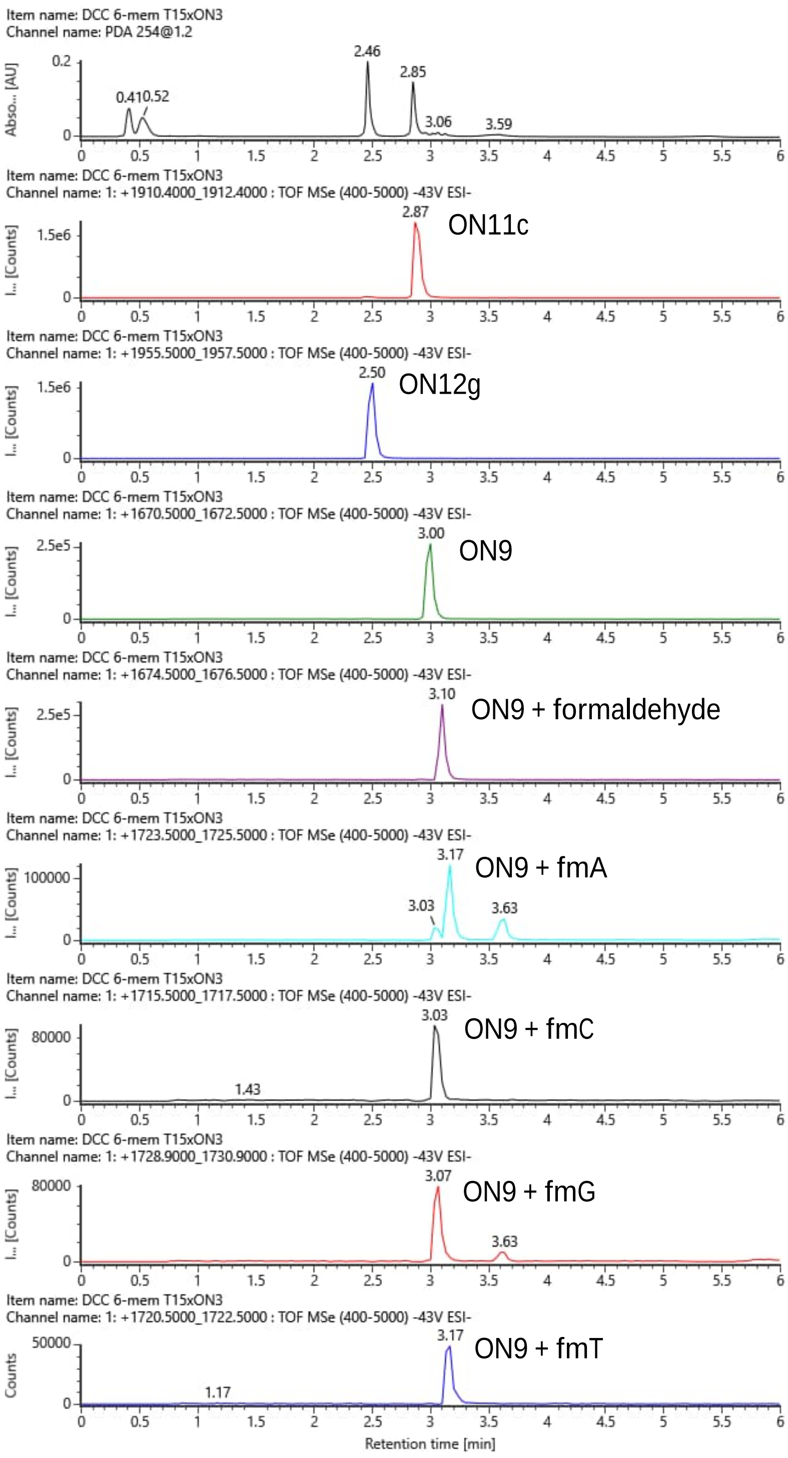

Figure S113. UV (top panel) and extracted ion RP-UPLC traces of the DCC product mixture on incubation of the oligonucleotide triplex ON11c•ON12g*ON9 (1.0 µM) with a mixture of aldehydes fmA, fmC, fmG, fmT and fmB (2.0 µM) at 23 °C, pH = 5.5 (20 mM cacodylate buffer) and $I(NaClO_4)$ = 0.10 M for 120 h; ACQUITY Premier OST column (50 × 2.1 mm, 1.7 µm); flow rate 0.4 mL min^{-1}; linear gradient (5—25% over 4 min) of MeOH in aqueous solution of hexafluoroisopropanol (40 mM) and triethylamine (7 mM); λ = 254 nm; T = 60 °C.

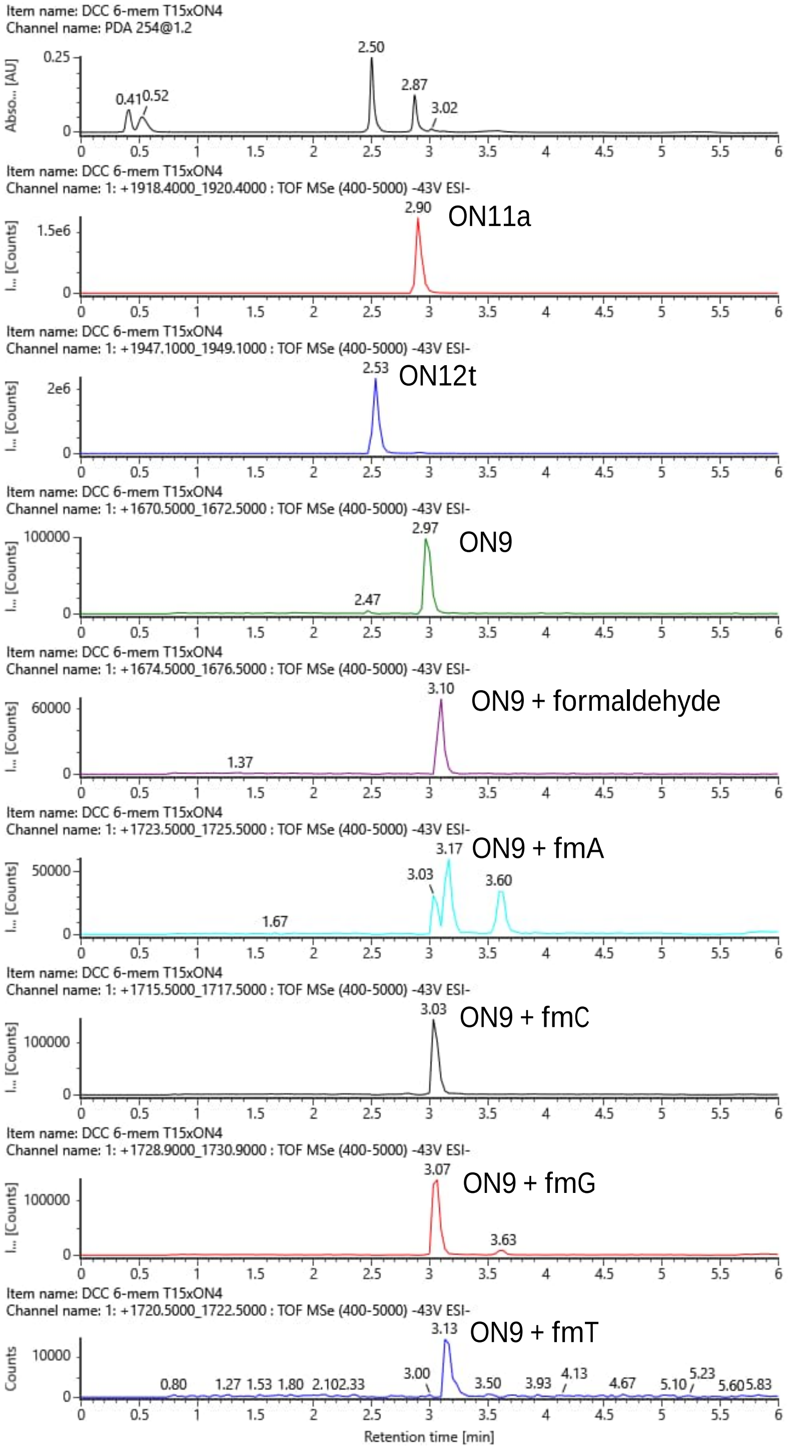

Figure S114. UV (top panel) and extracted ion RP-UPLC traces of the DCC product mixture on incubation of the oligonucleotide triplex ON11a•ON12t*ON9 (1.0 µM) with a mixture of aldehydes fmA, fmC, fmG, fmT and fmB (2.0 µM) at 23 °C, pH = 5.5 (20 mM cacodylate buffer) and $I(NaClO_4)$ = 0.10 M for 120 h; ACQUITY Premier OST column (50 × 2.1 mm, 1.7 µm); flow rate 0.4 mL min^{-1}; linear gradient (5—25% over 4 min) of MeOH in aqueous solution of hexafluoroisopropanol (40 mM) and triethylamine (7 mM); λ = 254 nm; T = 60 °C.

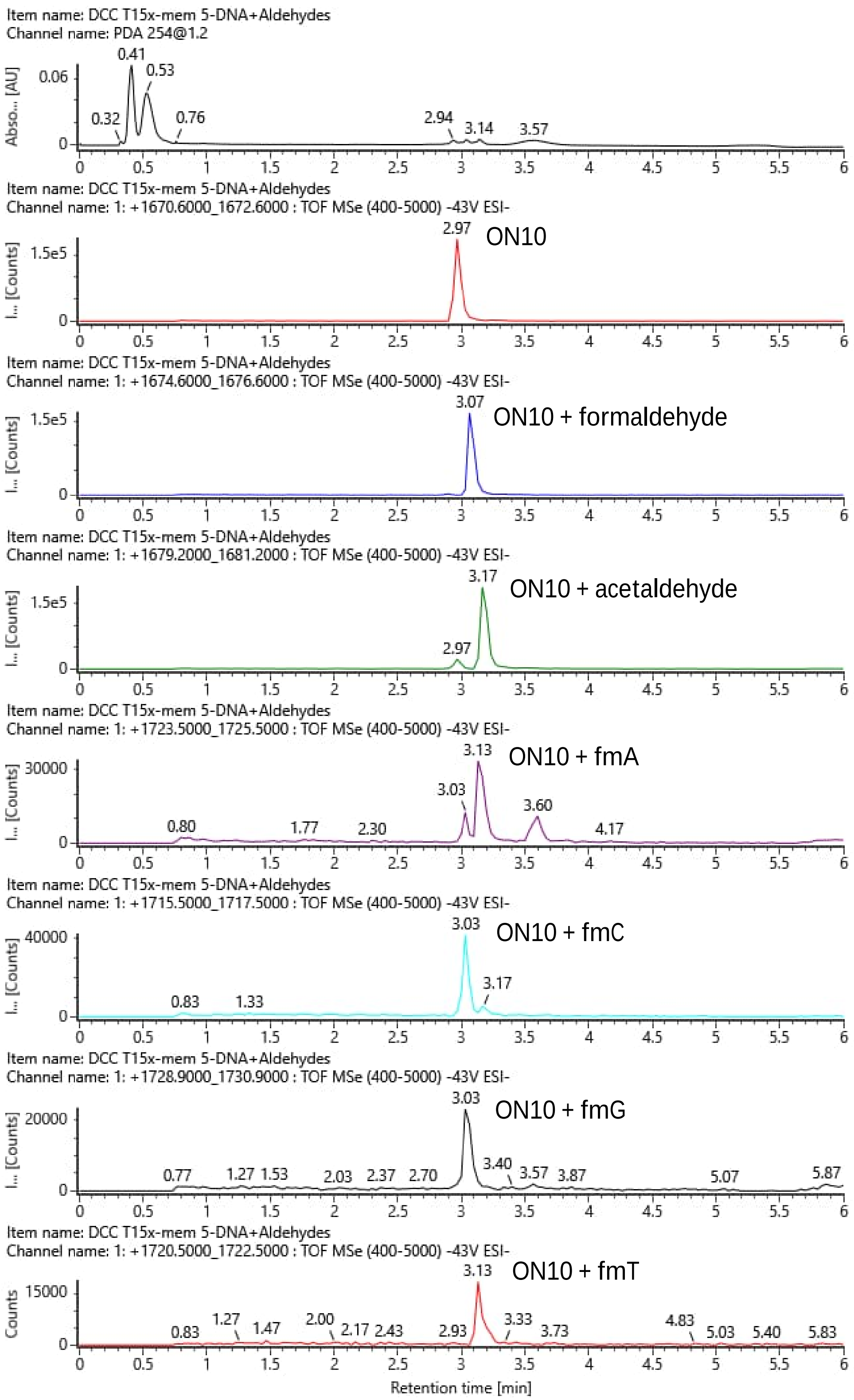

Figure S115. UV (top panel) and extracted ion RP-UPLC traces of the DCC product mixture on incubation of the single-stranded oligonucleotide ON10 (1.0 µM) with a mixture of aldehydes fmA, fmC, fmG, fmT and fmB (20 µM) at 23 °C, pH = 5.5 (20 mM cacodylate buffer) and *I*($NaClO_4$) = 0.10 M for 120 h; ACQUITY Premier OST column (50 × 2.1 mm, 1.7 µm); flow rate 0.4 mL min^{-1}; linear gradient (5—25% over 4 min) of MeOH in aqueous solution of hexafluoroisopropanol (40 mM) and triethylamine (7 mM); λ = 254 nm; *T* = 60 °C.

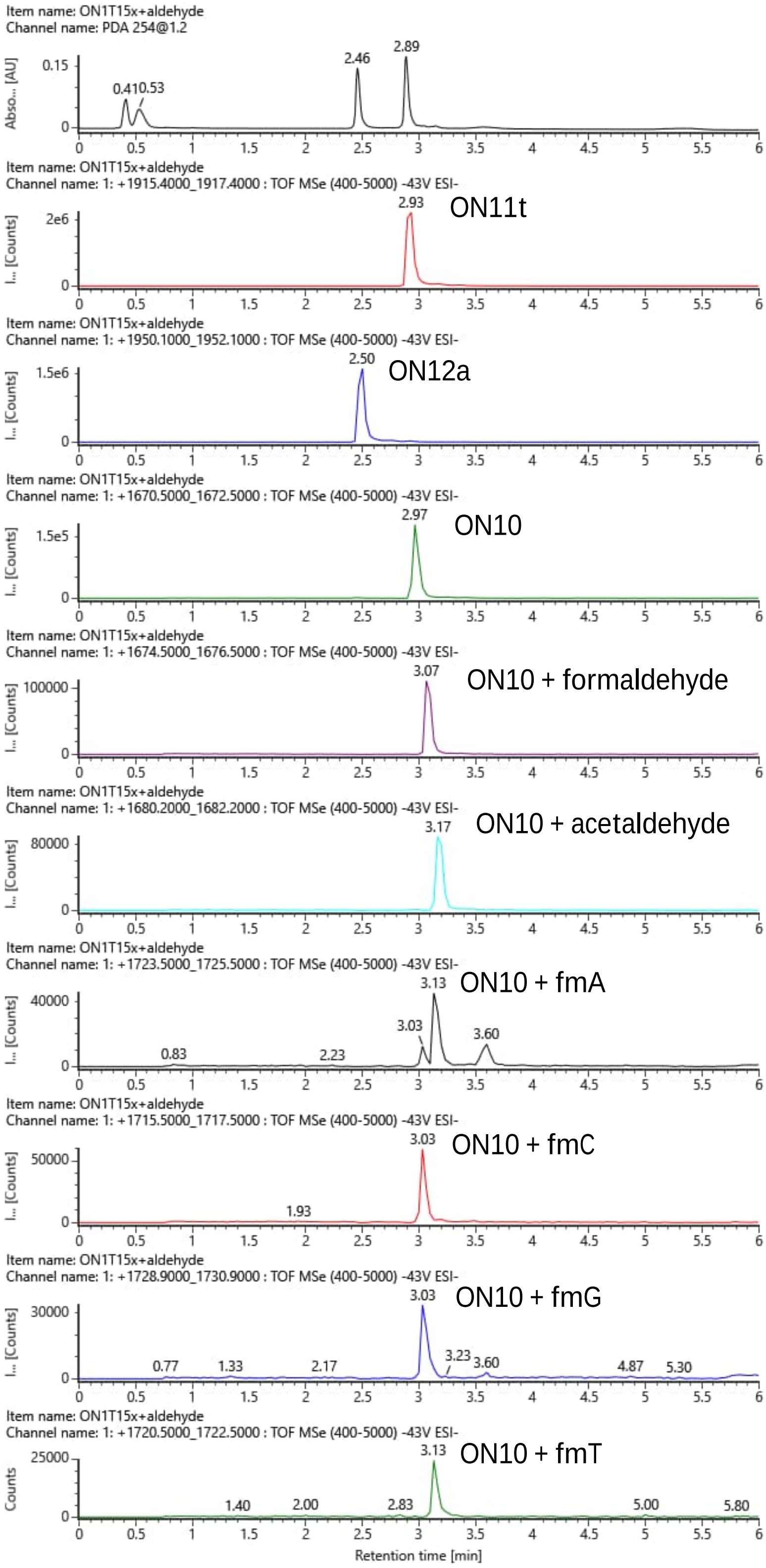

Figure S116. UV (top panel) and extracted ion RP-UPLC traces of the DCC product mixture on incubation of the oligonucleotide triplex ON11t•ON12a*ON10 (1.0 µM) with a mixture of aldehydes fmA, fmC, fmG, fmT and fmB (2.0 µM) at 23 °C, pH = 5.5 (20 mM cacodylate buffer) and $I(NaClO_4)$ = 0.10 M for 120 h; ACQUITY Premier OST column (50 × 2.1 mm, 1.7 µm); flow rate 0.4 mL min^{-1}; linear gradient (5—25% over 4 min) of MeOH in aqueous solution of hexafluoroisopropanol (40 mM) and triethylamine (7 mM); λ = 254 nm; T = 60 °C.

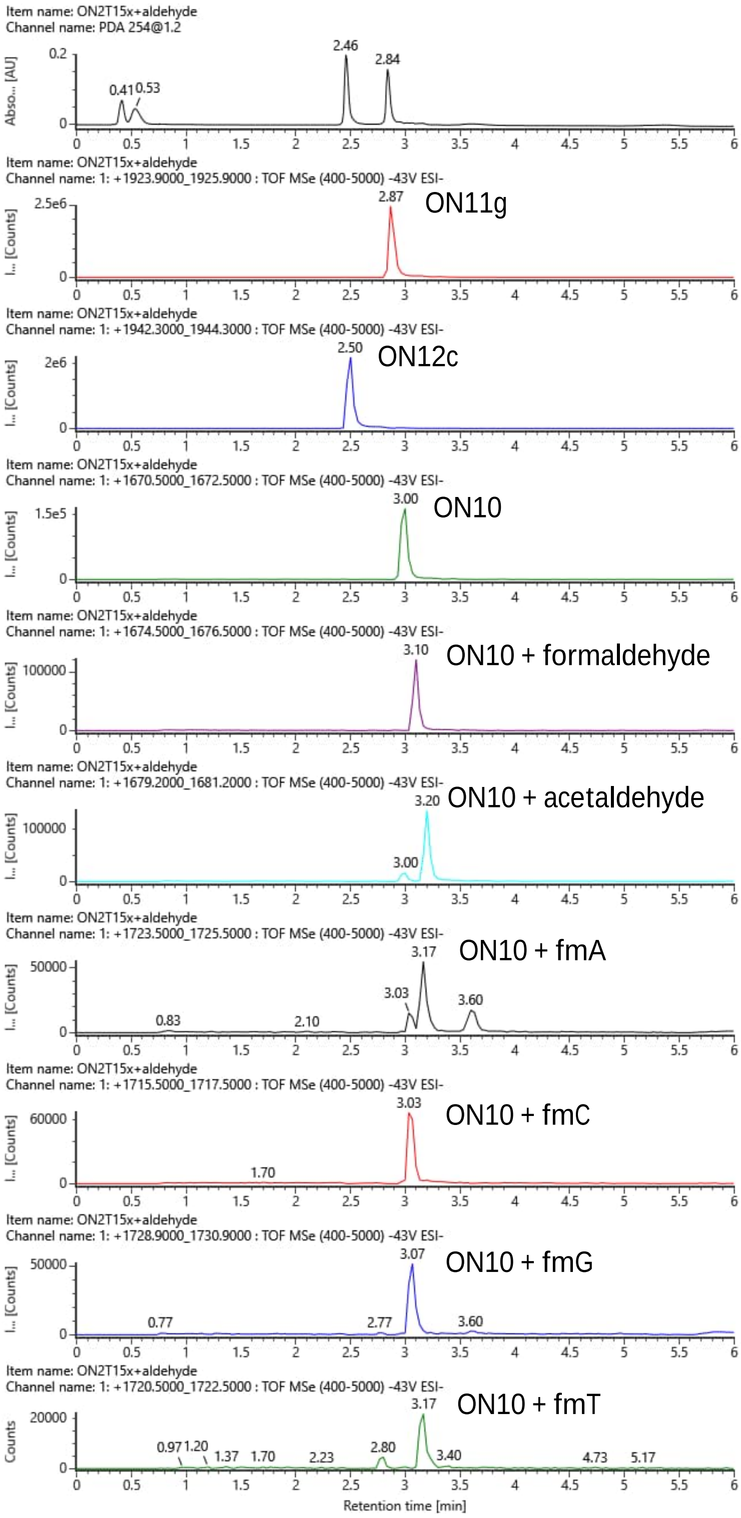

Figure S117. UV (top panel) and extracted ion RP-UPLC traces of the DCC product mixture on incubation of the oligonucleotide triplex ON11g•ON12c*ON10 (1.0 µM) with a mixture of aldehydes fmA, fmC, fmG, fmT and fmB (2.0 µM) at 23 °C, pH = 5.5 (20 mM cacodylate buffer) and $I(NaClO_4)$ = 0.10 M for 120 h; ACQUITY Premier OST column (50 × 2.1 mm, 1.7 µm); flow rate 0.4 mL min^{-1}; linear gradient (5—25% over 4 min) of MeOH in aqueous solution of hexafluoroisopropanol (40 mM) and triethylamine (7 mM); λ = 254 nm; T = 60 °C.

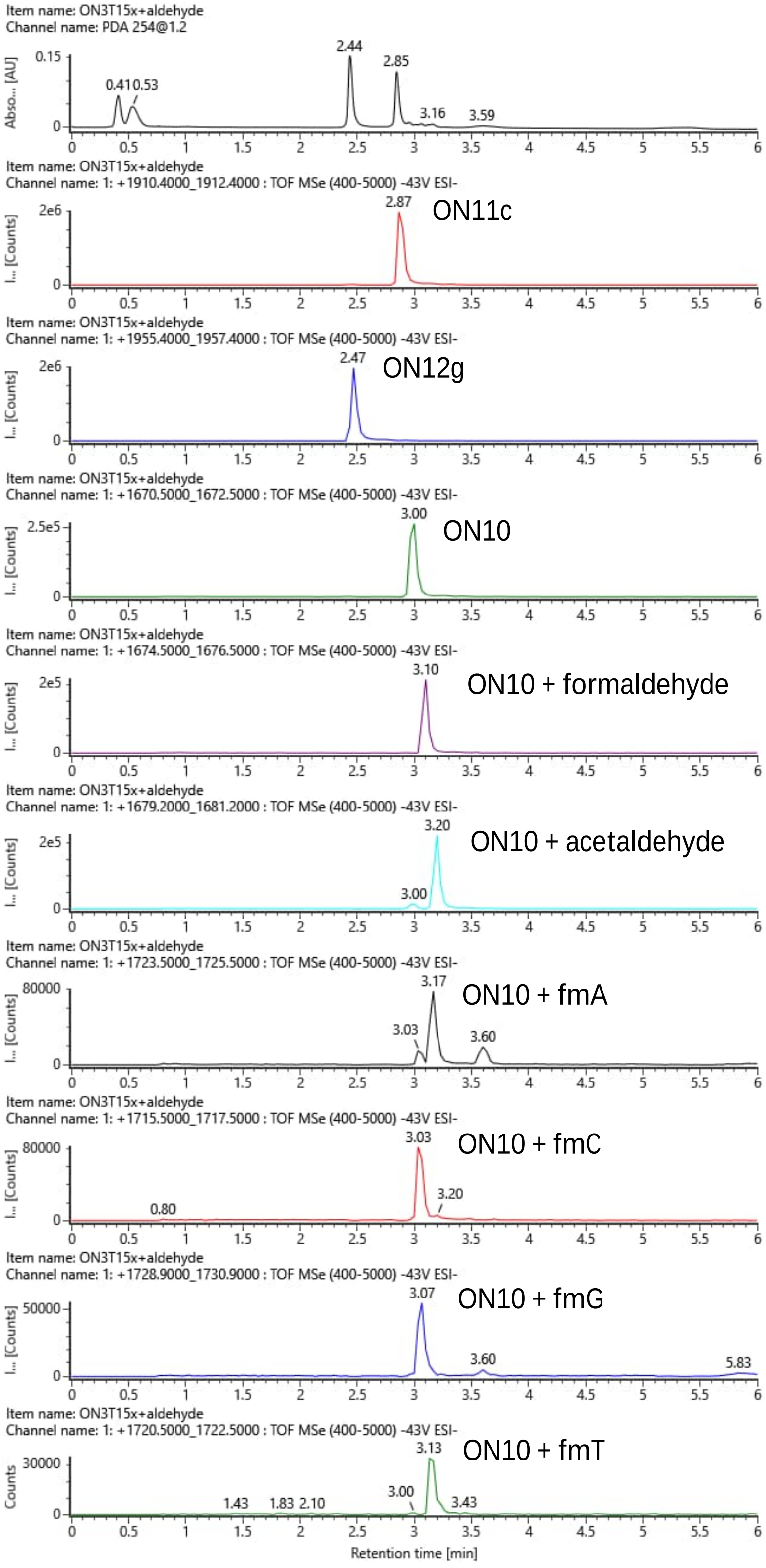

Figure S118. UV (top panel) and extracted ion RP-UPLC traces of the DCC product mixture on incubation of the oligonucleotide triplex ON11c•ON12g*ON10 (1.0 µM) with a mixture of aldehydes fmA, fmC, fmG, fmT and fmB (2.0 µM) at 23 °C, pH = 5.5 (20 mM cacodylate buffer) and $I(NaClO_4)$ = 0.10 M for 120 h; ACQUITY Premier OST column (50 × 2.1 mm, 1.7 µm); flow rate 0.4 mL min^{-1}; linear gradient (5—25% over 4 min) of MeOH in aqueous solution of hexafluoroisopropanol (40 mM) and triethylamine (7 mM); λ = 254 nm; T = 60 °C.

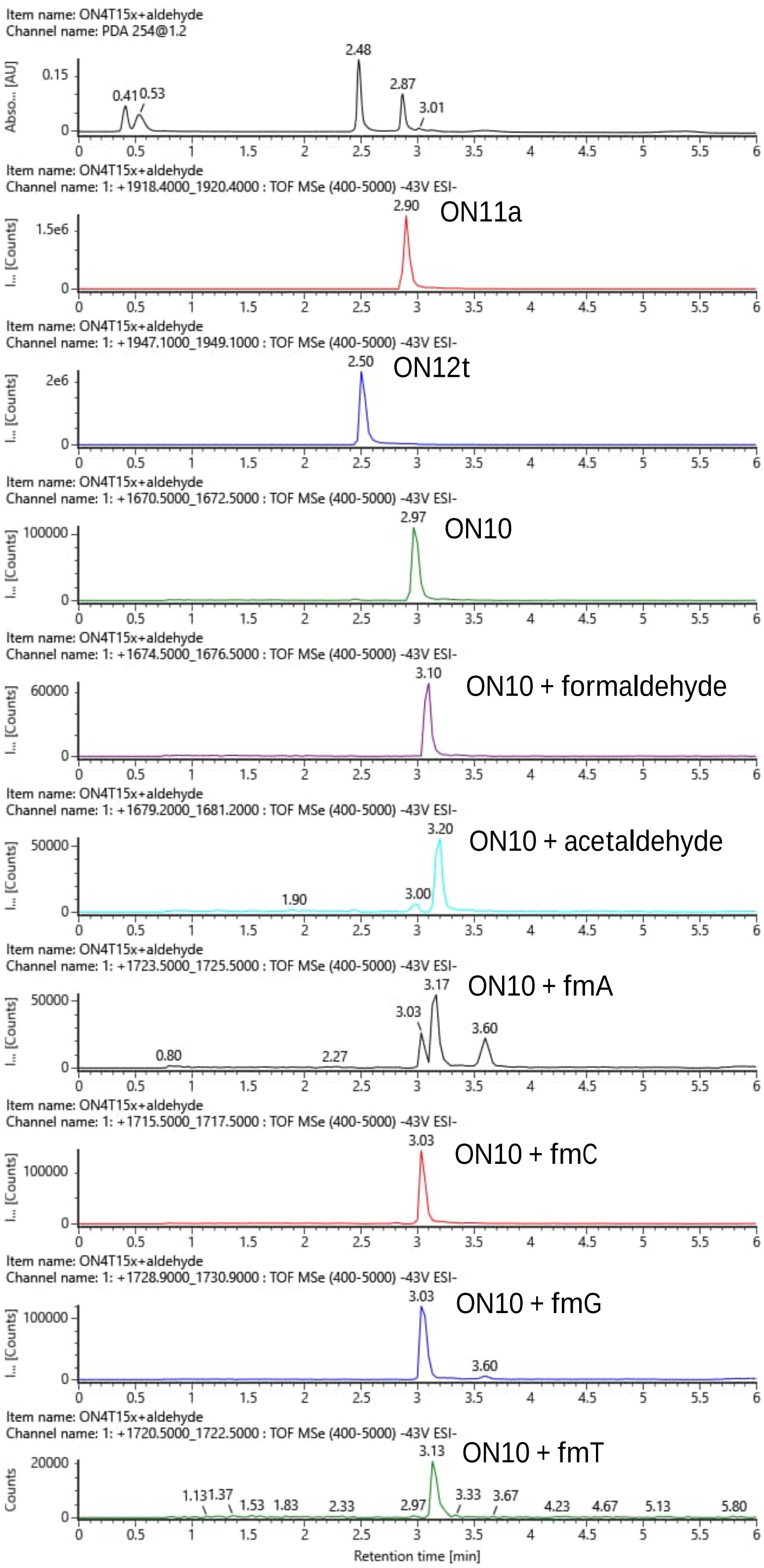

Figure S119. UV (top panel) and extracted ion RP-UPLC traces of the DCC product mixture on incubation of the oligonucleotide triplex ON11a•ON12t*ON10 (1.0 µM) with a mixture of aldehydes fmA, fmC, fmG, fmT and fmB (2.0 µM) at 23 °C, pH = 5.5 (20 mM cacodylate buffer) and *I*($NaClO_4$) = 0.10 M for 120 h; ACQUITY Premier OST column (50 × 2.1 mm, 1.7 µm); flow rate 0.4 mL min^{-1}; linear gradient (5—25% over 4 min) of MeOH in aqueous solution of hexafluoroisopropanol (40 mM) and triethylamine (7 mM); λ = 254 nm; T = 60 °C.

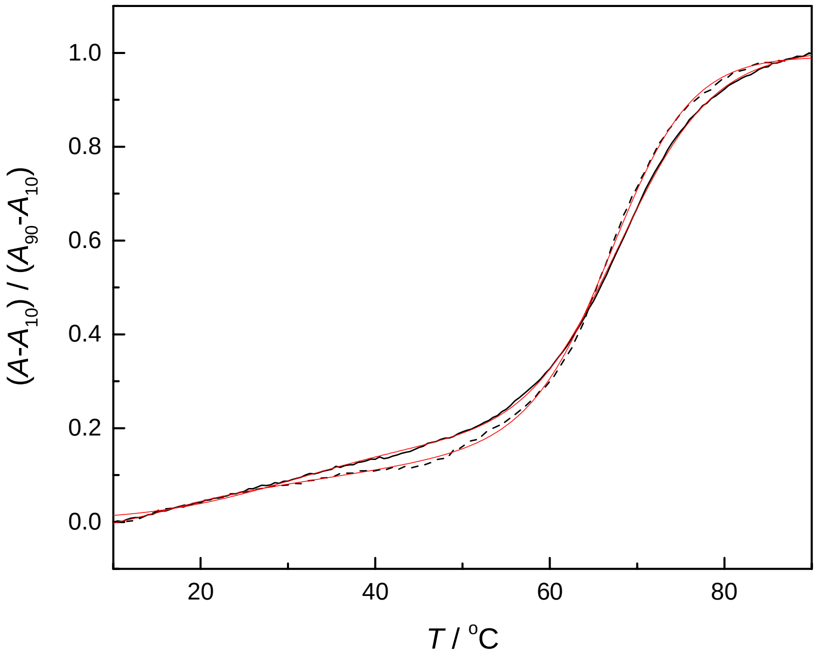

Figure S120. UV melting profile of 1.0 µM hairpin ON2a naked (dashed line) and functionalized with aldehyde fmA (solid line); pH = 7.4 (20 mM cacodylate buffer); $I(NaClO_4)$ = 0.10.

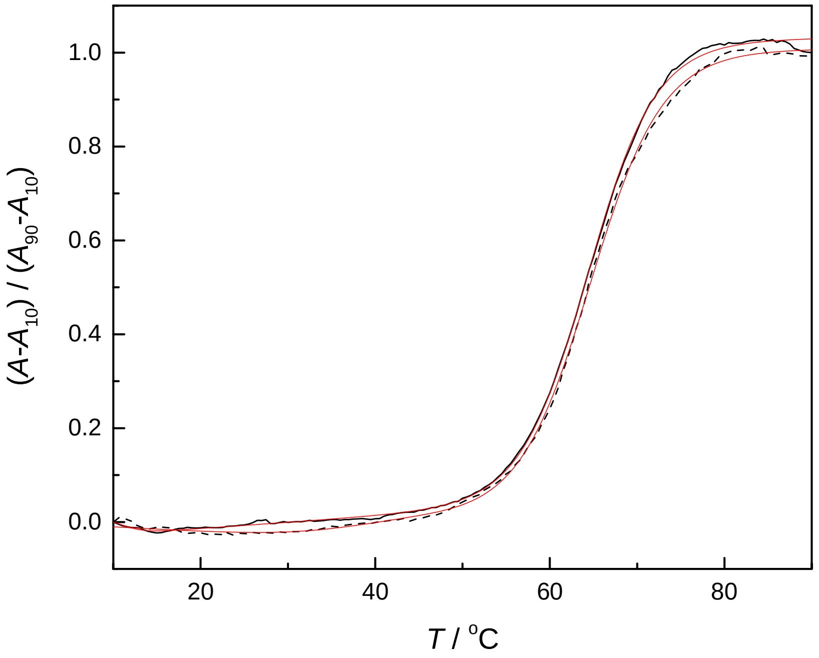

Figure S121. UV melting profile of 1.0 µM hairpin ON2c naked (dashed line) and functionalized with aldehyde fmA (solid line); pH = 7.4 (20 mM cacodylate buffer); $I(NaClO_4)$ = 0.10.

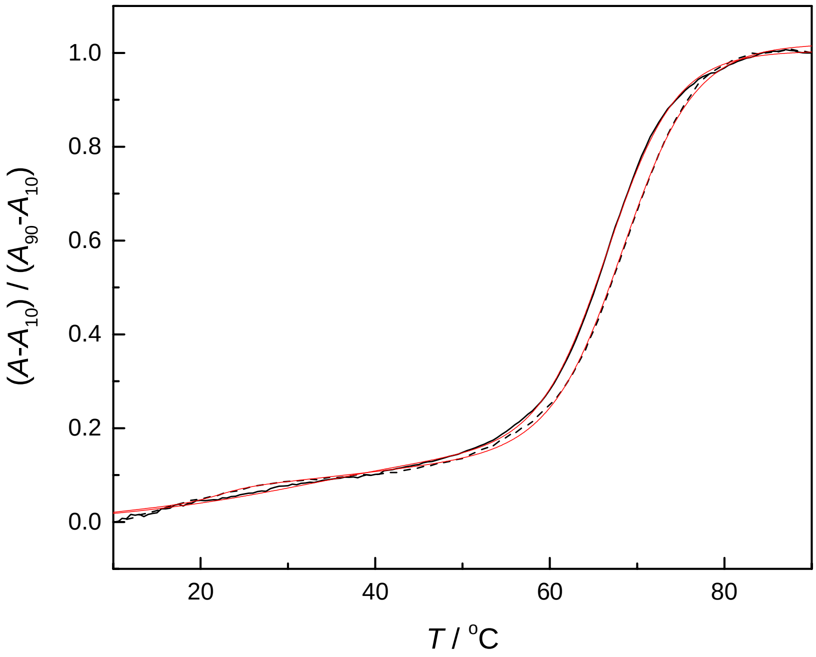

Figure S122. UV melting profile of 1.0 µM hairpin ON2g naked (dashed line) and functionalized with aldehyde fmA (solid line); pH = 7.4 (20 mM cacodylate buffer); *I*($NaClO_4$) = 0.10.

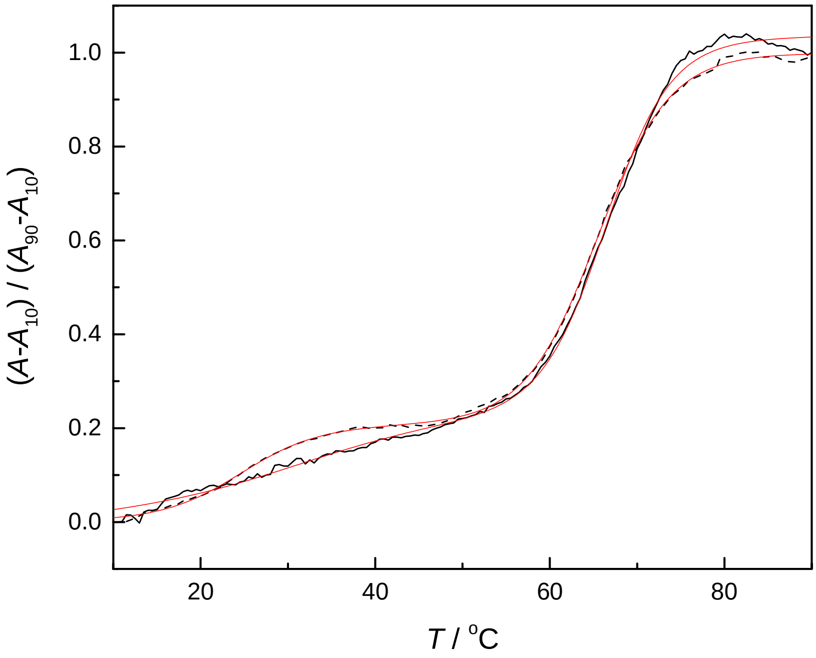

Figure S123. UV melting profile of 1.0 µM hairpin ON2t naked (dashed line) and functionalized with aldehyde fmA (solid line); pH = 7.4 (20 mM cacodylate buffer); *I*($NaClO_4$) = 0.10.

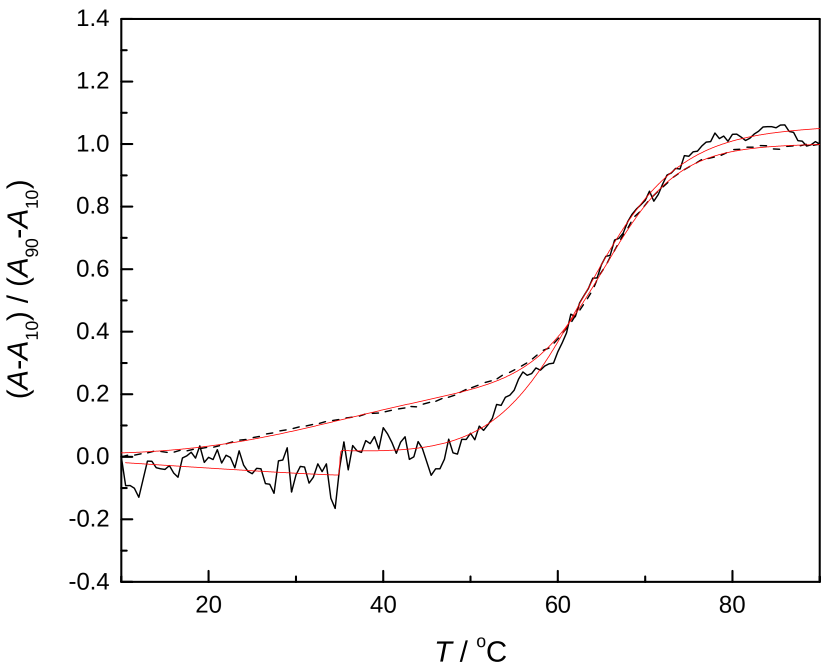

Figure S124. UV melting profile of 1.0 µM hairpin ON2s naked (dashed line) and functionalized with aldehyde fmA (solid line); pH = 7.4 (20 mM cacodylate buffer); *I*($NaClO_4$) = 0.10.

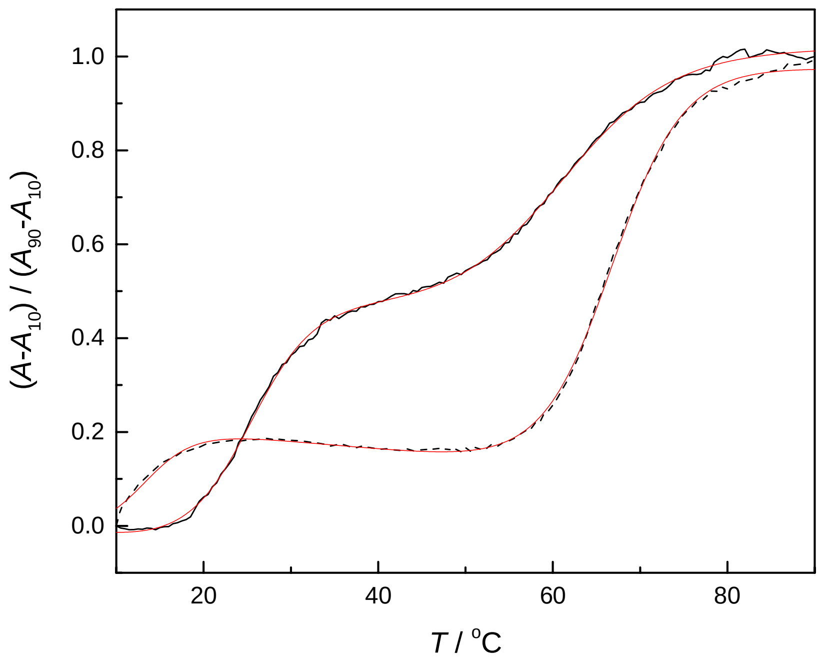

Figure S125. UV melting profile of 1.0 µM hairpin ON4a naked (dashed line) and functionalized with aldehyde fmA (solid line); pH = 7.4 (20 mM cacodylate buffer); $I(NaClO_4)$ = 0.10.

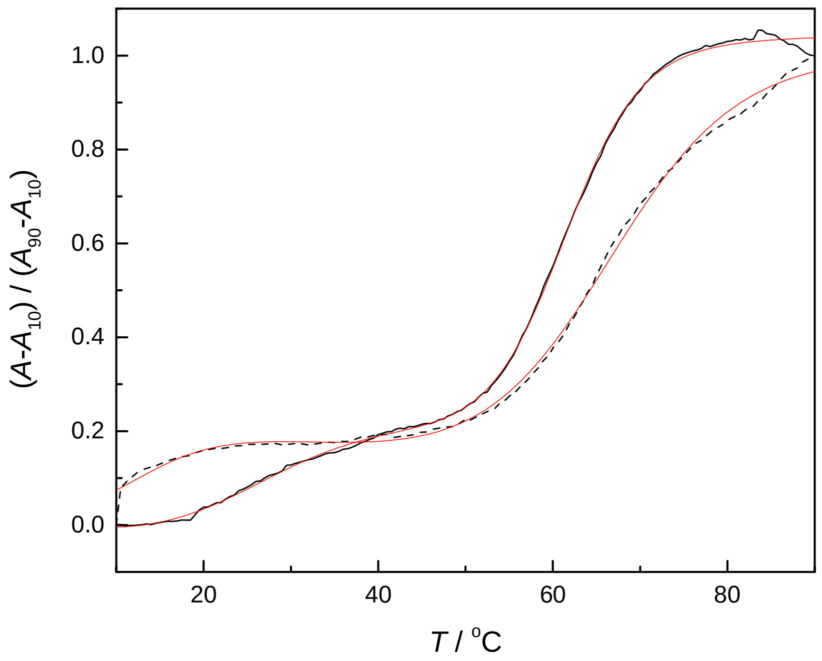

Figure S126. UV melting profile of 1.0 µM hairpin ON4c naked (dashed line) and functionalized with aldehyde fmA (solid line); pH = 7.4 (20 mM cacodylate buffer); $I(NaClO_4)$ = 0.10.

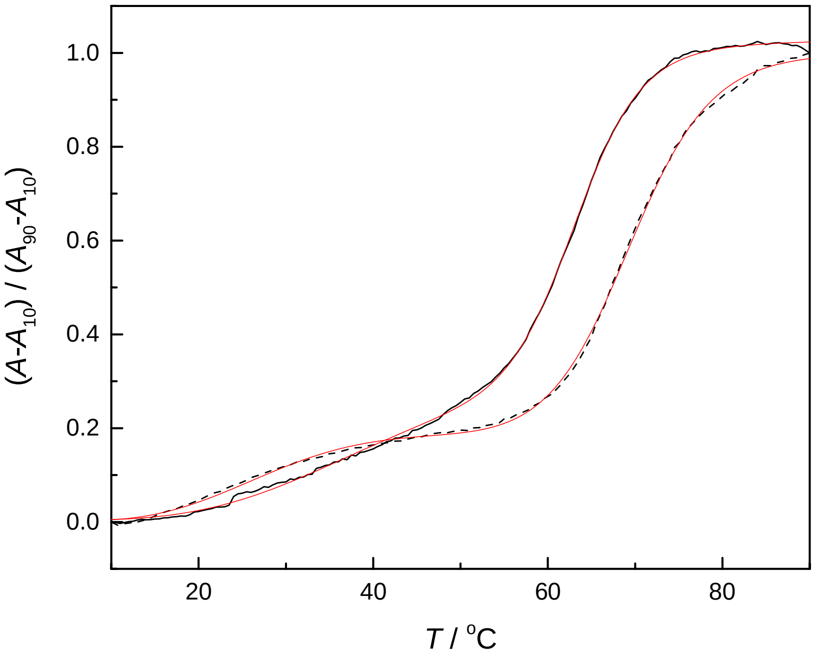

Figure S127. UV melting profile of 1.0 µM hairpin ON4g naked (dashed line) and functionalized with aldehyde fmA (solid line); pH = 7.4 (20 mM cacodylate buffer); $I(NaClO_4)$ = 0.10.

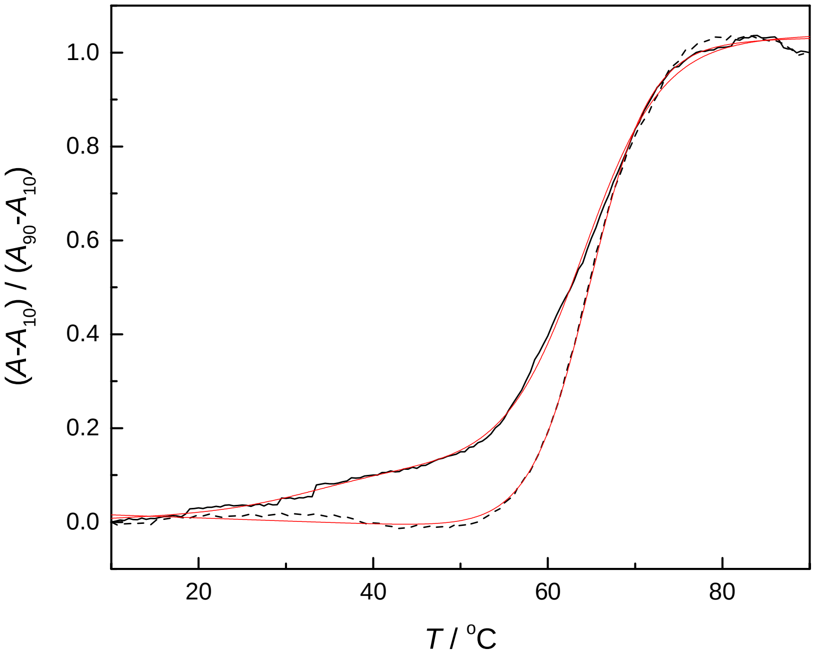

Figure S128. UV melting profile of 1.0 µM hairpin ON4t naked (dashed line) and functionalized with aldehyde fmA (solid line); pH = 7.4 (20 mM cacodylate buffer); $I(NaClO_4)$ = 0.10.

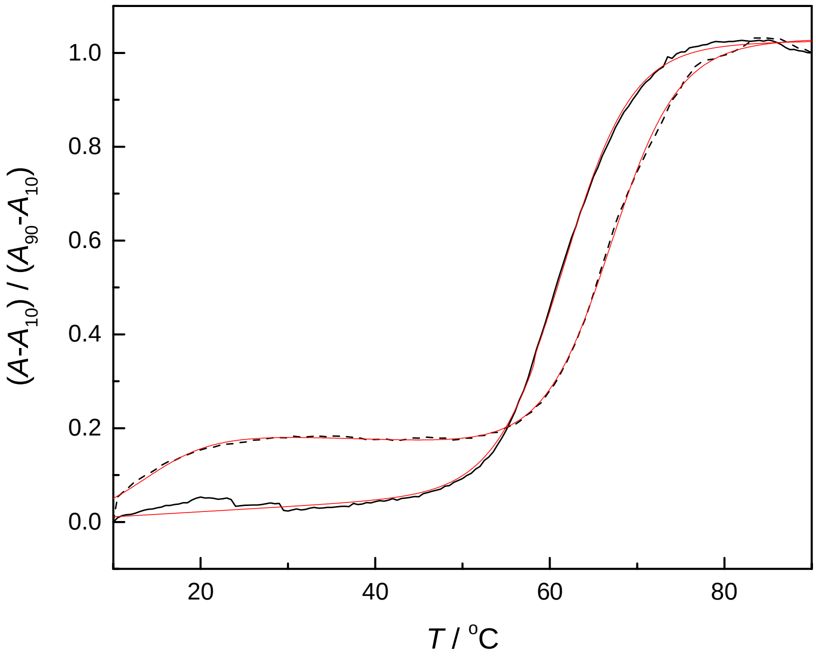

Figure S129. UV melting profile of 1.0 µM hairpin ON4s naked (dashed line) and functionalized with aldehyde fmA (solid line); pH = 7.4 (20 mM cacodylate buffer); *I*($NaClO_4$) = 0.10.

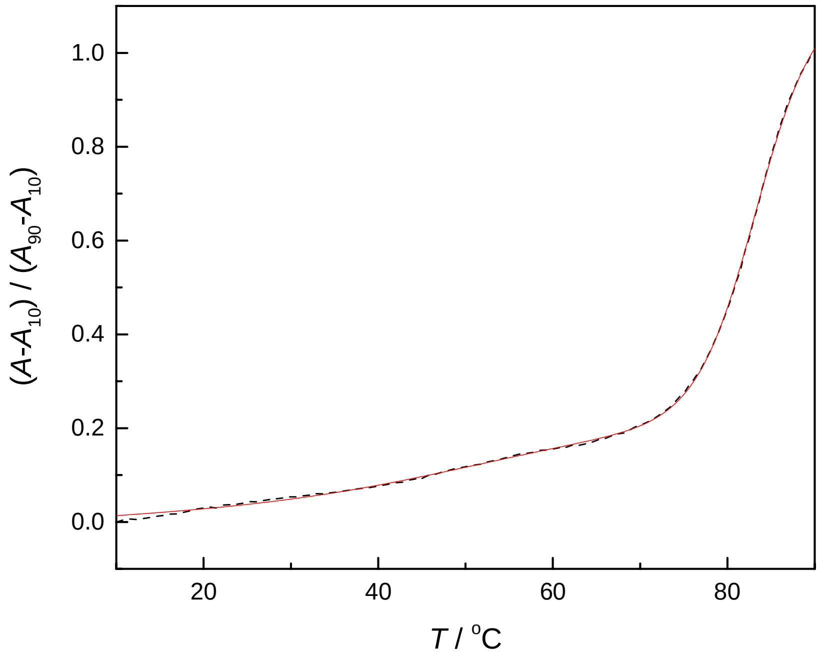

Figure S130. UV melting profile of 1.0 μM hairpin ON6a; pH = 7.4 (20 mM cacodylate buffer); *I*($NaClO_4$) = 0.10.

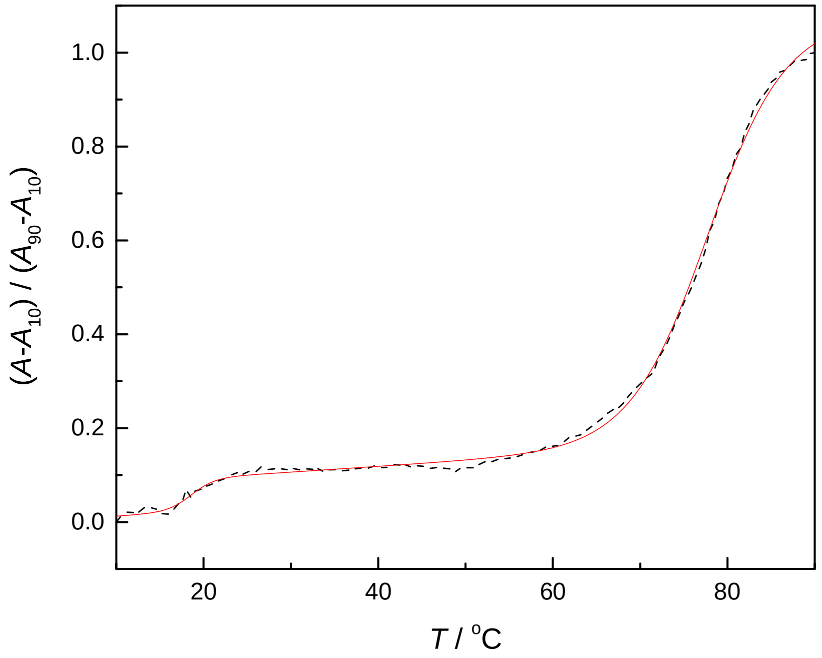

Figure S131. UV melting profile of 1.0 μM hairpin ON6c; pH = 7.4 (20 mM cacodylate buffer); *I*($NaClO_4$) = 0.10.

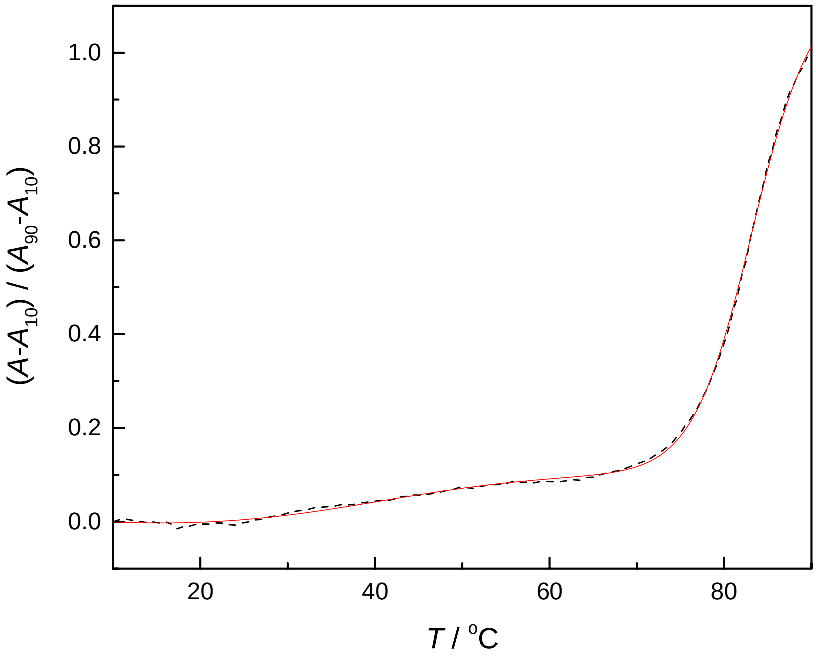

Figure S132. UV melting profile of 1.0 µM hairpin ON6g; pH = 7.4 (20 mM cacodylate buffer); *I*($NaClO_4$) = 0.10.

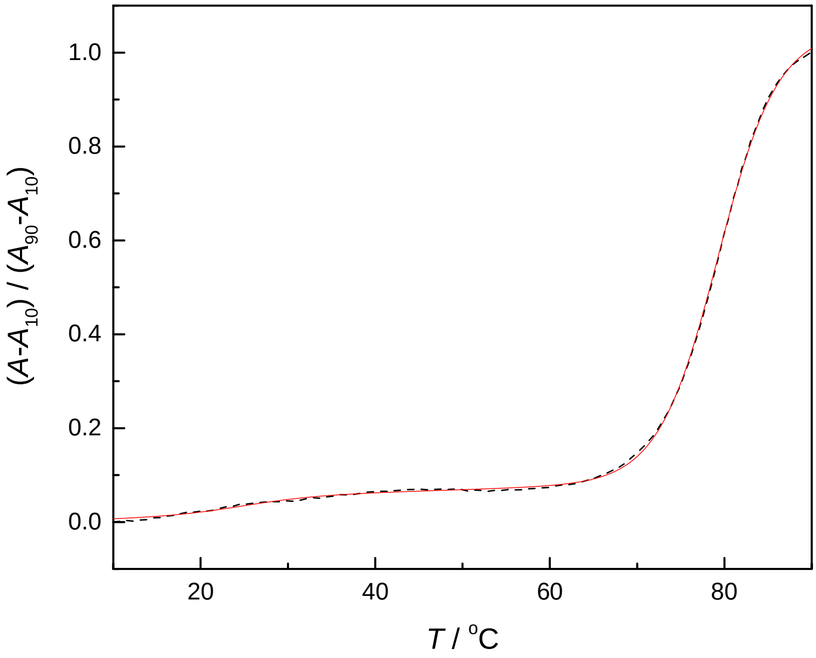

Figure S133. UV melting profile of 1.0 µM hairpin ON6u; pH = 7.4 (20 mM cacodylate buffer); *I*($NaClO_4$) = 0.10.

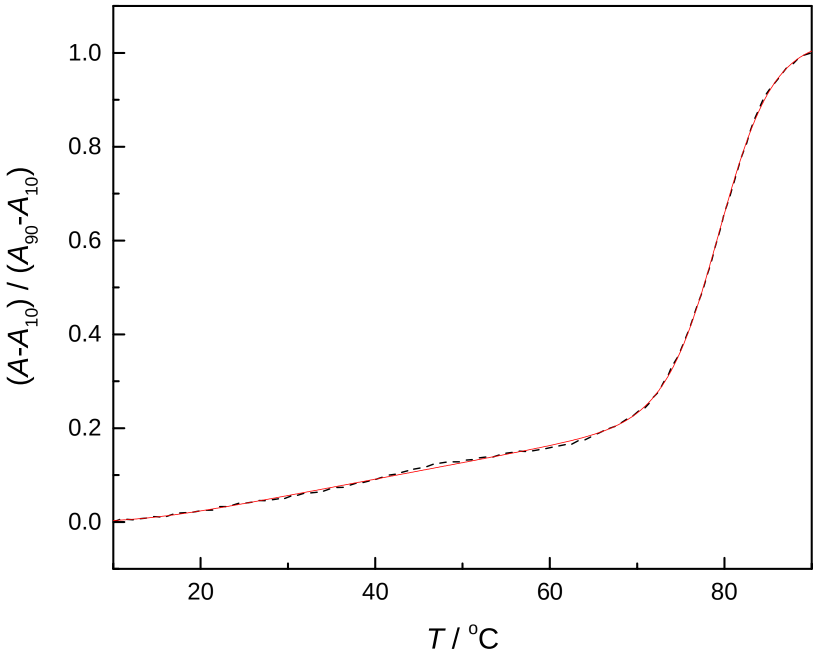

Figure S134. UV melting profile of 1.0 µM hairpin ON6s; pH = 7.4 (20 mM cacodylate buffer); *I*($NaClO_4$) = 0.10.

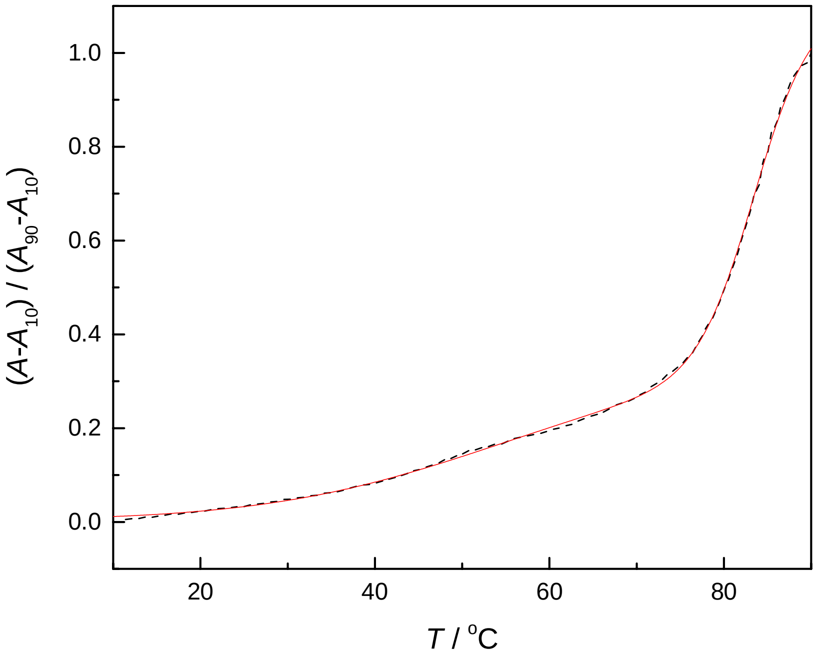

Figure S135. UV melting profile of 1.0 µM hairpin ON8a; pH = 7.4 (20 mM cacodylate buffer); *I*($NaClO_4$) = 0.10.

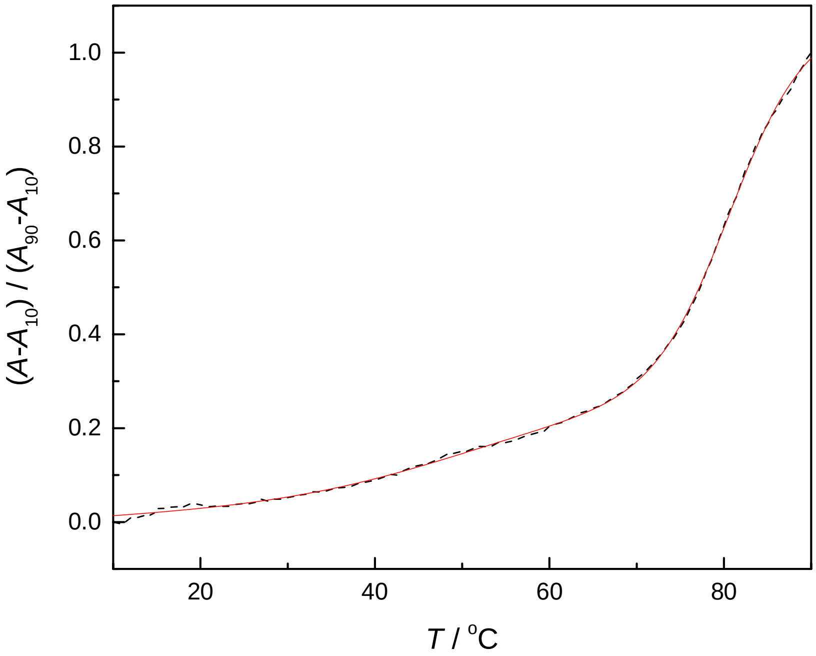

Figure S136. UV melting profile of 1.0 µM hairpin ON8c; pH = 7.4 (20 mM cacodylate buffer); *I*($NaClO_4$) = 0.10.

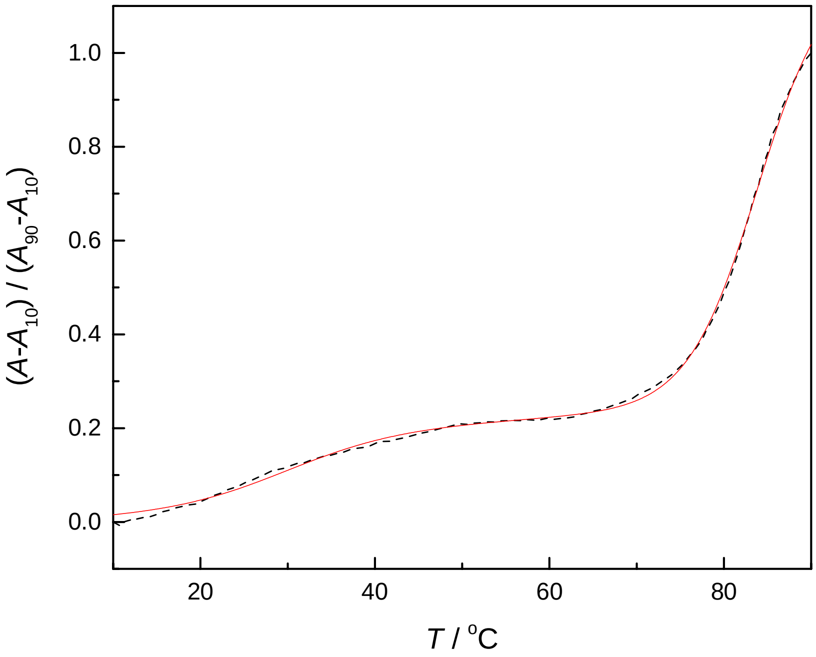

Figure S137. UV melting profile of 1.0 µM hairpin ON8g; pH = 7.4 (20 mM cacodylate buffer); *I*($NaClO_4$) = 0.10.

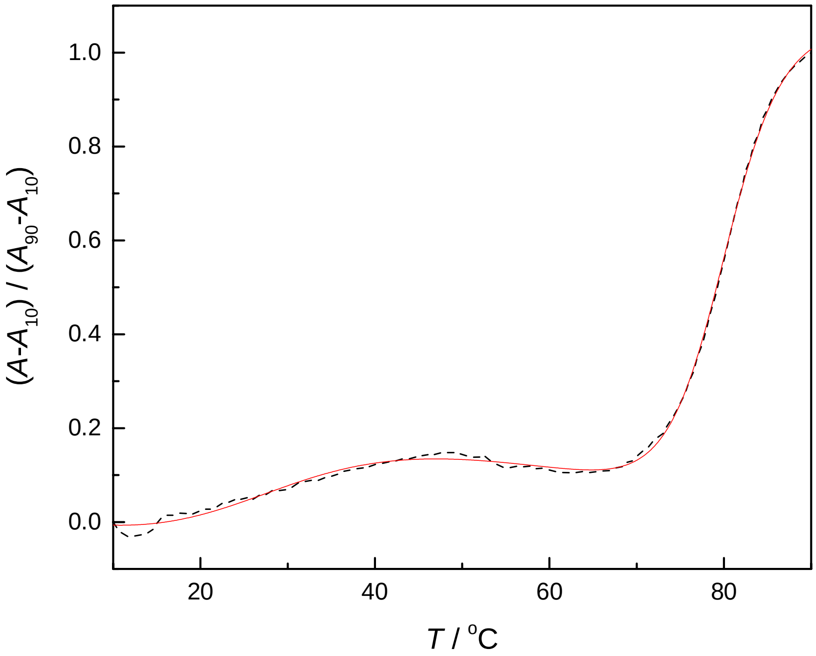

Figure S138. UV melting profile of 1.0 µM hairpin ON8u; pH = 7.4 (20 mM cacodylate buffer); *I*($NaClO_4$) = 0.10.

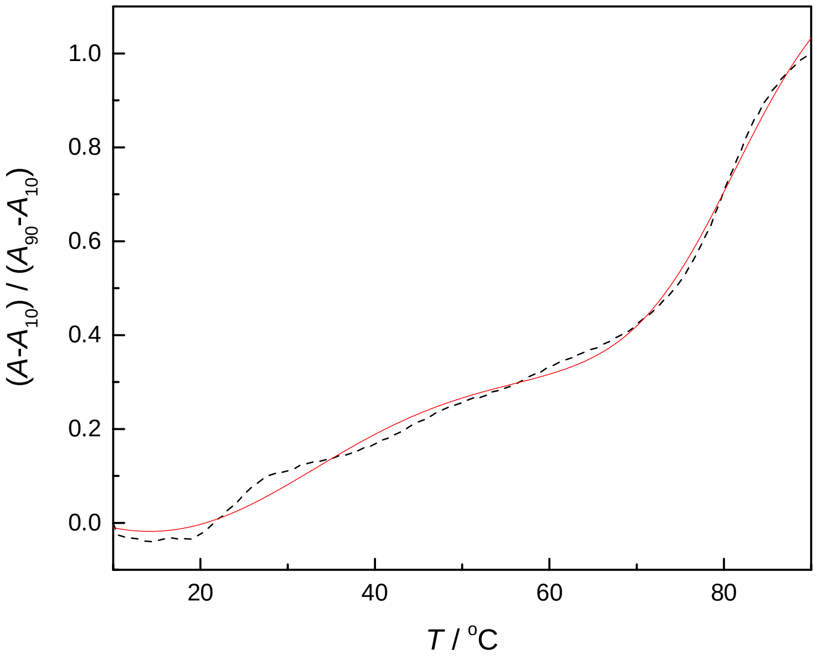

Figure S139. UV melting profile of 1.0 µM hairpin ON8s; pH = 7.4 (20 mM cacodylate buffer); *I*($NaClO_4$) = 0.10.
